# Perceptions of the parents of deceased children and of healthcare providers about end-of-life communication and breaking bad news at a tertiary care public hospital in India: A qualitative exploratory study

**DOI:** 10.1371/journal.pone.0248661

**Published:** 2021-03-18

**Authors:** Manoja Kumar Das, Narendra Kumar Arora, Harish Kumar Chellani, Pradeep Kumar Debata, K. R. Meena, Reeta Rasaily, Gurkirat Kaur, Prikanksha Malik, Shipra Joshi, Manisha Kumari

**Affiliations:** 1 The INCLEN Trust International, New Delhi, India; 2 Department of Pediatrics, Safdarjung Hospital and Vardhman Mahavir Medical College, New Delhi, India; 3 Division of Reproductive Biology Maternal and Child Health, Indian Council of Medical Research, New Delhi, India; ESIC Medical College & PGIMSR, INDIA

## Abstract

**Background:**

Parents of dying children face unique challenge and expect compassionate support from health care providers (HCPs). This study explored the experiences of the parents and HCPs about the end-of-life care and breaking bad news and related positive and negative factors in Indian context.

**Methods:**

This qualitative exploratory study was conducted at paediatrics department of a tertiary care hospital in Delhi. In-depth interviews with the parents (n = 49) and family members (n = 21) of the children died at the hospital and HCPs (6 doctors, 6 nurses and 4 support staffs) were conducted. Also events and communication around death of eight children were observed. Data were inductively analysed using thematic content analysis method to identify emerging themes and codes.

**Results:**

Doctors were the lead communicators. Majority of parents perceived the attitude, communication and language used as by resident doctors as brief, insensitive and sometimes inappropriate or negative. They perceived that the attitude and communication by senior doctor’s as empathetic, positive and complete. Parents recalled the death declaration by resident doctors as non-empathetic, blunt and cold. Most parents received no emotional support from HCPs during and after death of their child. All doctors expressed that death of their patients affected them and their emotions, which they coped through different activities. The overcrowded wards, high workload, infrastructural limitation and no formal communication training added to the emotional stress of the HCPs.

**Conclusions:**

Majority of the communication by the HCPs during the hospitalisation and end-of-life period were perceived as suboptimal by the parents. The HCPs were emotionally affected and faced end-of-life communication challenges. The study highlights the communication by HCPs and support for parents during the end-of-life communication and breaking bad news. It suggests adoption of context specific communication protocol and materials and training of HCPs in communication to improve the quality of care.

## Author summary

The parents of terminally ill and dying children face unimaginably psychological stress and have strong need for compassionate professional support. To improve the parents and family experience, there is need for obtaining feedback and documenting the experiences of parents, family and healthcare providers with reference to the local contexts. There is limited information on parents experience on end-of-life communication related to their child death from Indian context. An exploratory qualitative study was conducted at a tertiary care hospital in India involved in-depth interviews with parents and family of deceased children and healthcare providers (HCPs: doctors, nurses and support staffs) involved in treating children. Parents remembered the communication by junior doctors as brief, inappropriate and insensitive, while the senior doctor’s communication was positive and complete. The death declaration by resident doctors was remembered as non-empathetic, blunt and cold. No emotional support was received by them around and after child’s death. The doctors were affected emotionally with death of the patients, but were constrained with high workload, infrastructural limitations and lack of training in communication. Preparation of context specific, sociocultural appropriate module and communication training for HCPs can improve their competence for providing end-of-life care, breaking bad news and also parents experience.

## Introduction

Parents experience unimaginably intense painful emotional and social crisis during the death of their child [1]. Most of them have lifelong or long lasting impact of the event, irrespective of the cause of death [1]. Additionally, the parents also undergo immense anxiety and psychological and physical stress during the serious illnesses, when things don’t look better [2]. During the terminal phases of illnesses, several times they are confronted with the unknowns and are challenged to make difficult decisions, e.g. initiation or withdrawal of life-support measures, referrals, finances, etc. [3] Care of the child during serious and terminal illness is most challenging for the parents. The healthcare providers (HCPs) also undergo stressful situations while dealing with the seriously sick or terminally ill children [4, 5]. Breaking bad news including prognostication and death declaration are challenging situations for the treating doctors, which almost all doctors face in their clinical practice. Breaking the news to the parents and family that their child is anticipated to die prematurely is one of the most difficult aspects of child healthcare.

The efforts, attitude, behaviour and communication by the HCPs have impact on the acceptance and response from the parent and family. While in majority of the instances, the parents and family accept the bad news, there are instances of unpleasant reactions and violence against the HCPs and hospitals [6]. A study from India reported families dissatisfaction with the services, miscommunication and ineffective communication and death as the important reasons following the non-availability of medicines, facilities and manpower challenges in healthcare services [7].

Support from the HCPs and hospital during the terminal event and after child death have positive impact on the parent’s long-term grieving outcomes [8]. The perceptions of parents and challenges faced during the terminal phase, especially for the children in intensive care unit (ICU) include loss of parental role, lack of physical contact and not being present with the their child during the terminal phase and ambivalence in decision making [9]. Reviews of articles on parents’ perspectives regarding the end-of-life care for their children have identified poor or lack of communication and information, strained relationships, inadequate emotional support, their desire to maintain parent-child relationships in life and death, quality of care (QoC), influence of services, and the difficult decisions to terminate life support, as the key themes [10, 11]. The common challenges identified in the studies were insufficient communication, lack of respect and lack of emotional support for parents [9–11].

Several frameworks and models for breaking bad news have been proposed [12, 13]. Patient-centred communication is most positively accepted and perceives the physicians as most emotional and least dominant while breaking bad news [14]. Thus, it is essential for the clinicians to understand the parent’s experiences around the serious illnesses including the end-of-life and their needs to provide good QoC. These end-of-life and death declarations have implications for success of the after-life activities like organ donation and autopsy or minimally invasive tissue sampling (MITS) for establishing the exact causes of death, when needed. The post-mortem MITS is a suitable alternative for complete diagnostic autopsy for establishing cause of death across all ages [15–17].

A pilot project on MITS to identify causes of child death and stillbirths was initiated in India. To gain insight about the events around child death and stillbirths, a formative research explored the perceptions of the parents and HCPs regarding the end-of-life care and death declaration for children and also observed the death declaration processes. The findings were used to inform the consent process for MITS. This article presents data from the formative research and focuses on the perceptions and experiences of the parents of deceased children and HCPs regarding the end-of-life communication including death declaration to inform the clinical practices.

## Methods

### Study design and setting

This formative research conducted at a tertiary care hospital in Delhi (where the MITS pilot study was undertaken) during September 2018 to April 2019 used exploratory multi-method qualitative design. This government hospital has 300 bedded paediatric medicine ward, 20 bedded paediatric ICU (PICU) and 25 bedded neonatal ICU (NICU). It is a major referral hospital for Delhi and nearby areas. The paediatric ward (60 bedded) is managed by 16 doctors (3 senior and 10 junior residents) and 18 nurses. The PICU is usually managed by 10 doctors (3 senior and 7 junior residents) and 20 nurses. The NICU is usually managed by 14 doctors (6 senior and 8 junior residents) and 25 nurses. The faculty and senior doctors are available during the day time and on call. The routine round-the-clock care is provided by the resident doctors and nurses.

### Study participants

The study participants included: (i) parents and family members of deceased under-five children including neonates; and (ii) HCPs (doctors, nurses and support staffs). The parents of the children who died in the hospital during August-October 2018 were contacted after 6–8 weeks of the unfortunate event. The parents from outside Delhi were excluded. Out of the 97 children died during the reference period, 39 were untraceable and 58 eligible parents were approached. Out of these approached, parents of 13 deceased children and 12 deceased neonates consented for IDI. The HCPs including the doctors (junior residents, senior residents and faculties), nurses and support staffs from children and neonatology wards were purposively selected. Observation of events and the communications between the HCPs (doctors, nurses and staffs) and parents of critically sick and dying children (till departure from hospital) were undertaken for supplementation and triangulation. The parents of the children who participated in the IDI were different from the observation of events around the children died in the hospital. These participants were informed and consent was obtained.

### Data collection

Guides for in-depth interview (IDI) contained open-ended questions targeting the objectives and issues identified from literature (S1 File). The IDI guides for parents focused on their experiences and perceptions about the end-of-life care, overall attitude, communication and behaviour of the HCPs. The IDI guides for HCPs focused on the experiences around end-of-life care, support systems and challenges faced while breaking bad news. The IDIs were finalised after piloting. Four female researchers (GK, PM, SJ, and MK, qualification MPH and PhD) trained in qualitative research and interviews conducted the IDIs at homes (parents) or hospital chamber (HCPs). The IDIs included participants with different sociocultural background and were continued till data saturation. After data saturation 2–3 additional IDIs were conducted. Senior researcher (lead investigator) with long standing experience in qualitative research and maternal and child health and health systems research (MKD, paediatrician) supervised the research staffs. During the IDIs, presence of other persons was avoided. The IDIs were conducted in local language (Hindi) and were audio-recorded with consent. During the observations at hospital, the communications between healthcare providers (doctors, nurses and hospital staffs) and parents/family members and the activities done, non-verbal expressions were documented without interference (S1 File). No audio recording for the observations was done. The same research staff conducted IDIs and observations and data collection continued simultaneously. Detailed field notes captured the verbal and non-verbal expressions of the participants. The median time taken for the IDIs was about 60 (45–90) minutes and for the death observations was 90 minutes (60–150) minutes.

### Data handling and analysis

The field notes and audio recordings were transcribed verbatim in local language by two researchers followed by translation into English (S2 and S4 Files). Two other members checked the transcriptions quality with the audio-records and completed/corrected as needed. The data was entered using INCLEN Qualitative Data Analysis Software (IQDAS), which allows data entry, organization and retrieval for analysis in Indian languages and English, and saved into the server with daily back-up. The transcripts were independently read by two researchers and inductively analyzed following the steps: free listing, domain identification, axial and selective coding and cross tabulation. The emerged codes and themes were regularly discussed and discrepancies were resolved. These codes were reviewed for organization under the axial and selective codes under the key themes. Reiterative processes of transcript reading, coding and thematic summarization were adopted till the investigators agreed on the thematic framework. The themes across the participant categories were triangulated to check consistencies and differing perspectives. The findings were summarized for semi-quantitative expressions: almost all (>90%), most (76–89%), majority (50–75%), less-than half (25–49%), some (10–24%) and very few (<10%). The detailed protocol for the complete formative research has been published earlier [18].

### Ethical considerations

The study protocol was approved by the participating institute ethics committees (The INCLEN Trust International, Ref: IIEC 51 and V.M.M.C. and Safdarjung Hospital, Ref: IEC/SJH/VMMC/Project/August-2017/1000). The participants were recruited and in-depth interviews were conducted after obtaining written informed consent including permission for audio recording and publication without identifiers. Confidentiality and anonymity in data handling were assured.

## Results

From households of 25 deceased children (12 neonates and 13 children), 49 parents and 21 family members (mostly grandparents) participated. Sixteen HCPs (equally from paediatrics and neonatology units) also participated in the study. Table 1 shows the characteristics of the participants participated and observations included in the study.

**Table 1:** Characteristics of the study participants.

Sl no	Parameters	Results
*1*	*Parents and family members*	
1.1	Age, average (range) in years	
	• Mother (n = 25)	25 (17–35)
	• Father (n = 24)	30 (22–45)
	• Other family members (n = 21)	55 (48–64)
1.2	Religion (n = 25)	
	• Hindu	16 (64)
	• Muslim	8 (32)
	• Christian	1 (4)
1.3	Mother’s literacy (n = 25)	
	• <5^th^ standard	5 (20)
	• 6^th^ -10^th^ standard	9 (36)
	• >10^th^ standard	11 (44)
1.4	Father’s literacy (n = 24)	
	• <5^th^ standard	5 (20.8)
	• 6^th^ -10^th^ standard	8 (33.3)
	• >10^th^ standard	11 (45.8)
1.5	Other family members (n = 21)	
	• <5^th^ standard	5 (23.8)
	• 6^th^ -10^th^ standard	8 (38.1)
	• >10^th^ standard	8 (38.1)
1.6	Mother’s occupation (n = 25)	
	• Housewife	24 (96.0)
	• Working (skilled worker)	1 (4.0)
1.7	Father’s occupation (n = 24)	
	• Skilled worker	10 (41.7)
	• Self-employed or business	12 (50.0)
	• Daily wage laborer	2 (8.3)
1.8	Other family members (n = 21)	
	• Skilled worker	2 (9.5)
	• Self-employed or business	5 (23.8)
	• Daily wage laborer	1 (4.8)
	• Not working	13 (61.9)
*2*	*Healthcare providers*	
2.1	Profession (n = 16)	
	• Doctors (junior residents 2; senior residents 2; faculty 2; one each from Pediatrics and Neonatology units)	6
	• Nurses (three each from Pediatrics and Neonatology units)	6
	• Support staffs (two each from Pediatrics and Neonatology units)	4
2.2	Gender, n	
	• Female	9
	• Male	7
2.3	Age group, average (range) in years	
	• Faculty members	47 (45–53)
	• Resident doctors	28 (26–29)
	• Nurses	44 (37–51)
	• Support staffs	41 (33–48)
2.4	Service tenure, average (range) in years	
	• Faculty members	14 (14–20)
	• Residents	2.5 (2–4)
	• Nurses	15 (14–21)
	• Support staffs	14 (13–16)
*3*	*End-of-life care observations*	
3.1	Patient category (n = 8)	
	• Neonates	4
	• Children	4
3.2	Family members present	
	• Father	7
	• Mother	4
	• Grandparent(s)	3
	• Other family member(s)	3

Inductive data analysis identified six overarching themes: (1) communication during hospital care; (2) process of death declaration; (3) respect and dignity for patient’s family; (4) emotional support for family; (5) experience of services and care received; and (6) experience of the HCPs. The findings along with the illustrative statements from participants are presented below. The coding tree for these domains is attached as supplementary document (S4 File).

### Communication during hospital care

The communication theme included perception of the attitude, body language and wordings of the HCPs during interaction with the parents and family prior to terminal event. Some of the parents recalled the communication by the doctors to be empathetic and appropriate. Almost all parents perceived the senior doctors had positive attitude, provided more complete information and used sympathetic and appropriate languages. Majority of parents perceived the communication, body language and wordings used by the resident doctors to be inappropriate, insensitive and sometimes negative. Several parents stated that the doctors blamed them for their child’s condition (not taking appropriate care, delay). Majority of the respondents perceived the communications by resident doctors about the status and prognosis as brief, inadequate and blunt. Some of the parents expressed unhappiness with the behaviour and languages used by the nurses. Some of the parents mentioned that the status communicated by different HCPs were conflicting, included English language and medical terms and gave false hope when the outcome was evident.

“*We call the doctor to see out child and asked for hot steam (nebulisation)*. *But they forcibly inserted emergency oxygen pipe (intubated)*, *attached a balloon (bag) and asked us to keep on pressing*. *And removed the hot steam*. *That is what killed our child*.*” (Father- 11103)*

“*The doctors used to tell about the child daily*. *They (doctors) used to tell in English usually*, *which I couldn’t understand*. *One doctor was there who was not speaking Hindi well*. *He used to tell me*, *but we couldn’t understand well*.*” (Mother- 11104)*

### Process of death declaration

According to the parents, all of the deaths were declared by the resident doctors. The deaths occurred at different times during the day and night. Some recalled the death declaration process as very brief and the doctors soon left the bedside. The attitude and language used by the doctors were non-empathetic, blunt and cold. Most of the parents mentioned that the doctors declared death to the male members present and asked them to take the mother away before the declaration. Parents of two children stated that the doctors delayed death declaration because of incomplete records. Few parents mentioned that they were called over phone to inform the death of their child, as no family member was present in the ICU.

“*They came*, *saw and told that injection for increasing BP was given*. *After 10 minutes they came*, *checked and told that the child is dead*. *There was no empathy*. *Just told that the child was dead and left*.*” (Father- 11105)*

### Respect and dignity for patient’s family

Majority of the parents were neutral about the attitude of the treating doctors towards patient’s family members. Few of the parents whose children were admitted for longer period and in the ICU had good experience of being treated by the doctors with respect and dignity. Few respondents narrated being scolded, shouted and abused by all categories of hospital staffs during the hospitalisation period. Few parents perceived that the HCPs behaved differently with them, as they were poor. Many of the parents felt that the doctors were also under pressure of work and had to look after many sick children. Majority of them felt that the resident doctors need training on how to communicate.

“*They were good*. *From the beginning they were telling that the condition of the child is bad and giving the medicines*. *It would have been good if the child survived”*. *(Mother-11207)*

“*They used to chase me away and ask to come later*. *They said that “Saline and medicine are being given*. *You are able to see it*. *We cannot do more than this*.*” They used say this and leave*. *The language was not good and they did not have any empathy*.*” (Mother-11203)*

### Emotional support for family

Most of the parents and family did not recall any emotional consoling support after the death declaration. Few mothers recalled receiving emotional support from the nurses and female support staffs, which was appreciated by the family. Several mothers broke into tears while responding to this question.

“*The nurse was good at the hospital*. *She used to tell us how to do and not to worry*. *They only allow one person*. *But for us two people were allowed*.*” (Mother-11201)*

### Experience of services and care received

Majority of the parents reported about careless and indifferent attitude of the resident doctors. Almost all recalled that during the daily rounds, the senior doctor informed about their child’s condition and answered questions asked by them. Few parents accused the nurses for negligence in the care and medication administration. Few parents even blamed the intubation and some aggressive actions by the doctors for worsening and death of their children. Majority of the parents felt that the number of doctors and nurses were inadequate to provide appropriate care for the number of patients admitted in the wards. About half of them blamed non-availability of equipment like breathing support machines as the reason for their child’s death, as they had to ventilate their children using the breathing bags. Most of them perceived the policy of one attendant per patient to be challenging for the sick children, especially for the children on manual ventilation support, as one person couldn’t continue the activity for long. Also they felt that during the terminal phases or immediately after death declaration, the family members wished to visit the child and be with the parents to support. Some of the parents were satisfied with the kind of treatment provided to their children. Most of the parents were willing to return to the hospital again in case of need.

“*They have time to talk over phone for long time*. *When we called him*, *said I am coming*. *They used to crack jokes among them*. *Nobody was seeing the patients*.*” (Father-11113)*

“*Sometimes I feel that doctor has given some wrong medicines*. *But again I think*, *if they give wrong medicine*, *will they for only one or my child”*. *(Mother-11104)*

Y*es*. *Doctor tried to save the child and we also tried*. *But*, *when our luck was bad*, *what can we do*. *(Mother-11201)*

I *tell you*, *there were 20 patients in the ward and 2–3 staff for care (2 nurses and one doctor)*. *How can they see the 20 patients*? *(Uncle-11106)*

### Experience of the HCPs

The doctors mentioned that the condition of the child and plan of treatment were told to the parents and family when they see the patient first in the ward or in the emergency. The detailed information and prognosis sharing were usually done by the senior doctors during the ward rounds or the residents, as needed. Usually the on-duty residents declared the death. According to most of the HCPs, the families accept the death and the causes told by the doctors. In few cases the family of deceased children reacted and became aggressive, where involvement of the security guards were needed. According to them, the aggressive reactions were observed when the child worsened quickly and died. According to the HCPs, the doctors declared death usually to the father or male family members present, not the mothers. The doctors and nurses considered their workload to be quite high and due to constraints they were unable to spend time with the parents or caretakers for detailed interaction.

All doctors and nurses expressed that death declarations affected them and their emotions. The senior doctors mentioned that they have learnt over time how to handle them. The resident doctors told that they were learning and trying to cope with the challenges. According to them, the emotional affects were more when the cause of death was not clear or child worsened suddenly.

“*Initially it used to affect a lot*. *Sometimes*, *if death occurred and we couldn’t explain to ourself*, *I wasn’t able to sleep at all*. *Seeing the scenarios of life and death*, *it has changed me a lot*.*” (Doctor-Pediatrics-11702)*

“*It is very bad for us*, *no matter how rigid we appear on the face*, *but in the night when we go home*, *there is a striking force that child expired on our duty*. *Many times I am not able to sleep because of this fact*.*” (Doctor-Pediatrics-11703)*

None of the doctors and nurses reported of being formally trained in communication, breaking bad news and handling family of deceased patients. According to them they have learnt from their seniors and through experience.

“*We don’t get any specific teaching and reading materials on the part of counselling*. *It is self-reading*, *observational*, *sometimes told in classrooms*.*” (Doctor-Pediatrics; 11705)*

The doctors expressed that the sampling and cannula insertions take lots of time and limited support from the nurses. With the crowded wards and high patient load, they were time constrained for counselling and appropriate interaction with the parents and caretakers.

“*The workload on doctors is high*. *Here staff nurses don’t do any sampling*. *Occasionally put the IV cannula*. *All invasive procedures are done by doctors*.*” (Nurse-Pediatrics; 11711)*

### Observations of end-of-life and general communications

The findings from the observations of the end-of-life care and general communications in the children wards are summarised below.

#### End-of-life communication and death declaration

We observed eight deaths; four children and four neonates in the paediatric wards. At the time of death, two doctors (one senior resident and one junior resident) and at least one nurse were present. In four cases, the parents called the doctor or nurse to see the child anticipating deterioration. All the deaths were declared by the senior resident doctors followed by the cleaning and packing of the bodies by the nurses.

Following declaration of the death, the doctors left the bedside and were engaged with completion of the paperwork. After the paperwork completion, they either continued with the other parent care in the same ward or moved to other ward. The nurse with the support staff prepared for handover of the body to the parent/family member. After 20–45 minutes of death declaration, the bodies were handed over to the family. Around the death declaration, the body language of the doctors was more towards neutral side with minimal facial expression of empathy and none touched the parents to sooth or console them. In two cases the HCPs (doctor and nurse) shouted at the family member around the declaration. In one case, the nurse left the post-death body preparation midway to give injection to another sick patient, which annoyed the family. The interactions between the doctors during resuscitation effort and death declaration were brief and sometimes in sign language.

After the death declaration, the doctors were available in the ward but none interacted with the parents or caretaker. In some instances, the doctor-nurses interactions in presence of the caretakers appeared as inappropriate and less empathetic. Arguments with security guard were observed when the family members tried to enter into the ward to see the deceased child and console the parents.

#### General communication and observations

The paediatric ward was crowded with the number of children admitted were more than two times of the beds available. Several of the children including newborns were on assisted manual breathing support due to lack of ICU (PICU and NICU) beds and ventilators. The resident doctors and nurses were observed shouting frequently while talking to the caretakers of the children. The behaviour and communication of the resident doctors changed in presence of the senior doctors. The attitude and communication by the senior doctors were appropriate and appreciated by the caretakers.

For the patients admitted to NICU, periodic briefing with the caretakers was practiced and usually senior doctors were present. These conversations were accepted by the families, but there were room for improvement.

*A mother of triplet (two babies already died and third was near death) asked the Doctor “should I call him (her husband)*? *Doctor replied “what will he see*? *There is little chance*. *Ok*, *call him*.*” Doctor gave a slip and asked the mother “call him*. *Show this slip*, *otherwise he would not be allowed to come in*.*” (Observation- NICU*)

## Discussion

This qualitative study provides valuable perspectives of the experiences of the parents and family who lost their children at the hospital and also of the HCPs who served them regarding the end-of-life communication. The study supplemented and triangulated the responses from different respondents and through observation of the end-of-life and general communication.

While the parents of deceased children perceived the attitude and communication by the senior doctors to be empathetic and appreciative, the behaviours and communication of resident doctors as blunt, inappropriate, insensitive and sometimes negative. Although majority of the parents had no serious dissatisfaction, some expressed unhappiness with the behaviour and languages used by the nurses. The death declaration made by the resident doctors was recalled as non-empathetic, blunt and cold. Majority of the parents had relatively neutral opinion about the interpersonal relationship with the HCPs, but few had unpleasant experiences during the hospitalisation period. The challenges in communication of the child’s status and prognostication were observed, which could have been augmented due to the language barrier. Most of the parents received no emotional support after death declaration. The parents perceived the doctors to be under pressure due to workload and need communication training. Death of the patient(s) affected the doctors, more the resident doctors and they were learning to cope with the situation. The end-of-life event observations also supported the expressions and challenges expressed by the parents and the HCPs. Overall the setting with overcrowded wards, overstretched HCPs and limited infrastructural facilities were putting stress on the HPCs and also patients.

Patient satisfaction evaluation is considered as a key aspect of QoC, but mostly used for the patient getting discharged, not for the deceased patients. The caretakers/family experiences related to end-of-life care and death are also important component of QoC, which must be explored to refine the services.

The end-of-life communication and supports (emotional, physical and instrumental) provided by the HCPs and the hospitals have an important impact on the experience and coping of the grieving parents. The views and suggestions from the parents and family on the experiences, care and assistance received during this critical phase would be useful to guide the doctors, nurses and hospital administration and the relevant policy makers to bridge the gaps for improving the services. The end-of-life care, breaking bad news and death declaration issues have been explored from various aspects for the chronic diseases, and cancers and more among adults. There are few studies related to these aspects related to the death in children, especially with the acute diseases.

Although the patients from diverse socioeconomic, language and educational backgrounds are treated at the study hospital, most speak and understand Hindi (locally spoken language). The resident doctors and nurses come from different parts of India with diverse sociocultural and language background. Thus, the pronunciation and use of Hindi words by them might be different than local dialect. The doctors are primarily responsible for delivering the bad news and death declaration. The communication skills, facing the family and handling emotions during difficult times are not part of the medical training and generally clinicians are poor at these. The studies from various parts of the globe, both developed and developing countries have reported the communication challenges faced by the clinicians [3, 4, 9, 19–23]. A report indicated that 35% of pediatric residents in India were comfortable in breaking bad news and 16% only received any training [24].

An enquiry for the end-of-life communication and breaking bad news for dying children in an Indian hospital identified preparing the families, using simple languages, doctor-family rapport and parent education status as critical issues. The doctors in the study considered themselves more attached to the paediatric patients than adults. They were aware about the families reacting differently to the child deaths according to the age and gender. The doctors perceived that unavailability of facilities (ICU bed and ventilators) led to deaths, which affected their emotional status. The doctors have been coping with the situation through clinical care and engagements in recreational activities. The doctors perceived the physical clinical care as their responsibility and the psychological care to be outside scope and feasibility. The resident doctors felt high patient load, long working hours, no training in communication and no formal psychological support as the challenges [19]. A study from Pakistan explored the causes of violence against HCPs. The HCPs from different categories perceived impatience, lack of respect towards HCPs, unrealistic expectations from HCPs and hospital as the client related factors and bad attitude of HCPs towards patients and caretakers, lack of timely communication, inability to counsel caretakers and prepare for undesirable outcomes as the provider-related factors for the violence against HCPs [6]. The Italian parents perceived that their role as parents and the physical intimacy were compromised during the PICU period. They were willing to be with their child and continue care throughout in the PICU including the dying processes, which were prevented by the HCPs. They also reported the need of space and opportunity for family intimacy during the critical times [9]. A study from Switzerland identified differential experiences among parents regarding end-of-life care according to the underlying diseases. While parents of children with cancer rated their experience as highest, the parents of children with neurological illnesses rates as lowest. The parents of the neonates reported shared decision making as key challenge. The key negative experiences reported were support for the family and communication [3]. The findings from our study are similar to the observations from other countries.

Studies have observed that the emotional and cognitive care, empathic attitude of the HCPs are more effective in management and improves patient’s treatment compliance [25, 26]. A systematic review found that according to majority of the parents the nurses provided better emotional support to them, but few studies noted parent’s disappointment about interactions with nurses. The parents reported the doctors to be cold, neural, less helpful and supportive than the nurses. The key factors for dissatisfaction included lack of communication and emotional support and insensitive comments. Some studies reported the parents appreciating the emotional, physical support and time spent by the HCPs during the distressing times [27]. A meta-synthesis found that during the end-of-life care and decision making, the parents found lack of communication and culturally sensitive information and sometimes depend on secondary sources like internet for resources. The parents wanted receiving honest, clear and complete information including the uncertainties in simplified language, but blended with hope (not false hope), compassion and sensitivity [28]. Our findings also reflect similar themes regarding the end-of-life care and death declaration by the doctors and support provided by the HCPs and institution to the parents and family.

The role of doctors and nurses in the end-of-life care including, their attitude and communication style are critical for the patients, parents and family [29, 30]. The doctor’s cool, detached and neutral posture might be considered as evasive, cold, and non-empathetic by the family and may be counterproductive when they need empathy. The doctors may feel powerless, ineffective and helpless at times of these crisis and seek blocking behaviours to immunize himself from the potential distress [31, 32]. Assessments have observed poor competency among the resident doctors in delivering bad news and limited understanding of the patient’s perspective [21]. In a study in South India, 71% of the doctors identified breaking bad news as the top priority core competency area in communication [33]. The doctors, especially the younger resident doctors reported to have death anxiety, stress related to care of dying patients and death declaration [34]. Lack of proper training for the doctors, nurses and other HCPs leads to emotional disengagement from the patients and their family [19, 35]. While good communication has positive therapeutic effect, bad communication may lead to detrimental outcomes. The medical and nursing curriculum didn’t provide adequate skill building in communication and crisis handling till now. Recently Medical Council of India has included comprehensive AETCOM (attitude, ethics and communication) module in pre-service curriculum of medical graduates [36]. Similar efforts are needed for the in-service and postgraduate courses for healthcare providers. Structured training programs through contextualised lectures, group discussions, role-playing, simulations and real-life coaching can build skills. Additionally, psychological support and institutional efforts for decreasing the HCPs anxiety and stress from uncertainties can improve their attitude and better prepare them to provide end-of-life care. A review observed that despite increased focus of the medical training on physician-patient interactions, the doctors were viewed as less supportive [27]. The family directed interventions have higher focus on bereavement support than enabling the compassion and support from the HCPs [27]. According to The Institute of Medicine, the HCPs engaged in childcare need skills in end-of-life, bereavement and palliative care, as part of the curriculum and continuing education using model curricula [37]. Several studies have stressed on importance of cultural and context appropriate patient-centred communication in improving patient satisfaction and psychological well-being [38, 39]. The medical schools and hospitals providing paediatric and obstetric care need standard socioculturally appropriate written and communication protocol using simplified language and training for all the HCPs engaged in childcare. Communication training models are being attempted targeting the trainee doctors [33], but it needs to be standardised and contextualised. The parents and family may experience variably the five stages of grief including denial, anger, bargaining, depression and acceptance, based on the type of illness, duration, type of care, and several other influencing factors [40]. The textbooks and communication training in medicine and nursing education need to include these along with appropriate counselling methods.

The inclusion of different categories of the HCPs and triangulation using the observation of end-of-life care were the strengths. The study had some limitations. The results reflect the perspectives and practices of the HCPs at one hospital, which may be context specific and hence may not be generalizable to other settings, where the patient load and hospital resources including may vary. The purposive selection of the participants (parents and HCPs) and the experience of the parents who consented might be different from those who refused to participate, which could have biased the findings. The observations could have been influenced by the patient and parents characteristics including types of diseases, socioeconomic status, education, religion, etc. No validation of the findings were attempted.

## Conclusion

This study found the experiences and challenges of the parents and HCPs regarding the end-of-life care and breaking bad news for the paediatric patients in north Indian context. The parents of deceased children expected empathetic communication and attitude from the HCPs, especially the doctors. The doctors, especially the residents with suboptimal communication competency, thin and overstretched staff strength and infrastructural limitations furthered the negative parent experiences. The end-of-life is a crucial situation and children, a special patient category, make the issue further complex for the family and the HCPs. While there is increasing attention to QoC and patient-centred care, there is need for improving the end-of-life care for paediatric patients through better integration of medical and parental priorities with provision of psychological and physical support. Future efforts need to design sociocultural, context and task specific modules for the different HCPs (doctors, nurses and support staffs) and document the impact of their implementation through various channels on the experiences of the family and HCPs about the end-of-life care.

## Supporting information

S1 Checklist Consolidated criteria for reporting qualitative studies (COREQ): 32-item checklist. (DOCX)

S1 File The in-depth interview guides and observation tools used for data collection. (PDF)

S2 File Transcription of the in-depth interviews with the participants. (PDF)

S3 File The narratives of death observations. (PDF)

S4 File The coding tree derived by inductive analysis of the in-depth interviews. (PDF)

## Abbreviations

HCP: Health care provider
ICU: Intensive care unit
IDI: In-depth interview
IQDAS: INCLEN Qualitative Data Analysis Software (IQDAS)
MITS: Minimally invasive tissue sampling
NICU: Neonatal intensive care unit
PICU: Paediatric intensive care unit
QoC: Quality of care
