# Supplementary material for: Perceptions of the parents of deceased children and of healthcare providers about end-of-life communication and breaking bad news at a tertiary care public hospital in India: A qualitative exploratory study
Source: PLoS One. 2021 Mar 18;16(3):e0248661. doi: 10.1371/journal.pone.0248661 (PMC7971872; doi:10.1371/journal.pone.0248661)
Supplement: S1 File — (PDF) [file pone.0248661.s002.pdf]

In-depth interview guide- Parent(s) of child who died

1. **Basic demography**
   1.1. Demographic information
      1.1.1. Age of mother
      1.1.2. Age of father
      1.1.3. Education status- mother
      1.1.4. Education status- father
      1.1.5. Type of residence- owned/rented
      1.1.6. Religion
      1.1.7. Origin- state (if belong to some other state)
   1.2. Family structure
      1.2.1. Type of family
      1.2.2. Total family members staying here
      1.2.3. Total number of children
      1.2.4. Male children
      1.2.5. Female children
   1.3. Occupation
      1.3.1. Mother
      1.3.2. Father
      1.3.3. Other family members

2. **Events that led to death of the child**
   (Please document as the event were told by the respondent)
   2.1. Could you tell me about the illness/events that led to her/his death? (include all the clinics, hospitals attended as ambulatory patient or hospitalised)

   Hospitalisation period

3. Now we would request you to tell us something about the period of hospitalisation for your child?
   (Probe: hospital, ward, duration of stay, course of illness)

4. Could you please tell us about the communication about status of your child during the hospital stay?
   (Probe: who communicated, frequency, clarity in communication, attitude)

5. Could you please describe you overall experience about the hospital care?
   (Probe: investigations, treatment given, involvement in decision making, satisfaction level, quality of care, cost of care)

6. Could you please describe your experience about communication by the various hospital staffs during hospital stay?
   (Probe: who all communicated, who was the primary communicator, nurses, doctors, other staffs, frequency of communication, mode of communication, details, completeness of information, skill of communicator, consistency in communication)

Events around death of the child

We would like to know about the events around death of your child.

7. Could you please describe the terminal evets that lead to death of your child?
(Probe: what happened, who detected deterioration, who was called to see, what was the response, the communication by the person, attitude and body language)

8. Could you please describe the death declaration process?
(Probe: who declared death, cause of death explained, meeting with any senior doctor/member, attitude/body language/expression/skill of the person, expression of empathy/sympathy, language and simplicity, perceived completeness of information)

Death and post death period

We would like to know about the events around death and after death of your child.

9. In your view what caused death of your child?
(Probe: understanding of the cause or disease, possible blame for anyone or any process)

10. Could you please describe about you and your family member's reaction to the death of your child?
(Probe: what was the reaction of mother, father, other family members who were present, did someone try to console you or your family member)

11. Could you please describe about you and your family member's experience about the procedure after death till handover of your child's body?
(Probe: processes asked to follow, documentation and papers given, interaction and behaviour of the staffs, respect for religious norms, time taken, cost/payment, assistance from the staffs)

Knowledge about the cause of death

12. What was the cause of death of your child and what is your perception about the value of knowing the cause?
(Probe: knowledge about the cause of death, willingness to know the detailed cause, potential factors that led to the illness/death, potential impact on the other family members or next pregnancy)

Decision making dynamics

13. During the hospital stay who discussed with the doctors/ nurses to make the decision(s) about treatment and other procedures.
(Probe: father, mother, any other family member, did you/your spouse talk to someone in your family like elders or relatives to take critical decisions)

Summarisation

14. Now, we are completing the interaction/ interview. Would you like to add anything?

Thank the respondent(s) for their critical contribution.

In-depth Interview Guide- Health care provider (Doctor- Pediatrician)

1. **Basic demography**
    1.1. Designation
    1.2. Total years of service
    1.3. Total years in current position

Service delivery and communication

2. Please describe your role related to the clinical care for patients and types of patients seen by you.

3. You come across several patients on daily basis. How do you inform and counsel the parents/ family members of children/newborn under your care?
(Probe: Approach immediately after hospitalization, during the course of illness and discharge)

4. You come across several sick patients, who are at high risk for death. How do you inform and counsel the parents/ family members of critically ill children/newborn under your care?
(Probe: How does the approach for critically ill children differ from the regular non-critically ill children, time devoted, frequency, person counseling/discussing with family)

5. In your opinion, what has been the hardest part about being a doctor treating sick children/newborn?
(Probe: informing/counseling the parents and family members, handling the deaths, etc.)

Death and related procedures

6. Several of children/newborn under your care die. Please describe the death declaration process usually followed at the hospital.
(Probe: How is the death declaration done, place of declaration, time taken, whom primarily targeted, usual reaction from families)

7. Please let us know about your personal experience of last death declaration in patient under your care?
(Probe: Mode of declaration, time taken, family members present, reaction from family members)

8. In your opinion, what are the challenges encountered by you regarding death declaration in patients under your care?
(Probe: explaining and convincing parents/family members, handling reactions, time needed, workload and other competing priorities, support from other colleagues/senior and staffs, security)

9. In your opinion, how your juniors (including trainees), co-workers, and other hospital staffs usually support around death of a child under your care?

(Probe: Who supports, what type of support given, any expectations)

10. How are the children brought dead to the emergency department handled in this hospital?
    (Probe: assessment, documentation, medico-legal aspects, autopsy)

11. How do you prepare yourself and parents/family members of the child who is dying/dead?
    (Probe: mental preparation, seeking support from seniors/colleagues, informing parents/family in advance to prepare them)

12. How does the death(s) of children under your care affect you?
    (Probe: mental stress, frustration, work performance)

13. How do you handle the reaction/response of parents/family members related to death?
    (Probe: explanation, seeking support from senior/colleagues, security)

14. What is your view about the causes of death, declared and exact/underlying cause/etiology for the children dying in the hospital or brought dead to the hospital?
    (Probe: approach to declaration, past records, mention of cause of death, effort for autopsy)

15. Have you received any training in communicating and handling death and critical illnesses? If not what all you would like to have in such training?
    (Probe: any training on communication- formal or informal, reading or self-learning)

16. Any other comment/ suggestions.

Thank the respondent for his/her critical contribution.

In-depth Interview Guide- Health care provider- Nurse (Pediatrics/Neonatology unit)

1. **Basic demography**
   1.1. Designation
   1.2. Total years of service
   1.3. Total years in current position

Service delivery and communication

2. Please describe your role related to the care for patients in this ward/unit.

3. You come across several patients on daily basis. How are the parents/ family members of children/newborn in this ward/unit informed/counseled about the illness and condition? (Probe: immediately after hospitalization, during the course of illness and discharge)

4. You come across several sick patients, who are at high risk for death. How are the parents/ family members of critically ill children/newborn in this ward/unit informed/counseled about the illness and condition?
   (Probe: How does the approach for critically ill children differ from the regular non-critically ill children, time devoted, frequency, person counseling/discussing with family)

5. In your opinion, what has been the hardest part about being a nurse caring sick children/newborn?
   (Probe: informing/counseling the parents and family members, handling the deaths, etc.)

Death and related procedures

6. Several of children/newborn whom you care die. Please describe the death declaration process usually followed in this ward/unit.
   (Probe: How is the death declaration done, place of declaration, time taken, whom primarily targeted, usual reaction from families)

7. Please let us know about your personal experience of last death declaration in patient you cared?
   (Probe: Mode of declaration, time taken, family members present, reaction from family members)

8. In your opinion, what are the challenges encountered by you regarding death declaration in patients you care?
   (Probe: explaining and convincing parents/family members, handling reactions, time needed, workload and other competing priorities, support from doctors, other colleagues/senior and staffs, security)

9. In your opinion, how the senior doctors/consultants, residents, other nurses, and other hospital staffs usually support around death of a child whom you care?
   (Probe: Who supports, what type of support given, any expectations)

10. How do you prepare yourself and parents/family members of the child who is dying/dead?
    (Probe: mental preparation, seeking support from seniors/colleagues, informing parents/family in advance to prepare them)

11. How does the death(s) of children under your care affect you?
    (Probe: mental stress, frustration, work performance)

12. How do you handle the reaction/response of parents/family members related to death?
    (Probe: explanation, seeking support from senior/colleagues, security)

13. What is your view about the causes of death, declared and exact/underlying cause/etiology for the children dying?
    (Probe: approach to declaration, past records, mention of cause of death, effort for autopsy)

14. Have you received any training in communicating and handling death and critical illnesses? If not what all you would like to have in such training?
    (Probe: any training on communication- formal or informal, reading or self-learning)

15. Any other comment/ suggestions.

Thank the respondent for his/her critical contribution.

In-depth Interview Guide-Health care provider- Support staff (Pediatrics/Neonatology unit)

1. **Basic demography**
   1.1. Designation
   1.2. Total years of service
   1.3. Total years in current position

Service delivery and communication

2. Please describe your role related to the care for patients in this ward/unit.

3. You come across several patients on daily basis. How are the parents/ family members of children/newborn in this ward/unit informed/counseled about the illness and condition? (Probe: immediately after hospitalization, during the course of illness and discharge)

4. You come across several sick patients, who are at high risk for death. How are the parents/ family members of critically ill children/newborn in this ward/unit informed/counseled about the illness and condition?
(Probe: How does the approach for critically ill children differ from the regular non-critically ill children, time devoted, frequency, person counseling/discussing with family)

5. In your opinion, what has been the hardest part about being a staff caring sick children/newborn?

Death and related procedures

6. Several of children/newborn in this ward/unit die. You might have observed death declaration for several children/newborn. Please describe the death declaration process usually followed in this ward/unit
(Probe: How is the death declaration done, place of declaration, time taken, whom primarily targeted, usual reaction from families)

7. Please let us know about your personal experience of last death declaration you observed?
(Probe: Mode of declaration, time taken, family members present, reaction from family members)

8. In your opinion, what are the challenges encountered by the doctors/nurses and other staffs while death declaration in patients?
(Probe: explaining and convincing parents/family members, handling reactions, time needed, workload and other competing priorities, support from doctors, other colleagues/senior and staffs, security)

9. In your opinion, how the senior doctors/consultants, residents, other nurses, and other hospital staffs usually support each other around death of a child/newborn?
(Probe: Who supports, what type of support given, any expectations)

10. What role do you play in the process of death declaration and the related processes?

11. How does the death(s) of children in the ward affect you?
(Probe: mental stress, frustration, work performance)

12. In your opinion, how are the parents/family members of the child who is dying/dead prepared for death declaration?

13. In your opinion, how are the reaction/response of parents/family members related to death handled?
(Probe: who supports them, any counsellor or counselling mechanism available)

14. Any other comment/ suggestions.

Observation: Events around death in Hospital

Ward/Unit:

Information of the deceased child:

Name: Age: Sex:

Date of Hospitalization: Date of Death:

Cause(s) of death:

Context of the child and illness:
(Please capture about the patient and course of illness in the hospital)

Family members present at the time of death:

Capture the course of events around death: Please try to capture the events, the communication between the health care providers (HCPs) and family members, actions/reactions by the family members in response to the communication by HCPs with reference to the time. The process and sequence of activities are to be captured till the body of the child leaves the ward.

Start Time	Service provider present	Family member(s) present	Event/ Activity	End Time

Start Time	Service provider present	Family member(s) present	Event/ Activity	End Time

Any other specific observations

Observing team members Signature
