# Supplementary material for: Perceptions of the parents of deceased children and of healthcare providers about end-of-life communication and breaking bad news at a tertiary care public hospital in India: A qualitative exploratory study
Source: PLoS One. 2021 Mar 18;16(3):e0248661. doi: 10.1371/journal.pone.0248661 (PMC7971872; doi:10.1371/journal.pone.0248661)
Supplement: S2 File — (PDF) [file pone.0248661.s003.pdf]

List of In-depth Interviews- Parents of deceased children

S. No.	Participants	Age	Religion
1	Parents 1	Mother-20-25 years<br>Father-25-30 years	Hindu
2	Parents 2	Mother-20-25 years<br>Father-25-30 years	Christian
3	Parents 3	Mother-20-25 years<br>Father-20-25 years	Muslim
4	Parents 4	Mother-25-30 years<br>Father-25-30 years	Hindu
5	Parents 5	Mother-25-30 years<br>Father-25-30 years	Muslim
6	Parents 6	Mother-20-25 years<br>Father-25-30 years	Muslim
7	Parents 7	Mother-25-30 years<br>Father-30-35 years	Hindu
8	Parents 8	Mother-30-35 years	Hindu
9	Parents 9	Mother-30-35 years	Muslim
10	Parents 10	Mother-25-30 years<br>Father-35-40 years	Hindu
11	Parents 11	Mother-20-25 years<br>Father-25-30 years	Muslim
12	Parents 12	Mother-25-30 years<br>Father-30-35 years	Hindu
13	Parents 13	Mother-35-40 years<br>Father-40-45 years	Hindu

In-depth Interview- Parents of Deceased Child 1

1. Demography

**1.1**	**Primary respondent (relation with the child)**	Mother, Father
**1.2**	**Age of the primary respondent**	Mother-20-25 years<br>Father-25-30 years
**1.3**	**Other members present**	Nani, Maternal uncle (mama of child)

2.1 Where do you usually go or whom do you consult during the illness of your child?
Mother- There are many doctors here, people go to the doctor which they like. There are many more doctors here.
2.2 Where do you usually go or whom do you consult for vaccination of your children?
Mother- Everybody goes to the Anganwadi center for vaccination.
2.4 Please specify the reasons for the preference for the facility and/or healthcare provider.
Mother- There is lot of traffic here, we cannot take him at distance so, if the child is less sick then we take him to nearby doctor.
3.1 Can you please tell us know about the illness and events that led to death of <name> your child?
Mother- Our child got ill, as when he got vaccinated at Aangawadi center after that he got swelling, then we used to do ice fomentation (NANI- swelling was there), then we took him for check-up at Aangawadi centre, it was not getting treated even after 20 days. Then doctor there said to seek advice of other. Then we took him to doctor and then doctor gave the tube to be applied and advised to do ice fomentation. Then we used to apply the tube, then it was getting less and still little was left. Child had a fever that was shown to the private doctor, then doctor said to give medicine, he gave fever medicine bottle, and then also the fever was coming. After doing this, he left drinking milk one day, then he used to drink it with spoon, I used to give him my milk only. He used to weep, one day he started crying a lot and not drinking milk then we showed him to doctor in Sarita Vihar where everyone was saying to take him. Then, doctor gave medicines to eat, and he checked everything, they checked ears, nose and then said all is right, then I said that he does not drink milk and he had fever. I gave him milk with spoon one by one, then he fell asleep. Then after sleeping in afternoon I gave him milk, then he again slept and woke up in the evening then I fed him milk, I said to my mother that now he is drinking milk, then she said to feed him with the spoon. Then when I was feeding him, he drank some milk, during drinking milk he started shouting and crying loudly. Then I took him in the lap and tried to counsel him, then his hand and legs started getting tight.
After looking at him, all of us started shouting and then we took him to the doctor immediately, the old doctor, he checks everything and then we quickly took them him to that doctor. I was not able to understand anything at that time, we were the mother daughter alone at that time. The doctor said and told that it is a problem of breathing. He said, take him to K Hospital early, so we said okay. Then we came home and we could not find any car, then the brother who stay nearby took us. It was Monday, and there was a lot of traffic, and we got struck for 2-2.30 hours. Our child was shouting from home and also on the way, he did not stopped crying, as soon as we reached hospital his voice stopped but he was breathing. Then I took him inside and called doctor, then all doctor put him on machine, then doctor gave me the machine to press it. Then for 3-4 hours he was breathing. Then around 12-12.30 he was not alive. We took him at 7 o clock from our home and reached at 9 pm, due to traffic. We went to hospital with our neighbor.
3.2 Who all supported you during the illness and hospitalization of your child?
Mother- When I went to the hospital, my husband was with me, my father, my mother and neighbor went along with to the hospital.
4.1 Now we would request you to tell us something about the period of hospitalization for your child? (Probe: hospital, ward, duration of stay, course of illness)
Mother- The hospital took a sign and asked what happened to the child, he asked if the child ever turned blue, then we said no, did the child get a high fever, or such a problem had happened earlier So, I told that

it had not happened before, this all happened after vaccination, he got fever and after that all this happened. He had played for two months, after vaccination in the second month, he had all this condition.
<duration of stay>
From night 8 till 1 o clock we were in hospital. At 12.30am he died, after that they told us it will take half an hour to do paper formality, then they will leave us. One report was still not available, they said it will take 5 hours to give the report to us, but we said we cannot wait for 5 hours.
We didn't know that he has died, my husband told me that doctor has said that you should take your child to other hospital. Then we left hospital at 12.30am. We got one report, and it was mentioned in that there is blood infection. And we didn't take 2nd report as it said to be given after 5 hours.
Nani – He was dead so we came, these people did not tell us that it has died.

4.3Could you please describe your experience about communication by various hospital staffs during hospital stay? (Who all communicated, who was the primary communicator, nurses, doctors, other staffs, frequency of communication)

Mother- behavior of doctor was good. When doctor provided all things then, they were all looking at other children also, they were not looking at one child only. Cleanliness was fine, it was clean. No money was spent.
<nurse>
Mother- we need to tell nurse that our child is not moving. Nurse just gave the bottle of water injected, and said your child will be cured.
<primary communicator child health Well, no one would tell, doctor called my husband and explained everything, that there is such condition, there is no hope of survival, and after this they took signature. Then half an hour later, he died.
<did you understood what doctor explained> |
Father- When I did not understand what to do, I understand that time which I understood, took the signature and then went away.

5.1 Could you please tell us about the death and death declaration of your child? (Probe: who declared death, the body language and expression, preceding events before death)

<who declared death>
Mother- she was looking as head doctor, as nurse and other person were telling about the things they gave to the child.
<body language & expression> Mother- I had heard that time too, from them that let the mother of child go out then we will discharge, this all is said by doctor.

5.2 What was your and your family member's reaction to the death of your child?

We didn't know when death happened, my husband knew about that he was unable to speak he was not able to come in front of us. Even my father knew about that, he was also not able to speak. They didn't say anything because we would have started crying in hospital. Even doctor told not to tell mother here, the doctor said, please take mother out, then we will give you discharge. Then I came outside, then my husband came after discharge formality.

5.3 Do you blame someone/something for the death of your child? (Probe: who or what is it, what did you do in this context)

Mother- whom should we blame
Father- we can blame, the person who vaccinated the child, who gave the medicine (mother- we went to khadar village for medicine), today he got ill we got him medicine then he drank milk, then slept, then in evening the problem started, then when he woke up and then he started shouting.
Mother- This all has happened in one day. We gave medicine and after that he woke up in evening, and all this started happening.
Father- There was no problem before.
Mother- Whom we should blame either this doctor or that doctor? We have not got the report, so how can we know what was the reason and how did this happen.

5.3 How was your experience about the procedure after death till handover of the body to you? (Probe: processes, documentation and papers given, respect for religious norms, time taken, cost/payment, assistance from the staffs)
Mother- It took half an hour.
Father- We did not get any slip, we only got one report, we got one slip to take the child out of hospital.
Nani- At 12.30 -1 we took the auto, and we came home at 2 am in night.
Father- When in hospital how can stay long, I made the excuse to my wife and mother-in-law, came with stone on my heart, these people will be separated then who will handle them, we were already in tension. So, I didn't tell anyone that child is dead, as if I would told them they would have started crying and shouting there.
5.4 Who all from your family and community/neighborhood supported you during this period? (Probe: who informed them, who all came, what type of support given)
Mother- our whole family was there like, my mother, father and my husband.
5.5 Is/there any change in the interpersonal relationship between you and your spouse and other family members after the event
Nothing has changed like this my husband tells me that what is there in life, it can happen to anyone in life. It is not necessary that we keep sitting on the same thing and do not do something. He says that you should be happy, the thing which happened has happened, what to do now, new beginning can happen again everyone tells that. I will tell my sadness to my husband, he is supporting me.
6.1 What all rituals and procedures did you follow after death of your child? (Probe: body preparation, who prepared, burial/ cremation, other rituals and timings; who performed them according to the rituals)
Mother- All night child was at home, then around 6-7 in morning we take him for cremation. No bathing was performed, because it is said this all is not performed for the child. No worship is performed, only essence sticks are Enlighted, then we bury him, then nothing was done. We didn't cook for 4-5 days.
6.2 How did you, your spouse (wife/husband) and immediate family members cope with the difficult phase?
Mother- Both of us handle each other, we understand. My husband had started working on 15-20 days later. As it is not yet engaged in the company, he is still learning. So, I said him to resume his work as how we will run the house.
6.3 How did the family members support you and your wife during the difficult phase? (Probe: burial/cremation rituals, community norms/rituals, coping with the phase, financial)
Mother- My father was present with my other relatives and all other family member was there. My mother-in-law also came.
Father- My family stays in the village, they came later, 7-8 members came from the village.
6.4 What were the reactions/ counselling and suggestions given by the relatives/neighbours/community after the episode?
Mother- The neighbors were saying that they should took the child to a big hospital or takes something else. Someone said that they should have taken the child to Apollo, someone said to take Kalawati. So, I said which hospital is bigger than this hospital?
Father- In hospital all check-up is done. My wife delivery and check-up everything happened there only. So, which hospital is bigger than that?
<knowing the cause> Mother- Neighbor says that child was suffering from pneumonia, but I said if this was the reason then this would have appeared in the report. And we would take-care of everything, we avoid putting him on floor, avoid taking out he was 2 months old, we would keep him inside as there was air outside. He was weak when he was born his weight was 2 kg, and after one and half months his weight was 3.30 kg. We thought when he will be of 3 months then we will take him outside.
6.5 How your and your family life changed after the death of the child?
No such change has happened, we live as we were living before, they only explain to me, yet remember the child and cries and explains to me. We give support to each other and tell each other we should live like this, what else we can do, if anything happens we will tell each other only. My husband is such that

he remain happy in sadness and in happiness. He doesn't share his sadness with anyone. He says if he will share his sadness everyone at home will remain unhappy, so if he hides his sadness everyone will remain happy. But man will always be in tension, he cannot forget everything so soon.

7.1 What was the cause of death of your child and what is your perception about the value of knowing the cause? (Probe: knowledge about the cause of death, willingness to know the detailed cause, potential factors that led to the illness/death, potential impact on the other family members or next pregnancy)

Mother- My delivery was normal. Only after vaccination, we were in trouble, because after that he developed fever, we took him to the doctor and doctor gave the medicine of fever.

<willingness to know>

Mother- the cause of the death must be known, that how it happened. We didn't even go to collect the report because there is delay in giving report. Whole day is spent, and there is a lot of traffic and there report is not given on time.

<reason told by doctor>

Mother- The doctor told the infection in the blood and explained the problem of breathing.

7.2 In your view, what more could have been done to know the exact cause of death and the reasons causing the disease? (Probe: investigation, autopsy, any other)

Mother- If we find that report then we would know something from the report.

7.3 Are you aware of any method for identifying the exact cause of death? (Probe: autopsy, investigations, etc.)

Mother- We have heard, post mortem is performed for some accident or some incident, we have not heard that it is performed for some disease.

Father- for the disease, even I have not heard.

7.4 What are your views about the autopsy/ post-mortem for identifying the cause of death? (Probe: necessity, religious aspect, time needed, disfigurement, etc.)

Mother- we don't want tearing thing. We do not know, there is something different, in the last rites of post mortem body. We do not know anything like this. We have never seen anything like that.

7.5 What are your views about collection of the tissue (like done for biopsy to diagnose diseases) and body fluid samples for identifying the cause of death? (Probe: necessity, religious aspect, time needed, disfigurement, etc.)

Mother- if they do not tell us it is fine, we cannot see in front of our eyes. And that time if someone has told we and we would know the reason then we would have agreed.

Maternal uncle- There was nobody to tell that time about MITS. If the doctor told us, then we would have agreed, but we do not have any information about it.

7.6 How parents and family members can be approached for the tissue and fluid sampling? (Probe: who should approach, whom, when and how should approach; what should be told and in what detail; should some picture of the method be used to explain)

Mother- That time parents are not in such condition. My father or some relatives they would explain us. In hospital if head doctor tells us about this it is ok.

7.7 In your view, what factors are important for acceptance of the tissue sampling by the parents and family members? (Probe: desire to know the cause, trust, confidence on the health care provider)

Mother- We also used to think that the person who has gone is gone and the time has passed, now what we will do after taking the report, but in our heart, it is the wish that if we had brought that report, then we would have known what was cause of death and we would have become aware of the future.

Maternal uncle- if some person falls ill, this happens step-wise, we know person was not well, but if everything happens in one day, then we don't understand anything.

Mother- Therefore, we wanted to get the report, as there is still we are in our mind that we should have get the report, and then we will become aware in the future.

<trust about tissue sampling>

Maternal uncle- In everyone's mind postmortem is about tearing out the body and body parts are taken out. More than half the people are scared. All these things have ended a man's life and now tearing will take place.

8.1 In your view, what can be done to improve identification of cause of death and efforts to reduce the deaths in children?

Mother- Only after seeing the report will we know that how it happened.

Maternal uncle- Could this be the reason that we have shifted our sister house when the child was born?

Mother- As we were living in rented house and we shifted their 1-2 days before, so could that be the reason of our child getting ill. Then, the people used to say that there is something in that house. But, we don't in all these things, but people will speak what they think.

Maternal uncle- But, this could not be due to the house.

7.1 In routine day to day operation who takes decision about the major activities about the family?

my father takes decision about major activites as my in laws stays in village. Everyone has a decision for treatment and everyone speaks, who should take it to the doctor and whom to show. Everyone speaks including my husband, father, brother and mother.

7.2 During the hospital stay who discussed with doctors/nurses to make the decision about treatment and other procedures

At that time nobody was at home, only me and my mother was there, my neighbor who is like brother accompanied me to hospital. i took the decision during that time for reports, admission and for making slip. After sometime my father, husband came to hospital. My father was doing al work in hospital, my husband was sitting when doctor came for inquiry.

7.3 After death of your child, who decided about the cremation and other post-death activities and rituals

My father was taking all decisions during cremation, he didnt discuss anything with anyone because he was a small child. my mother, neighbor and some relatives was also present, took the child and buried.

In-depth Interview- Parents of deceased children-2

**1.1**	**Primary respondent (relation with the child)**	Mother, Father
**1.2**	**Age of the primary respondent**	Mother-20-25 years<br>Father-25-30 years
**1.6**	**Other members present**	None

2.1 Where do you usually go or whom do you consult during the illness of your child?
People around here refer to the Astha clinic for their child. It is a private clinic and the is the CH Hospital, where they go, and if their condition becomes more serious than, they go to S hospital.
2.2 Where do you usually go or whom do you consult for vaccination of your children?
For the vaccination, we go to the nearby dispensary, or Asha sister or Aganwadi worker come to us for same.
2.4 Please specify the reasons for the preference for the facility and/or healthcare provider.
There is no need to stand in the line, gets their early. Go directly and meet to the doctor. It is private and there is less time consuming. My wife does not know Hindi properly, then I do not have too much time, I have duty also, I work in the restaurant.
3.1 Can you please tell us know about the illness and events that led to death of <name> your child?
<Pneumonia or hole in the heart >
My child ... she had become pneumonia, so we went to the hospital, she was born at home. Then when we went to the hospital, we know that she had a hole in her heart, he did not check it, just said seeing that it could happen. We know all this in Nepal only. Then the doctor said that I have to do echo test, then I said that I cannot live in village so many days because I have the duty, that's why I brought them here. She had pneumonia first then there was a big hospital three or four hours ahead of my village.
I was not here; my wife took my baby. She was there for 8-10 days in hospital. They suspect a hole in baby's heart. Then I told to my wife that let we go back to home otherwise we have to pay hospital charge for next 10 days even baby's condition was not improving. Then we came to home and left untreated till six months. I called my mother and child to Delhi, when I came back and consult at R Hospital. A doctor at Malviya Nagar advised us to go to R hospital because S hospital and A hospital were usually overcrowded. Otherwise I prefer E hospital and we have ESI card also. If we went ESI, it could be free of cost and time saving.
Here, no more expenses incurred but they were demanding 30-35k for heart operation. Then I thought, we have ESI card, we should go there. Still, arranging 30-35k was not a big deal, at cost of baby health. We had arranged blood from 4-5 persons for the surgery and money. Each time when we went for surgery, baby was suffering from cold and fever and doctor postponed the surgery. It was about 5-6 times when doctor cancel the operation and refuse to admit.
3.2 Who all supported you during the illness and hospitalization of your child?
In the whole procedure, my wife and mine are there and sister-in-law came for a short time as she could not be with us for a long time.
4.1 Now we would request you to tell us something about the period of hospitalization for your child? (Probe: hospital, ward, duration of stay, course of illness)
<How did you reach Safdarjung>
At that day, He had fever it was about 4-5 days. Once it got cure by consultation to CH Hospital. After that my wife took him to AS Hospital in my absence. I was then at duty. Nothing improvement had been seen at there. Once a time, when I came from duty around 12-12:30am, then baby's face started swell, his face was not looking normal and had irregular breathing. His face became tilted. Then we were scared and called Ambulance for referral. First I thought to go RML but later on when the situation became worse, we decided to go S Hospital as it was in nearby.
We went to emergency ward. Doctor told ''he had an episode of seizure'' and look our previous report. They were unable to start treatment as earliest. We had a long list of reports, they were copying all. Then they took injection to my baby as she were not sleeping and keep crying all time. Then, they started treatment and shift the baby to ward no 18 at morning. After shifting in the ward,

there was random treatment had happened. We reached at 2 am (night) and she was dead at next day 11:30 am.

4.2 Could you please describe you overall experience about the hospital care? (Probe: investigations, treatment given, satisfaction level, quality of care, cost of care)

<Satisfaction> They know more about this. Either they were giving oxygen or water or vapor. After some time, we had urged to shift baby in emergency but they did not and send me to take medicines. After 9-10 o'clock, Security guards were not allowing us to go in ward, they allow only in case of providing medicines.

My wife was there. Doctors came to ward only after call. Once, Doctor took water from her bone for investigation and told me to take medicine. I went to purchase medicine. It was long que there so became late to return.

A bone test was conducted another was blood test actually blood test was repeated. One report came earlier and baby was dead before second report came.

4.3 Could you please describe your experience about communication by various hospital staffs during hospital stay? (Who all communicated, who was the primary communicator, nurses, doctors, other staffs, frequency of communication)

<Primary communicator> I missed the name of all but in hospital, there was a senor doctor, a young age doctor, a learner and another one. A person was there who always been scolded by seniors.

<Nurse Behavior > <supporting staff> Nurse's behavior was average. There were many people like us in hospital and she had to cater all. But, limited facilities were there, Doctors came there (at ward) later on. Its common among all govt. institution, staffs are not talking politely and finished all task instantly without mind the quality.

5.1 Could you please tell us about the death and death declaration of your child? (Probe: who declared death, the body language and expression, preceding events before death?)

<Who told you> <about declaration of death>

Baby dead at 12:45 AM, he was dead before I reached with medicine. They did not talk about, even after death they (Doctor) continued to inspect baby. I told them that there was no use to check him, baby was dead. They were silent even after. Again I told him to make a discharge card. They did not tell me about death, probably told to my wife. Death was occurred when I took medicine outside from ward. My wife called (Phone) me then but I missed phone as congested network. I call back but could not connect with. When I came back child was dead.

<Wife didn't know Hindi; father was asking in his language about the question asked to her>

<Husband asked to wife about the behavior of doctor during death declaration>

It was average they just told she is no more.

5.2 Do you blame someone/something for the death of your child? (Probe: who or what is it, what did you do in this context)

We can't blame anyone even we are responsible for that. We had tried what we can rest of all also put their effort. Whatever happened was done. We can't blame after that. We were continuing their treatment since their birth. She could have cured at earliest if possible. We went to private doctor also they did not treat after that we went to govt. hospital. We can't say, they were irresponsible because doctor came every time when we called. That's it.

5.3 How was your experience about the procedure after death till handover of the body to you? (Probe: processes, documentation and papers given, respect for religious norms, time taken, cost/payment, assistance from the staffs)

After death, we told to discharge. They took a bit time in packing after death and did not come even after 2-3 call. It took 30-45 min after death to release. After handover the body, we had a shawl to wrap and went directly to crematorium from Ambulance. Did not come to home.

6.1 What all rituals and procedures did you follow after death of your child? (Probe: body preparation, who prepared burial / cremation, other rituals and timings; who performed them according to the rituals)

We are Christen so not followed that (funeral). After death, we did pray and gathering

6.2 How did you, your spouse (wife/husband) and immediate family members cope with the difficult phase?

Some sympathies after came here, some over a phone call. But nothing was possible after death. It's good to leave all things to god, would be better at that time.

6.3 How did the family members support you and your wife during the difficult phase? (Probe: burial/cremation rituals, community norms/rituals, coping with the phase, financial)
Many people gather after that, brother, etc. we took from there at 12:30. Jija ji, Didi was with us. Actually she tied Rakhi. No expense had incurred, as we went from Ambulance.
<Emotional support post death> (religious leader)
My sister and brother were with us and people and the priest were not regular at Church. After getting a call my sisters would visit me. One of my sisters who would live in Saket also came. Father was busy at that day, so his wife was there.
<Body preparation>
Baby did not bathe, they taken directly from the hospital.
6.4 What were the reactions/ counselling and suggestions given by the relatives/neighbours/community after the episode?
<Knowing the cause of death>
Parents told the lady that you were not destined to have the child that is why she left this abode. I had not spent any single penny on the heath care of the child. The only money being spent was on the transportation to various government institutions. Everyone seeing us, first we were at ground floor later on we were not feel well there so shifted to upper floor.
No one told me about this but what we can do after knowing the reason.
7.1 What was the cause of death of your child and what is your perception about the value of knowing the cause? (Probe: knowledge about the cause of death, willingness to know the detailed cause, potential factors that led to the illness/death, potential impact on the other family members or next pregnancy)
As per my understanding, knowing reason is important. It can improvise our next case. She is pregnant and expecting baby by 25th. This learning may be useful there. We can work more confidently in next time. Even it's important for others also. It's for knowledge.
7.2 In your view, what more could have been done to know the exact cause of death and the reasons causing the disease? (Probe: investigation, autopsy, any other)
<Post mortem>
Doctor can do these things to know the death reason and we can do as per our home. Postmortem was needed but we did not.
7.4 What are your views about the autopsy/ post-mortem for identifying the cause of death? (Probe: necessity, religious aspect, time needed, disfigurement, etc.)
<Religious aspect >
We can know all the reason behind the death. I mean if any other reason was there beyond fever. May be other reason was there like wrong meal consumption, wrong medicine or over dose.
All rituals are same. In our case we put it into box. It's nothing affected to postmortem. MITS is not interfering our rituals.
7.5 What are your views about collection of the tissue (like done for biopsy to diagnose diseases) and body fluid samples for identifying the cause of death? (Probe: necessity, religious aspect, time needed, disfigurement, etc.)
<Time needed>
Yes! It could be possible, no harm in this. We could stay 30 min more for this. Even if they told about this we can stay a bit more.
7.6 How parents and family members can be approached for the tissue and fluid sampling? (Probe: who should approach, whom, when and how should approach; what should be told and in what detail; should some picture of the method be used to explain)
We could be convinced from doctor or our brother. After knowing the reason from post mortem, one may feel regret about our effort in order to overcome.
7.7 In your view, what factors are important for acceptance of the tissue sampling by the parents and family members? (Probe: desire to know the cause, trust, confidence on the health care provider)
We can say him like what happened to be done (sympathize), now try MITS to know the exact reason. You must get the answer by this technique. It should be done at the time when they pack (Wrap) and release because after packing, it may be difficulty in MITS. It should be better if they tell after death. It should be in knowledge of their parent. It obviously not happened before death.

8.1 In your view, what can be done to improve children's efforts to reduce deaths and to identify the causes of death?

Doctor can do these thinks to know the death reason, we can’t do anything. He went there with faith. We are not expecting this at there. If you give time, treat good etc. then its ok.

In-depth Interview- Parents of Deceased Child 3

**1.1**	**Primary respondent (relation with the child)**	Mother and Father
**1.2**	**Age of the primary respondent**	Mother-20-25 years<br>Father-20-25 years
**1.3**	**Other members present**	Maternal grand mother

Maternal grandmother- my child was fine, he suffered from cough and fever, then we went to doctor, when we reached senior doctor came and took investigation of child, he said your child is normal. he said the pipe can be removed in 1-2 hour, your child will be fine, then second doctor came, didnt know what she does the nerve burst, doctor said nerve burst has happened. My husband (child maternal grandfather) got expired 6 months ago. i dont have any support, i have 3 daughters and 1 son he takes drugs, why should i lie, i am living in rented house. my husband also died in S hospital.
Mother- I was in bad state, after my son was born i felt like i was born again. my child was born in G hospital, they have all my records of my name, my address they can tell you how i have delivered my child. earlier doctor refused and said you are serious and you may not survive. i had normal delivery with small operation.

2.1 Can you please let us know about the illness and events that led to death of your child?

Child was having cough two days back, we gave him medicine of cough, he got bit relieved, on same day in afternoon his health detoriated and he was also having fever, then we took him to local doctor, he gave medicine and said dont worry your child will become fine.

In the morning his health was getting worse, we was better at night after we gave him medicine, he was even playing, he slept near me on floor, and he was fine. At 6 in morning he was not drinking milk and then at 8 we took him to local doctor. He nebulized the child, he was better after getting nebulized, and then doctor told to give medicine at 9-10. To avoid any reaction I gave medicine at 10:30, as he had one dose in morning. in afternoon his face started becoming white and he was crying, we took him to hospital. We took him to S hospital in emergency ward, he was crying at that time. tell me onething even you can note that point, that if child is not breathing then how he can cry. i doubt on this point, when child can not breathe, i also have breathing problem, when we can not breathe then we cannot even speak. that child was crying so much, he was even crying in my lap. In hospital they said to admit the child, we disagree initially, we said give medicine, we can go home. our heart was not allowing to admit the child here. when i saw my child crying, then i said to nebulize my child, after nebulization he was fine, after that even he slept.

when child was on nebulization one doctor came and started giving oxygen using a pipe other doctor even refused to do this as child was normal and said there is no use for this pipe, the child was crying, doctor inserted the emergency pipe (bag and tube) and removed the nebulizer, and in this process we lost our child. our child died and doctor didnt gave us report from the hospital and we didnt even signed till now.

Hospitalization period

3 Now we would request you to tell us something about the period of hospitalization for your child?

Mother- we stayed for one night at hospital, the child died at 10 in the morning. we went at 4 in evening and next day at 10 in the morning child died, and then we was handed to us. in morning at 8 when doctor saw the child he said your child is normal, he said till 2 we can remove emergency pipe (bag and tube) if child is breathing normally. then i dont know what other doctor at 10 did, he was giving glucose using bottle infront of us, we thought he was giving medicine. tell me onething my child was not given fever medicine then only his fever reached to brain. they said fever is there, thats why brain nerve bursted. hospital think we dont know anything, tell me if they had given medicine on time then fever would not reach in brain. they didnt give medicine on time then only fever reached brain, we took the child to hospital for treatment. he was not having fever at home. fever appeared on the way.

Maternal grndmother- child was breathing fast.

Mother- earlier we didnt know why he was breathing fast. in hospital neighboring bed ladies told about this. we didnt recognise earlier about fast breathing, when we came to know we rushed to hospital.

4 Could you please describe your overall experience about the hospital care?

Mother- The doctor did not perform any test in the hospital, just inserted two needles and glucose. No blood was taken for the test.

Maternal grandmother- We are telling, one doctor told other doctor not to insert the emergency pipe , yet the second doctor put the pipe in child. No money was not spent in hospital. We are not educated that's why we were driven away and was told one person stay inside and other person go outside.

Mother- We do not know which doctor came at 8 o'clock, he was a big doctor or a small doctor, the big one was of black color, when our baby died and the one who had checked and saw the report, this doctor shouted at other doctor that when child and his report was normal how he can die. The big doctor came on round at 10, he was shouting at other doctors and were having some meeting.

5 Could you please describe your experience about communication by the various hospital staffs during hospital stay?

Mother- the doctor inserted the canula, no she was not nurse. When we said why are you putting this, then she scold us and said you take home then, why our child was nebulized, my baby cried, he was getting relived by nebulization, he was sleeping, they took oxygen from baby, when you want to go home, then what is need of oxygen, then we admitted baby and said no problem you can do your procedure. They forcefully inserted pipe in baby mouth when baby was sleeping and playing. I think my baby died due to forceful insertion of pipe.

<Doctor told the reason for inserting pipe>they said baby has problem in breathing. We know that our baby did not have breathing problems, our baby was playing well, was looking with eyes open, was moving his feet and was also crying, he cried on during nebulization. Now you tell me that when the child is crying, how he can have breathing problem.

Death and post death period

6 Could you please tell us about the death and death declaration of your child?

Mother- they told us nothing. When the baby died, he started moving his hands to remove his pipe, he was hungry, we asked doctor baby is hungry remove his pipe so that I can feed him, then doctor said you cannot feed the child, we are giving glucose, they give 2-2 bottles of water, tell me in fever no water is to be given.

Maternal grandmother- we didnt know what to do when baby was handed over in lap, we should cry or what to do, we buried him, who would support a poor person.

<Who told you about death>when I saw my baby and checked him, I saw his hands and feet are cold and his stomach is warm, and when I touched his chest he was not breathing. Then I rushed to doctor and called the doctor and said my child is not breathing. Then doctor came and said your baby is no more. I don't know anything, they said your baby is not breathing. The doctor who came told about the death of my baby.

7 What was your and your family member reaction to the death of your child?

Mother- I was not aware what was going on. His father showed some courage but I was not aware what was happening. Even his grandfather was not aware what was happening. My mother was not present at that time.

8 How was your experience about the procedure after death till handover of the body to you?

Mother- he was handed over to us within 10 minutes, and returned immediately. Like he died at 10 o'clock and at 10.30 we had left the hospital. His father was made to sign on one paper and said we will give paper to you after one month. Child was wrapped in blue cloth when he was handed to us.

Maternal grandmother- baby was handed to us immediately, when my husband died in evening he was handed to us next morning. Child was handed to us within 10 minutes.

9 Who all from your family and community/neighbourhood were present at the hospital and how they supported you during this period?

Mother- my husband, my father-in-law was with me at hospital.

Maternal grandmother- my sister-in-law visited hospital when child was normal. Then I called and ask how the child is, my sister-in-law said that there is no need to worry now, the child is safe, the child is now normal there is no fear. She said me to visit hospital in morning at 4, I was waiting for my sister-in-law when child died.

Maternal grandmother- Baby was well, my daughter and sister-in-law took baby to hospital. Child father and grandfather came later. My sister-in-law was saying that there is no need to panic, child is being nebulized in evening. My sister-in-law returned at 11 in the night from hospital.

10 What all rituals and procedures did you follow after death of your child?

Mother crying

We brought the child home from the hospital. Child was bathed at home after that he was taken for burial. Mulana bathed the child, like there is pandit in Hindus, he is big person in our community, he performed all the rituals from bathing to burial.

<Who accompanied till burial place> A lot of people had gone, all the neighbors, men had gone, the ladies had not gone, the ladies did not go in us. I watched the baby at home only. Nothing was done afterwards as he was small child, one day morning was observed.

11 How did you, your spouse (wife/husband) and immediate family members cope with the difficult phase?

Mother- One day in dreams I saw that the child is playing in my lap, my mother brought baby to me and I was playing with child very well. This dream came after 3-4 days of child death. The child was healthy as he used to drink my milk, no one could say he was three months old.

Maternal grandmother- i am vegetable vendor, one day I saw in my dreams that baby is playing with me in my lap, I am cuddling with him. The child is happy and playing with me.

12 Is/was there any change in the interpersonal relationships between you and your spouse and other family members after the event?

Mother- my husband and me supported eachother.

Mother crying

Maternal grandmother-After child death her in-laws fight with my daughter, her husband and her father-in-law were ready to leave my daughter, her husband was ready to give her divorce, this all happened after child death, they blamed my daughter that why you took child to S hospital.

Mother- they were saying why you took child to S Hospital, they said you shuld have called us, i called him (child grandfather) he said to take child to private hospital, this would have not happen. my husband was at his home, i took the child to hospital with my relative. my child became healthy and even he was playing after nebulization.

<What is the situation now> mother-now the things the alright. They were not saying anything their neighbors were putting some things in their mind, then person mind will divert to those things. At that time he was misguided by his neighbors, he was right before. Now all things are right.

13 How did the family members support you and your wife during the difficult phase?

Right now my mother and family members understand me and support me.

14 What were the reactions/ counselling and suggestions given by the relatives/neighbours/community after the episode?

Mother- I read namaz (holy book).

Maternal grandmother- Whose child is gone, the attention remains in it. Like I am maternal grandmother, when we still see the picture of the child, we start crying, the child's attention is not getting off anyone.

15 Do you think and/or discuss with your spouse/ family member(s) about the death and related events and blame someone/something or feel guilty for the same?

i blame the doctor who checked my baby and inserted the pipe, his fault is there. and 2nd i blame who gave glucose to child, i dont know whether she was learning or something as in this hospital some come to learn things, i think she was nurse. one doctor came and checked the baby, and 2nd one came and said the baby is no more.

16 When did father and mother (if working) returned to work after the death of the child?

Mother- I stay at home and do not work.

Maternal grandmother- I earn, I care for child. I sell vegetables. My daughter's husband started going to work two months after the child's death, first he was staying at home and mourned for baby death. We too have been in mourning because of baby. I still cry thinking of the baby. When the child dies, who was brought so hard, when he was born he was in hospital for 12 days, my daughter had 6 bottles of blood then, she got her treatment in G hospital. The doctor there was also shock to see 6 kg child.

Other talk

Mother- my child was born in 9 months, there was no problem in child, all investigations were done at G hospital. I became anaemic at the time of having a child and had swelling on my body. The doctor checked the baby with all the machines he was fine. After delivering baby 6 bottle blood transfusion was done.
Maternal grandmother- one doctor was very nice in nursery, god bless her always, one day i started crying she said dont worry if you cant arrange blood from anywhere then i would help you to get from the hospital, 2-3 bottle blood was arranged from outside. when that doctor used to come then she would play and talk with my child.
Mother- i was patient to doctor in G hospital, she was very nice doctor. She used to see that if I was not feeling good then she used to say that what happened, if you have anything tell me, I used to say that nothing just I am not able to go home that's why I am upset. I got discharged after 12 days.

17 How your and your family's life has changed after the death of the child?

Mother- Ever since the child is gone, I have become more attached to my baby girl. Earlier both of my sisters used to take care of my baby girl and I used to handle baby as he was a small boy. My one sister is in sixth class and other is in eighth class. Both of them after returning from school used to take care of her. Love was not less for the first child, but as he was small, so we started loving baby more. My daughter was handled by her grandmother and sister, when I stayed for 12 days in the hospital, then I would miss her, now my baby girl is going to be two years old.

Knowing cause of death in detail- Autopsy and MITS

18 What was the cause of death of your child and what is your perception about the value of knowing the cause?

Mother- I want to know the cause of death, I need to know what caused our child to die. We want to know why our baby died.
<Next pregnancy> yes It is also necessary for the future that no such thing happen in the next child. If my child was alive then we would take no chance and do not have to think about the other child.
Maternal grandmother- If a child is poor or anything else, it should not happen. This should not happen to anyone as it has happened with us. Right now not thinking that there should be a second child, during this pregnancy then there is a nurse in GTB, she was very happy, bidding my baby girl that now you have two children, it is enough and she was very happy and prayed for her, the nurse brought bread from her house, she was not a nurse, she was a doctor (Mother), the same doctor said that my girl is short of blood, and the next child be a threat to your health.

19 In your view, what more could have been done to know the exact cause of death and the reasons causing the disease?

Mother- You will find out by having a test, but now nothing can happen, what was to be happened has happen. Many children die in S Hospital, many children were finished in front of us.

20 Are you aware of any method for identifying the exact cause of death?

Maternal grandmother- You people will know the reason for death, by which reason our baby have died, we are illiterate. If I will not earn anything, how will I eat it, and how will I pay rent. Only you guys can tell how death happened. It is here that we cannot do anything but we can appreciate you, when you tell then we can come where you say.

21 What are your views about the autopsy/ post-mortem for identifying the cause of death?

Maternal grandmother- The child cannot have a post mortem, anyway he has been buried into the soil. I have no idea why the post mortem happens.
Mother-I know that post mortem happens when someone is killed by hanging, if someone consumes poison then post mortem take place. Post mortem of child cannot take place. It is not allowed in our religion also. The person who has an accident has a post mortem in the hospital itself. If his heart is alive, so doctors take out all organs by doing post mortem.

22 What are your views about collection of the tissue (like done for biopsy to diagnose diseases) and body fluid samples for identifying the cause of death?

Mother- This technique is good, there is no tearing in it, people of our religion will agree for this, there is nothing like it.

23 How parents and family members can be approached for the tissue and fluid sampling?

Maternal grandmother- Nobody told about this technique in the hospital, I wouldn't have agree to perform this, as for now baby has gone, it cannot come back.

Mother- Like when the doctor told us about the death, then he should have told about this technique, all this should be told by nurse and the doctor.

<Whom should they tell> if I was not in that state, then this technique could be explained to child father or to my father-in-law. At that time child father was alright, doctor would have told to him about the procedure which can tell us about the cause of death of our child.

<How should they tell> doctor would have told this verbally.

24 In your view, what factors are important for acceptance of the tissue sampling by the parents and family members?

Mother- no one told us the about the cause of death at that time, they only told the child brain nerve has burst, there is bleeding it first started from mouth and then from the nose. we should know the reason at that time only, when our child was normal, how did this happen.

Decision making dynamics

25 In routine day to day operation who takes decision about the major activities (purchases, treatment, spending money) about the family?

Mother- For routine activity decision is taken by my husband, for any treatment decision is taken by my father-in-law. My husband does what his father says. All the decision in our house is in the hands of our father-in-law.

26 During the hospital stay who discussed with the doctors/ nurses to make the decision(s) about treatment and other procedures.

Mother—Nothing was told to us in the hospital, so what decision we take, we were just sent from one ward to the other ward from the emergency, and they did not tell anything in the whole night. We were just pumping bag and tube all night. They were not saying anything when we were asking.

Maternal grandmother- When my sister-in-law came in the night, she was saying that the child is fine, no need to fear.

27 After death of your child, who decided about the cremation and other post-death activities and rituals?

Mother- All decision were taken by my father-in-law, he only gave money for shroud, during burial. When the child dies, it costs less money and the elder cost more.

28 Now, we are completing the interaction/interview. Would you like to add anything?

mother- We tell you that in S Hospital, there is too much carelessness, G Hospital is still fine even child dies there, it is also a government hospital. Doctors do not let go deliberately, S Hospital is too dirty, no toilet is right and no water comes, nothing happens there. No facility of bathroom and toilet is there which is very troublesome. Where we were sent there was no water in the old building, we were kept in ward 18. In toilet there is no supply of water, even huggees are lying in toilet. The guards do not let anyone in. The guards disturb everyone. For us two person were allowed due to bag and tube, even if we had to ask something from doctor, the guard would show up, not allow us to enter. There should be place for the attendant in the hospital. All government hospitals are like this only, they intentionally mess up. One thing I must say that the doctor in hospital does not pay attention properly, so all this happens.

In-depth Interview- Parents of Deceased Child 4

**1.1**	**Primary respondent (relation with the child)**	Mother
**1.2**	**Age of the primary respondent**	Mother-25-30 years<br>Father-25-30 years
**1.3**	**Other members present**	None

Context- Her child was admitted in ICU at S Hospital, the total stay of her baby was 25 days before that baby was getting treated at K hospital.

2. Events that led to death of the child

2. Can you please let us know about the illness and events that led to death of your child?

When she was born, she was very weak, had jaundice. The delivery was done 10 days after the scheduled time. Then after that he had jaundice. She was kept in her S Hospital for a week, then she became healthy. The doctor said that the child is very weak.

Then it was good for two to three months, it was thick. But he started getting fever, I used to show him to the ordinary doctor, one day a very high fever came (102) and he was not getting well. The doctor said, put a bandage on the head. I put the bandage on. His chest became jammed, with it running cooler overnight. I was alone and the other child had to leave school. It was raining outside, sometimes I used to get wet in the rain, we had to feed milk. Due to crying, we had to be fed immediately. The child was taken to K Hospital due to excessive cold. Was admitted there for 15 days. His money was off. There was a pipe for urine. When he pulled out the pipe, blood was coming out. I got angry after seeing this. The child was screaming and the doctors were not paying attention. In this way my baby girl will die. My son was watching all this. His mind was getting worse. After the debate, he said in K Hospital that you take the child. I said stop now, it has urinated, it will be fine when I give it milk. But the doctor there does not have a treatment method. If we had money, we would not come here, go to a private hospital. As soon as he got out the oxygen pipe, he started having trouble breathing.

After that, the child was taken to S Hospital. Admitted to ICU there. Admitted there for 25 days. There was swelling in his body day by day. The girl died 25 days later.

Was admit there, then day after day, day after day, it was swollen, it was swollen and then it was over at around 12 in the night, did not tell what is the disease, it was said that his chest is not this lung. It is done, and brings this medicine, bring that medicine, do this, do that, after 25 days of admission, it is over.

3. Hospitalization period

3. Now we would request you to tell us something about the period of hospitalization for your child?

<Duration of stay>

15 days in K Hospital and here (S Hospital) admit in 25 days

<About HCP of S Hospital >

All was right, I did not go and forcefully took me away from time to time to make milk, the nurses give milk to them

<course of illness>

When I first went, put the girl in the emergency ward. He said that the bed is not empty in the ICU, then the doctor of emergency ward phoned the doctor with ICU and asked if it would be empty in the morning, these are small children. There was some pipe and bag in his mouth. Pump it up, he had to press only that night in the emergency, then my hands started hurting all night, one night I did not sleep there (kalawati) for 15 days, then my father arrived at 5 o'clock in the morning, then he pressed for a while. Then it got accepted (ICU) at around 8-9 pm, so we were thrown out, there was no loss in his body, so how to deliver medicine to him, he was bald, dipped in his head In my mind, I used to give glucose or medicine to him, then after two days, after applying such swollen medicines, I asked the doctor how Madam it has swollen, so he said that you are looking swollen, I said I have a baby girl, I do not know, she was such a bone, if she was swollen, she would have done something, she did not say anything, she has come here to get treatment, not to answer the question, she said that and

then after that The doctor who did not come in the round, in the morning there was a lady doctor in him, he had also said this that he is telling me that we are trying to save him, if the son will be right then it is a good thing or not We are trying our best, so those people who used to speak and who used to keep it during the day, the doctors used to decide how much milk to give from that medicine, and the reports that were made during the day were filed. I used to check the paper which I kept, then I had to feed him after two hours every day, then later his stool stopped, after 20 days he was completely fine. He was a doctor again one day, Who speaks the language, he was not an Indian, he was black, long, good, very normal, talking like ordinary people do, so he called me that now it is difficult to save, I said what you will tell, I am his mother. I can feel every beat of him, I touch him, then it seems to be over, his eye did not close like these 20 days later, when I opened his eye like this, it looks like water starts coming out of his eye. Father, where he works in the Kothi, he has a domestic doctor, he had taken it to him earlier, he meant that some specialist of the child was such a doctor, he once in private, he once meant money from the Kothi. To give my child a treatment, he wrote that S Hospital said, "Take it there, there are good nurses, there will be oxygen. You will not be able to treat it here, so we took it there when they started removing water from their eyes." I understood that its entire body is filled with water, now nothing can happen, then they explained to me, I thought that what was to happen is in the hands of the above, now what do they know about it? Doctor) I do not even understand Hindi as much as the people who used to talk in English, who used to explain to me, I used to understand that, they used to tell me those people. Once you said about my baby, you give your milk or you feed it on the top, I said that if there was no milk, then I mostly gave it the top milk, which one is it, then I said whatever This 21-rupee packet does not come with a pouch, it used to drink, I used to drink it, it was suppressed, the girl became very good to me, then after that you said, bring milk from the dairy.
I did not say, I will not get it from dairy, I have spent so much money, I will bring a packet of 4-5 hundred rupees, I will give it by heating water, I do not know how the milk will be, so if I can buy it then why I did not say Will I be able to buy, can I not buy medicine here, I do not buy, I buy it every day, then they gave it in writing, then it started feeding, then it was good, at that time I was thinking, my baby is well , Two days later I do not know what happened Madam, she did not realize it, after removing it from here, she again injected it, then she did not feel conscious, meaning there was a needle here, there was a needle With, soldering that one, then it was applied in this thigh, if it was not applied in this thigh, the blood was not going to stop, even after it was finished, its blood was not stopped, which is applied by the solder. The needle was made of plastic, then it was finished at 12 o'clock at night. Then 4-5 days he (doctor) did not call me, he used to call only for medicines, then stopped his milk, he stopped urinating, latrine too. The same people used to see a doctor for 24 hours, then other doctors used to go inside the ICU, that is, they call time after time, like those people call after half an hour after an hour, which means that those people are negligent in the day. Did not, now at night we cannot say, now there were two doctors who used to treat for 24 hours, that means they used to stay in front even if I went out, sometimes I used to go for feeding, then the nurses did not come forward to everyone. My child used to come here after feeding me. My child had 14 number beds. So it was the last of all, then it used to take time to make milk and these people used to go to talk on the phone too, two or three doctors were very good, they used to say, come here to nurse and sit in two minutes. . There were two madams, both were Priyanka. A South Indian was a doctor, he was good, he was also very good, and he was very good, and he was like a rage, if we have children, he is in a mess, then we will make it clear, he spoke to you or something like that in some English, we do not understand anything, do not do that. Now if there is dirt, then we will clear it, otherwise the infection will spread, if we change the sheet one day in the morning time, the nurse is saying that it is clean to the children. A packet does not come to clean the potty of the children who are from the throat, I used to clean their body every day because they used to remove blood, neither did the blood sit anywhere in the body, so it was said that if you keep it clean Would be so nice Most used to call me because I was a small child, saying that you should come and try yourself in an hour or two, I was allowed to go inside, the youngest was the same, so the guards did not stop me. I used to look at him, meaning someone is a nurse, he used to say a little harsh, so one day a doctor came, he was also on the round, I was inside him, which meant that the doctors who came round had come. I said that I was doing it very clearly, in such a long time, those people were 4-5 doctors used to come together, these people were also together, they used to talk in English, so I am telling my son your baby girl That

pneumonia has worsened, we got it done several tests, that means x-rays were getting worse day by day, then showed me that it was a board like a TV, it was switched on and it turned white and then the TV became white again. After that, I put this X-ray again and showed it to me that I did not understand anything or else said, look, it has started to be white, when it was first done, it was so much, after that it happened at least 8-10 X- Ray got it done, did not explain it on the same day, when it was over, the girl was alive, explained that day, and the same night the girl was finished, the same doctor came that day was very good, explained to me, he is saying son Look we tried Do not say that it is the doctor's fault. Now we are not so educated, what did those people talk about in English, we used to just stare, then those people used to tell medicine, then I do not know what they used to say in English, they used to tell me that you should not stop going, medicine. We used to give expensive medicines from there and then we used to buy them from outside, outside the main road there are all those medicines which were used to buy medicines from there. <about cost> At that time, I did not take that much attention, as they kept writing medicines, writing the goods, buying them, it was more important to save that time, one was upset that here it had become typhoid (live child). , I was worried about taking two children, I had no one here, I had left the child near the neighbour, after seeing these people, my father-in-law came, so for 15 days, he was ill, I could come here, there Was not able to live either, I said it will also go, it will also go, so let the above one, that person has become lovely. After that, it hurt me more, it sat on his mind, he was passing urine from the pipe, neither did he act like crazy, he had seen it, he loved it more than his sister, most of the night. In the same mobile till two and a half hours, both the two sides used to listen to song in the mobile. If I would put sleep there, I was lying on the pillow of the clitoris, both of them (live child) still know that its sister S Hospital is with the doctor, I did not say that the doctor is a doctor, so I am happy that my sister is there If we say that she has gone to the one above, then we say no or she is in the hospital. He is 6 years old, so later the doctor did not speak it and did not sit on his mind, he did not take the child to the hospital, he was crying a lot to see his sister, the doctor said yes Show it for 10 minutes at the same time that the scandal took place, playing before that, both of them were playing a lot in mobile.

4. Could you please describe your overall experience about the hospital care?

<About treatment>

I liked the way they talked. You cannot know the method of their treatment. Often, check slips were given to me. People who did not understand, used to explain. Where you could not go like OPD then send it with someone there.

5. Could you please describe your experience about communication by the various hospital staffs during hospital stay?

<About HCP>

Doctors used to explain in Hindi, they understood it. But he told us directly. That the child's pneumonia has deteriorated, otherwise we will do our best and it is God's will. We already knew that is pneumonia. The heartbeat started growing at his home. Foam started coming from his mouth, he was very much. Previously she was taken to the private doctor. Bola is pneumonia. Now double pneumonia is done. Take it to the hospital soon, then I took it there. At K Hospital But the treatment at K Hospital is not as good as that of S Hospital.

<About communication of HCP>

I used to cry day and night. Nobody used to bother me that much. Spoke directly. I used to live alone. One day the doctor told me that no one is with you, so I said that my husband goes to work during the day and here at night. After all, money is also necessary. There are so many expensive medicines here. If no one does the job, the treatment will be from where? My little child used to do small things in the hospital during the day. Like bringing test report from another building, bringing some similar purchases during the day. Right now my boy is very small. When there was some urgent work, the husband used to take leave from the job.

<Primary communicator>

Nobody used to tell me clearly. Every day I used to ask them how my child is today, whether to feed him or not. He used to say less when asked about everything. He was a doctor and he used to talk a little bit and everything was fine.

<Skills of communicator>

There were 3-4 doctors who liked the best. A doctor was a bit mean, it was not bad in the heart, none of the meaning was like they do not put pipes and say what they say inside the mouth, do it in English,

when the foam increases. He used to do it in his mouth, as if he was late for two minutes, the nurse used to come and shout, the doctors were saying "Do this work first", they used to speak in Hindi, otherwise I don't know if there was any patient. Some who used to get exhausted, a lot of men came and abused them. I have seen how many corpses I have seen in 25 days, I do not know there, I used to take out one or two every day, I mean I have not been there every single day, I have not seen a single day coming out of ICU, in three days in 25 days Since then I also felt in my heart that what these people used to explain to us, they used to do something else, they were unable to understand. Even a patient should come right out of it from the ICU, no one came, there was a girl, what she had come to speak, after four days from UP she was in my number 13 and my daughter in number 14, So after 3-4 days, he was transferred to OPD, after a week, he was brought back to the same bed again. He was negligent in OPD. I mean, the doctor brought 4-5 operations to his ICU, then put the pipe in it, the doctor came from outside, to do the operation, one has to clean it with water, which used to wash the kidneys, then a doctor always chairs But he used to sit near the patient, meaning the child's doctor was negligent, he was a little lacking, whenever he used to go he meant duty, he used to sit in a chair and saw how much water was filled under the measurement, how much was left. Used to change immediately.

Death and post death period

6. Could you please tell us about the death and death declaration of your child?

No one came to tell me that my heart was very nervous at 12 o'clock in the night, I was sleeping, I went and asked the nurse to feed the child, he said that the baby has to be stopped. His toilets and potty were already 2-3 closed. One day the condition of the child had deteriorated, there was a lot of water in his eye. It seemed that now my child will not be left. I started crying. I thought if there is luck, then the child will be cured or else God will snatch it. People explained to me, said, "Go out and come and eat something, or if your health is bad, you will see the child." I sat outside for an hour, then went inside, at night when I was feeling very nervous, became sweaty, sweaty, I used to walk so fast AC in ICU, we used to sleep two or three blankets. , I was completely wet in sweat, then stood up, then I come to see once again, how is it that when I went to the girl, I could not see the machine directly, one day if I had explained it to me in Kalavati It will be above 90, neither its heartbeat will be right, if it starts coming down from 90 to 70, 80,60, then it was my attention to call us immediately, then I did not see my daughter, I see above, I am machine locked. Then I paid attention to the bed, then I see that her (dead child) dam is kept blind, so if I went inside, I was not going inside any more, then I asked the doctor, doctor sir, like I told the doctor, till then he started speaking, his father is there, send the father, you go out, I picked up his father. I did not say right I have come to know, what is the matter, tell me, why are you so nervous, I have not said - do not go, the doctors are calling, then after seeing it they finished it. He (husband) was a doctor thinly, who used to do duty during the day, that was also good. Doctor means three men were doctors and two lady doctors were cheated, five meant that they used to see all the day, so as its father out. When he came, he said to me, "You sit here, I am praying from outside, I started to fire after them, now at that time no one was able to stop me."

7. What was your and your family member reaction to the death of your child?

I was crying a lot, my father was also crying, then he called his relatives, all four of them are on fire, this is the same, when they came to the hospital, I was looking at them and was crying a lot, so its Father said, take it home, I myself forbade him, everyone was speaking, take it home, its grandma, grandpa is at home, everyone will see the child, my son will one day see if he is sick yet, then he will see in such condition No, what will happen then it is gone. The second thing is, I live in a rented house, I know why the tenant has brought here, where he was so ill, he had brought the infection, spread the dirt, then my elder brother, Tau, uncle's first elder. Then there were uncles, she was saying that she is very right, and it was a bit strange that she was so weird, so I said don't take it, then she said that she presses the little child, so she asked the doctor So the doctor said, we do not know whether the people who are driving outside ask them with the ambulance, they will know them, then they asked if they asked outside, and told a place, Kalindi Kunj had told something like this. Take the same thing, bury the child there, first we used to stop here, then we stopped at Rohini and then I called the father-in-law here at night and told the neighbor. Then we live here very well, in whose time these days, who is there in 15-20 days, I put the child in their hands and got these people treated with their money (), their parents are not there Later, he came and gave me the breakfast of the morning, the

child used to send the lunch, the woman who sent it to school, I used to take care of it, I was carefree, I could even call my jewelry from my hand and call it Therefore, we do not have such a good house, we pay more rent, at 12 o'clock at one o'clock if there is any problem, then we give it to each other, that is, there is 8-10 women working here and there. Reached me, at 12 o'clock my daughter was finished, at one o'clock these people reached me at night, then we went to the same place with me, they all took me too, so I wanted to see, show my children a little, So the doctor had refused to do it earlier, its (dead baby) was not grossly spoiled, do not open it, the doctor had tied it, its (dead baby) used to remove a lot of dirt from the nose, a lot of dirt from the mouth, do not touch your hands, infection will spread, you Have more children.

<Cause of death explained>

Anything told, it ended, at 12 o'clock, it was over, and it was told to us that the pneumonia has worsened it, the lungs have worsened, but what was the reason for it, nothing was told Madam.

8. How was your experience about the procedure after death till handover of the body to you?

<About documentation>

After the death, the father of the girl gave the discharge paper, he gave it to me, what use was it when the girl died. Then on the same day the day my daughter died, we would eat and drink food during the day, in the evening we were sitting outside in the hall which was made like hall. Whenever he used to call, he used to go inside, there was a girl from ICU, went to college, his mother worked in the brick bhatti, her husband lived in the village. She used to talk to us. My daughter has become like this. He has undergone complete surgery from here to here, he is not wearing anything. It is kept just like this, I go at night, I am scared in myself, to see my child. God says my child is right, if it is not right, if it ends, then if something happens, I will give a lot of bad to the doctor here, I cast a lot and shift my survivors, I hope so, on the same day At 4 o'clock in the evening, her child died, her mother gave such a badge, she was crying even more to the doctor and to the guards. She was going to faint again and again. Said that these people killed my baby girl. For this kind of disease, the whole child tore my child, from here to here, saying, I do not know if the infection has failed, I do not understand anything, then another death occurred on the same day, he also spoke very bad. We have not even spoken as much as it means to do anything. As soon as we asked questions, lied only, but only understood us, we understood, thought the doctor would be speaking right, the doctor is in the form of God, when everyone speaks outside, when someone goes inside the ICU, Goes to die nowadays no right hock returned. Meaning that other people who live outside, they say that we have been living for 6 months, some say that we have been living for 3 months, some say that we have been living for 2 months. I did not talk to anyone as long as we were there for 25 days, when I was crying a lot, neither many people gathered at night, then everyone started speaking. Everyone used to sleep on the bed outside.

9. Who all from your family and community/neighbourhood were present at the hospital and how they supported you during this period?

<Who all supported you>

His father called his mother, he called my father-in-law, then the father-in-law told his mother-in-law, at that time my son was very ill, then the father-in-law had not gone. If you go, then you will get the bath, then I said that the bath will be in your house or not, if you go to take a bath inside the bathroom, then everyone will say why are you bathing a child with infection, then I did not bathe, went direct from the hospital.

<Who all supported you>

All the people were there, their father was crying a lot, then Tau, uncle were those people.

Post death procedure and coping mechanism

10. What all rituals and procedures did you follow after death of your child?

<Number of people was going to burial place>

I, its father, its grandmother, uncle, two aunts, 10-15 people who come to work at night, can do it at night, we can do it at night also, and there is no prohibition.

<Timing of burial rituals>

These people came to the hospital at one o'clock, then we sat there for a while, saying, stop now, you are going from there asking this car, you are asking for more money, then it took an hour to find the

car, it was two o'clock then there Everyone sat in a car and then we came, meaning those who went from here, some people went in the car with the corpse.
<About neighbour> those people were in their own car, so it was three o'clock on the go. Then while talking there while we were trying to be in the mosque and buried it was also dark around 4-5 o'clock.
<Who performed burial rituals> its father only did everything.

11. How did you, your spouse (wife/husband) and immediate family members cope with the difficult phase?

I was faint at the time when these people went to bury him. For a month and a half, I was not getting food and drink properly, I was a heart patient myself. I felt at that time that I would die. I could not see anything, everything seemed like a dream. I was dreaming that I am playing with my child, like in the house, I opened my eyes, I fell asleep crying, a woman says, I empty her from here and go away, that means her on the shoulder She was sleeping with her head still in her arms
I opened my eyes once again and I started crying my daughter then, I see these people coming from the forest, I was scared to see them, they had taken two rooms, one was in her circle, one was that if there was a room, he emptied it. I was crying a lot there. I came home for a month and a half I was right, after that it seemed as if she (dead child) came and drank milk.
<When after death>
After a month and a half, she was born, since then my period had not come, on the day she was 13, my health was very bad on that day. Had 103 fever. Four days ago, I had so much fever, 103 did not come out, I had the test said that typhoid is there, typhoid medicine used to say that I used to have more fever. 102 Fever used to be like I used to sleep or did not feel like my baby. I am giving milk, the bed gets wet, I do not know where the milk was coming from at that time, when it was alive it was not milk, I used to get the bed wet, so my mother-in-law said, in the custom is to put milk in the bowl and mother would do it, I used to do it when the cat came and drank milk, then my son felt that the sister had come and drank milk, so this (live baby) night When my health got worse, then one day he was sleeping, he started crying while sleeping, his hair started scratching, the head started banging on the wall, he said, "Why did my baby leave, why did you put money in the piggy bank?" When the Raksha Bandhan comes, my sister will tie a rakhi in my hand to bring the doctor an hour of speaking, because I used to call her (neighbour) girl every year and get her tied, it was very nice to get her sister tied, Now some child said that your sister has died, someone has gone inside the soil and someone has told her in my mind, I did not ask my son, when he was ill, he used to say that the sister will come to the hospital. Now later the doctor said not to keep this in his mind, to tell him slowly, otherwise he will remain in his mind, but now we speak, he does not believe it, he still knows his sister in the hospital is.
<What rituals performed after the burial>
They do not worship. But washing clothes, temples, pooja lessons did all this and then after a month and a half I became very ill. People used to come to see, then they said that the daughter also went, now the mother will also go, so in our Calcutta there is a lot of sorcery, then people used to say, do you know if it will be possessed by a ghost, the mother is after the child is over Children do not find the father, they seek the mother only, what address does the soul of her (dead baby) bother her. Send it to the village, so when I got blood test done again, the typhoid came out, the doctor said that its typhoid has worsened, pneumonia has happened, at that time I did not even have much money, I spent my money in his (dead baby) affair. It is done, debts were also incurred at that time, so then (her) owner gave my ticket. The owner is good, sent the ticket to the village. I went there and got medicine, medicine is also of no use to me. I mean, Baba called me, he means I have perished and said that he has settled in his blood, who is dead inside the hospital, then he has to come out, then asked me you see something, I said I get strange sounds. Hai, I see a woman, wearing a black sari everyday and doing havan from the front and wearing a big dot. Big hair used to look at him, close his eyes, be full of his eyes, he would look gross, so Baba said, yes, its evil thing has entered its body. They dusted me in the morning, they say dust in the evening, till then the people live, they are still alive, when it is finished, it becomes the devil means we believe it, so like Madame dust me twice in the morning, in the evening, like in the evening My body did not stop sweating for three days and I fell ill on the 5th, my ticket was on the next 5th, went home, reached the 6th, my treatment started on the 7th, it is the month of October, I have just come on the 5th, then took them to the doctor, then he said that if he is sweating, his body is getting cold, then do not give medicine for fever, he becomes a person and

becomes ill, cold is one and fever If you give medicine then it will get cold, do not give antibiotic yellow and give medicine for fever, if fever comes, then I give puja recitation, we get it done in us, nor can we go to the temple until it is worshiped, worship Cannot do, I cannot feel festival. I made him a great pundit, he knows a lot, then he said that if you will take a child within this year, you will be left or neither will that child bother you again and again, the body will ruin it, So you have to do a lot, then we did all that, and the child looks at me, ma'am, still looks like I am giving him milk, so he said that he tries to come here, so he has to stop now 2018 If this is not good then I saw her (dead child) horoscope, when she was born, we told her that it is like this, so my horoscope is mixed saying that she is trying to come again, so she has to stop this year after January. Meaning 2019 will take you in it.

12. Is/was there any change in the interpersonal relationships between you and your spouse and other family members after the event?

There is no such change, they just take care of me, I cry to them, as they (husband) at night, when I open my eyes, I see that they are not asleep because I have just given birth to both children, but care for them (both baby), I didn't know anything. When I got married at a very young age, it was the cleaning of the latrine, the feeding by her husband, then she taught me, then later on, when I started taking care of them, so they have a little more.

<Relation and attachment with other children>

Yes, more attention is paid to the other child, but it still needs a sister, then I will talk to the doctor if they will say what they said otherwise, if they want to take another after 2 years to defend it. You cannot take it now it has not been even a year.

13. How did the family members support you and your wife during the difficult phase?

My mother and father have done the most to me, be it with money, whether through treatment. Had my mother been here a few more days, my health would have deteriorated (mother's health). Everyone said that admit it in S Hospital. My children died. I saw that there is a useless treatment, if any good happens, it will become ill. Some do not pay attention. They give injections anywhere. The blood keeps coming out. I am very scared to see all that. Now I will die but I will not go to that hospital.

14. What were the reactions/ counselling and suggestions given by relatives/neighbours/ community after the episode?

15. Do you think and/or discuss with your spouse/ family member(s) about the death and related events and blame someone/something or feel guilty for the same?

I think the same thing happened in Kalavati because 36 times a day he used to extract blood from the needle in his body. Those people did not mean much. Like when to feed the baby etc. There is a separate machine for the person who is Aamir, different for the poor. The poor one goes bad. A child has pneumonia. When the AC is running, it gets colder, then cold milk will go in and it will cool, now I did not have my baby with me, I was not getting salary, I will not lie given the same milk, my father works , Which was taken to me for 15 days, so I made me believe that my father used to go to get 1000 rupees of his daily, he used to go near it (live child), 250 rupees auto, then from here I used to go to 250 rupees auto in the morning, then from there they used to come to Malviyanagar and Kalavati for 250 rupees autos, then they used to go to reach me at 9 o'clock at night, meaning poor had to go to four places four times a day. Twice I have duty once here.

16. When did father and mother (if working) returned to work after the death of the child?

The owner of the company himself said that I had kept another driver, as long as your child is treated, then you take care of him. The Japanese company said it was not going to cut your money, it is good means a foreigner, but it is good, then I finished the whole month, gave it to my father for 20-25 days. Used to call for hours, then they used to ask the doctor that there is no work, because my work was outside. These people used to get me to go outside to get a report and go away, some work was done, the outside work was done by the man and the inside work was done by the woman. His father went to duty after finishing two or three, which means it has been four days, and after that he had gone for five days.

17. How you and your family's life have changed after the death of the child?

18. What was the cause of death of your child and what is your perception about the value of knowing the cause?
I do not understand if the girl died due to doctor's negligence or because of my negligence. Sometimes it seems that the doctor did some reverse medicine. Then, it seems that if the medicine were upside down, many people would have a problem, but yes! There is a lot of carelessness in the treatment that is done in OPD. One girl was very healthy. She had gone in front of me, said mother, I will go home and have food. She went there for three to three days, it made the situation worse, the doctor also makes mistakes, but where do these people (doctors) believe. Now I cannot enter the treatment, how was the treatment done. Now that she has left the world, what reason should I say? You have to ask your father only, he will take the decision.
19. In your view, what more could have been done to know the exact cause of death and the reasons causing the disease?
No I don't know anything
20. Are you aware of any method for identifying the exact cause of death?
They all say, they check, what is not there, then they find out whether it was caused by the doctor's medicine, if someone dies like this, they kill, then it is known in how much time Yes, a woman told me there, she used to work there, there is a post-mortem, those big men, they are disgusted to clean up the potty people, then people came If one day she was eating food, then she used to shift to go daily, tell someone else if needed, then share the money in each other, so I said, come and eat the food. What have I brought, I have brought it from the hotel, I have to eat, I want to eat it, I have brought Bachura Aajao Chhola, so my husband was saying my husband is over, I will not burn him, I will get him posted and someone else will say that in the hospital. She came, was saying, the doctor killed my man a lot. He ate food at night, the one in the front, the one in the front, the one in the side, see it is over at 4 in the morning. I do not know if I have given any medicine or Gave needle, bleed from nose you done, speaking like this, then the aunt was telling the post-mortem that this thing happens if the medicine is over, neither will it be known that the doctor gave the medicine wrong.
21. What are your views about the autopsy/ post-mortem for identifying the cause of death?
I would have thought of having my baby girl. Now if the girl is there or not, then what is the benefit of the post mortem, if there is a death in the hospital, then it must be done once, it will be known that it is your fault or the doctor's negligence. Must be done once.
<Religious aspect>
No our religion is not forbidden.
<Disfigurement>
we have heard that people speak, take out kidneys, do not know what they do, they tear it out, they tear it all, doctors all sell it, now what to tell All is true that some people say lies, some people speak, doctors take all these things and those who are short, they want to stay alive.
22. What are your views about collection of the tissue (like done for biopsy to diagnose diseases) and body fluid samples for identifying the cause of death?
This is right
23. How parents and family members can be approached for the tissue and fluid sampling?
<Who should approach>
Like you - people have come to ask that if someone like this can go to them and ask something, the doctor should tell himself the size that you do this thing. Some- doctors are good, one had TB disease there, whenever she used to come and not inside, there was no patient, who used to take care of them, then she used to say that you should wear gloves which are not in the mouth. If you wear what you wear on your feet, then your germ will go into someone else, someone else's germ will come in you, the doctors used to explain it, the doctors did not explain who were feeding, the men were weaning and There were also nurses, these people used to say all these things, the doctor never spoke all these things, he had explained to them, it has been kept here to wear all these things and then go to the patient.
If the doctor himself told me that you should do post mortem or my man, then we will say that yes, I should do it once, as if I am a mother, I would control myself at that time, someone used to say, While speaking there, who will take my man to someone else, I will get him tested. What is the fault of the doctor? If I had this in my mind, I would have said the same at that time. I knew that my child was suffering, he (the doctor) did not leave anything that I doubt on him. They (doctors) already knew that

this would be over, now these people used to pay a lot of attention to the children and there is negligence among the big people, nobody saw three children among the children were children 13,14,15 Only children lived in them, there was very little negligence in them.
Earlier, I did not even know the language here myself, now I understand and speak.
24. In your view, what factors are important for acceptance of the tissue sampling by the parents and family members?

Decision making dynamics
25. In routine day to day operation who takes decision about the major activities (purchases, treatment, spending money) about the family?
My father does everything there, my father lives in the village. If I want to do anything, meaning I want to do any work, then I definitely consult them. <About treatment> This would decide its father
26. During the hospital stay who discussed with the doctors/ nurses to make the decision(s) about treatment and other procedures.
When there was an admission in the hospital, the report that the doctors used to give, which means, mostly I, they were afraid that its father is very timid, my child stayed inside for 25 days, he did not go, and he was very scared. Because there were very terrible patients. One day I was scared myself. I myself have been ill for two months during her affair. Now I did not know, that girl is finished, I went and laid her hand on her bed. I was absolutely scared.
27. After death of your child, who decided about the cremation and other post-death activities and rituals?
My uncle was doing this the child is buried in us. When they went there, they said that they had to buy land for it, then they took 2100 rupees and then buried them there inside the forest. I do not know where my father was buried, then the father was saying that his hands were open, so he meant the soil which he makes by digging the pit, who cares there, he did not ask for anything, those people know Have not said, put money in his hand and then put mud on it, but his father closed his fist with 101 rupees in his hand, then put mud on it, then when he came from there, his father said It had something, it was something of the last birth, they came to do it, put money in his hand, this was told by his father later. Those people do all this day and night, neither the soul is not afraid of the soul, that is, they know a lot by doing something in themselves, because these people only do this work day and night, burning, then they know everything.

In-depth Interview- Parents of Deceased Child 5

**1.1**	**Primary respondent (relation with the child)**	Mother
**1.2**	**Age of the primary respondent**	Mother-25-30 years<br>Father-25-30 years
**1.3**	**Other members present during the interaction**	None

Context- father works in marketing line field work and his age is 30yrs.
Not just this incident, these stresses are there since marriage. I think because of stress and tension the child has got so many problems.

Events that led to death of the child

2. Can you please let us know about the illness and events that led to death of your child?

There was a normal delivery but the child had a lot of problems, he also had cleft lip and cleft palate, we came to know after a month that he also has problems in the heart, when we were taken to S Hospital for the first time the doctor said directly that the operation will be done. We were not sure that there would be a problem in the heart of the child we thought that as we first came to know about cleft lip with ultrasound, the heart problem should also be detected by ultra sound. We brought S Hospital for the same reason that it was born. Still we were not able to believe that its heart really has a problem, but when eco, etc., found out in S Hospital, it had a lot of problems in its heart, the pipe was leakage and the baby was born After that, the vein that stops the natural, that too was not closed. The doctor sent him to the operation in the SSB building in S Hospital. But these people said that there is no operation of small children here. Then we were sent objectives. When the doctors referred to the aims, I asked when the operation should be done then the doctor said that in one to one and a half months. But in the aims, it had been a month of circling. And these people also only spoke to aim, did not give anything in writing. If these people had given anything in writing, or if the operation had happened quickly, then my child would have been cured.

Hospitalization period

3. Now we would request you to tell us something about the period of hospitalization for your child?

When the child was diagnosed with Pneumonia, he was first shown in freezing private, when he could not get rest, the doctor said that you should either go to our place or take us to S Hospital where the child is born. So we also thought that he took the same place where it was born, because it also had a cleft lip problem and the doctors in S Hospital are also good there. We went in the night and went to S Hospital in emergency. Stayed in the hospital for eight days, this was the first time. Then get the eco test done, which had a very long date, 15 days.

We also came to know from this test that the operation will have to be done. We were running here and there for the operation that the child used to get infection of Pneumonia in between. Everybody used to say that operation, operation is the only option. Those people had told that its operation is not done in our place, and those who have heart problems get pneumonia infection very quickly.

We came to the hospital five times in four and a half months, sometimes one, sometimes five days, sometimes ten days lived in the hospital.

<Duration of stay>

Admitted the number 20 in the ward, then we had been there for ten days then discharged and we came home. Two days later, the child had a problem again.

We then assigned the child where she was the first admit, in the ward number 18, the doctors told that there is Pneumonia. We had told the doctor that just two days ago, two discharges have taken the child, then the doctor told that this is only a treatment operation and it will not be here in another hospital, do the operation as soon as possible. This doctor had said, but we did not have enough money to get the child operated out. When we went for the last time, we were in the hospital for five days and on the fifth day my child was finished.

4. Could you please describe your overall experience about the hospital care?

<About treatment>

<About investigation> we used to go ourselves.

<About cost>
Nothing much had happened because he would have had the same infection of Pneumonia, then it would not have cost much but when I lived in the hospital, I used to see that people who used to write tests to people, they used to write from outside also, but our child did not have any tests done from outside.
<Who had supported you in the hospital from your family>
My mother and my husband were with me, whatever decision I made, I would take it myself. I had consulted my husband about the children who apply for breath because I knew that even the winning children had a separate tube for breath, no one was left all the children were dead. My child also felt that the doctor had done a lot of carelessness and did not put it properly the light did not burn properly. In one line, I can say that I was not paying attention properly.
<About HCP>
Behavior we used to talk in a very useless and dirty way, I also told an employee that we should take care of our patient that cleanliness here. He gave me the opposite answer, said that you should spread the dirt and cleanliness.
5. Could you please describe your experience about communication by the various hospital staffs during hospital stay?
<About doctor>
The doctors were all lost in themselves. Not good there. Some doctors were good, but more doctors used to speak very rudely. The nurse used to speak in the same way incorrectly. According to me, a senior doctor should always be there. They used to come to the round. Because if we had reached on Saturday, the medicine would not have started till Monday, because I used to go to the middle of the week and senior doctors do not come at that time. If any senior work was done by senior doctors, everyone had to wait till Monday.
<about nurse> The nurse was fine, I had a lot of attachment with 2-3 nurses, I had gone many times, so much nurse was fine, it was enough anyway, the rest of the number 20 is the same in the ward. So, we speak in a very eloquent manner, we are afraid to ask something again, whether we ask or not. The nurse was fine. Some nurses had become familiar. The behavior of some nurses was also bad. The doctor used to speak abusively. I used to be afraid to ask them.
<Who all communicated>
Senior doctors used to tell. They used to tell rightly whether there is improvement in the child or not. They used to take this decision only if they said that they do not have to give anything to eat and drink, then people did not give it, they used to come the next day and tell how to treat it, what to do next. The condition is fine for the child then he used to say that the doctor said yes, something can be fed or otherwise.
<Frequency of communication>
They used to come to see only one, senior doctor used to tell only once in a day, understand.
<Skill of communicator>

The method was quite good for the senior doctor, otherwise the same people used to go, they can do this to ask, can they milk or not. Yes, he used to answer well.
Death and post death period
6. Could you please tell us about the death and death declaration of your child?
At that time only I and my mother were in the hospital. It had happened in front of me, I was there, it was not known, when he was applying oxygen or not, while applying oxygen, he died. When the doctor was putting in the death, he was putting oxygen, first he said that he should suction every two hours when we know that our child is serious, then we will come to see the doctor or we will go there again and again Had to ask whether to suction it, not to suction it, the doctor used to look at it and said, "It is not working right now, it has to be done suction, then its oxygen pipe was completely tight since morning, then I said ma'am Tight is going on, then again saying that it has to be suctioned, then they sent a band who was learning things there and while my daughter was serious, at that time, the doctor sent such a band now to her Do you know how to do it so quickly, do not have to do it because oxygen is not going that little at that time, if done suction, they were doing it by removing oxygen, I was there at that time. Just like they were doing the massage of the heart and the people there, then after that, they had applied injection to increase BP, to increase the rate of heart, then it remained

close to them for a long time, if not, then we said that we had injection Have you tried it now, watch 10-15 minutes, then I was giving oxygen from the same oxygen pipe, then after a while, the doctor told me that the baby is not there. Earlier, it was said that it has become very dirty, meaning Pneumonia, and it was 3-4 days when he saw it, there was no difference in his breath, he started breathing fast, breathing fast, understanding the medicine It seemed like it was not making any impact, I do not know what treatment was being done, I did not understand anything.
<Who declared death>
At that time, he was junior doctor, in coming to the senior doctor, he might come around 9:30 in the morning, his (baby) death has taken place in the morning; At that time there were junior doctors, they did not come and told me that they were the same, mother was also there, when the doctors were removed, then they said that you try many times, the heartbeat comes, but at that time there is nothing like it Was not, yet I pumped for 10 minutes, nothing happened, nothing was seen, nothing happened in the child, then the doctor came and told me, he did not give any sympathy, he just spoke And went again.

7. What was your and your family member reaction to the death of your child?
I was just crying, I did not understand anything, at that time my mother was taking care of me, we were two people, when the husband came to know, his BP was also low, there (S Hospital) brother came. , husband did not come to the hospital. His health had deteriorated neither when he came to know. Brother, I had called him immediately (brother) when I was changing the oxygen, I had called him only then brother had sent me.

8. How was your experience about the procedure after death till handover of the body to you?
<About documentation>
Brother is doing
<Time taken>
The brother had come there when he had given it, and the sign was made by the brother. Did not take time there. Those people stayed here with us because from here, these in-laws, mother-in-low, brother-in-law did not go from here, they were saying that we are living there, then we will go there, so these people time to come got it.

9. Who all from your family and community/neighbourhood were present at the hospital and how they supported you during this period?

Post death procedure and coping mechanism

10. What all rituals and procedures did you follow after death of your child?
From there we came till one o'clock. Then I came to my in-law's house. The shape is then buried. Just like what happens in us, the bath was washed again after washing it.
<Who prepared body>
They seem in our relationship, uncle, they did, Yes she was from the in-laws' house. I do not know which cemetery they went to only gents goes there neither will my husband know which cemetery he went to. We do not grieve among children here. No, nothing happens in our children. Nothing normal happens after feeding because a small child is deemed to be a crime in us.
<About time>
brought home, then took him to the cemetery within half an hour, his soil was added around 4:30 pm and went till five and a half in the evening, and we came home from the hospital about one o'clock, yes around one and half o'clock Came near

11. How did you, your spouse (wife/husband) and immediate family members cope with the difficult phase?
Well, remember, I still do my housework, I do a little sewing, I do sewing at home, not outside, I do sewing already, from the beginning.
<Religious activity>
Yes, just praying for Namaz and praying for him, the rest is housework and nothing else.

12. Is/was there any change in the interpersonal relationships between you and your spouse and other family members after the event?
No there is no change in our way of life. Nobody handled me at that time, I took care of myself and did not have any support, after two to three days I went to my house and then after four to five days I came here.

13. How did the family members support you and your wife during the difficult phase?
No one comes around to support the neighbourhood they do not mean that our in-laws do not mean anyone. I was not even here, but the neighbourhood does not know what happened, what happened, what they would suddenly say they had just come to see the in-laws keep it from the three people the same people had just come because no one knew anything, because I was living in McKay. Even the in-laws do not speak the same, the above was their wish, she had her wish, she took it, she also had a lot of trouble, she would have recovered if the operation was done, we went out of the way, went to the curry, went to the hospital Had gone to max, Gangaram was asking for four and a half lakh rupees for the operation, now there was not much money, if it was done by operation time then it would have been fine.
14. What were the reactions/ counselling and suggestions given by relatives/neighbours/ community after the episode?
Right now there is no talk to anyone there is no talk about it with anyone
15. Do you think and/or discuss with your spouse/ family member(s) about the death and related events and blame someone/something or feel guilty for the same?
I feel the fault of A Hospital people and S Hospital people also feel the care of both and there is not even care in S Hospital. If you are referring, if you know the condition of the child this is the condition, you are also saying that the operation should be done before one to one and a half months, then you have to do that, in this thing you will have complete ways, if you are sending If not, send it in such a way that in one to one and a half months, they have done all their tests, whereas eco etc. all the tests are here, that girl is finished fighting while fighting with her life. She took S Hospital Referred Paper. I doubt that while on the treatment of (S Hospital), I did not understand even some treatment.
<Blame someone>
I cannot tell you anything right now and I cannot say anything further, right now I do not consider anyone guilty of this incident, it is nothing like this. Yes, she was stressed at the time, which she has to give like the one above, but cannot blame anyone, tension was there, yes tension was at all times even when I was pregnant. Yes my delivery was normal.
16. When did father and mother (if working) return to work after the death of the child?
Right now my husband has joined work from 12th, he is going because he had left the job at that time did not have a job, has joined from 12th to another place.
17. How your and your family's life has changed after the death of the child?
Everything is the same, there is no change.
Knowing cause of death in detail- Autopsy and MITS
18. What was the cause of death of your child and what is your perception about the value of knowing the cause?
The doctor said that Pneumonia had become very dirty and he had the same heart problem.
19. In your view, what more could have been done to know the exact cause of death and the reasons causing the disease?
I don't know
20. Are you aware of any method for identifying the exact cause of death?
<About PM>
Yes, I know a little, I don't know much. That means it shows that because of which, what was the problem, why it was dying, what was the reason, I do not know much more than this, I do not know the process.
21. What are your views about the autopsy/ post-mortem for identifying the cause of death?
<Religious aspect>
It is forbidden in our religion
22. What are your views about collection of the tissue (like done for biopsy to diagnose diseases) and body fluid samples for identifying the cause of death?
Yes it should be
<Religious aspect>
No, so it is not forbidden. Those who bite the part are forbidden for that, yes it can be done according to our religion.
23. How parents and family members can be approached for the tissue and fluid sampling?

<Who should approach>
Yes, if the doctor had said, then there would have been a lot of doctors who were right, they understood well and talked well.
<Whom>
Speaking to my husband, or meaning I used to ask him (husband) to say like this then if he had agreed then something could have happened.
<Explain about MITS>
If you are not explaining to me well, nor can you explain in a similar way, then it can be understood, yes it is possible.
24. In your view, what factors are important for acceptance of the tissue sampling by the parents and family members?
There is no shortage in future planning and treatment, it is important to know
Decision making dynamics
25. In routine day to day operation who takes decision about the major activities (purchases, treatment, spending money) about the family?
Mother-in-law takes, yes father-in-law.
26. During the hospital stay who discussed with the doctors/ nurses to make the decision(s) about treatment and other procedures.
I used to take it. I was at Mother House at that time which is Sangam Vihar. Yes, We were separated from there, actually we have been here for some time now, do not know further whether to live together or we were separated, yes our family was separated, we do not know anything of the present. Only after we have come here and later we will be seen that we have to live together, what is not known, the husband used to walk at that time, although I was the only one who had taken my daughter, they do not know that much. I only know about the treatment.
27. After death of your child, who decided about the cremation and other post-death activities and rituals?
They were taking our father-in-law.
Summarization
28. Now, we are completing the interaction/interview. Would you like to add anything?
There is nothing to say, I just want to take good care of the children there, any parents, because I had not seen anyone who had that oxygen, nor have I gone there (S Hospital) five times, hardly any half-child. It was okay, the rest all expire. There is a deficiency in treatment and there is no care, let us see once right, if any problem is happening, then we are telling people that such a problem is real, in that also those people are irritable, speaking. Do not be afraid to see this again and again, when the child is serious, then the parents will not panic then who will panic.

In-depth Interviews- Parents of deceased children 6

**1.1**	**Primary respondent (relation with the child)**	Mother
**1.2**	**Age of the primary respondent**	Mother-20-25 years
**1.3**	**Other members present**	Grandmother, uncle of child

During interview child mother, grandmother and uncle were present. Father was not at home. The child stayed for 5 days at S Hospital.

2.1 Can you please let us know about the illness and events that led to death of your child?

Uncle: This was born a hole in the heart.

Grand Mother: Birth, there was a hole in the heart.

Mother: It was just shy, and there was a fever one day.

Uncle: I have been telling from the beginning, I was born in the village, then after two or three days, I was crying a lot, it was blue, it was red, then if I kept giving medicine to the little doctor, then be right immediately. Go, when E.S.I. When he reached the doctor, he made an account of the whole, that there is something wrong in his heart, there is a hole in the heart, according to which it is bluish-yellow, he said, "Go and show it in Delhi, etc. Then we brought him to Delhi, and we did the first day in the medical (A Hospital) recruitment. First admitted in the medical, there was no hearing in the medical there, after doing full formality, he gave an estimate of 80 thousand rupees, nothing happened there, then we knew that he gave us the BPL that you have, BPL was not in a hurry we forgot everything in haste or we have We made it simple, then to look back on the other side, we showed in S Hospital, in the new one, from there we showed it to the BPL, nothing even happened there, the same was raped, treated Kept hiring day, etc. There is no thought about the operation.

Grand Mother: Stayed 15 days, meaning slightly corrected, just discharged.

Uncle: What is the name of the doctor, well if his operation is done within 15-20 days, or within a month and a half, then there were chances of survival. Was a boy.

Uncle: The problem was such that the doctors used to say that you must have listened to the hole of the heart, but here you keep that disease and this one is equal to two, these drugs speak something which makes good filter, they were in bad shape, and bad What she does in blood has come in good, the heart was also dry, the drugs were blocked, etc., she was very strong, you will see the reports or not.

Grand Mother: Both the files are stored son all. < S Hospital had taken or referred to himself from medical> did not refer. He used to say (in A Hospital) there is no empty bed, go take it from here.

Hospitalization period

3 Now we would request you to tell us something about the period of hospitalization for your child?

<duration of stay>

Grand Mother: Then it was 4-5 days.

Mother: 5 days

<course of illness>

This was described as pneumonia.

Grand Mother: Drain a lot, so big and so thick needles in your arms and legs, everywhere you mean it was given a full hole, as many children have not done.

Uncle: In Emergency in S Hospital, put the old one in New, and then transferred from there directly to S Hospital itself, number 18 in the ICU ward, 6 days, the sixth day may have ended, while inside dead in Hospital.

4 Could you please describe your overall experience about the hospital care?

<investigations / treatment given>

Uncle: Yes, there were tests, etc.

<cost of care>

Grand Mother: Yes, outdoor medicine means very expensive two to two hundred rupees injections yes they brought it from outside.

Uncle: Yes, if someone used to ask for medicine from outside, then he would write that go get it from outside.
Grand Mother: In the end, they did not get five to five hundred rupees injections, no matter what happened, only in the end when the child was at the end.
5 Could you please describe your experience about communication by the various hospital staffs during hospital stay?
<who all communicated>
Mother: The doctor used to come, the sister like you used to come, meaning the big doctor was just like you used to come.
<frequency>
He used to come on the round once, like the little nurses would not see him.
Uncle: No, the doctors did not say anything.
Grand Mother: I do not tell anything and nothing at all, if I say something, I used to shout that nothing is there, now there is no condition to be saved, so I said you keep doing it, my luck is left or not. Where you had given a date now, you were asking for money, deposit money and then you will give a date, 80 thousand rupees.
<80 thousand in S Hospital?> Uncle: No-no in medical
Grand Mother: In Medical
Grand Mother: In S Hospital, I do not say that nothing will happen, that means there will be no operation, that is, it can be a medicine for cold it cannot be operated, that it will be operated in medical, Apollo was taken there and how many lakhs. Used to ask for money.
Uncle: It was said in Apollo that if you apply BPL, it will not cost six and a half lakh rupees, and if you do normally, then you will get 16-17 lakh rupees.
Grand Mother: Yes, he had taken him to the AP hospital too, he had taken one there.
Uncle: That was also sent by Kejriwal, he wrote that there means a ladies that sits on the name of Barkha he did not see the BPL. There is no idea that it will take years that too you will avoid the submission line.
Grand Mother: It takes two years, no idea, what life will know, and one is here like Palwal, there brother took two time Satya Sai.
Uncle: SS Hospital
Mother: SS Hospital, near Ballabhgarh, it is government.
Uncle: Everything free, everything free of cost for the people living there.
Mother: Heard the name of All India and returned there.
Grand Mother: Hearing the name of All India and returned it, it will not be here.
<How was communication in S Hospital when you stayed for 5 days>
Mother: The correct practice is that sometimes they used to never come, to see that the medicine that is going on is going on, put it on, and put it on Is sitting.
Grand Mother: Yes, you put it on, and you are sitting, and if you ask, keep quiet - shut up otherwise you carry your child, go, take more, take your child at home, we are doctors that you are a doctor, if you are becoming more doctors then take it home.
Uncle: Doctor you are that we are, the practice is such that the government is the one who gets care in private, it is not there or it is 10% -5%. Suppose it makes a difference out of 100%, no matter what. If you go, the message is giving; the doctor is me or you.
Grand Mother: Two times my boy has a fight son, understand this, as if I asked for blood once, in the middle of January, I asked for blood in the month of January. People sat on a drink, they said, "My child, you are sitting on a drink by yourself, and you are calling me an alcoholic, so all of them gathered together and killed my boy one or two hands and then I got an equal boy. He said that do not raise your hand, he is the one who has killed the child and you are killing him, meaning then took blood at three o'clock. My boy's there, then some blood must have been given to him.
Uncle: Not plated but kept empty
Mother: Had kept it, I had offered it a little bit so that the blood came out to check.

Grand Mother: And the blood was taken so much that we kept watching, my boy is still having weakness, understand that he has put a machine and filled it very big polythene, he filled it up completely.
Uncle: No blood taken
<Nurse Behavior>
Mother: Nurses were also like this, we used to say Madam this medicine is over, you said, if you will put it then you will not say, I will not come and you have to go there.
Uncle: It means to say that if there are 20 patients, then there are 2-3 caregivers, 2 nurses and 1 doctor, now how will you look at 20 patients?
Death and post death period
6 Could you please tell us about the death and death declaration of your child?
<who declared death>
Mother: The doctor who sits there is the one who was there; I do not know when the child was dead.
Uncle: The child is over; the child was dead around 5.30-6 in the evening.
Mother: We did not know, the mother told.
Uncle: They did not tell them, they had told their mother.
Mother: At the time when the child did not die, it was just me, then after 5 minutes my mother came and my grandmother came to my husband after that.
Grand Mother: She had gone to take medicine, did not know where she came but did not find any medicine, someone writes such medicine.
Uncle: What did he write Yusuf Sarai all seventeen sites, etc., I got there, but I did not meet, then I went to Rajiv Chowk, Connaught Place, there were chances of meeting there but there was a crowd I said that the child is weak. Anything can happen quickly; he went out and got a call.
7 What was your and your family member reaction to the death of your child?
Uncle: Direct is not told, they were told (child mother) when they have brought the dead child from there, brought it here, brought it to home.
<you didn't tell about death of the child to mother>
We said that we are taking others to the hospital, they were broken there already, they were shattered, they were alone, and they were not alone. Then I have transferred from here, there is a hospital near my house, Majidia has to take it there, I said a lie and got all the people in the auto and brought all the people.
Aunty: The maternal grandmother, who was the grandmother of the boy, was not.
Uncle: I took a call from someone from the neighbourhood. The child's grandmother was told by the doctor that there is no child in the hospital. First granny had to know, the doctor said that there is nothing in the child anymore.
(Uncle showing us the picture of the child)
Uncle: This child was someone tells us that there is a problem.
Grand Mother: Photo will be of Lalla (child), all buried in it, nothing is kept son, kept so many sack-filled clothes, all the paper-wagons who kept everything as they said In the cemetery, where the soil is buried, everyone said that after putting the cloth on the bury, they said that there are two pieces of paper, filled with sacks, there were many files, some are stored there, nothing is kept at home. , There is no sign of it, that the child (father) has become very mad.
Uncle: No sign kept.
Grand Mother: Now his (child father) knot is coming out here, he is saying what will happen to our brother, when the child is gone; we will understand the son and give.
8 How was your experience about the procedure after death till handover of the body to you?
Uncle: Yes, I gave it a slip, yes it was done.
<Time taken>
He must have written the time etc. according to me. They took a lot of time to make the paper, how many are being made of the paper. I told you that if you do 1 man on 20 members, it takes time, oh yes; you are going to be made, at least after death. After half an hour we have been able to leave, after half an hour we can leave at 6-7 pm.
Grand Mother: Could leave at 6-7 pm, and then brought it home.

Uncle: He kept all the papers in formality, gave a slip so much as to sign me.

9 Who all from your family and community/neighbourhood were present at the hospital and how they supported you during this period?

Grand Mother: Yes, yes, there were four people, the child's mother, father, uncle and grandmother.

Aunty: Four people.

Uncle: The rest and those who were his patients were giving us comfort, they were explaining to us, they were also supporting me in lying that no-no is right, your babu (child) doctor has transferred, has referred the other. Do not take too much tension.

10 What all rituals and procedures did you follow after death of your child?

Grand Mother: Yes, the graveyard does not open at night, and then it was found here at 10–11 in the morning. Yes-yes, bathed means from my law, it is right here among the Muslims, all should work according to the law. Like a bath, then a bath.

Uncle: The child body remained kept throughout the night, such children in relatives do not wait for the soil, should not be taken as soon as possible, the time was lost, we do not have it, there is a time table, Then we started before 2 o'clock in the morning, before the namaz, the elder brother just came from the village and did not see any wait for anyone. Woke up like this throughout the night, bathed in the morning, washed it, then brought the shroud, here is the shop, did everything that the formality belongs to us, fixed it in the shroud and took it back, we said it in the morning. At 6 o'clock to dig, the tomb was ready by 11 o'clock in the morning; all the prisoners were again buried. There were many of his entire family, many from the neighbourhood; at least 30-40 were closed.

Grand Mother: Enough was enough, enough was enough?

Uncle: Grandpa and I bathed the child.

Grand Mother: Dad was crying, he was very mad, he was begging and there, he was falling on the feet of the doctors too when the condition worsened, but where do the doctors listen to anyone.

<rituals after death>

Grand Mother: Yes, it was imparted, like a child would be given milk there for 40 days, like someone who is fascinated, poor, that means a packet of biscuits that a person or child eats, that is given in a little milk. If I used to eat biscuits or papes, then they come in the morning.

Uncle: Which means the poor are more.

Grand Mother: Yes, yes, we give it for 40 days, then if it means a little bit of scope, then you divide the child by tearing.

11 How did you, your spouse (wife/husband) and immediate family members cope with the difficult phase?

<So how the parents have emerged from that thing>

Grand Mother: Understand, they are comforting, they are grandparents at home, then they think that they will give more sons, as long as they were in luck, they stayed with you and the luck If I was not gone, the boy is so mad that my Babu is sitting there, calling Babu, this is Babu, he will go out of the house so loudly, that seeing him, the boys cry out We will keep on thinking, Lalla will give more.

Uncle: Give comfort.

12 Is/was there any change in the interpersonal relationships between you and your spouse and other family members after the event?

Mother: No, no, nothing.

Uncle: No, no tension is there.

13 How did the family members support you and your wife during the difficult phase?

14 What were the reactions/ counselling and suggestions given by the relatives/neighbours/community after the episode?

Uncle: Yes, yes, outsiders also understand.

Grand Mother: Yes, yes, even outside people understand, they say that the son does not go crazy like this, look great, then what do they do, our girl's 22-year-old accident is over, How is she living, now this is comforting here, everyone does not talk at all in front of her child, that she will miss it.

15 Do you think and/or discuss with your spouse/ family member(s) about the death and related events and blame someone/something or feel guilty for the same?

Uncle: No, it is not so, yes it is that S Hospital can hold those responsible, so much that they can do anything when they were fully aware that they wanted to talk about everything.

Grand Mother: No one is responsible.

Grand Mother: That means if they could have wanted, then they could have said that you can give us half 10-20 thousand rupees and bring your children, we can do the operation, they could, but they did not. Did not take it in there in the manner, used to say, bring - bring money, it will be operated, bring - bring, you will not be depositing money, go from here

Uncle: You guys will have to deposit money, when admitted to medical for the first time, then I saw a week, seven or eight days, then they saw that they could not deposit the money, then they used to pretend that they referenced it. Here for (S Hospital)

Grand Mother: Then the meaning started to get more, then I used to run away from here, kept it for a while - after two hours, then from the Medical (A Hospital) in S Hospital that the beds are not empty here, yes they used to keep the same hours and two hours.

Uncle: Now, if anything happens, he used to run away from medical, medical to S Hospital he kept 1st time in medical then used to refer to S Hospital.

Mother: Three children, four children each on a bed.

Grand Mother: Looked a little and used to say that now take S Hospital, yes, it was said that there is nothing to be done here, it is the cold pneumonia, its medicine, the operation will be in medical, nothing can happen, three on each bed -Three, four-four children, yes, four-in-one, five-in-five children in S Hospital, all kinds of good and bad living in it, even if there is no such disease, then the disease will spread.

Uncle: Cleanliness is good in medical, but there is no cleanliness in S Hospital, neither in toilet, nor in toilet

Grand Mother: Yes, so much dirty in the toilet, there is too much in S Hospital

Uncle: In the ICU, cockroach used to be lying around the bed, the boards keep going inside.

16 When did father and mother (if working) return to work after the death of the child?

Grand Mother: At work going nowhere, they have not been able to work for 2-3 years, do sewing, the work is not going on at all, meaning cutting is not done on that day, public knows, if you know cutting Meaning there is no full teller, no cutting work is done Noida etc. has run, the company is not here at all

Mother: Not going now.

Uncle: not a Full Taylor.

17 How you and your family's life have changed after the death of the child?

Uncle: Nothing special, as before you were still living, you are still living, yes there is a bit of grief that at least earlier there was a difference in it, earlier it was to play songs, TV. Gone, that is nothing, no feeling comes now, see the sorrow.

Grand Mother: We are very sad, my condition becomes like this.

Uncle: No, not at all, their husband did not have any bad habit after this accident. This is as bad as it was after, whatever was there, it is improving gradually, I tell myself that I have suffered a big shock I could not collect enough money to get my child treated.

Knowing cause of death in detail- Autopsy and MITS

18 What was the cause of death of your child and what is your perception about the value of knowing the cause?

Mother: Nothing directly said that the child is no longer there, its shock has gone, they gave some reason why the child is finished.

Uncle: Didn't say anything.

Mother: Then after this I was sent out, my mother explained that there is no strike, then started saying, try and see if there is a strike, then they tried for 5 minutes, did not in front of me, tell the mother She had been, then told in front of him, then did not tell anything, just phoned him (child chacha) and he came.

<Perception about the value of knowing the cause>

Uncle: What is the cause of death? This is just the child was ill and in exactly the same condition, when the doctor admitted to medical (A Hospital) for the first time, then this operation is as soon as possible before two and half months - If it is first, then it is fine, otherwise there is no chance after that, even if you do the operation or not.
Grand Mother: Now whenever we had an operation guarantee, there was nothing
19 In your view, what more could have been done to know the exact cause of death and the reasons causing the disease?
20 Are you aware of any method for identifying the exact cause of death?
Uncle: Yes, just like there is a death, accident, etc., hanged and dies, then post-mortem takes place, it becomes known, a government case.
21 What are your views about the autopsy/ post-mortem for identifying the cause of death?
Uncle: They comfort their hearts that there was a disease, heart attack, etc., that heart attack came to that person, it happened.
Grand Mother: Yes it is done - it is done, a person takes peace in his heart.
<ritual aspect>
Uncle: It is not justified, we do not want to get any death done here, it is another matter that there is a long case, if the police case is there, then forcibly.
Grand Mother: There is no such thing as dead body.
22 What are your views about collection of the tissue (like done for biopsy to diagnose diseases) and body fluid samples for identifying the cause of death?
Grand Mother: Yes, we would have got it done, it is a little bad, it is a good thing, it is good, it is not forbidden for this test, that when our child is gone, it will be so much more that it will be known to the heart. It will be comforting for what reason my child has gone
Uncle: Yes, it would have confirmed it, yes it would have cleared us what caused the death
<religious aspect>
Grand Mother: There is no prohibition of religion in this.
Uncle: There is no prohibition of religion in it.
23 How parents and family members can be approached for the tissue and fluid sampling?
Uncle: It won't happen without parental permission
<whom should they approach>
Which man would be in the responsible of a doctor like I
<who should approach>
Main doctor should tell him
<mode of explaining MITS>
They say the same (main doctor) in their mouths that we will take out every part with some injection and after testing it will tell what has caused your child's death
<when approach>
After giving yourself some comfort, you can come to the side and say that if you explain such a thing about sudden death, you will not understand it soon.
24 In your view, what factors are important for acceptance of the tissue sampling by the parents and family members?
Uncle: Now we have come to know that this is new technology that we will be the reason whether the doctor has died or the child has died due to illness.
Decision making dynamics
25 In routine day to day operation who takes decision about the major activities (purchases, treatment, spending money) about the family?
Mother: They used to go together (brother in law).
<treatment>
Uncle: I take decision
Grandmother: S Hospital used to take it anywhere

26 During the hospital stay who discussed with the doctors/ nurses to make the decision(s) about treatment and other procedures

Grand Mother: So they (child Uncle) were doing all this work here only.

Uncle: I have been the main.

27 After death of your child, who decided about the cremation and other post-death activities and rituals?

Uncle: Those who were educated there are maulvi, etc., who tell all of the child that to do this, that is what the father allows, allows and the first three fists are the only father, then the grandfather Then add any.

Grand Mother: Those who are educated tell them as they are

Uncle: No-no never consulted anyone.

28 Now, we are completing the interaction/interview. Would you like to add anything?

Uncle: Well, I have my own walk in S Hospital; I have just had a baby girl on August 15, the last girl, so copper-T is putting copper-T on everyone's side of their choice

Grand Mother: Yes, put it on everyone, now it is hurting, it is causing trouble

Uncle: Ever since he got copper-T, the pain, swelling has increased here, there is a normal check-up or not, all is right, just leave the medicine and food, not taking any combinations, we want That the two copper-T got out, why the sagging aunt in her family had got copper-T, she was in a very bad condition, remained 25%, after that the copper-T came out, she got it right and the same happened to her aunt's girl. Copper-T was in place, the same happened 25-30% of their chances, then the Copper-T came out, they were correct, We have done this for two cases in his family, we have also gone to remove the copper-T and postponed it and then they are not coming out and then should not be applied without permission, he has said that after getting the child, the copper-T will look like yours, so he said I will not do it with my will, my husband is outside and my mother-in-law has to ask him that yes we will ask him later, You sign, forcibly sign, he signed in fear that he will threaten many children there, etc. I will do so, that is, the nurses will kill one of the ladies just like my wife. Don't know why the wrong behaviour means yes to copper-T.

Grand Mother: Yes, they start speaking upside down, they die, and they turn a lot.

Grand Mother: What would you like to ask, now that they have a future ahead?

Uncle: Just give me the opinion that how the copper-T will come out, my wife is suffering a lot.

In-depth Interview- Parents of Deceased Child 7

1.1	**Primary respondent (relation with the child)**	Father, Mother
1.2	**Age of the primary respondent**	Mother-25-30 years<br>Father-30-35 years
1.3	**Other members present during the interaction**	None

2. Events that led to death of the child

2. Can you please let us know about the illness and events that led to death of your child?

Father - Mam actually happened, the girl was at home, and all three of us who were born have also been at home, all the children have been at home, this happened once we had all the children of the three children. The thing had become of MM dispensary, from OPD, he took it there and showed it to the elder boy, it is time, just after bringing home, delivery was done, when he took the middle girl, he started saying that S hospital has to go here is It is a month's time when she was also brought home, the younger boy did not take any time for the boy, in fact it happened that she comes to our house as a nurse, so do not take her away when the two made up their mind So do not take it, you did not carry it, tried to carry it this time as well. Asked not to speak to me, left well, what happened after 10-15 days, a little bit came, this was the fourth child After coming and coming I said to take the MM dispensary, when I took the MM dispensary, then the wife was taken. She said that she is telling the hole in her heart I said how can it be done, such a niche was set, and there was a hole in my heart, I could have said, these MM dispensary doctors had told, I said more experience will I have said, I can transfer and then transfer S hospital has happened, so I said go get eco wager, then we will see, if eco etc will be done in front, then I saw that there is a hole, I asked the doctor, I told him that he was telling about 3-4mm eyelet infection, everything was telling. After treatment, the treatment continued and 2-3 times were admitted. Blood also happened, everything happened, just look at what we say is ours, so let's say we have an unknown expression, something that happens may be a little bit from the present to the parents, some may be such an education, some may be careless and the doctor people also have to do a little duty for 24 hours the same night, some people feel tired at some point in it, who went three times, the girl was completely come. For the fourth time, if the old doctors were not found, then the girl is a bit careless, the old doctors were not found in S hospital, there was such a good madam by the name of Deepali, I talked to all the people, but I am not good I thought, because she never used to come, I used to think that laughing at a crying person is behind the big success and anytime the big men who feed the baby, they would have fed very well even if they did not have the desire. She used to play, she used to speak and recognize everyone it was just that, just a little mistake has been made from them and for that I will say that they should pay attention to what they have done from their side.

Hospitalization period

3. Now we would request you to tell us something about the period of hospitalization for your child?

Father - last 6 or 7 days admit and or who has gone, has been taken from here in the evening on 20th date and expires around 3 and 2 pm, expired on the 20th of the night, yes it is a The day before it happened, it was 4-5 days, it was admitted, the treatment was done and then it was going to be discharged, this was the fourth time when she had gone to admit, she was removed from her in the emergency and went to the ward and negligence in the ward. Happened It is not that there is no negligence, at last he felt as if he had too much cold and that there was a hole in the heart, then the infection was spread by him. The eyeball catches pneumonia very quickly and becomes double. Sometimes, in this round, we have seen and went to see it and it was right after taking it. Husband, quote, everything happened, after that I came and had three children at home. Wife was telling that often on the night when her first two three girls turned up once, for the second time, the tightness of such hand went on again She went. The wife was in the hospital with the rest and she was alone, there was a pump on the ground, still there is a shovelling, she is walking, see what happens, what does not happen. The doctor is nothing, actually the one who was about to leave is gone Now the dead body is left, the doctor said, yes this is the feeling, I see two minutes and

you press the pump, I tried for two minutes from my side, every parent tries from their side and often this If it happened, then after 2 minutes, I removed the pump after five minutes and say, take it away. In the last case, Madam, I did not ask anything and at that time the wife would be crying, I could not ask it and I am feeling sir that the wife was saying something, something has definitely happened.

4. Could you please describe your overall experience about the hospital care?

Mother - Doctor, it was right 15 days before it started, it became normal at home, the child was not able to feed, so this little boy fed him while feeding him, feed him comfortably, I told him not to feed my son. , So he said that he is a very good person himself, so I said let's get medicine, I can't leave Babu like this, I went to MM dispensary, after going there, Doctor Sahab said what happened to this little baby, I said yes he has a niche I started to check, I said that it is not bad That boy is bad, I said, so he said that the boy is not bad, this girl is ill, why did I say that there is a hole in his heart, I said how such doctor, we said that we are referring to S hospital and want to eco Is, do not be negligent, we were taken in emergency, went there and asked in emergency, if not such great doctors said that they sit in OPD, they do not sit in emergency. You show me in it, then after that I made a slip in it, then I got everything done in eco, after getting the eco done, Dr. Saheb said that you will come to Friday, that day the heart doctors will come there at 2 o'clock. It was a good girl for us to know that the doctor knew that now, when the doctor said that the child will have to be admitted, the health of the girl is very bad. One day, I was feeling ill while doing home, I was also feeling ill, I used to drink less milk and wept blue. It was after that, she was admitted in the morning, got discharged in the evening, the doctor said in the morning the child is right in the evening I had to leave at four o'clock, I was admitted for the first time, I did not know what the meaning of recruitment is. Evening is a holiday, leave at four in the evening. Then said nothing happened to bring again, for the second time, in the emergency, he said that the child is very ill, the cold had increased and the light puffed up, that day increased as if he had to need a medicine and his medication was not there. Was getting a heart CF was not available, wrote tablet, said four parts, one part to be fed and the fourth part, then I started to drink by myself, the other one used to drink medicine like urine. And his weight also started increasing, slowly the weight increased, the doctor said, son, now there is no need to panic, heart holes are being filled and there is no such problem. After that it was x-ray for the second time. The old people and eco were the old ones who had done it before, then the field doctors had come, they were older, they said, the child is right, he went for the third time he had such a mild cough, so let's not take care of this heart Doctor sir, Lenny will have to pay the child may die So I was scared, but I went again, it was ten days in the third time, the doctors just said you take it, there is no cure for it, I did not know at that time I did not know whether there is a cure or not, Dr. Saheb did not tell, I had not seen this thing, so I did not know that he had said it repeatedly. He is senior, saying that the child will die again and again. Say the same thing then we said, Sir, can you tell me something? People say that doctors are in the form of God, they are not saying that we have come here so how can we refer, then what should I say, then I said okay, I am looking, blood and blood started to rise, but there is a lack of people There used to be those who used to sit at night, three out of 10 doctors were right, but whatever there were 7 or 5 who were like big doctors come in the field, they are telling us something. So let's go and sit there and scold him so badly, after scolding, we used to say that we are for the emergency child, it is not for your child who was going to die more than the child, he used to sleep in his own room. Used to get stuck, when the doctors of the field are going to speak anything, there is no medicine, then tell me, we do not have it, so if we do not bring medicine, then we could have explained it that we were lacking. Did not bring medicine and Dr. Saheb used to speak when he used to come to the field, if you do not give this medicine, if you do not get the needle, then you will not bring any matter from outside, then you will see it and will talk again later. He used to suppress the same thing he would not let us speak further, when we used to say this to Doctor Saheb, Doctor Sahib used to say why you have not written problems. Mother should know, Dr. Saheb used to say that when senior doctors come to the field, more comes Dadagiri, he will die one by one, who used to speak like that, senior doctors used to talk well "son like that No, "He used to talk very well about medicine, he was a senior who used to sit at night. He was very good. He kept me for the third time for four days." Here we were two, there is a front street, there were also a child, we were two, my daughter was weight, 4 kg, 24 grams said son weight is right, the steam used to go and the oxygen did not

reverse by applying steam. They used to get some rest again, they never got sick this girl was such that many people used to say that the child is right, the operation will be done in the sixth, but some people were right in it. Some were wrong, they used to shut our mouths in it and the children of the house are small, so we had to think about everything, carelessness is more in it, the girl finished the fourth time, yes, the fourth time I went, the girl was lightened at 11 o'clock. I had a cough, so I was afraid, took it and after going there, I had milk, after feeding, it was just that, the doctor was sitting feeding ladies girl Bid once you can see my report started saying that the treatment is going on from this, I showed all the reports of all the recruitment papers. , Then bid this girl will have to admit, she should not steam or put a bottle, just put so much needle and then reach there, she said, why are you bringing this sick so often, I said, I am in my hand, started speaking He will remain sick like this, he will die like this, he said the same thing again and again, I did not feel bad after that the doctors who sit there for steam from there do not steam in the emergency Earlier, we used to give a 15 to a 20 in a 5 minutes on a 10 minutes, but these doctors who were sitting that day, the ladies who were there, did not say anything, I was very stubborn, I met an old doctor. I started crying with them, then why are you crying, I said doctor, its health is a little worse, then I am saying why I said that green is making green latrine And the eyes started to do like this, when I was about to die, I did not die when I was feeling very bad, it was good enough, the doctor gave the medicine to steam, the girl turned around, started playing after turning, she had some old patients, she came. They are saying, 'Agni', I said yes, everyone spoke with fire, after that, the girl played her husband, then after that she started having strange eyes at 11 o'clock in the night, then I went away. If you say something about sleeping, when you go there, no one would meet, if you go again, we used to listen to data, when the health of the child was very bad, sit and sleep. What is the matter, doctor sir, he is speaking again and again, why you are getting scared that oxygen is coming out, is there a dare that the child will die, I said doctor sir, you can see once. I used to see one thing again and again, it did not work well, the life had already passed, the steam was right to turn back then wrote the medicine. I went very hard to get steam medicine and steam medicine As long as the girl was right for the part, I did not let it steam, I did not get steam, the child's heart would be stressed, she did not die much of try, she would not die if she let a steam Heart-born children do not increase their weight when they die, when there is a problem or there is an allergy inside it, even get everything done for a date, do not understand that we are unaware you are a doctor, you can see that report. Do not ignore that thing, let us know what is the disease in it, it can run medicine, otherwise it is not possible to answer the child Then in the morning after that, saying before the junior's arrival, remove the body very quickly, it used to be that no one with my child will die if the child ends up with him at 4 o'clock at 12 o'clock as the field is known That the doctor is going to come, after 8 o'clock, before clearing the body, he used to leave the hurry, take the body away. , When we have luck, let's have three, we had fourth, we did not want it when time has gone, it is not revealed that when the one above was given to us, we did not take it, it would not have been a sin if we had killed it, after taking such a disease. It started happening just as the doctor said, but for the fourth time he was careless.
<about investigation>
As the doctor took the blood of something, he used to give it to us. He used to tell us to stay safe. When we used to ask, he would tell me to be told, one hour and two hours he will be spoiled. Were, when we used to speak 2-3 times, the great doctors used to come on the field, when we used to say that it was your fault, we used to get bogged down, then we used to be silent. Tell us if there is a shortage in the morning before we were told, people used to do field work, which used to write what happened in the file, they would not let us speak.
<about cost>
expenses, what will I lie to you doctor sir, I am a poor man, his (husband) is also suffering from kidney, medicine is going on, I have three children, there were some of my 15 thousand yes medicines, some were written outside. Were not found in S hospital injection medicine was not available even in it, which was not available from outside, so there was a girl in it, she was a girl, she was right when I used to cry, do not cry, she used to help me, so sister should ask for anything. I used to ask for helplessness, but I did not feel bad about anything, it was just that the girl should be right.
<about behavior of HCP>

It was strange, it was not good to scold, the nurse medicine that people who are learning like this were a bit right, like anybody is a little proud, ask lovingly, when you can scold you can explain but only one doctor was right in that Deepali doctor. The child is in the ward 20 in the H block.

5. Could you please describe your experience about communication by the various hospital staffs during hospital stay?

<about communication>

No, they used to read on their own, they used to understand on their own, they did not tell that the medicine is running, they were also telling some such medicine that Deepali is running, if that medicine is used then it will stop.

Death and post death period

6. Could you please tell us about the death and death declaration of your child?

Doctor sir, like I said, after calling in ICU said that your mind is bad, mind has gone, if you do not have courage inside me, will you see the child? Why is not it, when I was fumbling, its leg was cold, so I thought fever would be going up, I used to measure with a thermometer in my head He never had a fever, then the doctor said, fever came, do the bandage, I started bandaging, I started jerking the bandage thrice, I could not try to turn three times, after that, she was tearing eyes and her eyes As you sit, neither was you agile, but you used to say, Mama play His life is coming out, these people did not come, I did not know, I was sitting with my oxygen, after sitting, I started crying a little more, the girl is not moving, which is not like the baby is not automatic. She is afraid, I went to Deepali, I was not a doctor, but I forgot her name in it, she was also right, I said, doctor, you see, see if this is a child, I have not said it means my child. Is in front what not? Inside this, I could not understand the gestures between them for 5 minutes. After that, bring the pump that is pumped and tear the mouth and put it in the drain, remove me then after that one is not saying now. Do not put the hose out, said it, put the steam, I made the boy sit with the steam, now the doctor goes out of his life. So the child shows life in their eyes, when it is their time, then they remove it, so do I, after bringing the needle at 3 o'clock in the night, I said someone with you, I did not bid, so I went The person who brought the medicine to steam her running, said after 2 hours, apply it at 5 o'clock, I said, "Okay, then call your family, I said that I am very sick, then you are crying, then you will What to call, I called her husband, she said this phone, what is the matter, I said, doctor, keep calling After that, after doing a full hand shake green latrine, Dr. Saheb came saying that he felt steamy. I got dialled 2-3 times, did not hear anything, and did not hear anything. Get a little hand started shaking, after that, doctor said, now it is good, then I started doing like this again, I kept running and crying, saying what happened then the child will not die How much should I say, do not bring here, do not bring, like it was finished when you put the pump on, it was over 10 minutes after 4 o'clock, then they do not speak a little bit, half of the children come out of their mouths as they come Started as soon as the drain was poured out, he often did the same thing, he will do the same thing for 3 days and 4 days, a child will go very well. Is, I bid you guys.

<cause of death explained>

Did not tell why Mara was killed, just said that there was a hole in the heart, it was said that there was a hole in the heart, I had gone out.

Father- It did not tell anything that there was a hole in the heart, so the child was killed, all these things were talked about; when the child is finished, then after that, take away the dead body. , No Meaning

7. What was your and your family member reaction to the death of your child?

Mother - crying there when it was found out that the baby is not there

<did someone try to console you>

The son started thinking that he was destined, the guard was right, he was also poor, the doctor was also there, in that he gave me Deepali (doctor), she did not come, so I was afraid sometimes she loved him (m) . Very good nature, no one is angry with anyone, no matter how much, someone is personal, no matter what anyone means to me, we do not mean to him, we do not mean to work. Whenever she meets, she works with one mind with a passion (father). But this time, the doctor who had done the report, he was getting scared about the eco for the second time in the same way, the doctor said, if someone is with you, then I did not bid, then say good you come once again. Then for the second time, when they used to do it

for the third time, they used to come round as a round, then kept it and had a child, then call him below and call him too, then do not tell anything after that, all these tests have been done in S hospital new building. In, the report that went to the Emergency, if they had seen the doctors, they could have saved it all. They ignored the girl, did not see the report, and they were asking for the third time that the recruitment papers were there, they suppressed there, no old doctor had met me or met only one and Deepali Doctor came in the morning.

8. How was your experience about the procedure after death till handover of the body to you?

<about documentation>

Paper was signed on the father (father). I have (mother) so big to tear it.

<about cost>

no money nothing (father).

<time taken>

It took 10-15 minutes to do the paper work, and nothing was done (Father).

9. Who all from your family and community/neighbourhood were present at the hospital and how they supported you during this period?

Whoever did the help with our neighbours and the doctors in the hospital and some of the people who were with me went to see a little listening to the bathroom to talk.

<what type support given>

Neighbours also made money with the help of children and even after washing their cry, it remains to be helped in everything.

Post death procedure and coping mechanism

10. What all rituals and procedures did you follow after death of your child?

Father- 4 o'clock in the morning, the girl was finished, so I waited on there and called someone from the house, and had to bring the wife also, I had to bring the child too, then went auto from here, I have my own auto here, I have them Had called and they had gone there, it was at least 6 o'clock in the morning to take the whole thing from there, in the morning, we left from 6 o'clock in the morning.

Yes, a lot of people came from relation, father etc. My mother had not just come from my heart, my mother was finished with cancer, my father is mad and my younger brother village once asked about poverty, then he went out and all my in-laws came from his mother-in-law, his father-in-law had come, mother-in-law had come. He was in a relationship (mother).

The mother-child has been recording when she was 6 days old; it was over when body came to have a photo. We have 3 children, we will be patient, after only one day, after how many days the mother is there, the mother is so much, the mother becomes mad for the child, and the disease remains something else.

Father- After coming home, all the relatives came and just took the dead body, then the body was washed and everything was done, then they were all dead and then they took the dead body to the cemetery in the hill. I want to say that the head that happened to us should not be with anyone, I think that the doctor who is coming to us is the future. It is his duty to see someone and tell someone that his religion is from one side. The doctor is the form of God is actually right, Because if we do not do it all over the place, then who will consider them, they say that no one has seen God, yet they believe that the educated people believe in them, they believe in them, otherwise I want to say that when we believe in them If they do, then they will not trust us until they follow up.

<who prepared body>

We were served neighbourhood they did the work, they did all the work, there is a little 10-minute road nearby, the forest is all dead body goes there; yes we did bury the baby's soil. We had gone to at least 60 people, all the people had gone, friends, how many people could not know because the relation was very big after working in the field, how many people started saying, I did not know, I said that was to be He is done.

11. How did you, your spouse (wife/husband) and immediate family members cope with the difficult phase?

Father - Look to get out of it, ma'am actually comes out the same thing, as much as what you think will feed in the mind, if you have to do something new to get it out of the mind, just work a little here and there, To show that I have not read till 12V in the field, yet I have to do all the work, I had to explain to them that a lot of things cannot be done, what is possible is possible which is impossible. is impossible
I have to manage myself, I do field work, the field worker will sit in the house with the people, so actually it will be as if I am reading this by taking the paper, then the focus will be on that I am reading the paper. I am a worker in the field, goes to the field and talks with the people, a little mind is removed.
Mother - Now everyone started saying that your child was small, the children of the heart do not live, now I did not know that much, who said that I listen to everyone, I cry in the house, neighbourhood explained, do not cry. On top of that, her (her) kidney is bad, she also thinks that she has gone whoever is 4 days, 5 days, 5 months, whichever day she is, now I think that she should be treated, so I think where will the money come from on rent? I live with 3 children, in that the first report showed kidney failure Now if I go, I say the stones are bad and the medicine is given a little comfort, I keep on worrying a lot, I think I will not be able to walk anywhere, I will go with 3 children and no one is tight to the in-laws and no one is in-laws' tight Will feed 3 children.
12. Is/was there any change in the interpersonal relationships between you and your spouse and other family members after the event?
No change is like before it is still there, not like it was before, it is still there, we talk in the same way, and there is already a little mind. What I am today was not there before whatever happens to get education is very good. I never really understood what all this happens, because I am a working person living in a village, he does not know what he is working in agriculture, he knows that it takes crops, crops are harvested in the house. He does not know what things are planted in the crop, how chemical is added to it when the crop is ready.
13. How did the family members support you and your wife during the difficult phase?

14. What were the reactions/ counselling and suggestions given by relatives/neighbours/ community after the episode?
Neighbours just say what has gone to sleep, if this is what it is, get out of your mind, do some good things, we have 3 children, think of their upbringing. The suggestion was to find out the cause of death, brother, there was a hole in the heart, because of that, it happened to us, even the actuarial doctor suggested something and neither did it tell us that yes, the disease was caused and that it went to the doctor. Did not happen And there is no problem even with the senior, we said, let us have a hole in my heart, brother, now we have to have patience because the status of living and dying is not the same, one day everyone is coming and everyone has to go. The way and the same path will be the end of everything.
15. Do you think and/or discuss with your spouse/ family member(s) about the death and related events and blame someone/something or feel guilty for the same?
No, I don't believe anyone guilty, but actually this is what I will say, on whom I tell you now, the doctor believes the people to be God, they should not believe in superstition, that is all I would like to say, I was patient Took how many people have to have patience, they have a big responsibility, they should understand their responsibility in a good way. There was a doubt in the treatment that you did not give us something that if you do this then your child can do it, and if you are asking for suggestions like this, are we also asked in the meeting, then we can say everything that we cannot In fact, anyone is getting benefit of this thing, in fact, it is the reason that they should benefit and why they should benefit Even then, he needs benefit. Our child does not want his child to die and I want that when the doctors are giving us an example of something, we are talking about something, we are talking about something. We do the same thing. And we have to listen to that.
16. When did father and mother (if working) return to work after the death of the child?
I was at least a week, that time the work was completely closed, now I have to be busy because even before you told me I do 2 working one part time one full time, full time is my clothing business part time my networking marketing work.

17. How you and your family's life have changed after the death of the child?
In life, everything goes on above, I think that for the rest of my children, as long as I am alive, my life will do something for my child, because I want to change my future and give them a good credit, I So today it does not explain that I give very good advice, I understand that I give as much knowledge and as you guys, I get very good knowledge and very good talks and this is what I want to put in my child. I am No matter how much anger someone says to someone, even if you call them dirty, you call it good and fold your hands, brother, you will grow up and we will remain small.
After that incident you have to take more care of the children (father), care has to be taken when there is no money, then you have to think that you have to pay rent. If you do not understand the poor then you sit down silently (mother).
<about substance abuse> No, not all of these, nothing from before, no drinking water from starting nothing, nor even today, I add my hands to all these things, I also tell the people who come, nothing is gained from their bodies. Drugs are nothing but waste. (Father)
<Plan for future pregnancy> No, I don't think of all this, I have a lot, I did not want to have children, for this I had tried lakhs, when I gave the above, then when I found it very late, I came to know after 5 months (mother).
Knowing cause of death in detail- Autopsy and MITS
18. What was the cause of death of your child and what is your perception about the value of knowing the cause?
No don't know about it (f)
19. in your view, what more could have been done to know the exact cause of death and the reasons causing the disease?
If our post is not done, how would we know (m)
20. Are you aware of any method for identifying the exact cause of death?
<about post mortem>
That is why, what was lacking, what caused the child to become a disease. It is our suggestion that when we could not get any advice in life, then after death, what should we do to the soil which is in our destiny and sat down thinking.
Father - Actually this happens when there is no faith inside your soul inside your mind, then it is useless to accuse someone if there is no faith in it, then if we ourselves are thieves then why make the other a thief, if we are honest another thief If brother is there for a minute, then the next may be the thief, we are thieves ourselves, so what does it mean to be the thief of another?
It may be the fault of both people, it can happen to us as well, it may not have happened to us, we did not think of any such thing. I keep saying again and again that the doctor will have to respect a little, attention has to be paid.
21. What are your views about the autopsy/ post-mortem for identifying the cause of death?
Father - <necessity>
It is absolutely necessary to know the cause of death, but some people are also unaware of their lives, what happens is often some people are unaware of their lives because the parents do not pay attention, secondly the parents also pay attention, but the children's count has spread so much In today's time, 12 years 15 years and 8 years 10 years old child starts drinking water, I do not think that if India is such a situation, then the future will be right for us because nowadays there is so much pollution, the biggest is poison. That is And how does pollution happen to our people, it happens to our people, some people pay attention to good things, do not do good things, some say it is crazy, you should do as much dirt as possible and put it on the road Is, they burn it, some people ride the bike as if they think that there is a sprinkling of the medicine of the mosquito, nor does the smoke come out of the bike, then we are the only reason for the disease of Surrey pollution They put silence on the government The government is not doing this, the government is not doing this, I say that there are four sons in a house, one father is unable to explain four sons, then how will the government run such a big world. It is important to know the reason that if you are satisfied with your soul from within, to know the reason everyone will have to be united and work to stop all these things.

Little is known about the big people, it happens that if something has happened within it or if they have committed suicide, then what is the reason, what is the motive, in all these cases, they do post mortem and if I am now 3-4 days ago I am talking about this shooting range. While coming towards Tughlakabad, there is a red well there. There was a person talking about water, he was a boy, he was a boy of 20-22 years, both arms were severed. Eyes were taken out, is there so much atrocity inside India, such a huge situation, post mortem of this thing It is known that brother, why did he have any enmity, there is a motive too, it is good to fight the case and also to work the case, now we press on the doctor in the most useless things Not a good thing but yes actually I will say again and again that the thing that we have faced should not be done by other people.
<time needed>
As time goes on, a dead body is scattered in the entire way, by accident, it is quick to win it, there is nothing in it, there is some dead body which takes two hours and a half hours for 18-19 years. Yes, now it has become very easy and gone in 2 hours, previously used for 2-2 days, for post mortem.
<about disfigurement>
Yes, the body gets spoiled which is a useful thing inside, like the kidneys, kidneys and everything is removed, actually there are two people in my relation who are safe and they say that there are many things which are good It is taken out and wrapped in that amount of time. It takes a lot of time to recreate what is once made, I think it is very good post mortem, people think it is bad that after dying, this body is burnt or buried in the soil. If there is something inside me that is removed and put in the other, if it fits well, then the life of the person can be saved, the person of his house is very good, then I am very good I think Some people do not know the reason for what happens, often they focus on something we do not do and what we focus on, they consider that thing wrong.
<religious aspect>
No, it is not that what is wrong with us is wrong, what is right is right.
22. What are your views about collection of the tissue (like done for biopsy to diagnose diseases) and body fluid samples for identifying the cause of death?
Father - Day by day the world is technology, I can understand that people talking to you started coming to my mind also, I can understand that before the body is cut, it was cut and then it is checked. Now this thing is a new technology, I like it so much that the body can be safe and technical check is done and it also becomes a good report. Why did it happen for what purpose, India should progress day by day, this is our morning wish, to progress very well in the coming future, and the country which is outside country makes progress, our India does not progress.
23. How parents and family members can be approached for the tissue and fluid sampling?
<whom>
If I say all these things, then there are some people in the house who say, check, post mortem, this will happen, I think this is the situation. I am or my wife is fine, either uncle Tau is someone else. The other thing is, taking the biggest decision makes a according to parents.
<who should approach>
If senior doctors say that it is necessary to get all these things done then I am the one who has come up with this new technique, if the doctor says to do a little test, then I would say yes if there is any problem then you can (f). This can be known, if the disease is detected then another child will not go (m).
<how to explain>
At that time, I am really normal man this time is enough, actually explaining to a normal man will have a very good effect, but it is very difficult to explain a high patient quality and the mind sometimes does not work for him too. Sometimes when such an incident happens. So I think, if you talk about it for ten minutes comfortably, it can be understood if it is normal, but if it is high patient then it is very difficult, then it will have to show the picture, madam means sympathy to her. Give him a little sympathy; if something falls for something, he has to support something to lift it. Actually after giving support, he should be told that this thing will have to be done, like I believe my wife is right now, if something happens to the children, then I actually told someone to me, then I said, tell me everything is fine, a little It is technical, which is being tested and it will be known that what happened to your child, which disease

was caused, it will be taken with a little injection in the body and nothing will happen, then you can also refuse it. . One can be requested twice.

24. In your view, what factors are important for acceptance of the tissue sampling by the parents and family members?

To know the reason, you only tell it, because of this, you know the reason.

Decision making dynamics

25. In routine day to day operation who takes decision about the major activities (purchases, treatment, spending money) about the family?

If we have to take a decision, both of us (husband, wife) sit and talk, the decision has to be made and the wife says that you have to go home, you have to go to the field, you have to say Which means I can say it but I can't, because that is the reason that happens often, some people go to the field, some people do it from home, so it is good to take both people, of course we meet Takes.

26. During the hospital stay who discussed with the doctors/ nurses to make the decision(s) about treatment and other procedures

I was the only one there, so I used to take decisions, the children used to stay here on the room, the husband used to go to work, see my street child here (Mother). Neighbour is here, see it, it is necessary to work as well (father). His health was deteriorating, his (dead child) was called ambulance so that even when the poor man does not have money, no one is going to know, then that person dies like that, there is no time. , Illness is something and illness tells something else.

27. after death of your child, who decided about the cremation and other post-death activities and rituals?

<who prepared body >

Our neighbours were servants, they did the work, they did all the work, there is a little 10-minute road nearby, the forest is all dead body goes there yes we did bury the baby's mud. We had gone to at least 60 people, all the people had gone, friends, how many people could not know because the relation was very big after working in the field, how many people started saying, I did not know, I said that was to be He is done.

Not in children, I don't know what happens, sometimes I have never seen the perfect mother-in-law, she told me, then nothing happens in the children, just don't go anywhere for a quarter of a month, nothing to do. Yes, she said that her mother-in-law's rituals will not work for mother home, she had told, her mother-in-law had explained a bit, all of my mother-in-law's relations had come uncle, her sister's, tau.

Summarization

28. Now, we are completing the interaction/interview. Would you like to add anything?

In-depth Interviews- Parents of deceased children-8

1.1	**Primary respondent (relation with the child)**	Mother
1.2	**Age of the primary respondent**	Mother-30-35 years
1.3	**Other members present**	None

Context-I lost my husband (he died). I used to sweep before. Right now I do nothing since my child is finished, my husband used to work hard, it has been three years since his death. After that I sweep, I have a son, I live together, but I have work and eat.

Events that led to death of the child

2. Can you please let us know about the illness and events that led to death of your child?

When the child first got fever, the little moths here had brought medicine for him to the doctor, then I suddenly saw one day I was sitting like this, I saw his eyes, then the eyes were becoming very yellow, then I took her directly to K Hospital, which is K Hospital, I admit there for fifteen days then from there, they were not able to do there, exactly, what was there that had also given glucose and blood in the jaundice, Yes in K Hospital and only injections were doing the same three medicines and nothing else, there I saw that there was a small ICU first, then sent it inside Bharti (dr), then it was sent to the big ICU up, big There was nothing in the ICU as well, there were also only glucose, the doctor there said it is jaundice, its brain has gone up Anytime you can give blood from your nose through your mouth, I said that you can give it, then you can do something in it, then we said that we cannot do anything in it, so I said that you cannot do anything, then give us leave, then speak Large we will not give leave, you can take it on your own free will, we will not give leave in writing, I will not give leave, I said, okay, you do not make a holiday, I am taking my child from here Then I brought it out on Tuesday and I took it from there again. In AIIMS it says that the bed is not empty, we will not do it again. We have no responsibility, if you are approved then you can admit, we have no responsibility, so I said, "Let's not take your responsibility, then there is no further hospital, where will we go to pay, then I admitted there on Tuesday." After Tuesday, not on Wednesday, on Thursday, the child has finished at half past one in the night, went on Tuesday, Wednesday has stopped, Thursday has started, it has finished at half past one on Thursday, stayed in the hospital for one and a half days, has also provided glucose on there. , had asked for blood at eleven and twelve o'clock at night, so the blood he could not climb, he had asked for blood, but he could not climb, he had not even filled a bottle in S Hospital, he had already spoken to us No responsibility Signed to me, anything can happen anytime, the child has no responsibility for us, did not tell anything about the chance of survival, just said that it is not our responsibility to save, now the more you lie There is more danger, then he had done recruitment in ICU, also in S Hospital in ICU.

Hospitalization period

3. Now we would request you to tell us something about the period of hospitalization for your child?

<duration of stay>

I admitted there on Tuesday, not on Tuesday, not on Wednesday, on Thursday, the child finished at half past one in the night, went on Tuesday, Wednesday has stopped, Thursday has started, it is over at half past one on Thursday, stayed in the hospital for one and a half days.

<course of illness>

In S Hospital, he had said in the beginning that we have no responsibility, had signed with me, anything can happen anytime, the child has no responsibility for us, did not tell anything about the chance of survival, just said There is no chance of survival, there is no more chance of late lying, then he had admitted in ICU, in S Hospital also in ICU.

4. Could you please describe your overall experience about the hospital care?

<about treatment>

When he had admitted, the treatment was fine, after that he sent him to the ward, in the ward, he did not even notice that the child is bleeding from the nose, we are also speaking, please hurry doctor. No what to do.
<about investigation> As soon as the test was done, the tests that were done in ICU were done in it, there is no test or check up later, 21 numbers were shifted in wards, 21 in 20.
<about cost> No money should not be spent in medicine.
<about satisfaction> She could tell when my baby was fine, everything is fine there, my child's life was so much, there was no fault of the hospitalist, in the ward when I would ask to see the child. If they were, they used to say, "What can we do, his mind is jaundiced. The vein of the brain has burst. "There were just glucose on it. Just glucose came at eleven twelve o'clock in the night. Blood came for blood, after coming blood said that it is still cold. It had to be heated when it was twelve o'clock , So much blood has been put on it, at half past one, two bottles had come, so they were able to apply only one bottle, even if they had not climbed, they had not been able to climb.

5. Could you please describe your experience about communication by the various hospital staffs during hospital stay?

<about HCP>
The behaviour was fine, everything else was fine.
<complete information about treatment procedure>
No, in the evening on Wednesday, you said that you should sign on the paper that the responsibility of the child is not ours, the child had already signed before the end, it was signed twice, once at the time of admit in the ICU, when the second time was admitted in the ward. Was signed on that day at four in the evening on Wednesday, the child has no responsibility, and on Thursday it was over at half past one in the morning. At three o'clock in the night, I was to take home.

Death and post death period

6. Could you please tell us about the death and death declaration of your child?

The doctor said that I was his brother-in-law, at that time in my hospital, the doctor said send him down to his mother, I saw that the child's latrine and bathroom had gone out, I knew that it had died. The doctor came and told me that my brother-in-law does not have your child that you can take it, the child has gone, then I said at half past one, we got a car at three o'clock and brought home at three o'clock.
Doctor told my brother-in-law that the child is not there and the brother-in-law did not tell me, the brother-in-law sent me down that we have the child sent me down, I came to know that the car has been sent down from the top. Everyone has done it, I had to make all the preparations to come home, told me at the time that I have to go home now, then I came to know that I do not have a child. No, no sympathy was given in the hospital.
<cause of death>
The reason for this was that they were telling that the vein of the brain was ruptured, if the vein of the brain is ruptured, then there is no chance of the child breaking even of the older person, then it is difficult to save, the vein of the brain ruptured When the blood started coming out of the mouth and nose, where are the chances of survival, when I was about to come home, then my brother-in-law told me that the car should be, after that he told me that we are now walking home.

7. What was your and your family member reaction to the death of your child?

<reaction of mother>
My situation was very bad when I heard that my son is gone
<did anyone try to console you>
My mother-in-law was sitting outside, she handled the same, and later both my brothers-in-law were below my mother-in-law, everyone else was out of the hospital the child had two people, his uncle and tau.

8. How was your experience about the procedure after death till handover of the body to you?

<about documentation>
Yes, he had given a little sleep that by this you would become a death certificate, he had to be made sleepy, he was a little bit, no, he did not have my sign on his uncle's.

<time taken>
It was over at half past two o'clock at two o'clock at two o'clock, we left from there at three o'clock;
<cost / payment>
body, I do not know if yes, but the ambulance had received fifteen hundred rupees.

9. Who all from your family and community/neighbourhood were present at the hospital and how they supported you during this period?

There were no neighbours here, there were only five people there, but after coming here, there were all the neighbours, my brother came from my heart, sister-in-law and mother came. No help.

Post death procedure and coping mechanism

10. What all rituals and procedures did you follow after death of your child?

I came home at three o'clock in the morning, then we buried him at 12 o'clock, we took him, at 12 o'clock in the day, no child does not.
<about body preparation>
Yes it was bathed at 12 o'clock, then immediately took it away.
<who prepared the body>
His Baba had bathed by his uncle. Tau had all bathed no nothing else is there in the child, only bathed. New clothes were brought new clothes were worn and taken at 12 o'clock. Here it is nearby or buried in Mohan. Do not allow the older child to be buried, burn the child older than fifteen years. My child was five years old, had celebrated his birthday on eleven Tariq, on eleven October, the other eleven could not even come.

11. How did you, your spouse (wife/husband) and immediate family members cope with the difficult phase?

Can't you still manage to forget, I also had my health as a jaundice, this son also had jaundice, he was gone in the jaundice, we had only three sons and his mother, so I am still tomorrow Came for 200 rupees medicine, no work, I work a little at home Right now, I cannot go anywhere for the 1.25 month until my son's. Means stay in the house, 1.25 month is going to come in the house for months, as anyone who knows, let's start coming to see for the 1.25 month.

12. Is/was there any change in the interpersonal relationships between you and your spouse and other family members after the event?

Single mother husband is not alive.

13. How did the family members support you and your wife during the difficult phase?

A lot of people supported the mother-in-law, two in-laws are also related to the discussion, the most attachment is to the mother-in-law.

14. What were the reactions/ counselling and suggestions given by relatives/neighbours/ community after the episode?

Yes, they should have been taken there, it is better than that, they should not have been taken to that hospital it does not carry in it. Many people had said that K Hospital is very good about the child, so I thought let's take it there. If they go, they give glucose in the jaundice whereas glucose does not go up in the jaundice.

15. Do you think and/or discuss with your spouse/ family member(s) about the death and related events and blame someone/something or feel guilty for the same?

No, I do not believe anyone guilty, I only believe my destiny, his life was so long, whom should I blame, doctors also do a lot on their behalf so that no patient is spoiled by our hands.

16. When did father and mother (if working) return to work after the death of the child?

Nothing to do in 1.25 month tomorrow will complete 3 week.

17. How you and your family's life has changed after the death of the child?

No, nothing to change

Knowing cause of death in detail- Autopsy and MITS

18. What was the cause of death of your child and what is your perception about the value of knowing the cause?

No I don’t know I never had heard

19. In your view, what more could have been done to know the exact cause of death and the reasons causing the disease?

<about PM>

This is like taking out the stuff as soon as the eye is done, this heart is there, it takes out, it is in it like someone went to an accident and got hanged, takes them out.

20. Are you aware of any method for identifying the exact cause of death?

21. What are your views about the autopsy/ post-mortem for identifying the cause of death?

<necessity>

It is very important to know the reason, to come forward, if someone feels fever, then be careful before taking medicines quickly.

<religious aspect>

is wrong, by the way, people say it is wrong, even if we know why, we just say that we should not do post mortem, yes in our religion this is not to do post mortem. There is nothing written that people have said so.

22. What are your views about collection of the tissue (like done for biopsy to diagnose diseases) and body fluid samples for identifying the cause of death?

He is right by tearing, and then he is okay with tearing

<religious aspect>

No, it is not forbidden. People say things should not be torn. They say that a dead person should not give too much trouble, everything comes out, what goes on living in it, the person has left his life what can he do now.

23. How parents and family members can be approached for the tissue and fluid sampling?

If you want to say that we have to do this work then they cannot refuse, yes those people are unhappy at that time, but you will say at that time that they have to do this work, they will not refuse to say that, even on time they cannot speak.

<whom should approach>

Now I was sent down as if I was not there, I was a brother-in-law, now you could ask them that if we have to do this, they do not say a little or do not do it.

<method of explanation>

They will understand from the point of view, then they will tell from this that they will know what causes the child to get the test done, those who understand will understand it even if they do not understand Do it.

24. In your view, what factors are important for acceptance of the tissue sampling by the parents and family members?

Reason to know

Decision making dynamics

25. In routine day to day operation who takes decision about the major activities (purchases, treatment, spending money) about the family?

Decision taken by mother-in-law and father-in-law

26. During the hospital stay who discussed with the doctors/ nurses to make the decision(s) about treatment and other procedures.

In the hospital, the child had tau and there was uncle and I was there he was doing.

27. After death of your child, who decided about the cremation and other post-death activities and rituals?

My mother-in-law was the rest and it was as if the relatives were there, they called, bring clothes, then clothe them, then they took the mother-in-law, she was telling them.

Summarization

28. Now, we are completing the interaction/interview. Would you like to add anything?

My second son is older than that. He was younger with whom the incident happened. He was with him (son of sister-in-law). It was one day older than this. It has a birth date of 12, it is the birth of eleven dates, By the way, I was very fond of it because my elder one is also a little naughty he was so quiet that he did not know that there is a child in the house, he was a child, he was very dear to the whole street. The street was beloved of the street the street people are also serious of listening A lot of people had reached to see that he came to the hospital, he was in such a serious condition, he used to have a bottle in his hand, then he used to go to the latrine on his own, then the toilet did not go to bed on his own, never did the bottle, hand it I was engaged in K Hospital for fifteen days, used to remain in hand, yet went to the toilet Many children do latrine bathroom on the bed but they did not. His liver had stopped working, the doctor said, even if the jaundice will be cured, it will not be right, the liver was damaged due to the jaundice liver is such a thing that there is no other reason.

In-depth Interviews- Parents of deceased children-9

**1.1**	**Primary respondent (relation with the child)**	Mother
**1.2**	**Age of the primary respondent**	Mother-32 years
**1.3**	**Other member present**	Mother's brother

Father of the child was not present during the interview as he went to dispensary for taking treatment for his cough and fever. Elder son came in the middle of the interview and answered to some questions. And other three children went to school.

2.1 Can you please let us know about the illness and events that led to death of your child?

Mother- Our child has been ill, good enough to study in school, is five years old, has written names in school, had written in Jai Bharati. When he went to study, he suddenly went to play when he came home at 6-7 pm in the evening time, and then there is a slight fever, so there is a child doctor in Badarpur taken to the doctor in the morning. They have been given medicine, it has been relaxed. Then there was rest in the night and next morning fever start, then for the second time, his (father), our father, our elder child took his brother, looked at him, then gave him medicine . Then there was rest to taking medicine. Then on the third day, there was nothing to eat or drink nothing to eat, he tell him for water, I was getting more water, then that day he drink more water, he drink water all night. Be it, after drinking water vomiting started Then he went to the doctor on the third day from where he had come to bring medicines, so the doctor said that admit in S Hospital, he ran away from the same has been taken to S Hospital, then admitted at three o'clock in the day, admitted in emergency Have to take, then he give glucose water, then the elder son is asking for water to drink, then the doctor again agreed that do not give water, offered the bottle, hence he had considered water Then broke again after the water was about to end when half the bottle was finished, then they would vomit the blood, in front of the doctor show them to the doctor, some such disease is not there, we said that there is no such disease. In the same hospital, get all the x-rays all checked, because of which blood is there, some disease has not come out. last overnight and in the morning died 10.30 am.

Hospitalization period

3 Now we would request you to tell us something about the period of hospitalization for your child?

What did the doctor tell you about what was happening, when you recruit, tell me something> Mother - I kept asking them, what is the disease we said that it is not some disease. Then the slip was shown from where they were taken, to the doctor who had taken it, they saw it, and then they said that no one is right, to ask that TB disease is such a disease, not such a disease, nothing, (badarpur doctor) said, take it quickly, bring it to the hospital

<Who went to the hospital> elder child we Just like when he arrived there at three o'clock in the emergency, the doctor took him. He was the first private doctor

4 Could you please describe your overall experience about the hospital care?

<investigations, treatment given>

There were tests done (S Hospital), gave medicines in the same time, the medicine was given in pulse water in the needle, in the same. No medicines were brought from outside.

<cost of care>

Mother- Now I do not know this boy is known, I don't know.

5 Could you please describe your experience about communication by the various hospital staffs during hospital stay?

Mother- Now I do not know the name.

<who communicated>

There was a madam like you, just like a sister was a madam, she used to come and see.

<frequency of communication>

Yes, they used to come on time. <Those who understood you, they used to understand about the condition of the child> as they were taken in the ICU, they have shifted to the ICU at 3 o'clock at night, meaning where the child ward they have been in S Hospital. Where on the other side, where it is all, we went to the hospital first, but we did not go to the hospital first.
So the compounder of x-ray done stayed with him in the night, after taking him (x-ray), then he was a madam in the ICU and then he give a medicine, which means he says comfort. Then show up in the morning, the same are admitted in the ICU, so they should take care of all, how is the child not in the same bed. Did not tell us anything about what came in the report of x-ray.
Death and post death period
6 Could you please tell us about the death and death declaration of your child?
Mother- Madam, like you, they did not speak to us, they said to their child. We became nervous like this, did not get in front, the child kept on the bed, the boy is lying like we are sitting, we know as if our hands have become cold, so we started asking his father to go to the doctor. Ask what do you do, feet and feet are getting cold, some of them did not say Yes, I saw that she was sitting, it was getting cold with her hands and feet, call the doctor and see, he said (doctor) will break it later, the doctor will say, a madam Then the teacher who came like you looked after the voice, asked the voice, then as soon as the break came, after 10 o'clock, as his father was out of the room, so he said where will he find his father, call him, Call him, see the doctor and see that he is no longer your boy. Yes, I was there at that time.
<Give reason>
No, they did not tell us, gently told their (child) father in the ear, something was told, we have not heard so much in his father's ear.
7 What was your and your family member reaction to the death of your child?
Mother- My condition was very bad, wept on the bed, she was all over the sister, the gates were the ladies, they said, now what in the world is no more than crying, it became silent, so nervous as if we were the same Will die, and cried a lot.
<Your elder son was with you>
The elder son has not been together, when he has called, he has left the house, at that time there was only our husband, and no one was just both of us.
<Fathers reaction>
His condition also worsened.
8 How was your experience about the procedure after death till handover of the body to you?
Mother- Yes, it was one o'clock or maybe three o'clock by the time I came by car after we leave from there.
<How it took so much time>
kept the same, you have to read something written, so I will go away with the words, you have not said, do not have to take it like this, I had given a small slip, I do not know where we have kept it now. He said to show this slip outside, without going without showing, to show this slip outside.
<time taken for documentation and paper work>
I was late all the relatives of the house have brought everyone along with son. Took time. Time was spent in the hospital itself, was discharged late. We left and ran into the phone at home, we stayed there and our husband was there. Bewildered in getting up and sitting. How can we call the boy then, everyone has come and brought it back.
9 Who all from your family and community/neighbourhood were present at the hospital and how they supported you during this period?
Mother-elder son reached there, our nephew also arrived, and brother in law boy also came and stayed there and our husband.
10 What all rituals and procedures did you follow after death of your child?
Mother - Then brought it home.
<rituals>
We do it like we do, after Asir, 6-7 pm, go to body then buried. All relatives had come, all the relatives of had come, and more relatives came.

The bathing has been done by all the ladies. Many men had gone for burial, they had gone from outside to home.
<timings>
buried at 7 o'clock, followed by Asir followed by Azan buried after giving, Namaz went after recitation.
<rituals after death>
The third day is done like this, then forty-fourth, 40 months after one month, 1.25 month approximately, we eat food.

11 How did you, your spouse (wife/husband) and immediate family members cope with the difficult phase?

Mother - What to do now, now slowly have to be forgotten. Now recite Namaz to divert attention from it.

12 Is/was there any change in the interpersonal relationships between you and your spouse and other family members after the event?

Mother- No, not like this, there is nothing to fight. Our boys support us, all children give courage. He who passed away was the youngest.

13 How did the family members support you and your wife during the difficult phase?

Mother - nothing, speak silently, give courage, now you will not be able to cry because of crying, you will have to forget it slowly.

14 What were the reactions/ counselling and suggestions given by the relatives/neighbours/community after the episode?

15 Do you think and/or discuss with your spouse/ family member(s) about the death and related events and blame someone/something or feel guilty for the same?

Mother- We do not blame anyone. Now who is to blame, here everyone is a foreigner, everyone in his house is the same, who is to blame.
<No deficiency in treatment>
As the health suddenly worsened, at 6 o'clock in the morning, I went to the doctor, both husband and son.

16 When did father and mother (if working) return to work after the death of the child?

Mother - Our husbands do not work right now. <This happened after this incident or already> No, work was going down before this incident, and after which even does not work. Now age is over. <Started household work> Now you do it slowly, lethargy remains by breaking it. It took a quarter of a month, 40 days to mean like this.

17 How you and your family's life have changed after the death of the child?

Mother- Did not change, there are four such children, one elder brother, three more, now what to do, they are taking care of them.

Knowing cause of death in detail- Autopsy and MITS

18 What was the cause of death of your child and what is your perception about the value of knowing the cause?

Mother- Now someone said something, the house is all the same, someone is to be blamed, no one can be blamed, the playing child is gone, there was no illness at all, no injury was there. .
<willingness to know detailed cause of death
You should not be accused of being accused, but when you know that something has been done, it will not be known that you cannot treat anyone without asking, without seeing them, you are home. Or someone's outsiders see Bigger won't accuse you.
What do you think is the reason why you were playing suddenly, came from school, did not play in the evening, nor did all the children go to play in the evening like 6 o'clock in the evening, how did they come to the game, they broke the hunger was light, then took it in the morning His father, the elder boy, showed that he was ill and then gave medicine.

19 In your view, what more could have been done to know the exact cause of death and the reasons causing the disease?

Mother- How to know now, something would have happened now. Now they think for the child, they sit down while working, mind does not work.

20 Are you aware of any method for identifying the exact cause of death?

Mother- No, we have no idea about it. Now there is no what to do

21 What are your views about the autopsy/ post-mortem for identifying the cause of death?

Mother - for the post Mortem there, we said that we have agreed there, the child does not have disease, so we do not do post mortem

<who told you to do post mortem>

In the same hospital, there was a big madam there and said, "Take away the post mortem, then take it to do, then we said that we are a small child, we will not get it done, we have agreed." When there is no disease, the disease is nothing else, then let us take it, give us a slip do not know where the slip is kept, do not know where it is kept.

<why did you refuse to conduct post mortem of your child >

When there is no disease. Now I am nervous, have brought a small child. Nothing was detected when there was no illness

<any religious barrier>

We do not have a vein, nor do we have the same reason. Don't do it like a little kid now

<do denied because of some religious barrier or any other reason>

Now we don't care what the reason is, we don't know now

22 What are your views about collection of the tissue (like done for biopsy to diagnose diseases) and body fluid samples for identifying the cause of death?

Mother - they was tied to the bag by the doctor, the doctor said, how much has the water made, he offered water. So we asked the doctor who wants to put the bag of cloth, why the cloth, said that as much as we have offered water, now we have to see how much has been poured out and how much, the bag is all over How many bottles of water have been given and how much time can they have to finish and how to put the bags.

<Explained about MITS procedure>

Did not say anything. Just like the child had finished the holiday leave at 10 o'clock, we were calling each one, it was time, the doctor said not to write the first slip, so his father wrote the slip, now he is not getting the slip, Have been kept somewhere but forgot, do not keep it anywhere, you have not received the slip, the slip

<Have you agreed about MITS>

His father and boy are not ours

Elder son - Initially four blood samples were taken, and after that when he was admitted, three more bottles came out, the total 7 samples came out, and the test was also normal. When that four bottles were added, they were normal. <What do you think could be the cause of death> may cause overdose of injections, before that there was no Sirius Before I admitted myself, I was in line in emergency, five people were ahead of me, and I would have been before them if the conditions were more serious, but I had to wait and go from my turn. First of all was injected in her, then checked with hungry hand, then saw it is hungry then injected her, after 15-20 seconds of injecting she started bleeding. There was nothing before that. From here you will see that there is such a long route, reaching there, the bond with serious condition can be even more serious, but nothing like this happens. And the slip of medicine we were giving him was also kept, we also showed him the slip before being injected, then he saw it and injected it.

Elder son came during the interview.

Elder son- <Were you, when your brother died> Yes, I was there, morning time was at 9.10 minutes. <What the doctor told you> was not such a particular disease, I took him to sit on the bike himself, if there was a serious condition, he would not even sit on the bike, because here (from their home at prahladpur) S Hospital falls far away from And do not know how to travel on a bike. There was a normal hunger then first injected after the injection the blood started to vomit which was out of control, then increased blood, glucose, then he was in serious condition and then he Was put in another ward from the

Emergency. The hands that had to give us oxygen, had to shift from emergency when the emergency is shifted when the condition comes to normal, it was out of control and then it was put out in that ward, room number. 18 are probably, there was a shift when the condition was not even normal and we had to give oxygen, pump ourselves. Because that was not the machine it was at that time.
< he did not tell>
Elder son- No, he did not tell anything. He started his work as soon as death took place. The pipe, which was set up, left all the oxygen, they immediately wrapped it and wrapped it and said, "Death is a fire, you can carry it, you said it clearly."
<Who was told that death has happened>
My father, as I was entering, my sister got a call and was talking to him, then the father came right away saying that the doctor is saying that he is no more I said go and look in the room, when I went to see that they were wrapping it.
<The Post had spoken for Mortem>
No he had said nothing. Mother - Did not say like that, he was speaking to someone. Elder son- Just said that death is over, you can take it. She gave the sheet to wrap, did not say anything to post mortem.
23 How parents and family members can be approached for the tissue and fluid sampling?
<who, when should approach>
Elder son- Someone would have understood there was no one there. As soon as the Death is spoken, well, let's see what the problem was due to but no one said anything as soon as the Death, within 5 minutes, the pipe came out of oxygen quickly, immediately to the bed. Everyone took to cleaning. Nothing was given to any sensation. Said you broke it quickly and there are other patients too. Immediately started to wrap
<religious aspect for MITS> Elder son- It has no idea, but if you want to know the reason, then it can allow.
24 In your view, what factors are important for acceptance of the tissue sampling by the parents and family members?
Elder son- At that time what did (tissue sampling) had to see what the region is what was the region of death. It was necessary to know the reason as it was not a serious condition to be killed normal fever occurs to everyone he had fever in one or two ways, and in three ways was assigned.
I say about direct treatment because before that even the normal doctor showed it, there was nothing like this, vomiting of blood. The private doctor had said that the hunger was overstated, it was not happening in the control, that is to say, in this way, we are giving the treatment, if the hunger gets up again it was then we told you so Said that take you to S Hospital, there will be proper treatment I then reached S Hospital at the same time at 12.30 pm at 1.30 pm at the doctor’s and had him admitted at 2.28 minutes, I still remember the time.

Decision making dynamics
25 In routine day to day operation who takes decision about the major activities (purchases, treatment, spending money) about the family?
Mother - We all take care of ourselves, men give gifts from outside then I see the whole thing.
<Decision about treatment>
Asked what to do for treatment, he sees the elder child. Right now my husband does nothing. Used to stitch before, now does not How the expenses of the house goes Have just rented a room below, my son does not work right now, elder son is 21 years old.
26 During the hospital stay who discussed with the doctors/ nurses to make the decision(s) about treatment and other procedures.
Elder son- The doctor said, take S Hospital and I have seen and gone, so I said that it will be the best and on the spot I did not waste time and I came home and I was getting something 12.30 Picked up the bike and took it to sit. My father had taken the decision at that time he had said to take it. They were unable to carry the bike, then I went with the bike, they do not know how to ride a bike

27 After death of your child, who decided about the cremation and other post-death activities and rituals?
Elder son - At that time my father was and my elder father had a son, who did everything as the arrangement is and my elder mother was also telling me how the rules are followed. As soon as people came to know, they took leave then at 9 o'clock, when I was dead, I called my brother, brother, it is such a thing, first of all my elder brother came to my elder father's boy, then the younger one came again. And he was the brother who came home then we brought home then whatever happened to (big mother) bath.
<rituals after death>
As the death took place on August 4, this mourning lasted for 25 ways, in whose midst there was no such thing just as the death happens to eat food we could not get it done because Muharram in the middle In the middle, it is not done between the moon of Muharram, we have got it done by waiting till November 5. Eating food blossoms in it, 40 days when the child is completed, food is served again in the death of the child or in the death of anyone
28 Now, we are completing the interaction/interview. Would you like to add anything?
Elder son- It was like everyone was there so quickly, but did not tell the reason, meaning as death happens, it is said that take it away. You have to mention the reason, once you check all the reports will be normal, even in the four samples that went, all the reports were normal.

In-depth Interviews- Parents of deceased children- 10

**1.1**	**Primary respondent (relation with the child)**	Father, Mother
**1.2**	**Age of the primary respondent**	Mother-25-30 years<br>Father-35-40 years
**1.3**	**Other members present**	None

Context- father- Brother and sister are all in Delhi

2. Can you please let us know about the illness and events that led to death of your child?

The girl was treated privately, the papers were thrown private hospital, the girl was born in Madan Mohan on 6 February then after three months the girl became ill and suffered a cold, all her treatment. First got it done in private but it was not done properly then we took it to Madan mohan. I was admitted in the emergency, the girl was seven days then a hole in her heart came out. He transferred to S Hospital then the doctor did not go there from there. F Hospital On the side of Okhla we took him there, he had surgery for his heart, yes he had surgery in Fortis his treatment was done his wound was healed, I had to go to some work post due to which I had to go home, I have gone home so (wife) is telling me, I was not here but I came later but my wife's phone was gone that the girl will not survive is serious brought home from Fortis when she was cured. Then in her fourth month, four to five months was very fine, she had reverse diarrhoea, and nothing happened, then took her to the metro station. The money is fixed, it is good to see his child, the child is not of a big age, so he took it as if there was a number, he replied that look son, I am not taking any fee from you, but only taking medicines Let's see what we had to see because you are poor, we don't want to rob the poor So do it like this, take him to MM Hospital, otherwise take him to medical or S Hospital, your girl has become very serious, yet no one has told that the girl is serious, the girl is playing a lot, she had a good time. If there was vomiting, then Madan Mohan went to S Hospital dispensary and then admitted, then put him in the heating machine, the girl's hand had failed completely, then put a pipe in her mouth and put a pipe in the way of urine water bottle Yes, this is the matter of Madan Mohan then he directly transferred to S Hospital. He lived there for one day and one night, at 3 o'clock on the second night, the girl was dead, yes in S Hospital, one day remained one night and the other day. In the second night, the girl was finished, meaning it was a night for two days, in S Hospital, it was over at four o'clock in the morning, we had also asked for a bottle of blood it had been two to three hours in Madan Mohan, at 7 o'clock we had gone to S Hospital at 10:30. We had reached, had asked for ambulance to refer.

Hospitalization period

3. Now we would request you to tell us something about the period of hospitalization for your child?

<duration of stay>

Took there one day and one night, on the second night, the girl was dead at 3 o'clock, yes in S Hospital, one day there has been one night, on the second day, the second night, the girl has finished, meaning two days had been a night in S Hospital dead it at four in the morning.

4. Could you please describe your overall experience about the hospital care?

<about investigation>

Yes, blood test was done, x ray was done, urine test was done, the whole test was done, heart surgery was done, it was done in Fortis and there was also (S Hospital), yes there we too Every week I used to go to S Hospital and doctors used to call us every week, we used to go out at 6 in the morning.

<cost of care>

3500 rupees was deposited, then 1500 rupees of blood was deposited, then it was spent so much, 1500 because this needle will come from outside for the child, so at that time I had no husband, no brother and brother. Just my mother in law and I was So I told the doctor, look, we have no one, our mother-in-law is blind where you sit and leave her here, and if you do not get lost somewhere, then I gave the money, then do not take tension, we will come, then we came In front of us, who had taken 3500 rupees of blood, I had

given me a card of blood, the blood had come, the whole bottle had climbed so much that the whole had survived, could not climb, till then the girl died.
<quality of care>
See, we got it according to the mix. Ok, now look at your doctor and see for yourself that you have done so much, if your child is not there then what is wrong for us, I have shown the system right there, got what we said to someone (M). Our child has not been found, what will we complain to anyone now? Nowadays no doctor wants this (F). According to us, it was fine according to the mixed from the time the girl was admitted, the doctor started checking herself, then in the end he said, look, trust the person above, now you will be lucky if the girl will be saved. People do not do such a thing, do not do wrong. We said, now it is up to you, I thought it was right on one basis.
5. Could you please describe your experience about communication by the various hospital staffs during hospital stay?
<communication of HCP>
The conversation was fine, there were only two or three doctors right, but the rest of the doctor was all dumb, the boys (doctors) also came. Ask from the heart no matter what hurts, are you getting work money Let me talk to her, let me talk to her, I came to her speaking to her on the same day that my girl was also discharged, on the same day I came and said, See what pain is there from a mother's heart. You are not even married, I think, I came in this way, you are the doctor who is doing it or we have left it all, I have spoken like this, you think we are not educated, we have a thumb impression, I had spoken to three doctors, I told them Sir Madam, the child's breath is getting stuck once you see "those people started speaking" You have produced a unique baby " I got into an argument with two or three doctors (M). The condition in which the child's breath was getting stuck at that time could have happened (F). He was a doctor, but he was very poor, but he had given a lot of help to God so good wife should be happy that he did not lack anything in his life, he did not refer, he was a doctor of S Hospital but 100 was right in 80 percent (M). Many doctors used to talk well, got government jobs, got good money (F).
<information about child condition or treatment by doctors>
No, no one used to tell, just say that you have faith in the above, your child's condition has deteriorated a lot already, so I said that you have not taken care of yourself, so please refer me and we know to produce children Even if we put 60, 70, 80 thousand, then we will not mind, I even said this, do not say it cannot happen I had also asked, if you do not feel Sir, then you will refer me somewhere and we will see you there if there is a good doctor of your life so much that if your brain is not being used in a child, I have opened up Talked to the doctor, you tell me base Lesley let us out of the hospital, we are so tense, So this is what the doctor said, "No son, you do not tension your child is 80 percent, it will be fine, just do not remove oxygen from the mouth". When I went to admit, I had only asked what had happened, I was in reverse and had diarrhea and nothing had happened, what happened was the report came, it did not tell me anything, what is the disease of the girl, ask more. "You walk out, you answer so many questions the poor man can only ask and what can he do, nor can you debate or fight with you."We have also thought that if you produce children too, if you die thinking, then do not bury me in the house, but go to S Hospital," I will never go to the government.
<about nurses>
The nurse has it all for her own meaning, we have come so much that we feel that how the nurse talks that if it is time to see, then she is looking after two hours, many are talking Drinking coffee
<during round of doctors>
What did you say about the child? Well, it is not already well, and did anyone ever pick up the hair and see the eye? Just the ladies in the room were all getting sad, "Stop the doctor there I used to feel like I was so angry in the room "I was so angry, I had no place to put nurse dipping in the body, where they have not dipped on the two, they used two sticks together This side is being planted, the other is putting that side It was with the girl (M). You are also a doctor, you have to tell one by one that you have to put everything together, from one side to another, the nurse is lying there, not seeing what is happening to the baby, the girl's voice is not coming out, Her body had done such a thing, that girl kept looking at her, she used to keep on tearing the bus, I have come to sing the time we used to call her fondly when I used to say son

would hold my finger. My girl died, that means she did not let her drink water, in such a way that her thumb went in the mouth (Father weeping), she drove me out, dying my daughter (F). That is why I had to fight with her in the name of S Hospital as well. Do not call any doctor here (about us), the doctor also said that when the girl child was finished that your blood money was deposited. You take the money, I said my girl died, even the money was dead, we are poor Kothi is not passing in the bungalow. Came to the doctor's mouth, take this, hold your hospice, I don't want my baby gone, I don't want money, so much fight took place that day from the doctor

Death and post death period

6. Could you please tell us about the death and death declaration of your child?

<cause of death explained>

Did not tell anything, only the doctor did not turn white, he had put in the hospitals and had referred, said, ask for the car, so I said do not tear the car, do nothing, do your auto, take the girl from the auto and fire. I was sitting with the child, neither did I look at the child, nor did I see him, nor did I fall unconscious, I was seeing that nothing happened, I was talking to him a while ago. Then I blinked once I understood the girl was finished on the table I fell down immediately, what happened in front of you, how did you fall, then I bid sister, my girl was finished, then I said to the doctor, once the doctor went to see my baby girl, when the doctor came, now you are saying that you are at home Take away, we are currently off. They did not give any reason why the girl finished, those people used to come only and used to give needles, then the needle did not work then they went away.

<any sympathy from HCP>

No, nothing, that's why we hated S Hospital, three children were finished that day, and my last girl was finished in the same room. Our baby girl was in the seventh month of the seventh month.

7. What was your and your family member reaction to the death of your child?

<reaction of mother>

The doctor also said when the girl was finished that your money was deposited, take the money, I said my girl died, even the money was dead, we are poor, Kothi is not passing in the bungalow that my girl is dead My money also died, I did not want any money, I torn the paper and came to the doctor's mouth, grab it, hold your innards I do not want my baby gone, I do not want money, so much fight took place that day from the doctor. When I was with the child, neither did I look at the child, nor did I see her nor I fell unconscious, I was noticing that nothing happened, I was talking to her a while ago, then I was seeing the same I blinked the eye, I understood that the girl was over the table, I fell down immediately. What happened in front of me, how did you fall, then I bid sister my girl was finished.

8. How was your experience about the procedure after death till handover of the body to you?

<about documentation>

Yes, it was signed on paper, my sign no, my (husband) sign was taken

<time taken>

The girl was finished in half an hour, called us here, took our sign and said, take it and then do your auto, after taking the child home by auto, the girl was finished by 4 o'clock in the morning. There were fires, twenty minutes of 4 or even 1 minute were happening, I did not notice, no, they did not give anything, did not even give ambulance, we came auto reserve

<about cost>

No just sign Curry took the baby girl and came away. Whatever money has been taken before them, let alone see what has been taken and what has not been taken (f). 1500 rupees of injections and 3500 rupees of blood are close to you. Send them. I said no sir. No one is with me (M). Why did you spend so much money, why don't I lie to you, why do I lie? I am not going to lie It is a different thing to eat, but do not blame lies, we can hear what you have explained (F).

9. Who all from your family and community/neighbourhood were present at the hospital and how they supported you during this period?

When the girl was finished, all the people had come to the hospital at that time, our brother-in-law, Husband had all come, at that time and we had served both the people had reached the car with the motorcycle, all got out of the gate, Yes, we called and told that the child is no more (m). I did not get

angry, did not tell anyone anything that was the wife who spoke (F). I got angry with the fact that I am speaking then to the doctor that my baby girl is not surviving, please feel free to refuse and give it to someone else (M). At that time he said that you have a unique child, there is no answer from me (F).
My family was saying this, leave the son and trust the above person, what has to happen has happened, now what will be more than this.
<What type of support given>
All the people had come, all were there, yes everyone helped that even my baby girl does not have as much weight, her father is sitting in debt till today, I am filling up the money of the girl's debt It was not, my child would have survived, that wealth would have been in front of my eyes, 80-90 thousand, we had put it in the hole in his heart, this whole area is public, we husband wife had raised money on the 20 rupees for 10 days instead of money. It was cured, she used to play very well there was a reverse diarrhea (F).
Fortis was taken from Madan Mohan to the doctor, yes surgery was done there in free, medicines used to come from outside, they were expensive, one bottle of fifteen hundred rupees was so small, some two and a half thousand, some three thousand and three thousand Ka, used to go for medicine and neither bothered the mind, it was so expensive girl's.
Yes, I was able to serve what I had to offer, then later we are giving it to him, yes many people have given support, they say this, now we are forced to speak wrong, we will not get the money in the month of tax. Every month, we will give you tax slowly, then we will do it slowly, we have given the rest of the money and its 20 thousand rupees are saved, then we will give it slowly by tax in 2-4 months in this round (M).
Post death procedure and coping mechanism
10. What all rituals and procedures did you follow after death of your child?
I was taken from here it is near to Jamuna ji. I do not burn a small child Kalindi Kunj went from here to the car, talked there and dug a pit across the land and then buried it
Yes, he had asked for money there for Rs. 3100. Then I said I am a small child. we are very poor then we give 1600 and 1000 for vehicles (father).
<who prepared body>
My elder sister came from my house, there was little sister, there was Devrani, there was Devar, and the girl of our mother-in-law was fiery Was bathed.
11. How did you, your spouse (wife/husband) and immediate family members cope with the difficult phase?
We do not worship, etc. in people, it happens later (M). I tell them that they do not know (wife) as there are little girls nor they feed them, the rest is not there in us, they were fed after five days, as many girls as eleven twelve, as many as twelve little ones came Is (f). What to do now, we will not come back from crying Now four bottles of water have gone up in his affair, what can we do? The medicine is also running, this (live child) still cries, I told him today that the doctors will come and take Pucci today I said yes Babu is crying then (M). So we said that it is right to tell that the book is ok (F). She was the youngest, she is the eldest, then she is and she was the youngest
12. Is/was there any change in the interpersonal relationships between you and your spouse and other family members after the event?
After her departure, we did not believe it, but there was so much girl, every other third day, her box of 302 rupees used to come with milk, since the girl finished, she did not know what was there in that girl. , From the day the girl has gone, we do not know what disease has taken place, do everything, do something, something is happening, the girl is not getting out of her mind, it looks like here I am sitting, someone has taken out the bus to rotate, I feel like I will come from somewhere in a while. After that incident, we have formed a relationship, it is breaking up, I do not know what has happened. After that relationship which has gone, we feel strange in our house as if the girl has taken some time to visit me.
13. How did the family members support you and your wife during the difficult phase?

14. What were the reactions/ counselling and suggestions given by relatives/neighbours/ community after the episode?
This is what the sister-in-law neighbor is not able to do in today's time, so what is the service, you also know, listen, sister, if we have four paisa, neither does the world support and when a bread in the house If there is no flour, then the person does not come out and ask for bread flour for the child, nowadays it is time, sister, this God who has given me poverty, asks God not to give anyone.
15. Do you think and/or discuss with your spouse/ family member(s) about the death and related events and blame someone/something or feel guilty for the same?
No, I do not believe anyone who spoke to me, the people above said what I had written in my luck with me, now it is expected that those who are left are our two (about live child) which we have It is up to these children well, that only their names will be known, what to do, what to say more than this, it is so sensible not to run into anything. Now we poor man stays for 24 hours day and night, we come in the evening at 7-8 in the evening, then the children live in food, then there is tension in the morning that they have to do something in the morning tomorrow, look at the children in the morning Let's go, I leave at 6 in the morning, he comes at 7-8 in the evening.
16. When did father and mother (if working) return to work after the death of the child?
We could not go to work for a month, it used to cry here, it did not leave me, it still keeps sticking like this, we have to come again and again from work, we go so far, weeping so fast (wife) calls that this (live child) is not getting silent, now you had called, then I had come back from half a distance and was going to work, so you called me so I thought that you are so far away If you will stand then I will go (F) after half an hour.
17. How you and your family's life has changed after the death of the child?

Knowing cause of death in detail- Autopsy and MITS
18. What was the cause of death of your child and what is your perception about the value of knowing the cause?
sister, we said the same thing that we have not read enough to know all this, now that you have asked people, then you will tell that you will inform the number is near, tell us, we will not be able to tell more than this (F). According to your accounts, everyone is saying that there was a hole in the heart, put all this money and made all the things, when there is no child, then what will happen to them, what will they do because of their life You tell me, the girl remains alive, so let us also talk to you about it, brother, for this reason we need that what is there in the girl that the girl is not doing well, now that she has been dead, what has happened to her (M).
19. In your view, what more could have been done to know the exact cause of death and the reasons causing the disease?

20. Are you aware of any method for identifying the exact cause of death?
<about post mortem>
So much was known that the girl has had a heart surgery, so much was known that the girl has a hole in her heart, she cannot take deep breaths, and as the child has toothache if she has toothache They cannot reverse, what is the reason for this, the doctor did not tell me, we have said, look, Sir, if we feel bad, speak on your face or let me out of the hospital. What is the reason that there is weakness in the liver that there is a hole in the heart, we just knew that there is a hole in the heart. Just after his treatment was completed, he was given medication for three months to two and a half months.
No, I do not know about it. If we do post mortem according to people, do not do it in the right way or do not do it, according to us, it is very wrong to do post-mortem, after doing a post-mortem a person lost his life even on his death. Do not like it, then tear it apart, we only say that any government would have been formed, but not to do so was lost, so the post mortem is very useless.
21. What are your views about the autopsy/ post-mortem for identifying the cause of death?
<about post mortem>

Whatever is done in the post mortem, it is told that we also know this much, and we do not know much about it. Post mortem, see if a man is hanged and dies, he also has an accident, he also has a bullet, when the patient goes to the doctor, then the doctor says that this medicine was given to the patient. When the post mortem is done then there are many ways too. The whole body will be irritated, what it is, I have not heard much about it. According to me, all this is a useless thing, a person has gone poor by dying, if his body is made dead, then it is out of my mind, yes, he bites more. What is left in the post-mortem, Madam, who takes away the work of the body, we also know, what is left of it, we take money and suppress it. (F)
<religious aspect>
It has not happened in our family, look, we would have known so much, we have been talking about it, our father was over in childhood, I was younger then only the elder brother was finished, then all the younger sisters are younger. Brother, how have you grown up, then married everyone, have done everything

22. What are your views about collection of the tissue (like done for biopsy to diagnose diseases) and body fluid samples for identifying the cause of death?

<about MITS>
It will be known about this that if all his tests have been done then we will also know the disease, at least seventeen diseases of a human being live, who says we do not have TB, if we have all this disease We will not be able to live a little, we will not be able to walk and this is what the doctors say. (M) This is fine, Madam, it is okay with the injection, the account of the tearing is not good in my mind. (F)

23. How parents and family members can be approached for the tissue and fluid sampling?

<whom should approach>
It is as if what is to be believed that I am the father of the child, the mother of the child, can give permission that will be fine, then our understanding is that it is mixed with what you have said from our mind. The best thing is, because the chord which is the rag, it is the most useless, that poor child is already gone, after his death, whether he should be a child or a child. That sister is not right with our mind. At that time, if I had asked me, I would have agreed, I or our wife would have accepted that thing, if you had said that I would tear a child's trunk, I would not agree to it. So let’s not happen. (F)
<methods to be explained>
In the same way you have just talked, in the same way that you put in our mind at that time, we would have told you that it is okay sister you can check the injection with our child. (F)
<who should approach>
As if you are a big doctor, you understand that the way you are talking is explained in the same way, because the man is already hit by his gum then his mind gets mad. I do not come, it is very well explained with a good mind, "Brother, look, your child is gone we cannot give a child."
If you say this thing or you do another type of test, then you have got this injection in your brain that we will give you the opinion that my son is sick, do not tear your teeth if you can do the whole test by injection so use it "(F).

24. In your view, what factors are important for acceptance of the tissue sampling by the parents and family members?

I liked your only question that through which this girl was finished (to know the cause of death), which disease had got inside it, which, after so much treatment, my child did not get rest or benefit (F).

Decision making dynamics

25. In routine day to day operation who takes decision about the major activities (purchases, treatment, spending money) about the family?

I do all these things my elder brother was finished my father has expired. Most of the time, my madam takes care about the treatment. The bone also has a problem, its expenses are different, no job can do the work of weight, if an accident had fallen, fracture has occurred, then the same junk is lying out and it is spent on it.

26. During the hospital stay who discussed with the doctors/ nurses to make the decision(s) about treatment and other procedures

At that time there was no brother-in-law in the hospital, only I and my mother-in-law were there, I was the only one there, I used to do it.

27. After death of your child, who decided about the cremation and other post-death activities and rituals?

No one told me, I had it done by the hand of a younger brother there was a pandit, who provides the child, there in Kalindi Kunj (F).

Summarization

28. Now, we are completing the interaction/interview. Would you like to add anything?

What I asked, what you asked of us, we told, who knew anything (F). Like, if we want to ask you this, if God does not eat crispy if we produce other children, if the above God does not give the same dish to his God, if suddenly we have an affair with S Hospital, then in the company of a mother but such Must not be moving What happens is not a very difficult thing, which has not happened in the company of people (M). This poor man who has no hearing in the government hospital, if he does not go to take it or even the fever checker, he says in his purchase, if we can do this thing ourselves, then why should we go to the feet of people Not only will we do it, but not the thing which is known, the person who has knowledge, he will go to the same, why we can do the thing which we do not know, if we know about any work So you will speak to us Raju Bhaiya, we do not know about this, you know that you can do this work, which will be good, you will get it done. If we know how to do this, then we will definitely do this work to show fake, we do not listen at all in the hospital. (F) What do we have to do if we do not live even then you would also say that stop in the room and play what will be seen (M). I have been 3-4 times in Madan Mohan, will give a bullet for the waist, no check up, no nothing. It was also done by private X ray, expenses in private X ray again went to S Hospital, there would be an operation, at that time there were no children, I did not even have one. Whoever is in the private we will get medicine, they will be lying in the operation, they will not be able to sit, they will not be able to do any work will they eat? Is there a wrestler here? The old man was treated there on the first strip, at which time he did So in the first strip I got rest Had to walk a bit and started getting up, there was 4 bandage, after that there was no money, then did not get treatment, later daughter, we have to see our children again. (F).

In-depth Interviews- Parents of deceased children-11

**1.1**	**Primary respondent (relation with the child)**	Father
**1.2**	**Age of the primary respondent**	Father-25 years<br>Mother-20 years
**1.3**	**Other members present**	None

2.1 Can you please let us know about the illness and events that led to death of your child?
Mother- He was in ESI for a month, after that, after a month, he was not well, then he was discharged, then we brought baby home. Then after two days, he started having fever, again we took him to hospital, there he checked him, he has cough, then they kept for two days, then on the third day epileptic attack started coming, it had never come before.
Father- we stayed only for one night at S Hospital.
Hospitalization period
3 Now we would request you to tell us something about the period of hospitalization for your child?
Father- The child had pneumonia, cough had gathered in the chest.
<what doctor said to you in S Hospital>
Doctor, when we went to night, he got an X-ray done, got an ultrasound done and then he referred to the ward, then they were giving medicines , then in the morning his (child) temperature increased by the time I went to fetch the cannula, his breath got faster and then went away.
Grandmother- Child was 4 months old.
4 Could you please describe your overall experience about the hospital care?
Father- All the tests were done on the spot for the child. They got X-ray done, ultrasound done. His report was received then, after looking at it, they told us, all report was told to us. No money was spent in S Hospital.
5 Could you please describe your experience about communication by the various hospital staffs during hospital stay?
Father- how we can know about behaviour in one days and night time. They did not say anything while admitting.
<The condition of the child was told>
As soon as he went, he told that the condition of the child is bad, its lungs are drying up due to which its temperature is getting high, fever is coming, whatever is coming, they said that they would try and see. Before doctors visit the baby died. He died at 8 in the morning. We arrived at 11–12 at night.
<who was communicator>
There was a doctor in the Emergency, she was lady doctor. She was not a senior doctor, she was a junior doctor.
<nurse beaviour>
Father-There was no nurse there.
Mother- The nurse was there. We were in ward 18. The nurse was not coming in but was just sitting. In morning I felt child was having fever then she (nurse) said that if you come to get cannula then I will inject it and I will not put it like that. <You were told to get cannula from outside> no from where doctor used to get in ward.
Death and post death period
6 Could you please tell us about the death and death declaration of your child?
Father- I was there only. I came to know that it was on my lap, it had stopped breathing in my lap, I understood that is no longer there.
Mother- As he was playing earlier, I put the cloth on him to go and get insert the cannula. < Then you call the doctor > father- The doctor was going from there, it was time for duty, she was going, so I showed baby to her, then she pumped the baby chest then saw that his breathing was not coming, even after half an hour his breathing did not come. <any sympathy/empathy>Father- At that time, Madam told us that we tried but we could not save him, the child was ill since night because we were late enough to get him at this hospital. Your child was admitted in ESI for a month, you used have paid attention to this thing. If no improvement was seen you should have changed.

7 What was your and your family member reaction to the death of your child?
Father- I managed to control but baby mother condition had deteriorated. <did someone try to console you and your wife> Father- By then my aunt had arrived there, she handled my wife.
<Mother started crying during the interview>
8 How was your experience about the procedure after death till handover of the body to you?
Father- The paper work was all done in an hour. At that time, it did not cost anything. At that time, their behaviour was fine because the doctors had also come, 3-4 madam had come, so there was no difficulty at that time, they had made the papers on time. From my family mu aunt came.
9 Who all from your family and community/neighbourhood were present at the hospital and how they supported you during this period?
Father- I and my wife were just in the hospital at that time.
10 What all rituals and procedures did you follow after death of your child?
Father- First the child was brought to home and then from home, there is a cemetery in front of it, after going there, he has buried it. Yes, the child was bathed first after that he was buried.
<Rituals followed at home>
Father-child was bathed then wears the shroud and then burials at the cemetery were performed. Grandmother- even the child is small or grown funeral prayer is performed for everyone, Father – funeral prayer was performed.
<What time Azan was given>
Dadi- after the burial 40 steps backward moved then azan is given after burial at burial place. Father-ritual is followed for 40 days, it was for 40 days. <What happens at that time at home> Father- First, fourth day, then 10th day, 20th day and then 40th day is considered it is all done according to Quran.
11 How did you, your spouse (wife/husband) and immediate family members cope with the difficult phase?
Father- Now you will have to recover and neither can you sit in that thought. He was our first child.
<Mother started crying again when asked about coping>
12 Is/was there any change in the interpersonal relationships between you and your spouse and other family members after the event?
Father- There was no difference in our relationship after he left. I take care of her from the beginning, everyone also takes care of it. Grandma – we involve her in household work.
13 How did the family members support you and your wife during the difficult phase?

14 What were the reactions/ counselling and suggestions given by the relatives/neighbours/community after the episode?
Father: The family members say that what is going to be known, no one can stop it, everyone tried it completely, everyone spend day and night to save him.
<reaction of family members>
Father- they also says the same thing. Everyone says this, who can stop what going to happen, all suggest to wait and calm. No one said why you take baby to that hospital. I and my wife were both there (S Hospital) we had to decide where to take it and where not to take. I was with my wife and mother, only three of us were there in ESI hospital.
15 Do you think and/or discuss with your spouse/ family member(s) about the death and related events and blame someone/something or feel guilty for the same?
Father- we don't blame anyone. It is just that whatever carelessness has happened, it has been done from our side we have kept baby for so many days at ESI hospital.
<What was the treatment in ESI>
The treatment was running normal it was going on for the last one and a half months. They were saying child will become alright, there will be improvement, improvement was also happening, the child was getting right even he was getting normal at home. Then we had fever we again took him to ESI.
16 When did father and mother (if working) returned to work after the death of the child?
Father- I went to office ten days after all this happened.
<when did mother started doing household work>
Her sister came to help her she mostly do the household work, now my wife is also helping her.
17 How your and your family's life has changed after the death of the child?

Father- everyone still sitting in sorrow, what is the change.

Knowing cause of death in detail- Autopsy and MITS

18 What was the cause of death of your child and what is your perception about the value of knowing the cause?

Father- Yes, the reason was told by the doctor that the lungs have dried up due to its cough.

<Not wanting to know anything from the doctor>

Actually we already knew what the reason was, everything was told in ESI had already at time of discharge. They told us his windpipe is narrow he is premature as he was born in 7 months.

19 In your view, what more could have been done to know the exact cause of death and the reasons causing the disease?

Father-all things were already declared that what was the reason. The report was present there is nothing that they have kept secret. He told whole thing beforehand. Reports were all given.

20 Are you aware of any method for identifying the exact cause of death?

21 What are your views about the autopsy/ post-mortem for identifying the cause of death?

<views about post-mortem>

Father-post mortem is done when death is not identified it is done to know the reason. And it is also performed in claim cases, from which reason death happened, or whether doctor is not fooling, post mortem can happen for many reasons.

<religious aspect> Father- No there is no religious aspect it can be done. But we don't need this as we know everything.

22 What are your views about collection of the tissue (like done for biopsy to diagnose diseases) and body fluid samples for identifying the cause of death?

Father- No, we do not believe, because it is not needed, because we all knew it, there was no such secret. Father- No, we do not believe, because it is not needed, because we all knew it, there was no such secret.

23 How parents and family members can be approached for the tissue and fluid sampling?

<whom to approach>

Father- If something like this happens to someone else, then it should be to talk to the child's father about the technology, because the ladies are broke and continue to weep in sorrow to take such a decision at that time, because it is male who can decide for that.

<Who should approach>

Whoever is a senior doctor there, or who is available there, or whatever the person concerned is there.

<Mode of communication>

Can be understood only by talking, at that time no one will want to see a picture or video.

24 In your view, what factors are important for acceptance of the tissue sampling by the parents and family members?

Father- The reason may be that if the parents do not believe that they have done the treatment properly or not, baby may have some other disease and medicine is going on in that case. Correct treatment is given or not.

Decision making dynamics

25 In routine day to day operation who takes decision about the major activities (purchases, treatment, spending money) about the family?

Father- It is decided by my mother that what to do.

Mother- treatment decision is taken by my husband (child father).

26 During the hospital stay who discussed with the doctors/ nurses to make the decision(s) about treatment and other procedures.

Father- It was mine and my wife decision to take baby to S Hospital. My sister had some reference of one doctor at S Hospital we took their reference because they were not admitting directly because ESI next hospital is K Hospital there primary hospital was K Hospital so S Hospital was taking it. There is one doctor in charge who helped us in getting admitted at S Hospital.

27 After death of your child, who decided about the cremation and other post-death activities and rituals?

Father- I, my brother and my uncle performed the last rites. The decision of that time is taken by senior of the house. In our family my uncle friend was telling everything.

28 Now, we are completing the interaction/interview. Would you like to add anything?

Father- Yes, we knew that the reason is not very accurate many times, some people do not know what is happening or what is not happening, which medicine is going on, even when asked. Some people could not ask due to doctor behaviour. But nothing like this happened with me, all of things were told in S Hospital.

In-depth Interview- Parents of Deceased Child 12

**1.1**	**Primary respondent (relation with the child)**	Mother
**1.2**	**Age of the primary respondent**	Mother-25-30 years<br>Father-30-35 years
**1.3**	**Other members present during the interaction**	Sister in law

Context- child's mother and child's chachi was present during the interview, the mother told that the child genetic sample has been taken for knowing the illness/cause of death in private hospital.

Events that led to death of the child

2. Can you please let us know about the illness and events that led to death of your child?

Mother-first child had fever sine March onwards till May then we showed him to local doctor, he was good doctor.

Sister-in-law- we first went to doctor L, DG, then to AKM and at last to A, all are private clinic.

Mother- he was admitted in L hospital for a week, at that time he was having swelling in stomach, doctor said there is no swelling, child has this type of stomach. Fever was getting normal and then again he was having fever. Doctor was also not able to understand what the problem was, then my father-in-law told doctor to give us advice, then doctor said he will have to continue medicine for long time. No investigation took place for stomach or for his disease, other test and ultrasound was done.

<What they said after admitting the child>

They said child is having pneumonia, fever, they gave him medicineand injection he got better after that they said now they will start treatment of stomach for that they conducted ultrasound for two times. They informed there is water in body, liver is enlarged. Then my father-in-law asked him for specialist, then he referred to G hospital. We went to Gangaram there they admitted my child. There they performed CT scan, endoscopy and other test including genetic test. They said there is no cure for the disease.

Aunty- there was some genetic problem.

Mother- I feed him serlax on 2nd October, on 19th he died, he was given syrup, it went to his throat, his face turned white, after that he started screaming, even had difficulty in breathing, after that doctor started treatment, then he was given injection, then his heart stop beating.

Aunty- he was given medicine in series when he was admitted.

Mother- after having medicine, he immediately had difficulty in breathing, before that my child was fine, he had swelling in stomach, then in gangaram he had swelling in hand-feet, when he was in ICU he was having swelling in head, his hair was also falling, doctor said there is no cure, we had spend a lot of money for his treatment and doctor was not telling what will be the prognosis of that. Then we took him to S Hospital. They didn't refer us to safadrjung hospital.

Hospitalization period

3. Now we would request you to tell us something about the period of hospitalization for your child?

<What happened at S Hospital>

Mother- On 10th of October we went in the morning at S Hospital, they did all the test etc.

Aunty- test were done there in the afternoon and then he died.

<duration of stay>

Mother- 8 days, when on 1st day they saw the child, they said the chance is less but we will try our best, they conducted several test, there was no ventilator, when his heart beat stopped for the 2nd time, then they brought the ventilator to the ward, they didn't start his medicines even he was not given food or milk. He was getting food at G hospital. They didn't start any medicine which he was getting at gangaram hospital they were only giving glucose, and at gangaram his heart beat stopped for atleast 4-5 times.

4. Could you please describe your overall experience about the hospital care?

<course of illness>

They didn't tell us whether it was improving or deteriorating, there were about 5-6 doctors including junior doctor, they used to talk among themselves in English, which we were not able to understand. So what did I not say about medicine, etc. I used to ask them again and again that what medicine is to

be given, when to feed they did not say anything, they used to listen after a long time, they did not pay attention.
<quality of care>
I did not like the behavior of staff there it seemed that where I have come, they used us to go here and there, behaved so wrongly that I did not like it someone is already in pain, so I did not like the behavior and treatment there.
<about investigation>
They didn't tell anything about test report, we used to bring the reports. They were keeping the reports with themselves, they used to note the report in diary, and they didn't tell anything to us.
5. Could you please describe your experience about communication by the various hospital staffs during hospital stay?
<about communication>
We used to take samples and we used to come after taking reports, they didn't tell anything to us. Everyday after 24 hours we used to get to know that he was not well, or his heart is stopping or not, then doctor used to say that his condition is not good, in betwwen nothing was told.
<Senior doctor used to say that>
The doctor used to come and say his condition was not good, they were having ambu, they were not having ventilators. The child suction was need to be performed, I used to clean, I asked twice to doctor that how to perform that, she told me once, and when I asked nurse about medicine then nurse said to give four medicines/tablets then my father-in-law said four tablets of yellow color it was brain medicine, they even started Gangaram medicine, we were not able to find one capsule, my father-in-law got angry and said what it meant to give four pills, they didn't know about the rule of giving medicine , doctor know how to give but they were not present. They know but they were not there at that time, the medicine I gave him came out of the mouth and then the same day doctor said that its condition is very bad.
<behaviour of nurse>
it was fine, they used to give them injection, what they were giving was not told to us, child had fever in between medicines were given every 4 hours. Facility didn't have thermometer, then I bought my own.
<cost>
Aunty- money was spent on the medicines. A lot of money was spent at Gangaram hospital.
Mother – a lot was spent in G hospital.
Father: we spend nearly 5 lakhs in Gangaram.
Mother: And he was already admitted.
<Satisfaction about treatment>
Father: S Hospital is a government hospital, there so many patients come many children come that's why children die because a lot of patient comes there.
<satisfaction with S Hospital >
Mother: No we are not satisfied. Father-no not satisfied.
Mother: After that, they did not even go to the child, did not even check how is it, what was it the nurse did not come to see.
Mother: Senior doctors used to tell something, junior doctors did not tell us anything.

Mother: I used to ask doctor about child condition but did not say that much, they used to say less hope is there child condition is very bad, and the expectation is very low, we are doing what we can do, She only spoke but she had not started the medicine.
Mother: Medication means that it was not started since the admission. He was getting medicine for his hert and breathing, no medicine was given realted to brain, and he was not feed.
Father: The child had gone there, he did not get a single feed for 10 days.
Mother: And his brain was fine in Gangaram, but when he went to S Hospital he got swelling and his condition had become very bad there.
<who were present from family>
My husband, my father-in-law, brother-in-law and wife was present with me.
Aunty: we were always present.
Death and post death period

6. Could you please tell us about the death and death declaration of your child?
<Who declared>
Mother-on 18 at 7:30 in evening his heart beat stopped, he was given medicine, I didn't know the rule of giving medicine I gave 1 tablet, so when I gave medicineit came out of his mouth, his breathing also stopped, my father-in-law was also there, doctor removed pipe from his nose, then I came out of there, after 10 minutes now he was fine, then my father-in-law told me to go, he stayed at night, it was last day in hospital.
<What was time of death>
Mother: I did not even know at 5:30 in the morning.
Mother- I slept at night and wake up at 5 in morning, I was there in hall.
Father: I was not there at night that day.
Mother: He was in the office
Father: The child also needed ICU, but ICU was not available.
Father: I brought the child home.
Mother: There was a lot of blood coming out of his mouth.
Mother- doctor were again and again changing his nose pipe due to which a lot of blood was coming out, I don't know whether it was changed in Gangaram or not, I don't know why they were changing frequently, whether it was needed or they were changing to teach others.
Mother- the pipe was also there for faecal matter, after that pipe was changed he was not doing toilet since then, earlier he used to excrete 500-700 grams.
Mother- they were not giving proper treatment and not taking proper care of the child.
7. What was your and your family member reaction to the death of your child?
Father- what reaction, you must know that will the reaction of the family.
8. How was your experience about the procedure after death till handover of the body to you?
<Time taken>
Father- they left us at 7.
<about documentation>
Paper work was going on they made me sign the paper.
<assiatance from staff>
They made us sign the paper, my father signed. I went there at that time but no support was given.
Mother- till last day, I didn't met the doctor and nurse, I directly went outside, at night at 7:45 I came out, then didn't went to child, the guard didn't allowed me to go inside.
Father- we were in ward no.18.
9. Who all from your family and community/neighbourhood were present at the hospital and how they supported you during this period?

Post death procedure and coping mechanism
10. What all rituals and procedures did you follow after death of your child?
Father: What happened we took the child for burial.
<bathing of body>
Father: No bathing took place
Mother: bathing happen in adults not in case of children.

Father: we went to 40 feet lane for burial.
<who came during burial>
All family member and neighbour came.
<who supported>
Father: There were family members and neighbours who were supporting us.
Father- we bury the child.
Mother: Since my child was born, progress was very good, laughing, speaking, playing means that there was no problem, even he understood.
Mother: child was delivered normally. He was 15 months old, then he had stomach swelling and fever due to these two things his condition worsened.
11. How did you, your spouse (wife/husband) and immediate family members cope with the difficult phase?

Father- no prayer happens in us.
12. Is/was there any change in the interpersonal relationships between you and your spouse and other family members after the event?
Father- there is no change and tension between us.
13. How did the family members support you and your wife during the difficult phase?
Father- relatives support financially, as a lot of money was spent in G hospital, at S Hospital no money was spent.
Father- all supported and consoled us.
Father- I went to work after 10 days.
14. What were the reactions/ counselling and suggestions given by relatives/neighbours/ community after the episode?
Father- some gave suggestion that you should have taken child to CP Jain some said to take at K hospital.
15. Do you think and/or discuss with your spouse/ family member(s) about the death and related events and blame someone/something or feel guilty for the same?
Father- i dont blame it is of no use, because at government hospitals doctors are less and patient are more, no nurse are there, there are more guards when compared to nurse, there is no system to work, in one ward four ward will be there, bu one nurse for all.
Mother- there is no cleanliness.
Father- treatment didnt stop it was continuing since 2 at night, but doctor didnt tell us clearly, 5-6 doctors saw his stomach all said it was normal , when it started swelling then some decision was taken.
Mother- first they said he is anemic.
16. When did father and mother (if working) return to work after the death of the child?

17. How your and your family's life has changed after the death of the child?

Knowing cause of death in detail- Autopsy and MITS
18. What was the cause of death of your child and what is your perception about the value of knowing the cause?
Father: I have given this to get the test done, to know the reason. (do you know the reason for death)
Father: Yes-yes (you are saying genetic test)
Father: I have given it to test (report has not come yet)
Father- child DNA test was to be done.
Mother- the sample is given at G hospital, after that his condition was bad, so we decided to do test later.
Father- yes we want to know the result of the test.
Mother- we wanted to know what and how it happened
Father: person had probably just gone to Bangalore, we got a call for the test, just two days ago, 24 thousand is asking for that test.
Father- yes I would like to go for that test.

Father- in those 24 thousand two tests will be conducted.
Mother- the doctor told there is 30-40% chance that this condition can occur in next pregnancy also.
19. In your view, what more could have been done to know the exact cause of death and the reasons causing the disease?

20. Are you aware of any method for identifying the exact cause of death?

21. What are your views about the autopsy/ post-mortem for identifying the cause of death?
Father: post-mortem did not happen they had spoken for the test then.
Mother: Post mortem only happens in accident, etc.
<religious barrier>
Father: No, there is nothing like that, there is no religious barrier.

Father: Those people had not given any such suggestion, the doctors asked whether it was necessary or not.
Father- I know about post-mortem
Mother- we didn't face any situation like this earlier, that's why didn't know about it.
Father- there is cutting of the body, this much knowledge i have.
Father- this (MITS) was not done.
Father- i would have done, if someone had told about this.
Mother- this would be helpful for future.
Father- we would have known the reason.
Father- we would have known cause of death, it will be helpful for future pregnancy.
Mother- we would made clear, that we would keep in future.
22. What are your views about collection of the tissue (like done for biopsy to diagnose diseases) and body fluid samples for identifying the cause of death?

23. How parents and family members can be approached for the tissue and fluid sampling?
<who should approach>
father-if doctor would have told us we would have agreed about that.
<how to explain>
Father- this doctor could tell better.
Mother: As the condition of our child was very bad, we didn't have power to think what to do next, should we take to other hospital, whether other hospital would take the child on seeing its condition. It was very difficult to think what to do.
24. In your view, what factors are important for acceptance of the tissue sampling by the parents and family members?

Decision making dynamics
25. In routine day to day operation who takes decision about the major activities (purchases, treatment, spending money) about the family?
Mother- my father-in-law takes decision regarding purchase and treatment.
Aunty- father-in-law takes the decision.
26. During the hospital stay who discussed with the doctors/ nurses to make the decision(s) about treatment and other procedures.
Father-in-law was present, but I used to get medicines.
27. After death of your child, who decided about the cremation and other post-death activities and rituals?
Father- my father was present at that time, he was taking all decisions.

Summarization
28. Now, we are completing the interaction/interview. Would you like to add anything?
Father- I just want to say that S hospital is not for serious patints, for serious child private hospital is fine, at all places there is issue of money, that's why people go to S Hospital, if the child is saved at S Hospital he would always need someone support. They were not telling the truth about his treatment. Also in gangaram they were not telling the true picture 1% also, if it was less then they used to tell child condition was very bad. They were telling 10 diseases with one disease. When they were discharging, they were telling that everything of the baby has been corrected the kidney is working normal the child is passing urine, normalizing everything and telling that everything was healing.

Mother: very helpless people go there, when they give up, the same people go there and wise people do not go, and most of the time I have seen all the patients who have lost the same people. Everyone at this hospital who came said that I have come from this hospital, I have come from that hospital, I felt so much there, so what to do, I had to come here at last and the reason for our arrival was that the doctor at gangaram said that there is no cure for your child condition if we could have got treatment there we would have stayed there.

Mother: My child in S Hospital was 24 hours without ventilator, in beginning he was on ventilator but when they saw that his pulse is all right, the breath is right then the doctor there decided to remove from ventilator. But after removing it, when it was removed, it remained for 24 hours, then left it the same way, child was shifted to the ward the next day, then his heart stopped beating again, then if there was a need for ventilator then somehow My father-in-law has got it done by speaking and we needed ICU but no ICU was available. Due to unavailability of ICU he could get right medicine. In ward only 1 medicine was given through syringe so that his heart could beat.

In-depth Interview- Parents of Deceased Child 13

**1.1**	**Primary respondent (relation with the child)**	Father, Mother
**1.2**	**Age of the primary respondent**	Mother-35-40 years<br>Father-40-45 years
**1.3**	**Other members present**	None

During interview father and mother of the child were present. The child was first admitted to DD Hospital, there he stayed for one month then he was referred to S hospital, were he stayed for only one day.

2.1 Can you please let us know about the illness and events that led to death of your child?

Father: We have the nearest DD Hospital, our child was 22 days, he got loose motion, went to loose motion that he has lost motion, he is doing lettering again and again, but the lettering was not water- Is doing water, then said what was fed that the milk was fed upstairs, said that at this time we cannot give anything, we will not give medicine, just drink mother's milk, we said that we are not drinking mother's milk. Do whatever you say, just drink mother's milk, immediately sent us no medicine, no treatment.

<Did they refer to S Hospital>

No, it was 22 days. Then DD Hospital went to say that its diarrhea is not going to stop, feed him the same mother's milk and we have nothing, come, then we have taken him here. That the child is breathing fast, said that you have to do it quickly, you would like to do it in a nursing home or in DD Hospital, we said that it will not be done in DD Hospital. Will not, he wrote on the slip, took 500 rupees Said, go to S Hospital, take it and see it immediately. <Did not admit in DD Hospital > No, first kept a month, when I moved here from the nursing home to DD Hospital, then they kept admitting for a month, the baby did not gain weight, and the weight of the baby kept going down, just Just understand that there is a lack of water, glucose has to be offered. I said, offer it, glucose is given, then it is said that it has got pneumonia inside the hospital, said that now the pneumonia has worsened. I said that you are a doctor, you will treat us, we will do whatever is happening inside the hospital, neither do you, then we are watching all those tests, these tests, they did those tests when the baby was born So 2 kg was 800 grams, if discharged after a month, 2 kg was 400 grams, the weight lost, there was no significant success, he said leave, show it in OPD, bring it home On the 26th of the night, when I came, the child started breathing very fast. I said, what is the matter, I took advice from my friends and said, DD Hospital, I will give you rest, I said, I will take it in the morning, DD Hospital, after taking a shower in the morning, I said that such a condition of the child is happening, now they started saying that it was just 10 days ago. , How is it deteriorating now, he said that there is no blood in the body, we said that you have discharged, you should know what to do with the child, say take the child to S Hospital, the child will not be treated here.

Hospitalization period

3 Now we would request you to tell us something about the period of hospitalization for your child?

Father: Yes, on 27th we were taken from here, S Hospital directly, said, don't even ambulance, don't waste time for ambulance. Yes, (said in DD Hospital) the child was breathing very hard, we were not even drinking milk, doing nothing, taken to S Hospital, to say directly, the child is not breathing, no blood in the body of the child Turned yellow I said, "Let's talk with us, how will we take the ambulance? No, S Hospital is taken, then how are the children sent by the DD Hospital having so much problem, how did they send it without oxygen?"

Father: Yes, I went straight to the emergency, said why sent it, it was to be sent in an ambulance with oxygen, I said what can we do now as we said we took the auto and came, they admitted it and that pipe in mouth and nose Put, put pipe in place of bathroom, put syringe in hand, will have to say blood, then blood is given, blood got blood in a long time, some blood was given Run the pump separately, we will give breath, the pump was kept that two, keep pressing it, we kept giving it, then it said that the condition is very bad, we cannot say anything and there is no space in the ICU, I said no space in it If so, what will

you do? We will do something, refer you, say we cannot do anything we can send it to the ward. Yes (ward no. 18). They sent him to the ward and his health worsened. And the memes there are so many doctors, that doctor was speaking very diligently, she is thin, she smokes, one will stay alive, if you want to be treated, then do not make me do it. How to do a Jana, the child is also crying, they left me out of fear, as much as it happened, they sent me to Lao Medical to get the test done, to do the blood test in AIIMS, go there, I have been running all night, this night of 27 I was so elated Yes (27 at night). It was midday on the 27th, on the go, then on 28th morning I got run away, take this test, take that test, in the last I did not give my madam test, telling me that this test has not come, I said that we have given, we are not giving the report, they are saying, report two, we will cure today, no matter how we will treat, they said, last injection was given, and now there is no chance, child Sit near There is no chance now that the Madam child has died in front of us, meaning that we have wandered in DD Hospital from the beginning, you will cure the child who has lost motion, I will fix the government hospital which is open for what you can cure. It is not enough to drink mother's milk it will be okay, baby, drink mother's milk. I said, what should I do if mother's milk is not there?
<Medicine to mother for milk production> not given (no medicine given).
Mother-vitamin syrup was given
Father- Just, given a vitamin-D to the child later to give it yellow, but there is no improvement in the baby, the child was born very healthy. <Normal delivery was> No operation. It was on full time The child is up, but the child corrected in the operation. Now what can I say carelessly, <You stayed for one day in S Hospital > Yes, 27 was gone, the child was finished on 28th. Morning at 1:10, no-no in the afternoon.

4 Could you please describe your overall experience about the hospital care?

Father: Now used to send me again and again to get these tests done, now go to the new building now go to AIIMS. <Did you get AIIMS report> I had got the report. Those who were not telling anything, those who came in the report were telling the doctor (S Hospital).
<cost of care>
What medicine was given? They kept the pipe pulses, but nothing else, they used to put the injections.

5 Could you please describe your experience about communication by the various hospital staffs during hospital stay?

Father: Nobody tells you, (Doctors used to tell whether someone is improving or not), the condition is bad. Yes, he used to say that.
<behaviour of doctor>
The ward who was 18, the doctor was so filthy, he was thin, he was very dirty, I did not see any such ladies, witchcraft, did not talk in a manner whose child is about to die. You have no way of talking
<nurse behaviour>
Was the same for everyone No one was there, one or two nurses who were she was fine, but no one else, there is a very bitter doctor, there are number one. Did not say anything, did not say anything.

Death and post death period

6 Could you please tell us about the death and death declaration of your child?

<death declaration>
Father: I called the doctor and brought the child was not breathing, then all the doctors were coming, I feel that its breath has stopped, yes it is coming, it is just now, I said where it is, now She is crying and I am crying here, I feel that when I am giving a pump, the baby's stomach is bloating.
Mother crying during interview
Father: <because of the pump> yes sir. Means put the pipe on it.
Mother: There was bleeding from the mouth. I brought that blood is coming, they come and speak, we cannot leave the pump, you call, then we came to say that the blood is coming from the mouth of the child, so what did they get out of the mouth? , A lot of blood was coming.
<when did they told that child is no more> Father: 1:10 pm 12:30 pm He finds out that the child has swollen.
Father showed the video of the child.

Father: There was a lot of trouble with this. <who declared> was a junior doctor, he did not give time of 1:10, he means heart failure due to lack of blood, I said when we were undergoing treatment at DD Hospital, he did not treat it properly, as much blood was not formed. You are leaving so much.
Mother: Saying that the heart has failed due to anaemia.
Father: Yes, the blood drained in S Hospital too (the blood spilled in S Hospital too), the syringe that was put there turned blue at that place, meaning that they are joking like this. Yes, they used to say that who had given it away.
Mother: Blood was extracted 4-5 times; after every half an hour the blood came out, the whole hand turned blue.
Father: From where he had to offer blood, as if it was a joke for him, how much blood was flowing, how he was moving in such a fun way, I am telling you about the emergency, there.
7 What was your and your family member reaction to the death of your child?
Father: There was no one doing, how they were handling each other at that time.
Mother: No one was there, no doctor nor nurse, just kept crying and carrying my baby, my brother came.
Father: What would we have done while falling there? Later at the last time, when he was leaving, his brother came then from there we did auto, brought the auto here.
8 How was your experience about the procedure after death till handover of the body to you?
Father: Nothing, baby is over, take it.
<any reason of death>
There was no blood in the body, the heart failed so that's all. <No time or any problem in signing> No, let's say, it's done soon, not that stop, go quickly.
9 Who all from your family and community/neighbourhood were present at the hospital and how they supported you during this period?
Father: We were both just, yes. Now used to send me again and again to get these tests done, now go to the new building now go to AIIMS.
10 What all rituals and procedures did you follow after death of your child?
Father: He had brought home (where did you take it), everyone had come and kept it.
Mother: Saying that take away nothing can happen now.
<rituals>
Father: Nothing but sitting and wearing clothes, took him to the crematorium. The child was not bathed.
Mother: My father and everyone came and then took them. The children who came to are buried, they do not burn.
Father showing slip which he got from S Hospital
Father: Only I got it from the hospital, yes to get the child out and when the death certificate later. It is near Mayapuri (burial place), Delhi Cantt is located nearby.
<who all came>
We were father-in-law, our father-in-law, our brother-in-law, everyone were.
<rituals after death>
Father: Nothing happens. Mourning is still going on in our house, now when we see the photo in the phone, weeping starts.
11 How did you, your spouse (wife/husband) and immediate family members cope with the difficult phase?
Father: Daughter, now she will play at home, stay with the children, then take some rest in the day, then go out in the evening, and I come at 10 o'clock at night, never mind, and called If you know me, then I will come, I will see.
12 Is/was there any change in the interpersonal relationships between you and your spouse and other family members after the event?
Father: No, nothing just handles each other and pays attention to the child.
13 How did the family members support you and your wife during the difficult phase?
Father: What did you think, whose person he took, he says, it was not yours, comfort you, one to the other.

14 What were the reactions/ counselling and suggestions given by the relatives/neighbours/community after the episode?

15 Do you think and/or discuss with your spouse/ family member(s) about the death and related events and blame someone/something or feel guilty for the same?

Father: Here (child mother) used to tell me that he had taken DD Hospital - DD Hospital, could not show in private, said that DD Hospital Government Hospital is where the baby was born, everything happened, but the doctors here were negligent with him. , When we are running again and again.

Mother: They took out all the blood, when the blood was completely working, Jake gave it to me, and I was saying, Sir, the doctor himself said that there is a lack of blood, even if you want to offer blood, you can offer They were blood, I was saying there is a shortage of blood, then do something, saying that it will be right before you take yellow milk, it will be correct, if you wanted to offer blood, you could have given blood.

Father: Yes, when the delivery took place, they took a bottle of blood, they did not even offer it,

Mother: Did not even offer me, saying that there is no need, and that child should also be discharged, not offered, if he could have offered it, it is said as soon as it comes that everything is right.

<do you blame someone>

Father: I obey DD Hospital if I had stopped his diarrhoea at the first time, it might not have happened today. No, the treatment is not good there it is not good at all.

Mother: Everything is done due to DD Hospital negligence, whatever has happened, nothing happens if the child is not finished

Father: He was very careless that even when I am asking you, tell me the situation of your child, yes, yes, it is fine.

Father: Yes, you kept one month, the weight of the baby is not increasing, then you will see, I will see, if I had to see me, why would I bring it to the DD Hospital hospital, why did you feed me upstairs, I Said we fed the milk upstairs, when the mother is not doing, then you are telling me, what to do if the baby is crying, let it die hungry, we are also asking you what to do if mother's milk is not happening Give

Mother: When I came from there, I was drinking my own.

Father: It was very short, the baby's stomach was not even filling, it was a gap of seven years, the mother's milk did not increase in the middle, we said that if milk is not happening then what should we do in that mother's milk Just drink, oh tell me how to drink, yes, give some medicine, otherwise you give me milk outside, give some powder, so that the baby's stomach is full, not mother's milk.

Mother: He had said that give some medicine, etc., that milk starts descending, no such medicine is said, just correct your food and medicine, nothing happens. No, even after the operation, I had not been offered blood, they were right.

Father: Took blood, when you will give blood to the treatment, when you take the blood and do not offer it to the child, nor offer it to the mother of the child and they are saying that the blood has died.

Mother: But these people, after doing tests all over the world, sucked all the blood of the child out of the blood, his body is white, the photos are all there, the lack of blood is from here, do not test the surplus funds Nothing happens.

Father: Now when you are saying that you are not happening, some kind of hospital, Lady Harding would refer

Mother: Even the bones were taken from the back to reach the water but then they are saying that it is weak, so could not come out.

Father: Now there is no place in the ICU, when there is no place in the ICU, then you see that the condition of the child is worse, the child will be kept in the ICU and sent to the ward, three beds on one bed, yes, S Hospital In the |

Father: The situation is same here in DD Hospital, three times on one bed.

Mother: Not three, five, five.

16 When did father and mother (if working) return to work after the death of the child?

Father: I started ten days later. I was three months, two and a half months with the rest of the child, when the child was finished, I used to run away with DD Hospital on the next day, kept the admissions here for a month, then the child was on holidays, my work was very little at that time. Ever since the child was born, even when it was over, my duty was not complete, ten days in a month, fifteen days went just like that and my private job is there, now the owner has told the problem what to do if the child is admitted.
Mother: Even four-five times before pregnancy.
Father: Even before pregnancy, diabetes has happened four or five times, we have to administer more sugar, if not, we will do the cleaning.
Mother: Initially, not before but not after, then started insulin.
Father: Yes, coming home and injecting.
Mother: Used to come home.

17 How your and your family's life has changed after the death of the child?

Father: What is the change, that is what happened to the rest of the boy then I am happy that the girl is a boy, it was a boy after seven years, after seven years.

Knowing cause of death in detail- Autopsy and MITS

18 What was the cause of death of your child and what is your perception about the value of knowing the cause?

<any other reason apart from heart-fail>
Father: Yes, everyone said that the heart has failed.
<willingness to know the detailed cause>
Now what do I want to mean, I tried a lot, everyone tried to understand a lot, I myself was in depression so much depression, that I myself had to work after working , Bp I was high I have been admitted to DD Hospital, it is a matter of fact that only last week it is said that the doctor said that he is taking tension, yes he is taking tension, BP medicine said that you are taking too much tension, my hands and feet work Stopped, said this is happening due to tension, said you stop thinking more, now son is finished how are you saying that you forget, no one can understand ours

19 In your view, what more could have been done to know the exact cause of death and the reasons causing the disease?

Father: Now to find out the cause of death. If there is an accident, then even if some say what is there about the child.

20 Are you aware of any method for identifying the exact cause of death?

21 What are your views about the autopsy/ post-mortem for identifying the cause of death?

Father: Yes, it is true that if there has been a death in the hospital, the child who has taken a lot and if anything ends, then the post-mortem should be done, no matter what is said, now what will be known when you press the matter inside. Inside, the doctors suppressed this matter.
<religious aspect> No, there is nothing like this in our religion

22 What are your views about collection of the tissue (like done for biopsy to diagnose diseases) and body fluid samples for identifying the cause of death?

Father: No, nobody told me anything about the investigation.
Mother: Samples were taken from the bathroom, but there was no test HOA.
Father: No, nothing was taken after death.
Mother: Samples were taken earlier but nothing later, yes for treatment, all this did not happen.
Father: Yes, yes, I would have agreed if someone told me to do it.
Mother: I went there that day, the dispensary was saying Madame, bring some paper, etc., we should find out who did what, what happened, suddenly the child was absolutely right, you got some paper I said, Madam did not get anything.
Father: Here is the dispensary behind.
Mother: She was saying that it seems that suddenly it happened that this happened to you, it was over, then the child was right, healthy, you brought it that day, I said Madam, we do not know that blood Heart failure due to lack of

23 How parents and family members can be approached for the tissue and fluid sampling?

<who should approach> Father: Someone would have given us advice about this, we had not told it.
Mother: Did not tell at that time, if you told, no one came.
Father: It is like, we had controlled a lot on myself, now look, we were still crying, we had kept a lot of care to each other, what I can do now, speaks to us also that you can know the reason, we take blood and blood from here, then we would agree, yes, because it is over.
<method of explaining MITS>
understand only by saying, yes.

24 In your view, what factors are important for acceptance of the tissue sampling by the parents and family members?

Father: No, the treatment is not good there it is not good at all.
Mother: Everything is happening due to DD Hospital negligence, whatever has happened, if not the finished child, nothing happens.

Decision making dynamics

25 In routine day to day operation who takes decision about the major activities (purchases, treatment, spending money) about the family?

Father: Who is the decision to cook the house, is a wife.
<treatment decision>
remains mine

26 During the hospital stay who discussed with the doctors/ nurses to make the decision(s) about treatment and other procedures.

Father: The decision of both of us was whatever the hospital was.

27 After death of your child, who decided about the cremation and other post-death activities and rituals?

Father: The one who was a Pandit there. Give that milk like this to two children give milk pulses, salt pulses.
<from your family>
was my mother.

28 Now, we are completing the interaction/interview. Would you like to add anything?

Father: What will be the solution?
Mother: We also want to know what has happened, but now when there is no evidence, it cannot happen now
Father: They have time to talk on the phone, they have a head, see the children coming and coming, in S Hospital, it was the same situation, you are busy making fun of each other, no one is watching the children. Because your job is sure, you will get salary, if someone dies, it was like a joke for them.
Mother: And when the vein could not be found, there used to be a place somewhere, saying that the vein is not being found, to apply the cannula, even the whole body was lying blue, here in S Hospital saying that A cannula has to be applied on the forehead, saying that the vein is not being found, the whole is lying blue, said to get treatment, I said that it is done in DD Hospital sir, saying that it has a breath block, it was applied in the foot. Negligence was the biggest thing they did they just strayed and took them to S Hospital.
Father: I said write and give it to the referrer, saying that you should tell me that the child is being like this. Did not write it. I was discharged ten days ago, from here we were at home, I went to Emergency, I said, look at the child's condition, that day was Saturday, 28th was Saturday, I was off The day I was at home, I will not take it, I will come to check once, I went straight to the emergency, then he said, do not waste time, take it to S Hospital.
Mother: Just spent the whole night talking, crying, not drinking milk, mouth being removed again and again, disturbed the whole night, then went on like this on the second day, then DD Hospital sent people to S Hospital.

List of In-depth Interviews of parents of deceased neonates

S. No.	ID	Age	Religion
1	Parents 1	Mother- 20-25 years<br>Father-25-30 years	Muslim
2	Parents 2	Mother- 25-30 years<br>Father-3-35 years	Hindu
3	Parents 3	Mother- 25-30 years<br>Father-30-35 years	Hindu
4	Parents 4	Mother-30-35 years<br>Father-30-35 years	Hindu
5	Parents 5	Mother-25-30 years<br>Father-25-30 years	Hindu
6	Parents 6	Mother-20-25 years<br>Father-25-30 years	Hindu
7	Parents 7	Mother-35-40 years<br>Father-40-45 years	Hindu
8	Parents 8	Mother- 20-25 years<br>Father- 25-30 years	Hindu
9	Parents 9	Mother-20-25 years<br>Father-20-25 years	Muslim
10	Parents 10	Mother-18-20 years<br>Father-20-25 years	Muslim
11	Parents 11	Mother- 18-20 years<br>Father- 25-30 years	Hindu
12	Parents 12	Mother-30-35 years<br>Father-30-35 years	Hindu

In-depth Interview parents of deceased neonate-1

**1.1**	**Primary respondent (relation with the child)**	Mother, Father
**1.2**	**Age of the primary respondent**	Mother- 20-25 years<br>Father-25-30 years
**1.6**	**Other members present during the interaction**	Nani, Maternal uncle (mama of child)

2.1 Where do you usually go or who do you consult during your child's illness?
Father- We goes to the doctor.
Nani- She is our daughter, she stays in Muradnagar, and she gave birth to her child there. And for treatment she was referred to Meerut.
Father- We goes to doctor in 12 extensions it is 1km away, he is good MBBS doctor.
2.2 Where do you usually go or who do you consult to vaccinate your child?
Father- There is Kendra, we go there.
2.3 People in your area / community are generally asked for their children's illness?
Father- The people here goes to government centre where doctor sits it is 5 minutes away from this place
2.4 Mention the reasons for priority for the facility and / or healthcare provider.
Father- When child suffers from cold and cough, any other problem we go to that doctor only. For vaccination also of mother and child we go there only. The behavior of doctor and nurse is good. There is treatment for elder as well as for younger one, medicine is also available.
3.1 Can you please tell us about the illness and events that caused <name> your child to die?
Mother- The doctor only told that meconium has entered and nothing else. I was admitted in private hospital in Meerut, I was alright and I was normal, only they told meconium has entered, they kept my child for 2-3 days under machine, when the condition got worse my brother told me to get admitted at S Hospital.
Father- Then, after 6 days this happened (Death of child).
Mama- In Meerut only, they told us that the child will not survive, we took the risk and brought the child to S Hospital. We go to S Hospital only, there we get good facility and they don’t take money. Then, I brought him to S hospital, he was alive for 5 days in hospital and then died on 6th day.
First of all, in M hospital they took a lot of money from us, it is a private hospital.
Father- in M hospital, they didn’t show the child to us, so how could we know whether the child was ok or he is breathing or not.
Mama to father of child- You must be having the report of the child they are asking you where the fault did happened.
Mami- The expected date of delivery went 3-4 days above the date mentioned in the ultrasound. The movement of child was not felt in the womb, less movement was there, then she (Child mother) started having problem, then she was admitted in Meerut, there she got treated and her doctor refer there only.
Nani- Then after keeping her whole night, they told delivery will happen through big operation, because of meconium.
Mother- Rate of big operation was high, but it was necessary, so they delivered by baby through normal delivery performing little operation.
3.2 Who supported you during illness and your child's hospitalization?
Mother- When the child was admitted my whole family was there, my brother, his wife, my mother, my father, all were there, my in-law’s family was also there, all people were together.
4.1 Now you are requested to tell about what should happen when your child is admitted in the hospital? (Investigation: hospital, ward, length of stay, course of illness)

Father- Everything was fine in S hospital, when ever there was difficulty in child breathing, we would call someone they used to come, they tried their best, but they already told in emergency that condition of your child is critical. Doctor told us about the serious condition of the child.
Nani- They kept telling us that child is serious.
Father- Doctor kept telling us that child is serious, and chance of survival is less, they told clearly that there is only 1% chance.
Mama- Chance is less, it was told in S Hospital as well in Meerut.
Father- Cleanliness was there, but there was problem from guard side.
Nani- They cared for our baby, when we used to call them, they didn’t delay for 1 minute, they used to come.
Mama- We got a lot of facility in S hospital. For 5 days he was on oxygen, we used to pump by hand, for this his father, my mother, my father was present for 24 hours, because oxygen was coming through pumping by hand, machine was not there, so we were giving by hand. The baby also got better, for 3 days he was well, he also opened his eyes, even breathed, we also have video of the child.
Nani- We used to pump by rotation, three people used to stay in hospital. One of the reports even came correct, and other was not ok, they kept telling the condition of the child as serious.
Mama- The child was suffering from a lot of problems, firstly meconium entered, one of the tubes was there on child, one in mind, this was only told by doctor, and second problem reported by doctor was that blood is deposited in the chest, and they told openly that chance of survival is only 1%.
Nani- They were saying, he is surviving for these days otherwise he may survive for 2 hours only.
Mama- The investigations done were good, for the blood examination we used to go with syringe in new building, whether it was day or night all investigations were done. The investigation was done moderately as it was necessary. I also take my child to S hospital, they do a lot of investigation, they did all checkups, and performed all investigations.
We are satisfied with treatment, we also tried from our side, but we could not save him.
Nani- Yes, doctor tried to save the child, and we also tried but when luck is bad so what can we do.
Mama- They did not change the tube on the last time of the child, which was in child mouth it was filled with garbage, they couldn’t change that.
Father- Together, four tubes were changed, there his condition started deteriorating, garbage was there in the tube.
Nani- For draining water out from his mind one tube was there, the clear water was drained from his nose.
Father- The tube which was put for breathing, in that garbage was there and some blood was coming out, the child condition got worst when four tubes were changed.
They were coming and checking the condition. They changed the tube 4 times, once the heart had stopped working altogether, there was little carelessness, they used to make mistakes 2% out of 100%.
Nani- No, the doctor used to give attention to the child. The doctor looked after our child.
Mama- Doctor should stay at this time, because at this any time he may be needed.
Father- No, expenditure was there in S hospital, we spend money in U.P. hospital.
Mama- No money was spent for investigations, only they told us to bring 4 injections from outside, otherwise no money was spent.

4.3 Can you describe your experience about communication by various hospital staff during your hospital stay?

Mama- There behavior was fine, doctor used to see the child, check the child and used to tell about the condition of the child that it is not good. The senior doctor used to come twice, one at 9 o’clock in the morning, then between 3-4 o’clock, after that other used to come (who sits in room meant for doctor and nurse), medicines was given three times, injection was also given, the water which was given to the child, in that also they used to give medicines 2-4 times, blood transfusion was also done.
Father- Firstly, doctor used to talk to each other, then they used to tell us, we used to understand what they told us, the other who used to accompany senior doctor also used to tell us.
<MOTHER CRYING>
<nurse behavior>

Father- The nurse was good in the hospital, she used to come before the medicine got finished and give medicines 3-4 times, and when we needed any help, then we used to call her.
<supporting staff>
Father- There was fine, but why we need to talk to them, they were okay.
<guard>
Mama- What we should say about guard, they have habit in every hospital that they allow only one person, for us two people were allowed. All gents were there, two used to stay inside and other stayed outside. Whenever, we used to see that cleaners are coming then we left the place ourselves.

5.1 Can you please tell us about your child's death and death declaration? (Investigation: Who had announced the death, body language and expression prior to the events before death)

Father- When I was pumping, then there was no movement in stomach, heartbeat was there, I put my hand over his heart to see heartbeat. My younger brother was with me I told him to call doctor, then she came and told us that he is no more. <who told you> Junior doctor who sits in the cabin told, he checked again for our satisfaction. Then senior doctor came, who comes in the morning. Doctor told us he is no more then, they were talking among themselves, then I called my brother to come quickly to hospital.
Mama- As we felt, the condition of the child is bad, then doctor came, and checked and then told your child is dead, then after 5 minutes senior doctor came.
Father- Senior doctor were not there, they were called from the cabin, then they came and told that heart has stopped working.
Doctor stayed there for 1-2 minutes, and then left then my brother came and he saw the file. Before this incidence, the tube was changed. The nurse did not say anything about the child.

5.2 Do you blame someone / something for the death of your child?

Father- No, we do not blame anyone. To say, U.P. doctor told us that operation will take place, I had gone to deposit money for blood as they told blood will be needed, and when I came back I got to know that there was a normal delivery.
Mother- U.P. doctor forcibly performed the normal delivery, because of which child had the problem. There were six doctor, they all were trainee doctor, no senior doctor was there, they forcibly performed the delivery, because of which child health got worse, and he didn’t cry. Neither they told me I have delivered girl or a boy, did not say anything.
Mama- We got discharged her from U.P. hospital after 4-5 days and thought that now we will show her to S hospital.
Mother- When the U.P. doctor told us that big operation will take place, they were also preparing themselves for that, they even took blood from 2-4 persons, they didn’t even transfuse the blood to me, the time which my husband took to deposit the money, by that time they did the normal delivery. There who had undergone big operation, there child was with them and who have undergone small operation their child was not with them.
Mama- Many complaints were told of U.P. hospital.

5.3 How was your experience about the process after death until the operation of the body?

Mama- After death, I reached hospital in half an hour were they gave the slip and told that you can take the child. Then, at night we took the child to U.P. by van with our own money, as hospital do not provide ambulance, it is only for Delhi and we buried the child in morning 10-11.
Father- They provided us the slip which is needed for death certificate, it was provided us my taking signature, the nurse took the signature.

5.4 Who supported your family and everyone in the community / neighborhood during this period?

Mama- Only gents were there in the hospital at time of death, the child father cried, everyone knew it was god will. Ladies were not there. My sister health was not good, only child father was there with the child, his grandfather and one brother were present, then we took the child in night and came.
<staff sympathy>
Mama- Everyone is busy in the hospital they resumed their work after telling about child death, as many more children were having some problem as our child, even 5-6 children like our child died before the

death of our child. Our child was lucky, that he survived for some days. He even opened his eyes, started getting better, before that he did not cry, did not even breathe, then he even started breathing.
Mama- Doctor already told that blood is deposited in the chest, this was told 2-3 times, we are doing treatment but he can die anytime, this thing was told in both hospital (S Hospital and U.P Hospital).
6.1 What procedures did you follow after your child's death?
Mami- Azaan, was given to child in his ear.
Mama- After birth of the child, Azaan is given whether the child is girl or boy, as in your religion Gayatari Mantr is recited. In hospital we did not performed any ritual, just brought and buried him. Next day Fatima is performed, for that Quran Sharif is read.
Father- At 8.30 PM the child died.
<time taken after death>
Mama- We two brother took the bike and went to hospital. It took an hour to reach hospital.
Nani- These two brothers, brought him.
<cost>
Mama- The ambulance which we hired, only money was spent there, otherwise no money was spent. This is good in S Hospital. Rs 2500, was taken by ambulance for carrying him to U.P.
6.2 How did you, your wife and family members face a difficult phase?
Father- Nothing was done, I resumed to work after 3 days.
Mama- You should ask this to mother, father/men resumes to work, mother is upset more as she kept child for 9 months in the womb. Still, she has not overcome out the grief.
<mother crying>
General talk
Mama- The government should put more restriction on private hospital as they try to rob poor man, they write something else, tell something else. Private hospitals are more problematic. We also faced problem due to private hospital, the centre government should make pressure against them
6.3 How did family members support you and your wife during difficult times? (Investigation: burial / cremation rituals, community norms / rituals, phase, coping with financials)
Mama- Everyone supported us. At 10 o'clock in the morning next day he was buried, we left the hospital at 10 o'clock in the night, and at 1 o'clock we reached the house (U.P.). The guests came in the night. All the relatives arrived. The baby is bathed and buried; anyone can bathe the child, whether the person is adult or a child, he is bathed, then wrapped in a white shroud, a fragrance is put on it and then kept. All this was done in the morning. Everyone went to cemetery from our side as well as from their side, Namaz, the prayer is read. Afterwards, the prayer is read for the person, so that god apologize him, whether the person is big or small prayer is performed, as the child has not done any sin, he is pure from sin.
7.1 What was the reason for your child's death and what is your perception about the value of knowing the cause? (Investigation: knowledge about the cause of death, a desire to know the cause of death, potential factors that cause illness / death, possible effects on other family members or the next pregnancy)
Father- The reason was already known, they told reason in starting only, and told only 1% chance is there for survival, and they were trying to save. It is important to know the reason, we did not ask the doctor for the reason, due to which he died.
7.3 Are you aware of any method to identify the exact cause of death?
Father- We does not know about post mortem, this is done in accident. We can know the reason after talking to doctor.
Mama- Yes, we know about post mortem, organ donation is done, it is done after accident, from government side, it is that they take kidney and seal the body later. In today time, no attention is given to post mortem. There is no post mortem for natural death. If the person suffered from fever whether he was younger or older, the ritual is done, if the person died due to accident or murder, then postmortem is done. The report is then checked to know, who killed, how did he kill, whose finger print are there. The children who died in hospital, there post mortem is not necessary. When someone complaints to the police, when

there is doubt whether the person died himself or someone killed by murder, then it is sent for post mortem, or the police take itself.

7.4 What are your thoughts about autopsy / post-mortem to identify the cause of death?

Mama- In our religion post mortem is not done, we don't like that our near and dear ones should undergo tearing process. And if it is necessary, then centre government says post mortem is done for accident or murder, to check how did it happen. If you perform post mortem then you will know the reason of death. But we do not like that the person who is known to us should undergo post mortem a everything is removed in it.

Nani- Everything is taken out in post mortem, whether it is good bone it is taken out, and we also don't know who is the person/what is his caste who will touch the dead body, we do not want that wrong person hands should be put on our known person.

Mama- In accident or murder, post mortem is necessary, if the police got to know then even if we try to stop then also they will do. Police can take the dead out of cemetery if it is needed, they check report how the murder was done.

Father- It is not mentioned anywhere in our religion that post mortem cannot be done.

7.5 What are your thoughts about the collection of tissue and body fluid samples to identify the cause of death?

Mama- This technique is right and good, it is good then tearing, the body which have undergone post mortem nobody likes to see it. I have watched post mortem 3-4days back on YouTube, I did not like to watch it, they did post mortem after consuming alcohol. I have also read about post mortem in newspaper two-three months back. If this technique (tissue sampling) is done then it is good, it will be performed by machine. After doing this all rituals performed will remain same.

7.6 How can parents and family members be contacted for tissue sampling?

Mama- About this technique, if the doctor would tell to parents that child has died and they should now allow them to do this technique, then parents will get to know how the child died.

Mama- How much time is need to perform this technique? If it will consume less time then nobody will deny, if it will take time, after child death then family prepare to take the body. At government hospital these procedures take time. Our child was small, otherwise in government hospital a lot of time is taken. If doctor would tell about this new technique, that blood will be taken to tell us about the reason of the death, then nobody will deny.

<family approach>

Mama- We will try to tell anyone who will encounter this problem in front of us.

Nani- At that time to tell parents will not be the right thing as they are already in grief, so they can tell us about this.

Mama- Whoever is wise, you can tell them, if doctor will tell about this technique then no one will deny. We want to know how the child died, the reports would come, we will see the report, in today's time the report comes in the form of film, in post mortem these only things are revealed.

7.7 In your view, what factors are important for the acceptance of tissue samples by parents and family members?

Mama- Parents should be told comfortably about this thing (tissue sampling). Now the death has already happened, doctor checked, in todays time nobody wants to see the death of the child, as it has happened to us, doctor is engaged in their work as there are many children, there were 15 children in the ward where our child was kept. We liked the technique (tissue sampling), but the doctors can only tell whether blood will be taken or what will be done with syringe.

<any change in hospital>

Mama- More and more advertisements should be done, in hospital many things are written on the wall in Hindi and English. For this technique someone should be present to tell. Other than that, you and other officials can use their mind.

8.1 In your view, what can be done to improve children's efforts to reduce deaths and to identify the causes of death?

Mama- The doctor who is treating child for 4-5 days and if he has done some mistake then they will not try to do this procedure, as by doing this they will fear that their mistake will be known to the family and also, they would be suspended. The child has died because of their mistake as they didn’t change his tube. Now report will tell about the cause, so they will be suspended, so they will not try to tell about procedure. Now this technique (MITS) is in your hands that parents should know about this procedure.
Nani- They were telling about the condition of our child, we our not telling anyone mistake.
Nani- Tell me what to do next, since then my daughter's health is bad. Her health is not improving, she is still in the shock, that why I have brought her here in my house. Now what has happened, it was only Allah's will.

4.2 What was your and your family member’s reaction to the death of your child?
my wife didnt came to hospital, she was at home. i was there at hospital, doctor checked the baby infront of me. doctor said there is no movement of the baby, at that time i felt sad and felt sorry at that time.

5.3 Is/there any change in the interpersonal relationship between you and your spouse and other family members after the event
There is no effect in the relationship, just a little bit, now the wife is worried because of our child. I explain her not to worry. The baby was our 1st child, so we remember the child, sometimes she is also in shock it will take some time.

5.7 when did father/mother returned to work after the death of the child?
i returned to job after 15 days, my wife started household work after 40 days. Now she is little disturbed, but now she is cooking for the family.

5.8 How you and your family life changed after the death of the child?
Two or four things have changed since then, everyone says that there will be no other problem, do not take tension and the time for eating and drinking has changed. Previously we used to eat in the morning, now there is no fixed time. I do not feel like eating and drinking after that incident. So far, I do not feel hungry very much, now I have thought of the child further, but my wife is not in condition, she had swollen, now I am showing her to the doctor.

7.1 In routine day to day operation who takes decision about the major activities about the family?
for routine work decision are taken by my father. for other workdecision is taken by my paternal uncle.

7.2 During the hospital stay who discussed with doctors/nurses to make the decision about treatment and other procedures
when child was in hospital all decisions were taken by my father-in-law.

7.3 After death of your child, who decided about the cremation and other post-death activities and rituals
At that time my father and my brother-in-law was there, they were telling all things.

In-depth Interview parents of deceased neonate-2

**1.1**	**Primary respondent (relation with the child)**	Mother, Father
**1.2**	**Age of the primary respondent**	Mother- 25-30 years<br>Father-3-35 years
**1.3**	**Other members present**	None

Introduction
We are living with three members; mine, my wife and my daughter.
2.1 Where do you usually go or who do you consult during your child's illness?
Usually we go to MN hospital, but in serious case prefer to go S Hospital. My daughter was born at S Hospital.
2.2 Where do you usually go or who do you consult to vaccinate your child?
For immunization, we go to a dispensary at W-12, SAINIK FARM. We had plan last delivery at a private hospital as people says Govt. hospitals are not providing good services. I was scared from govt Hospital.
2.3 People in your area / community are generally asked for their children's illness?
Poor people usually prefer Dispensary for small disease. That is a govt. dispensary and cheap. But in case of serious disease, people go to govt. hospital, because we cannot afford costly treatment.
3.1 Can you please tell us about the illness and events that caused <name> your child to die?
Initially, baby was suffering cold and cough. After that, my sister said, he (Baby) might be infected and went for advice to a known Doctor at MN hospital. At there, Doctor gave a nasal drop and recalled after two days. Again we went after two days, doctor said, baby has breathing problem, Baby has not kidney, Heart and told about a lot of disease. After giving a nasal drop, doctor recall after two days. On next appointment, doctor declare the case as a serious and drop us with her car after insert a pipe (for breathing) into the baby nose. Then we went to S Hospital, at there, doctor refuse to admit. They said that we don't have machine to manage this case, at same time they said, this baby is about to die. The hands and legs of baby was shivering at that time. At the time when my baby was at MN hospital, he was good and that doctor help us a lot as she refers in their own vehicle. A lady doctor (Jr Doctor) from MN hospital, had come with us to S Hospital for admission. She put (lay down) my baby with earlier admitted child (3-4). In hospital ward, I called for emergency help but no one came to see my child. At same day, in midnight around 12- 1 o'clock, my baby was dead. They discharge us at morning 6 o'clock. No one at hospital care us, everyone was busy on their phone.
3.2 Who supported you during illness and your child's hospitalization?
We went hospital with wife only, later on Mother-in-Law and sister-in-Law came at hospital for help.
4.1 Now you are requested to tell about what should happen when your child is admitted in the hospital? (Investigation: hospital, ward, length of stay, course of illness)
Mother: There was a big trolley on which, around 4-6 children were kept. All together moves to next (top) floor for treatment. All children were fitted with an automatic breathing machine but mine (children) has a manual breathing machine, I had to push that manually for maintain baby breathing. We kept pushing the machine overnight for same. It was continued from 8pm to 6am. At morning someone (Doctor) told us, this cylinder (breathing equipment) had last at earlier means there was no gas (Oxygen) in that. No one informed us about this. We wasted our time to push an empty cylinder overnight which has no gas (Oxygen). Everyone was engaged in their phone.
4.2 Can you describe your overall experience of hospital care? (Investigation: investigation, treatment delivered, satisfaction level, quality of care, cost of care)
There was not any investigation had been performed at S Hospital. Prior to that, on MN hospital all investigations had been done. It was X-Ray, Ultrasound and all blood test. At S Hospital, they only gave injections (1-2) over whole admission period at name of treatment.
Prior to admission, we bought Pipe and 2-3 medicines that were wasted later on. They (hospital Staff) demand money for a machine (Breathing tool), but we did not have money at that time. We spent all our savings even at time of delivery. We request them to use the machine and give some time for payment but they demand money before. We were unable to instantly arrange the money at night time (10pm-11pm).

4.3 Can you describe your experience about communication by various hospital staff during your hospital stay? (Investigation: everyone communicated, which was the primary communicator, nurse, doctor, other staff, frequency of communication)

It was good but Doctor was very rude. Nurse was doing their work properly but doctor did not come to ward just even for a walk.

No one detail/brief about what had happen. Nurse only inject 1-2 injections and glucose. It was frothing mouth and many difficulties had happened at that time. No one was looking for manage frothing mouth. Lastly I use to clean my baby mouth at regular interval. It was supposed to be cleaned by machine but in our case we were doing same.

There was one person as caretaker of all at our ward (Hospital). I hardly remember whether she was a nurse or doctor. He visits the ward only on call. His advice only to push the breathing equipment and concentrate on that. The person who had come with us from MN Hospital to admit, told that, make a OPD card of S hospital and you will get machine (Breathing machine). The hospital staff refuse to provide that machine even in front of all. He demands 12000 to get that machine.

Usually, A junior doctor updates the status of my child. Senior doctor told us, your baby required machine, get it soon. We said our problem that we cannot afford that. He said all available machine engaged in earlier admitted patient, its already scarcity of this machine. We give it to serious case only. We continue request that our case was also critical please provide us a machine.

We reached hospital around 7-8 pm and that person from MN hospital made an OPD card of my baby. She told us that you get machine after the OPD card but that not happened.

5.1 Can you please tell us about your child's death and death declaration? (Investigation: Who had announced the death, body language and expression prior to the events before death)

Mother: Can you please tell us about your child's death and death declaration? (Investigation: Who had announced the death, body language and expression prior to the events before death)

We told that our baby is no more, he died at mid night but no one listens me.

After our repeated request, a senior doctor came to see our baby. No one told us about the situation. Even after knowing that baby was dead at midnight, I could not understand why they were not released us from hospital. Lastly at morning, they discharge and declare dead.

5.2 Do you blame someone / something for the death of your child? (Probe: Who or what, what did you do in this context)

Mother: Yes, I would like to blame doctor who told that your child will dead even before starting the treatment. They advised machine for baby but not issue same. We tried to get the services from hospital but not got. We fought from a lady staff for service. She rudely behaves with us. Earlier my child was absolutely right but don't know what happened after.

5.3 How was your experience about the process after death until the operation of the body? (Investigation: procedures, documents and papers given, respect for religious norms, time taken, cost / payment, assistance from staff)

They gave me a slip we left all things like cloth etc. at there and came home with baby. It took 2 and half hours. They told to collect death certificate after 1- 1.5 month.

Junior Doctors care a bit more as compare to seniors. The senior doctor came at once and instruct to push the manual (breathing) machine in 1, 2, 3 sequence. After that they did not came. A lady with that senior doctor repeats the instruction.

5.4 Who supported your family and everyone in the community / neighbourhood during this period? (Investigation: who informed them, who all came, what kind of support was given)

We went hospital with wife only, later on Mother-in-Law and sister-in-Law came at hospital for help.

6.1 What procedures did you follow after your child's death? (Investigation: Preparation of body, which prepared, burial / cremation, other rituals and time)

(After death declare) We came to home by an auto. We totally lost about what was happening with us. We admit our child just 2 days back in a good condition. At MN hospital, they told it was just cough and will get well soon. And suddenly, they told us about lot of disease (kidney, liver). As he told baby has not kidney and liver but it was not informed at the time of birth. Whenever the hospital where birth had happened was a good hospital, it's VB Hospital near M hospital.

6.2 How did you, your wife and family members face a difficult phase?

We lost our job because of this. Now, we are unemployed and not got another job because of tension. We are not able to do work

6.3 How did family members support you and your wife during difficult times? (Investigation: burial / cremation rituals, community norms / rituals, phase, coping with financials)

I was there with my mother and since I am new to this area, we took the dead body to mother's area. A gays residing at top floor is an auto driver, was also with us. A crematorium is there. We went there. In case of children, we burial the body rather than funeral. Yes, first bath the body and wrapping the dead body, etc. are the process. We were done Havan (offering prayers to god in front of fire) after one week of death, same was done at birth time also. A prayer on the name of child was also happened, in which cloth, flowers, rosary has to be offer to god. Wife did not go at cremation place. Around 10-12 people were present there, who belongs to my and my wife's family. Some people was my friend

6.4 What were the responses / counseling and suggestions given by the relatives / neighbors / community after the episode? (Investigation: what to do after death, observing rituals, finding out the cause of death, someone else)

Mother told that why you went there, these all were happening because of doctors' ignorance. Mother knows everything, she informed us immediate after death but we had not realized at that time. We had hope for their life.

7.1 What was the reason for your child's death and what is your perception about the value of knowing the cause? (Investigation: knowledge about the cause of death, a desire to know the cause of death, potential factors that cause illness / death, possible effects on other family members or the next pregnancy)

Many people told us to know the cause of death but we don't have time. In an incidence, where my sister lives, a doctor cut the breathing nerve of a child and go off from there silently, parent though that baby was unconscious but actually he was dead. It all had happened because of S Hospital. We should not go there for treatment. We should admit baby in private hospital even on the cost of our land of village (Asset). We went because my first baby born there. We are regretting from our decision to move S hospital. We had gone there just for treating a cold and cough and next day we lost him. That was only son of my family. Now we have a daughter only.

We knew the death when the doctor told to stop pushing the machine. They should be responsible for medicine and treatment. Doctor of MN Hospital told them my baby will get well soon, in S hospital, they will keep the baby in ICU, provide ventilator machine because manually it's not feasible and he was suffering from pneumonia. Here at S Hospital, he refused to admit baby in ICU, as Machine was not available. They clearly told that you can go somewhere else, but we do not have other option. We did not have money to treat them in private hospital. It was impossible to arrange money within overnight. We request him to provide ventilator for baby we will paid you next day morning for same. But no one cares. Once a while, they put pipe into baby nose for ventilator but remove immediately. Doctor came to ward only once during whole admission time. A nurse kept my baby under a small bed (SNCU) but senior doctor scold that nurse and ask security guard to keep my baby outside from bed. Doctor taunt us also that they don't know care of her own baby but always keep reproducing baby and make ill them.

7.2 In your view, what else could be done to find out the exact cause of death and the cause of the disease? (Probe: probe, corpse, any other)

Mother: They do not talk about cause of death. They do everything in solo mode and always try to avert us. In case of any query, they become loud and rude. Once I told to doctor, see my baby what is happening with his breathing, he (Doctor) answer, I will give an injection and will cure. After that, doctor did not come to see. Doctors of MN Hospital (Private) were better than here. They did full check-up and respond well. He said we don't have ventilator so you need to go S hospital. Earlier private hospital was must better than S Hospital.

7.3 Are you aware of any method to identify the exact cause of death? (Investigations: bodies, investigations, etc.)

Mother: Reason was that doctor not treats well our baby and insert pipe in mouth. That's why our baby is no more.

Don't know much about this. Yes, I have heard about postmortem that was conducted in my wife's dad case. That was happened in S Hospital in an incidence where someone gave a wrong medicine to him. As I remember from last incidence, postmortem identify the reason of death. A neighbor whose relative is in police department, ask me about postmortem of baby. He told me that you should do it

for stop this type of incidence amongst other. But my mom refuse, she told baby is no more so there is no point to do postmortem.

7.5 What are your thoughts about the collection of tissue and body fluid samples to identify the cause of death? (Investigation: need, religious aspect, need of time, disfigurement, etc.)

How is it possible, it should be done on same time? One fellow, who is driver in American embassy (Delhi) told me about postmortem and also ask that we should knew the reason of death. A wrong treatment had happened with their wife also at their delivery time in S Hospital. Then his owner (for which he drives) shift her wife to a private hospital that costs approx. 25 Lakhs. He was totally upset even his child was dead at that time.

There are no any changes as per our rituals. At the time of Dad's postmortem, they (hospital staff) extract everything and told us the reason of death. Some gave poison to him. At that case there was not any mistake from doctor side. Cremation ritual are same in all case. In case of young patient, they (hospital staff) extract some body parts and use it for others.

7.6 How can parents and family members be contacted for tissue sampling? (Investigation: who, when and how to approach and for whom)

I don't know about this but it's good. Next time it could be followed. In our case, we are totally lost/absent mind even we left all goods like mobile at hospital at death time. We can tell others about MITS. Not to Mother, Father but others like brother can help to proceed this. It's better than postmortem because less cuts and surgery will require in this. People will sure accept this method.

7.7 In your view, what factors are important for the acceptance of tissue samples by parents and family members? (Investigation: desire to know reason, trust, confidence on health care provider)

Some people afraid from this, but knowing reason of death is must. In our case we were very tense but people were emphasizing to do postmortem. Even they came at front for help during postmortem. Recently I went to my native place it's been just 2 days to come here. I am based out from Uttarakhand. We usually thought that why we will do this after death. If the doctor care well, talk well, treat well and still could not save the life then we could convince for their advice (MITS). If they would talk well no one refuse their advice. Even in our case if they ask for MITS we would go for same. Our baby was born at 1st of last month and dead at 31st

8.1 In your view, what can be done to improve children's efforts to reduce deaths and to identify the causes of death?

We don't know in details, but that death was because of ignorance, if they care well, admit my child in ICU in timely manner, provide ventilator machine, they could save my baby. Earlier, in MN hospital they diagnose this as Pneumonia so keeping that in mind at S Hospital, they should admit it in ICU but they didn't.

Updated (mother)

4.2 What was your and your family member's reaction to the death of your child?

at that time we were worried, that our child has gone, doctor showed carelessness towards child, we were angried at doctor, we didnt express our anger we were worried, we were not able to tell anything.

5.3 Is/there any change in the interpersonal relationship between you and your spouse and other family members after the event

my husband remains upset, at that time he even lost his job. earlier everyone was happy now we fight more, he says due to my carelessness we lost our child, you didnt take the child to doctor, you did this, now we fight alot. now also we remain upset, we have three year old daughter we are not even able to pay attention to her. We don't have anyone in our family who can sit with us.

5.8 How your and your family life changed after the death of the child?

We all cry every day and are worried that we think why we went to that hospital, we should not have taken our child their baby would have been fine if we had taken it elsewhere. My husband is just very upset, he does not even eat food, he has become very weak nowadays he was admitted in the hospital too he became very ill. To reduce the tension he goes to work and then he remain fine. Sometimes he don't feel good at work but what to do, you have to do it. He has anger for doctor, that they have killed my child. He doesn't drink or smoke.

7.1 In routine day to day operation who takes decision about the major activities about the family?

All decision are taken by me and my husband. my mother-in-law is not there. if some decision in which we dont understand then we ask my mother.

7.2 During the hospital stay who discussed with doctors/nurses to make the decision about treatment and other procedures

We did not think anything in the hospital, we were listening to what the doctors were saying, we did what the doctors said, the child was very serious, so we did not understand anything, what should we do and what not to do.

7.3 After death of your child, who decided about the cremation and other post-death activities and rituals

About cremation, my mother told us how to do and what to do.

In-depth Interview parents of deceased neonate-3

**1.1**	**Primary respondent (relation with the child)**	Father
**1.2**	**Age of the primary respondent**	Mother- 25-30 years<br>Father-30-35 years
**1.3**	**Other members present**	Father's brother

2.1 Where do you usually go or who do you consult during your child's illness?
A doctor is residing nearby area; we went usually there and take medicine from a dispensary that is a bit away. The doctor next door has their own private clinic.
2.2 Where do you usually go or who do you consult to vaccinate your child?
We go to dispensary for immunisation.
2.3 People in your area / community are generally asked for their children's illness?
A doctor is residing nearby area; we went usually there and take medicine from a dispensary that is a bit away. The doctor next door has their own private clinic.
2.4 Mention the reasons for priority for the facility and / or healthcare provider.
We usually go to dispensary for Immunisation. Govt. hospitals have more negligence; they are not responding in a good manner and hospitals are also not good they are like fake.
3.1 Can you please tell us about the illness and events that caused <name> your child to die?
Baby had jaundice. First we took to HF hospital (Private), At there a Doctor (M) had done nothing and just told that condition was very critical. As they had not machine for jaundice treatment, advice to refer at other hospital. Again we went to C hospital, they also refer so lastly we went to place where baby born that was S Hospital.
3.2 Who supported you during illness and your child's hospitalization?
My brother-in-law.
4.1 Now you are requested to tell about what should happen when your child is admitted in the hospital? (Investigation: hospital, ward, length of stay, course of illness)
Father was there till four days. Mother was for more than three days. At that time, Baby was eleven days old. We did not know where doctor was doing right treatment or wrong but they told about good treatment. They usually not tell the health condition of baby, told only when we asked.
<Ward> Otherwise, it was good. After new govt; many improvisation has witnessed, administration going to strict. Earlier, condition was worst. Since it was a preterm baby, they did not put him under machine. I had not seen any treatment procedure with my baby since admission. Initially, they told that we will keep it in machine but not did so. In the name of treatment, they only give an injection and told to come at 10 o'clock. After that, when we went at 10 o'clock, they asked '' is baby breast feeding''. I replied ''yes''. I replied all what they ask but they did not even check. It was about four days stay at hospital. They told ''what is blood group'' but our reports were lost, what we had. Did not know, where it was missed, either at dispensary, earlier hospital or somewhere else. They (hospital staff) are very irresponsible. We had handover all lab report to hospital but they missed. We got upset for report. In the wake of that, they were not discharging baby.
4.2 Can you describe your overall experience of hospital care? (Investigation: investigation, treatment delivered, satisfaction level, quality of care, cost of care)
We had performed blood test. They suck blood a lot. They did it themselves
At time of birth, they demanded Rs. 60. We did not had change so gave Rs 100, even they did not return rest amount.
4.3 Can you describe your experience about communication by various hospital staff during your hospital stay? (Investigation: everyone communicated, which was the primary communicator, nurse, doctor, other staff, frequency of communication)
Doctor had already started treatment; they were doing right or wrong don't know! Its only they know!
There was ignorance at their level. They came at ward only in emergency, otherwise not. When the baby salivating more in their worse condition, only after that nurse came and advice corrective measure like wipe baby's mouth. Lot of ignorance there.

When doctors came at round, they started to scold us. I was worried about baby condition, why was baby discharging saliva at every time, why his breathing rate was high. Staff had dull response on all of this concern. I used to speak them that baby was serious look at him.

Security guard not permit more than one attendant in ward, as they told only person with patient will go inside. Doctor every time pissed off on us and told why you people repeatedly ups and down the room. Some time when they need us, call us loudly from inside. We were totally confused where we sat. The patient beside us was also scold by doctors even more than us. Govt. doctors are more irresponsible, even they are earning more than their salary so they have very much attitude. When baby born, they took money but it was good as every things going to be good. Each time doctor showed their irresponsibility. We were urging every time to see our patient but they did not. On a moment, baby was facing seizure but doctor not done anything more than infusing glucose and blood test. Unfortunately, blood report was missed.

5.1 Can you please tell us about your child's death and death declaration? (Investigation: Who had announced the death, body language and expression prior to the events before death?)

My dad was there. For pushing a pump (manual ventilator), at any time any of us had to be there (at hospital). We had sense when baby stopped breathing. All of us knew about death, when doctor said that call her father. They forcefully push the ventilator pump. Nothing was clear, whether he breathe or not.

Doctor told to brother "call baby's father''. When I reached to hospital. I saw all doctors involved in our case and remove all support devices (Pipe). They didn't say that baby was dead. Didn't say to me about anything. At time of death, baby was not suffering from jaundice. Earlier doctor said '' baby not having jaundice''

They did not tell the reason of death. Did not say anything. At time of death just told ''call her father''. Around 11 o'clock, when I reached at hospital, baby breath and pumping was continued for one and half hour. After that I called doctor, they came and give an injection that was not effective and again after 5 min they told call her father. Baby was dead a little before that.

All the doctors coming to me came to force me, started pumping, started pressing and did nothing before (father). The doctor did not say anything. He (the husband) called the doctor there is a lot of breath in between (brother). Once, the doctor does not tell why the breath is moving fast, what is happening, they just told that the skin was full (mother). Did not keep asking (father)

In case of ask to the nurse, she keep screaming, and stop or see the other (brother). Come, she starts shouting like this, that is fine but negligence is there a little over there (brother). Neither is negligence but it is fine (brother).

5.3 How was your experience about the process after death until the operation of the body? (Investigation: procedures, documents and papers given, respect for religious norms, time taken, cost / payment, assistance from staff)

That was done instantly. All procedure was completed by them and frees us within 15 min. we came by our own car. That was so called so big hospital and when we need machine for baby that was not there. They baby got machine, he could be cured. It was told at the time of birth that it was a week baby.

5.4 Who supported your family and everyone in the community / neighborhood during this period? (Investigation: who informed them, who all came, what kind of support was given)

Relative supported us at that time. There was some ignorance at the hospital. We, around 8 people were present at hospital at any time.

6.1 What procedures did you follow after your child's death? (Investigation: Preparation of body, which prepared burial / cremation, other rituals and time)

We went to crematorium at around 8-9 o'clock mornings and buried. Lot of people were present except ladies. Brother-in-law prepares the dead body although we knew all the procedure.

6.2 How did you, your wife and family members face a difficult phase?

At that day, we went crematorium and called priest for worship and repeat worship after 4th days of death. That was pray for piece of soul. Its cured by time. Gradually we cop up and started our routine work. All should focus ahead. At that time all relatives were present.

7.1 What was the reason for your child's death and what is your perception about the value of knowing the cause? (Investigation: knowledge about the cause of death, a desire to know the

cause of death, potential factors that cause illness / death, possible effects on other family members or the next pregnancy)

They did not detail about condition. Like said ‘’see saline is infused, this is status of body’’ that’s it. Did not talk politely. Not talk with sympathy. My condition became worst by time as by standing in long que of hospital. We went there at morning but got number in evening.

7.2 In your view, what else could be done to find out the exact cause of death and the cause of the disease? (Probe: probe, corpse, any other)

We had not done post mortem. It depends on person. Through this, one can get exact reason of death. There are some benefit and loss of that as well. Loss is that ‘’ it is conducted through more cuts and surgery so people do not want to cut the dead body after death.

7.4 What are your thoughts about autopsy / post-mortem to identify the cause of death? (Investigation: need, religious aspect, need of time, disfigurement, etc.)

We needed to know the reason but doctor did not tell us (father). Nothing can be done after death (mother).

It is important. At least one should know the exact cause of death. They are not even talk in polite way. Do not talk.

7.5 What are your thoughts about the collection of tissue and body fluid samples to identify the cause of death? (Investigation: need, religious aspect, need of time, disfigurement, etc.)

This is a good technique. Doctor should pitch this otherwise they will say ‘’are you doctor or me’’

7.6 How can parents and family members be contacted for tissue sampling? (Investigation: who, when and how to approach and for whom)

Mother or Sister would be right person for this. We would must welcome the technique what you told (MITS).

One accept this, if was told by doctor. All staff at there was talked very rudely. Many of us were victim of that. But we all were helpless. In case of any request to staff, they call the security guard. There was a case beside our bed, was left the hospital because of ignorance. The hospital staff told them ‘’this type of service you will expect in low cost’’. If we have money, we must go to a good hospital. We have not, that’s why we came here. We can’t afford must we cannot shell out house and shop, otherwise how we will manage other family member. We had no other option. One should compromise quality in low budget. Doctors were overloaded. Space in SNCU was also less. In one SNU bed, there were three children. Later on, when my baby needed more space, they remove another from there.

7.7 In your view, what factors are important for the acceptance of tissue samples by parents and family members? (Investigation: desire to know reason, trust, confidence on health care provider)

At that time, it’s impossible to understand about post-mortem or MITS. It’s hardly to notice, what’s going on. As in our case, how time disappeared, we did not notice. Once Doctor told about death, we came directly home. They said themselves to take baby to home otherwise they made a big issue of this (Discharge). They called baby’s father after death in hospital and suddenly all doctors came to see, they did not come all together before death even at once.

Told; sorry we cannot do anything. They had dubious role, in one side they told “No hope in this, call her father’’ and another side when they saw us started to pump baby like they were tried to do something for save life. They always keep patient roam around old and new building of hospital. Even, for the lab test, we need to give sample to lab. The person at lab again asks to go somewhere else to keep the sample.

8.1 In your view, what can be done to improve children's efforts to reduce deaths and to identify the causes of death?

No one wants to do post-mortem but technique (MITS) what you told is good. Doctor should have told about this, about do’s and don’ts’. Usually, they do not tell anything. If they start to talk, everyone will listen. We will also follow if they teach.

In-depth Interview parents of deceased neonate-4

**1.1**	**Primary respondent (relation with the child)**	Mother, Father
**1.2**	**Age of the primary respondent**	Mother-30-35 years<br>Father-30-35 years
**1.3**	**Other members present during the interaction**	Nani, Brother

Context- The mother delivered her baby in 7th month the delivery took place in S hospital. The baby after delivery was admitted in nursery then he was discharged after few days. The mother of the child is presently staying with her parents.

Events that led to death of the child

2. Can you please let us know about the illness and events that led to death of your child?

He had cold in the beginning, the child was sneezing, and his voice was not coming, meaning he had a cold. Then we took him to the doctor, in emergency. The child was admitted in nursery at S hospital after the delivery, then after 6 days he was discharged. When after 6 days we came home, on 15th day I bathed the child, due to which he got cold. Then we took him to the hospital and they said that the child is drinking milk he is fine, they didn't admit the child on Tuesday, then on Wednesday we again took him as his condition got worsened. On Wednesday we took him to OPD, from there they referred us to ward 18. Then we went there, they did not see anything, just inserted the tube in his mouth and nose, 3-4 days the baby was fine, then on 5th day when the third tube was replaced it got hurt in the baby's lips and mouth, then to the chest and to lungs. He stopped breathing and was dead.

Maternal grandmother- When we stayed in the hospital for five days, all the children were put on Bag and tube, their parents used to hold that, they were not put on oxygen as staff will have to visit again and again.

Mother- when we go to doctor then they used to say that why are you roaming after us, we are seeing patients, i said baby has problem, then she used to scold me.

Maternal grandmother- We saw how many children were dying there. Not a single child returned safe from ward number 18, the child who has gone there means that 5-6 children were admitted and died in a day, there blood was withdrawn.

Hospitalization period

3. Now we would request you to tell us something about the period of hospitalization for your child?

We stayed in the hospital for five days, on the fifth day the child died. We was born on 23rd and died on 23rd after one month.

<about hospitalisation>

I had gone to the OPD on Tuesday, they said that the child is fine, then we returned home, then on Wednesday we again took him and got him admitted. From Wednesday we stayed for 5 days in hospital, at 4 his heart stop beating. The child was fine, the child had no disease. He was coughing and having cold, so we took him, he was also urinating frequently. He was not able to cough properly as the mucus was accumulated in chest.

I and my husband and my mother used to go to hospital, me and my husband used to stay in hospital for pumping bag and tube.

4. Could you please describe your overall experience about the hospital care?

When I call the doctor, she did nothing, she just changed the tube. When senior doctor used to come they tell us that child is fine, you have to take care of the child, when they left then nurse showed carelessness. Other doctor used to talk among themselves when we go to them and they scold us.

<frequency>

Senior doctor came at 11 in the morning after breakfast they tell us about child condition, they came once in a day. One senior doctor came with two junior doctors. They used to tell that give medicine, take care of it properly, the child started recovering. Then doctor showed bit careless.

The baby was born in 7 months and they used to drain his blood 3-4 times daily from morning till evening. In five days they had withdrawn so much blood, from hands and feet, the child started becoming blue. He was 12 grams less than 2 kg. They used to take 2 tests, the third test was normal

everything has come normal, if they haven't put the third tube in the last, nothing would have happen to the child.
Maternal grandmother- the tube hurt the child on lips because of which he was not able to cry and he was bleeding.
Mother- we didnt buy any medicine from outside,when we returned home from nusery earlier from hospital we got calcium medicine, then child was fine.
Maternal grandmother- When child was lying in bed he used to move, he doesnt take any covering of cloth over him, he was active and fine and normal.
Mother- after i conceived, after one and half month after conception in february, something stopped blood started coming, i visited private doctor, she gave me some injection, i thought i had a miscarriage but the child survived, this was that child only.
Maternal grandmother- Everyone is saying that 7 months baby is weak but he was fine.
Mother- <cost> in S hospital we didnt spent money as test was conducted in new building. two test were done outside, cost of one test was Rs 60 and cost of other test was Rs 100.
Maternal grandmother- one ultrasound was condcted in private during pregnancy.
5. Could you please describe your experience about communication by the various hospital staffs during hospital stay?
Mother- Nurse was fine, she used to listen to us at once. doctor used to scold us and everyone. she said dont roam behind me.
Death and post death period
6. Could you please tell us about the death and death declaration of your child?
Mother- Senior doctor said child is serious after seeing the child. during admission also they said child is serious, they said child is gone, i said when the child is breathing how he can die, i brought the child here in better position, they fooled us and inserted the tube. he was suffering from cough they would have given oxygen and child would have become better. they inserted the tube on very 1st day. <who declared>the senior doctor told child is serious, and there is no chance of survival. in morning i came back home, my husband was there in hospital, child cried and tube came out, my husband told doctor that tube has came out, then doctor inserted the tube through nose and mouth, it started bleeding, then after 4 his heart stop beating and he died.
Maternal grandmother- doctor came and saw the child, my child was lying with other two children and tube was there. the needle which was inserted in his hand to give glucose came out and there was blood all over even over his blanket.
Mother- When I went to the doctor and said that blood is coming out, then doctor said to bring cotton. I said to them to come and see my child once.
<cause of death>
They said child is serious and have double pneumonia, I said earlier he was not having pneumonia, in last doctor said child is having pneumonia.
<behaviour>
senior doctor was good, he said to take care of the child, when senior doctor used to leave then other doctor showed careless behaviour, they used to laugh among themselves, when we go to ask for something then they scold us. Only doctor who used to come and see the child was good. Also nurse was fine.
maternal grandmother- one child father slept during pumping the bag and tube, the staff came started scolding him that your baby is dying in life and you are not looking after him. the person get tried by constantly pumping bag and tube, there should be proper oxygen pump for children.
Mother- senior doctor was fine, other doctor used to scold, 1st they said they want blood donor, then i called my brother for blood donation, they said to him to sit and wait, my brother came at 12 at night, after that he said him to sit and wait because they didnt have time now, when we will be free we will call you. we dont know what they did of blood taken. for taking blood they said to sign the paper, and started scolding my brother then my brother also got angered that you have called me at night for blood donation and you are not taking blood now.
7. What was your and your family member reaction to the death of your child?
Mother- this all happened on sunday evening at 4. when all this happened i was with my husband, mother, sister-in-law, my brother went back after donating blood. my brother came when baby was

dead. doctor told my husband that your child is serious, they called me but sent me out because i was crying as baby died. after that they handed child to us and we returned back home.

8. How was your experience about the procedure after death till handover of the body to you?

It took 20 minutes after the baby was over, we had left soon. We just signed and they gave child to us. Family member left at 4 after taking the child. We did not bring the child home, my husband and brother took him straight to burial place from the hospital.

9. Who all from your family and community/neighbourhood were present at the hospital and how they supported you during this period?

Maternal grandmother- i and my dughter was there. My girl was crying and screaming and she was supporting her and I was crying myself. No neighbour and relatives came now, they came when child was born.

Post death procedure and coping mechanism

10. What all rituals and procedures did you follow after death of your child?

Mother- Money was paid at burial place. Child was directly taken from hospital, we didnt brought the child home. My husband, brother and his some friends went for burial, i was at home, i didnt go.

Brother- From the hospital we went near Green Park for burial of child, they took Rs 1500 for burial.

<who performed the rituals>

The child's father did. The pandit digged the ground, put on clothes, put some salt, performed some prayer, and then child was buried.

11. How did you, your spouse (wife/husband) and immediate family members cope with the difficult phase?

Mother- My husband supported me during that time, he said there is no use of crying now.

Maternal grandmother- She used to wake up at night and cry, she didnt properly sleep at night. child father was also very sad at that time, he supported my daughter.

12. Is/was there any change in the interpersonal relationships between you and your spouse and other family members after the event?

At that time my husband supported me, yes, he used to say, there is no use of crying. My sister-in-law also supported. When I used to cry my husband also sometimes cried along with me. He supported me, even my mother supported me. Now I keep my girl child with me especially during night to forget the memories of child.

13. How did the family members support you and your wife during the difficult phase?

My aunt used to come and explain, I used to cry, she said don't cry, it was not yours that’s why this happened it took one and half months to come out of that.

14. What were the reactions/ counselling and suggestions given by relatives/neighbours/ community after the episode?

Maternal grandmother- those who used to come used too counsel us.

15. Do you think and/or discuss with your spouse/ family member(s) about the death and related events and blame someone/something or feel guilty for the same?

I blame the doctor.

<view of family member>

they think I bated the child so all this have happened, that’s why they blame me for this.

16. When did father and mother (if working) returned to work after the death of the child?

<about father>

my husband returned to home after the dead of child as his sister-in-law also got expired. Then he returned to work.

17. How your and your family’s life has changed after the death of the child?

Mother- When i used to stay at home i always had tension, now i have joined job 10 days back, earlier also i used to work but left my job during pregnancy. it is better to work as you can earn some money even divert your mind from memories of child.

Maternal grandmother- i work in printing press of card.

Mother- my daughter has epileptic attack, she faints, we are giving her medicine from 3 years. my husband didnt smoke or drinks.

Knowing cause of death in detail- Autopsy and MITS

18. What was the cause of death of your child and what is your perception about the value of knowing the cause?
Mother- child died and we buried the child. knowing cause of death is important, doctor said he had pneumonia, we cant say anything. i dont think he was having pneunomia.
Maternal grandmother- What is the reason to know now, what has happened has occurred.
19. In your view, what more could have been done to know the exact cause of death and the reasons causing the disease?
Maternal grndmother- I do not know whether, what the doctor told me, what is right, what is wrong I do not know.
20. Are you aware of any method for identifying the exact cause of death?
Maternal grandmother- <about PM> yes i have heard of postmortem but never seen it, it happens in case of accident, suicide. i dont know whether it can take place in case of child death and we can know the cause of death. i dont have much knowledge as i am illiterate. i only visit S hospital for medicine and checkup.
21. What are your views about the autopsy/ post-mortem for identifying the cause of death?
Maternal grandmother- I do not know.
22. What are your views about collection of the tissue (like done for biopsy to diagnose diseases) and body fluid samples for identifying the cause of death?
Maternal grandmother- this is good technique if it happens, we can know the cause of death by this, in my view it is right. It is important for us to that no child should suffer. On Sundays I get worried and upset.
<religious aspect>
It is important to know the cause of death, it depends upon the person to do this or not.
23. How parents and family members can be approached for the tissue and fluid sampling?
Mother- they took out the blood many times, filled the blood in 3 bottles. nurse withdraw the blood many times, once she withdraw after 15 minutes agin she used to come and withdraw the blood.
Maternal grandmother- i can go to hospital if they call me for some thing by which we can know te cause of death, no child came out safe from that place. if they withdraw blood so many times then child is small it can affect his health, and he can become serious and turn blue.
Mother-<when> Should have spoken when the child was fine. <who told> senior doctor should explain about this. <method of explanation>they should have shown us the picture.
24. In your view, what factors are important for acceptance of the tissue sampling by the parents and family members?
What has caused the child to die.
Decision making dynamics
25. In routine day to day operation who takes decision about the major activities (purchases, treatment, spending money) about the family?
Mother- i take all decision myself, about treatment, husband used to go to work he dont know much about S hospital, even when i was pregnant all test, checkup and treatment was done here.
Maternal grandmother- she used to take care and took all decision by her known.
26. During the hospital stay who discussed with the doctors/ nurses to make the decision(s) about treatment and other procedures.
Mother- I and my husband used to bring medicines, he do not know that much. From my mother home S hospital is near as compared to my husband place. We decide on our own, we go to emergency in case we need.
Maternal grandmother- father stays far, if child was not well we used to call him so he comes direct to S hospital.
27. After death of your child, who decided about the cremation and other post-death activities and rituals?
Mother- I don't know about the rest, I had my family member and brother. In child we don't do any prayer it happens in elders, I didn't consult anyone.
Maternal grandmother- she doesnt have mother-in-law, her father-in-law and brother-in-law stays separate. i take care for her. Child medicine is brought from outside, and not from the hospital.
Summarization

28. Now, we are completing the interaction/interview. Would you like to add anything?
Mother- I have to say that this should not happen with any child from now on. The boy was in good position, boy only had fever, doctor came and put the tube on Thursday, that boy was finished. A good boy came from home, ate food, drank tea, ate bread next to him, the boy was fourteen years old. I and my husband was doing bag and tube turn by turn. The child was in the nursery, he was perfect, we brought back safe and there he was also provided with oxygen and was kept well. Ward 18 was very careless, the delivery of the child was also done in S hospital, fifteen days was fine at home, the child completed one month and then died.
Maternal grandmother- What should we say, the day we saw such a strong kid, he used to put his number first, seeing such a big boy, my mind gets upset. By doing bag and tube there is pain in hand, even regularly doing this person can sleep in between, doctor look at their own comfort, the best option is giving oxygen by machine.

In-depth Interview parents of deceased neonate-5

**1.1**	**Primary respondent (relation with the child)**	Mother, Father
**1.2**	**Age of the primary respondent**	Mother-25-30 years<br>Father-25-30 years
**1.3**	**Other members present during the interaction**	None

Mother of the child was staying at her parent's house; his husband was also present. During interview her mother and father were also present. Their two children have died at S hospital, first one died in 2016. Then after that they had 1 daughter and third child also died at S hospital.

Mother -Death in S Hospital is very high, its case is more. By the way, this time I have made a mistake, he told me that S people, because I was getting the same check-up from the beginning, I went from here, so whenever you talk about delivery, if you are pregnant. Would be fine with me okay. Father- That means whenever you have to have a child is sure to consult us.

Mother- Means you will consult me whether you are worthy of having a child or not, so I cannot speak of the lack of S Hospital, I had talked about this. Father- This time, we have no complaints in the hospital, but last time I had a lot of complaints and did a lot of wrong. Mother- Last time, when she was pregnant for two months, she had started the check-up, she had done all that, according to her decision, whatever she ate for food. As he had said to live, he even told me how to sleep, that the baby will not be turned upside down, but he told me in the last eight months that the baby does not have a spinal joint open. Is, there is water in the head, all this was told earlier in the boy, and this deficiency was first told in the boy, all this was told in the eighth month, not in the beginning. In three and a half months, in S Hospital, doctors had done ultrasound from inside, but they did not tell me what is in the report.

Father- Last time, as the doctor said, we have gone the same way, but this time we went in the last.

Mother- We had gone to the village, we are residents of Gorakhpur, when we came to know that the baby is upside down in the village, we came here for treatment, but when we got treatment, we also told about it in S Hospital. I have a large head of baby's spine, and the spine is open And do not know how long it will be alive after having a child, whether it will remain alive or not, but the child will come out alive, those people had guaranteed this thing, all of them had spoken for this child.

Mother- This time I was not mistaken when I started feeling pain when it was raining outside, and by the time the car arrived, my child was already home. Then we took our morning time there

Grandpa - It was raining so much that it rained outside all night we kept thinking that if taken outside it would get wind.

Mother- When I took her in the morning, it was said that this deficiency was asked, where is the child, so I said that the child was showing it, but the child is at home. Then they said that when they are at home, why have you brought them here, they have not treated me while keeping them at home, why should I lie, by referring them, they referred me directly to medical (A Hospital) Then when I went to medical (A Hospital), when I got time to see them, then treat them again, and gave the medicine, injection, and whatever was needed to the child. Then the child was kept till evening, then after that he said that now I do not have a seat and also do not have oxygen, I cannot keep it, that means I do not have a place in medical (A Hospital) , And said that you should move to GBP Hospital or S Hospital, first of all S Hospital was put on top. When I was going to S Hospital from the beginning, it would not be right for me to go elsewhere or somewhere, the decision was taken by me, my husband and my father, then we took the child to S Hospital itself. Then when we took the child there, those people transported me from morning till evening, where they said they went, then they sent me where the hospital is open now in the new building, after the new emergency there is SSB Surgery takes place Then when I went there, when I met the elder doctor, his name was…, he is the elder doctor of the hospital, he treated the child again, okay. And then when they see it, then they say that we cannot do any surgery for a five-day-old baby, we can do so much that we can put it on medicines, after 2-3 months, if we drink milk then it will come, then

we will Can do surgery on it and the rest of this child has no cure So then he asked me to get MRI, then see from the child's mind that even there is a shortage, then I got him MRI and gave it to him, I reached the report after five days, then he saw that the child's beating It is very small, so it cannot stand after the operation. It was said that even if we take it to Operation Theater, our hard work will also be sold and the child will not survive. My child was HOA on full time, but only after the doctor who gave time. He was not lacking in the rest of the body, everything was healthy and healthy.

Father- The doctor who had given the water and the head was all wrong.

Mother- There was no water in his head and the head was not too big, the head was small but the spine was open.

Granny-baby was like it was supposed to be, normal was the same, it was long, left, the weight was also like it was up to four kilos, it was a healthy child, it was not that weak, when expire, you left your arms and legs long, But he had to leave. There were a lot of breaks in the leg, what was lacking in the leg, when here (in the spine), there was no work, then the fingers were not moving, it was not moving. Tetanus was injected when the doctor, who is the doctor around me, told me that the baby did not cry after injecting, and then a week later, the finger started moving, both legs started moving. Then I gave the baby a kiss while massaging, then he smoothed the leg like this, then I told my girl that your baby's foot has become right

Grandpa - We are residents of Gorakhpur, there you will also know that there is a medical, but what is there is there are big drawbacks, we give medicines but there is a shortage, one is also in Lucknow - PGI, and many others Is a hospital. The operation of my brother's girl was done there, it cost him one and a half lakh rupees, in which the government had given 75 thousand, now it is fine. But what did the careless people do in S Hospital last time and this time too. There is a doctor NKD, S Hospital doctor has been there before, now he has retired

Granny- Doctor, we have to know why.

Mother- The former was in 2016, and the essence was over. And this time the baby should be 12-13 days old.

Father- This time we do not say that he has a mistake, but the first one is the fault of total, the doctor of S Hospital

Granny- My brother was married to a boy, had gone to marriage, I did not know the condition of my baby girl, I had given her the mode to go to the in-laws, we had given her a go, let's keep it for a year and a half. Have taken it, they did not think it necessary to talk to me, then when my girl came to the wedding, she told her mother that it was such a thing, when my girl came to see Me, I was leaving for Delhi. Then the quote is such a thing, then I got her ultrasound done, which is our nearest hospital, meaning there was an ultrasound done, ultrasound was done 2-3 times, water turned yellow and so on. The doctors said that the baby is upside down means that it is so bad, and then we said that this is not a big deal, the child is straight, it is reverse. , (Father- Even then, someone did not tell the deficiency, someone has something wrong in it), and did not tell the shortcomings, only this was told. If I leave it, then it is multiplied, it is not multiplied but I brought it.

Grandpa - I took it to the doctor, I was sitting in front of the computer at the time of ultrasound, the doctor saw with a stethoscope, the child's position was forward, the child's position was not getting properly, the baby was a little bit, the child was Whatever you will get, the doctor will talk, you will get what you can see, nor when the child was broken, when the child was broke, he was not able to see, only then he has no joint.

Mother- It looks like this in my boys only, my girl is very good, and she is after the first boy. When I had my first boy in the stomach, I had shown the same from the beginning, and my delivery was also the same and in the last eight months I told that whose spine has not been joined, and his head is large, He was saying that the child will be from the operation, careless care

Father - We have done a lot of carelessness while making delivery, now we are suffering the same trouble. Mother- It is a matter of time that very careless curry happened at two o'clock in the night (granny- water is not in the head of the child and after injecting so big, the water will come out if the water is coming out) When he was in the stomach, he was doing so many injections and checking if there

would be water in the spinal cord or head, there was nothing in it. Father- The boy was completely aware. He was born when he was born.
Granny - As soon as the injections were done, it was safe, as soon as the father came with it, we buried here and wherever he was injected, he had a totally different mark on the child. There is nothing visible in that period, no man can do anything except cry, the pain is painful then it is big or small, the former was just half an hour
Father- was alive for 15 minutes or half an hour, they were also taking it to get surgery, the doctors were taking it for treatment, it was there at that time, then it got expire.
Granny - So many injections were done, when I saw the child, there were signs of injection at every place, Nil was read wherever the injection was done, when there was no water, then it will come out.
<Whose decision was to take 2nd child to S Hospital > Father- The second time we did not go to S Hospital, we went to SV Hospital which was near here, took us there, our girl was born, it was clean, someone looked like someone There was no problem.
Mother- When the first child was finished, I did not go to S Hospital again, I would not lie down, and then when my girl came in my stomach, I did not get treatment in S Hospital, I have full treatment.
Father- There is a D Hospital, there is a hospital with children, all treatment is done in D Hospital, but delivery is done at three o'clock in the night, (Grandpa- is an old doctor, the government is retired). I did not think to go to the SV Hospital, then took it and then the delivery was normal, all this on the time of the other child
Granny- Doctor, as soon as I took her (2nd child), her father was in the village, I, my son and my eldest son, as soon as I took her as my granddaughter, immediately any doctor started crying. I did not come, everyone was asleep, when the baby cried, and all the eyes were filled with tears, and then said Madam, now you go out.
Mother- My girl was born normal in Shivalik, is a private hospital, and even this time (3rd child), when we came from the village, we had gone to S Hospital, thinking that she will go there, then she will be there, she will look like that. Father- Did not go there, the talent in the D Hospital, who was a doctor, was doing the treatment, then he said in the last that it will cost so much money to get it done in S Hospital itself. Then we went once or twice S Hospital, did not go again, and then started seeing these. Then they were taken when the boy was done, this is the story, whatever it is.
Granny- He said that you had to get the ultrasound done, and then went for ultrasound. Father- Then we went to get the Ultrasound done. Granny- Then went to see the report. Father- He did not enroll in S Hospital, got MRI done and got blood test done which is the new building where the blood test is done in the glass building, then he showed it after checking, then after that he said that after three months we have surgery will do By the time you take it to the house and do the dressing and bandage, if it had been admitted, the child could have survived or stayed in the hospital. Mother- I was told in S Hospital that we have our own children, who do not have oxygen for their delivery, we are putting our children on the pump and we cannot tell anything, if we want to recruit. So you keep running the pump. When I took the elder to the hospital, the second one opened In SSB, then he told me who to operate when the child is 60 days old, he wrote a vial like less, water was to be doused with glucose and he had to be wet on the child's wound and I did that I used to do this for three times by bringing here (mother place), as it used to be massaged, soaked warm water and then wiped it on her body first. Then open the bandage which was on his wound and keep the cotton cloth in Dettol and keep it so much, then he used to soak the cloth in water and keep it on his wound, and then put the tape, cotton on top. She used to put cotton on it. I used to do the same as told to me. To a nearby doctor who is a retired Doctor of Medical (A Hospital), I said that my boy is hungry, when he looked, and I showed the prescription of S Hospital, then it is said, you gave the child the infection medicine. Not now, infection has increased, wounds will open The wound will spread again and again, when they saw the leaflet, they had written only a bottle in it and had not written any medicine for infection, that means they should not have any kind of infection to the child (S Hospital doctor). Did not write then the doctor I showed them have written me an infection medicine. Then I gave the child the medicine in the morning time, like this was the time I took them to him, from there I was

fine. Then at three o'clock he had started crying, the child, then he felt so fast, then I took him (Father was -105 degrees). Then I saw that suddenly his stomach also got swollen, and it got badly.
Then me and my father, whoever sits here with private children doctors, it was Sunday time, all the doctors were closed, they were on leave, I could not find any doctors, I am telling the whole area near this. (Grandpa - everyone knows from here to Sector-30) No child doctor was open till Old Faridabad. I could not find any doctor with children. Then I brought her to S Hospital in the last. It took me half an hour to go At three o'clock my Babu was hungry, I reached S Hospital at 8-8.30 in the night, because when my child said to take me home, keep me at home, then he just kept I had given a medicine of glucose water, and then when my child was hungry, I took S Hospital again, which she took at 8 o'clock, there was a long line, there is a long line everywhere
(Father- When I spoke to the security guard, and then got ripped off) Then when I was taken in, the doctor saw how his blood got starved for the first test before treating my child, when 7-8 turn out when the blood came out, my child stopped beating, when the blood stopped, all the doctors were there, all the doctors were senior, they tried their best and started breathing and then started taking medicines. Done (Father- he started reading the tour, etc.) Mother- Then he started visiting on the tour quite a few times. He came back quite often the doctor was with the child until his visit was normal. When the child broke normal then he referred me from emergency, then shifted to the room (Father - here is the number 18 ward in the H-block) so when I sent it there was a line of children. Every five minutes two children were dying, then in the second minute four children were dying. Meaning no child had gone away from there, all was over, all the children, meaning by the time I was admitted till the morning, there were above fifty children. Father- He was also negligent there, the doctor was working well till he was in the emergency, after going there he started taking more carelessness.
<about treatment> Mother- The doctors of the emergency who wrote that medicine were running the same medicine, to change the medicine, because when the senior doctors came, they brought my child along with them. Beats were checked every two minutes. They said to raise Father-Blood, they did not offer it either. Then the doctors who came there started scold them all. The file did not even say our address was made disappear. Mother- Shivam, there was a shortage of the child, because of this, the people had suppressed the file of the child, that there is a deficiency in the child, the child was not to be saved anyway and those people had no other treatment, so What can we do with when the big doctor came, he asked for my child's file, then rebuked him and started treatment again. Then treated him as long as he had breath

2.1 Can you please let us know about the illness and events that led to death of your child?

Hospitalization period

3 Now we would request you to tell us something about the period of hospitalization for your child?

4 Could you please describe your overall experience about the hospital care?

5 Could you please describe your experience about communication by the various hospital staffs during hospital stay?

Father- According to me, who was doing us, it was not a mistake of the doctor. There was no mistake of the doctor in the treatment. Yes, but the lady who was taken for the first time was a lady doctor, she spoke upside down. Mother- They told me that when the child was normal, she was born at home, why did you bring it here, for whom have you brought it? The baby's tree is not moving, the leg is not moving, and why did you bring it here at home. Father- Meaning that on the other hand, they used to go upside down and were automatically thinking that we had not spoken anything yet, and understood themselves.

Death and post death period

6 Could you please tell us about the death and death declaration of your child?

Mother- The child was finished between 3.30-4 am. Father- As both of us were sitting together, as both of us came out, my elder brother-in-law was giving oxygen to the child, meaning we had been in places for two-three days anyway, and then broke out in the street. As soon as I sat down after 10-15 minutes, I saw that the child had expired, meaning he had come to speak. Mother- My brother was with me all the time, was not away from the child, as I was driving, on the go, my brother said that you are getting sleepy, I will take you rest, so my brother is pressing the pump Was. When the doctor came to treat him, his heart

stopped beating, then when the doctor saw it, blood came out of the mouth and nose and then it was over Otherwise I will not say that he had a shortcoming because he had told earlier, first Sister was when my first boy was over, then he said whenever I want to do a child after 3 years, I have to take a decision. And after that we will run medicine for some time, will test why you and your husband are lacking in such a child, what is lacking in the boy Still this time told me that whenever the child does, do it with opinion from us and do not do it before

Father- After the first boy was born, after that we went back to S Hospital. Mother- My case was spoiled by those people because I am telling you directly, whatever the deficiency. Father- We went to S Hospital, for that which we have run for 2-3 months, it is not, I did not run, I used to give the contract for paint, the boys thought that they used to go with them (wife). Went for three consecutive months but no hearing was held. Then said that the Chief Medical Officer who sits, went to him, then the same (wife) had gone, I was standing outside, so he also went careless. Mother said that your child's head was large, and it looks like this. Father- However, who lives in LN, there is an A Hospital, there was a professor and worked here. So I thought of going to them to talk to them to know what to do to get treatment, then I thought to work, it is a matter of home. He must have gone, he would have done something. Then showed in the emergency, it said that it would be surgery but nothing happened in the last

<cost> Father- Yes, the test was done from outside, MRI was done from outside, and the test was also done from outside. All the papers were there, now the child expired and threw everything.

<who declared the death> Mother- Do not know the name of the doctor, when she was taken to emergency, the girl was 25 years old, she was told that she had come

7 What was your and your family member reaction to the death of your child?

8 How was your experience about the procedure after death till handover of the body to you?

Father- The child was given immediately, that means whatever must have been put for 10 minutes or half an hour, keep the file, and there is no slip on the sister's gate without making a slip.

9 Who all from your family and community/neighbourhood were present at the hospital and how they supported you during this period?

Mother - It was May in the hospital, my husband and my elder brother were enough. We were just three. When he said it is no longer Shivam, it is now over. Then said now it is over, you can take it.

Father- Earlier Bauji was also there, but the time when he was expired was not the

10 What all rituals and procedures did you follow after death of your child?

Mother: I had brought him home directly from the hospital then he was taken away as the rituals are, as it is for the elders in us, in the same way as in the younger ones. Then my father took it all away. <who performed the rites> My father did all the action, the child's maternal grandfather did it. Father - 12-13 people went to burial. (Granny- All the people of our locality had gone, all of them had known)

11 How did you, your spouse (wife/husband) and immediate family members cope with the difficult phase?

Father - A quarter of a month is not worship. Mother- As soon as two months are over, my baby is finished. The 13th was over, now it has been two months, what is recovering as it was. One is my own little daughter, she is engrossed in it what else can she do. They pay more attention to it.

12 Is/was there any change in the interpersonal relationships between you and your spouse and other family members after the event?

Mother- Nothing, no problem, all is well.

13 How did the family members support you and your wife during the difficult phase?

Mother- My father and mother had handled me I have supported my husband more than that.

14 What were the reactions/ counselling and suggestions given by the relatives/neighbours/community after the episode?

15 Do you think and/or discuss with your spouse/ family member(s) about the death and related events and blame someone/something or feel guilty for the same?

The doctor and said that we tried our best but the child was not saved. Anyway, there was so much deficiency in the child. Father- See, because of that deficiency, we also did not say anything, now it looks like that, I did not say anything, I could not blame them either. Mother- It was our fault too much. Father- But when the first boy was the HOA, all the mistakes were made by the S Hospital. Even then, whatever tests were written, all the tests were done, an ultrasound cake was inside and the rest were done from outside.

Once the color ultrasound, all the tests, blood tests are inverted - all were done from outside. Mother- Whatever test they wrote, which could have been done from inside, they had got it done from inside, and what could not have been from inside, I had done it from outside, the medicine which they had given in my first child, I completed The medicine was eaten, whatever medicine they wrote from inside That means, whoever writes the pill from inside, I used to bring all the medicines, I have also consumed that medicine, earlier I did not leave any deficiency in the child. Even then my child had a spinal cord open and there was water in the head. That means there was no water in the baby's head, but there was no water in the baby's head, but yes was right, but there was a spinal injury.

(in between talk Granny telling her operation experience at S hospital) So in this child, I did not go there, when I went there in the eighth month, when the child was upside down, I did not know that time. Was that my child is lacking in this. He wrote for taking an ultrasound then got an ultrasound done and then it was found that the colored spine of the child has been opened again.

Then they saw me then gave them the medicine for infection, that the wounds of the baby which were water, should not be harmful for the mother.

Mother- This time I was late, I will not lie.

16 When did father and mother (if working) returned to work after the death of the child?

Father- My duty is not any government duty, you can go anytime. I started going to work after 15-20 days, but I felt less noticed. Nana - this is your job, if it comes to work, then it keeps on working day and night, the site does not come up, you have to sit, I do glass work, and they do paint work.

<The daughter enters the room> Mother- This is my girl. Granny - for this (2nd child) I did not take it anywhere but took it home. Nana - It is God who worsens, one is a doctor, another is a doctor, if there are deficiencies, he is not moving, everyone has to die, but when it is written, everyone has to die.

Granny- who is telling this (nana) Dr. N K Dogra is now old, he said that if there was a shortage in your girl, he did not do anything under pressure, he said that if the girl was lacking in you, then this (2nd child) child is also lacking in you. Your son-in-law is lacking, so the boy will fall, so I said openly to my son-in-law, do not consider it right for me, break the big people, get your check for understanding.

Mother - We will get that test done. Nana –Dr NK Dogra keeps talking to Saab, he worked at his place, he had said a test, got it done in medical, we said to get it done, but we will not get the report in private and will show all normal, will get it done in medical You will get a report.

17 How your and your family's life has changed after the death of the child?

Mother- Not like we were the same as before

Knowing cause of death in detail- Autopsy and MITS

18 What was the cause of death of your child and what is your perception about the value of knowing the cause?

Father- We knew that the reason is that the lump is there (Mother- They said that the infection has failed), the infection has occurred and has become hungry.

Mother- I want to know why this is happening. For this thing, think why there is a shortage, that whatever boy lives, why there is the same deficiency in one place. The remaining deficiency can also come in the hands and feet, it can come in any part of the whole body, but why the problem comes in the spinal cord, the information about this thing is necessary for us, nothing else is there for us.

19 In your view, what more could have been done to know the exact cause of death and the reasons causing the disease?

Father- Nobody said to get anything done. The earlier ones were probably asking for the test. Nana - No, I did not say that, just saying it upside down, the child is here then I said yes it is. Just then said that take it home.

Mother- delivery like baby when born was (1st child), they are normal baby as per the operation, they reverse the baby, whatever is inside the cucumber, it does not go inside, it is in the normal delivery It happens as if the child cried, he woke up and showed that you have to treat the child that your child is the same and this is the risk that you can see, the wound was also shown and the child had to be screwed. The child broke even cried then was taken for treatment.

20 Are you aware of any method for identifying the exact cause of death?

21 What are your views about the autopsy/ post-mortem for identifying the cause of death?

Nana- It is known that the post is in the accident of the mortem, but if not the HOA, what to think about it. Mother- We don't know anything about this (post-mortem)

22 What are your views about collection of the tissue (like done for biopsy to diagnose diseases) and body fluid samples for identifying the cause of death?

Mother- They did not take all of this. For this we would have agreed. Father- For this, what would we do for a couple of hours, for a day or two, if someone tells me to we do not see anything from him. We were never running out of money or treatment

Mother- If they had stopped me, we would like to find out why your child is falling, and why the child is dying. Means hungry, or infection, or because of which. He did not tell all these things. When Babu was over, we immediately drove away

23 How parents and family members can be approached for the tissue and fluid sampling?

Mother- At that time, parents are not in that position and they do not want their child to have a post mortem and there is a problem that in this way their child, the child is normal, will tear the child out, so any mother's heart is hurt. Will be there If we do tissue sampling in this way, then you will have to take the gift for treatment, then you can give it means that it is theirs, but I did not say it to my child, so I did not give it. <Who should approach> I cannot tell now, at that time someone from the hospital would tell

24 In your view, what factors are important for acceptance of the tissue sampling by the parents and family members?

Mother - The information you are taking from us, will be useful for giving feedback to someone later. Father- See, here I am talking about a boy who had become a boy, the doctors are talking like this, the people were quarreling with him, and the doctors had spoken as if his family had come to say remove this garbage, this garbage Remove, tell me like this. There were 7-8 people of that family the battle was fought again with the doctor and the fight with the guard. There were two or three children expire there then he broke and later the child got expires and then he too fought. It does not mean that they say that the child has expired or that there is no such thing that there is no quarrel and let go out of peace, they speak in reverse, and this thing is in front of me.

Decision making dynamics

25 In routine day to day operation who takes decision about the major activities (purchases, treatment, spending money) about the family?

26 During the hospital stay who discussed with the doctors/ nurses to make the decision(s) about treatment and other procedures.

27 after death of your child, who decided about the cremation and other post-death activities and rituals?

Father- My father-in-law took it, all the people who lived in the house also belonged to him, and the main decision was of the father.

28 Now, we are completing the interaction/interview. Would you like to add anything?

Granny - We were sitting outside, two or three women were on the other side, its father was also sitting there, my girl is also sitting there, she was sneezing, I asked what is the matter son no problem. Mom, nothing, and sent it in the morning to come to get it checked, it came to get the check done, to the doctor, there is a doctor nearby, but at my deposited Gurgaon work, I said if there is any problem, tell me, And I

was at home, my father was also there, my eldest boy was also at home Till then I asked him what time is there, there is no problem, so I did not bid, then I said you go in, I lay on the bed and then later told my father to speak to my maternal aunt, she came and said, Didi you sit here and Sushma are upset, as soon as I went in, hardly took 2-3 minutes and Babu got into it. Now half the torso and half the torso are out, tell me in that period, where can we take it now, nor can we take it private, nor can we take it to S Hospital, it is too far from here it takes time to go to S Hospital Now you tell me what it was raining outside. At 5.07 minutes, there was a born Babu got out of the bathroom like Babu got it on the ground. Now his father was giving me the street outside, you are not leaving, you are doing this, if anything happens to the girl, I will do it to you, as I was told to say, I am not one of them. Listened what I should tell them in which position they are in, like in the morning, I sent it to S Hospital, and the doctor said that it is like that

Mother- I am thinking that S Hospital will go and get feedback from him, whoever writes the test, he does not even know who will write the test or not. Father- He is speaking, we have no information on A Hospital to get the test done by A Hospital, just went for a turn, there is no knowledge, I have seen everything from S Hospital, so I go somewhere. Mother- The operation of my mother is the same, my younger sister also has the same operation of the appendix, and my delivery is also the same, now my younger brother-in-law is also treating his feet. And I have gone the same in this child, only then we have learned more and we always go there. Father- If he had said that he had to go to medical, then we would have gone; we have no information about medical. To get this test done in S Hospital, we speak upside down, see above, and tell me, I live outside, Gates stay outside, do not let them go inside. Mother- It is so, any problem has come in the child, as if there are any such things which are not understood by us, and then they speak upright in it. That you do not understand what we are talking about, this is the matter. They have a lot of cases too, now you have to see a man, see a man properly, they have to see millions. It is very crowded. Father- And if you ask anything, you speak, you become a doctor yourself, you speak like this.

In-depth Interview parents of deceased neonate-6

**1.1**	**Primary respondent (relation with the child)**	Mother, Father
**1.2**	**Age of the primary respondent**	Mother-20-25 years<br>Father-25-30 years
**1.3**	**Other members present**	None

2.1 Can you please let us know about the illness and events that led to death of your child?
Mother- It was Sunday, that day, the child was well all day, he had milk and slept, at 6-7 o'clock in the evening, baby had high fever. Father- He called me, then I came from the job, then I saw baby was crying a lot, and her body was very hot, after that we went with S Hospital. Mother- in morning we took her to hospital, after her admission there she got medicine and glucose after half an hour of medicine she opened her eyes, she was bit alright after getting medication. Then after that, she was shifted inside the ward. Then she was getting medicines, the doctor did not check her for the whole day that what problem she was having and he checked other children. She was getting medicines after half day passed doctor checked and said she is getting short of breathe that why we will have to put a pipe. We thought that if she is inserting pipe she will do well. When they inserted pipe, pipe got filled with blood, blood was coming from mouth. They went after inserting pipe, I went said a lot of blood is coming you can come and see that. Father - the blood was cleaned from the vacuum then more blood was coming. Mother- Then we asked why the blood is coming, so they said it is coming from the lungs, why it is coming was not told to us. Father- Did not tells the reason why the blood is coming, whether it was an infection or not they have read the report. Mother- I do not know how they inserted pipe we don't what happened to the lungs whether it hit the lungs.
Father – Blood should not come.
Mother –they inserted the tube after which blood came, they didn't told us about that. They inserted two tube one to clean her stomach, her stomach got enlarged, it was fine at home, after she had glucose some swelling appeared on stomach, second tube was inserted in her mouth. One child died in front of us. My baby was 14 day old. Other child died he was 10-12 year old, after that there was some fight in ward, they slapped the doctor. Then after that a child was killed in front of us, it was small. My baby girl was 14 days old. There was situation everybody was saying take your child from this hospital, here staff is not able to do anything. They give the medicine only and say if any problem, come to tell us. If we go to them, they didn't know that child is serious they should come and see the child. I said to my husband that go and call the doctor. They said child is serious they made us sign one paper after inserting the tube.

Hospitalization period

3 Now we would request you to tell us something about the period of hospitalization for your child?
<who inserted the pipe> Mother- there was one senior doctor and afterwards junior doctor came, senior doctor was telling junior doctor to insert tube and give injection. there was fight betwwen other patient and doctor, he slapped the doctor, he said you are keeping child for 2-3 days and there is no improvement, children keep dying there, the environment in ward was not good. we consulted one doctor that if we take our child to private hospital will he get proper oxygen through machine as here we were giving through ambu bag. they said its your wish, you can take to private hospital. then we went from S hospital to private hospital. there doctor saw the child and said baby condition is serious, baby had tube in his mouth. we took discharge from S hospital.
<how many day child stayed at S Hospital >father- we went the child for 2-3 days, we took discharge from S hospital and took the child to rainbow child hospital in malviya nagar, it was very costly hospital, then what could be done we again took the child to S hospital. mother- you have come with child with tube inside his mouth, child condition is serious, they said why you brought the child from S hospital. father- the child was not serious earlier.
<when did you come back to S Hospital> Mother- we took her back to S Hospital on the same day, after one and a half hours. Father- Within an hour or two, we took the ambulance to S Hospital. We had taken oxygen cylinder from private we had taken along with it. Earlier, at S Hospital they gave

LAMA as they said you taking your child by your known. Then when we went again they was not admitting the child in S hospital, they said go anywhere else. Mother- The child was very serious.
<how did S Hospital admit again> Father- After requesting a lot, I had requested for 45 minutes. The child was on a wheelchair, it was on ventilation.

4 Could you please describe your overall experience about the hospital care?

<what treatment they, after admitting the child again> Father- After admitting, they kept baby for 10 to 15 minutes inside Emergency, which is the new in S Hospital, then after that they sent back to ward 20-21. Mother- There was no machine they just kept baby. Father- earlier they were providing oxygen, on returning back they did not give oxygen, saying that this machine is not working, asked us to give oxygen by pressing Ambo bag, they told us to keep pressing, do well all night, till you do, the life of the child will remain, if you do not, we cannot do anything. Mother- we told many times to doctor to see our child.

5 Could you please describe your experience about communication by the various hospital staffs during hospital stay?

Father- we kept telling doctor to come and see our child she is having breathing problem, that my wife was having some problem, when we are vacumming the child we are not allowed to go anywhere.
Mother- they just used to say that they are coming, they are coming now. Their file work was not ending. Father- I did not know what file doctor had taken, they were noting something on it, don’t know what she was doing it was more important to them they did not come to look at the child. Mother- at last they come and telling us that why didn't you call us, mean they don't know that the child is serious there, the child should be checked again and again, and they don’t let more than two to go inside, I had an operation for delivery.
Father- my wife was there inside, it was her 1st delivery through operation, bouncer was not allowing me, my mother she is diabetic patient to go inside, not allowing any other patient, I thought my wife would have tried pressing bag now I should go inside to press ambu bag. I went down to give food to my mother till then I was pressing ambu bag, till then she was alive, after half an hour phone came that this has happened, I rushed back upstairs. Mother- when my baby stopped breathing then doctor came, earlier she didn’t came even on calling. Father- after those 4-5 doctors gathered one by one. Mother- Later when after this happened they gathered, but no one came when my mother-in-law went many times to call doctor that child is having problem. They were just saying that they are coming, but no one came.
<after you took your child again, then when did she died>mother- we went back early. Father- she died at 2-2:30 at night, we got back to hospital at 12:30-1. Mother- it was dark when we reached back hospital. Father- at 2 in afternoon we took discharge from the hospital procedure took 1 hour, then we returned back to hospital at 4:30 in evening after one and half hour. Mother- it was 6-7in evening, we don’t remember time.
<investigations>mother- test were conducted. Father- we didn’t to investigation at S hospital, it was performed in MCD dispensary in DC, mother- all test were done before delivery. We were taking all treatment from there. Even at last they also performed something then referred to S hospital for delivery, at night we took ambulance and went to S hospital, even they didn’t perform normal delivery, then I delivered the baby at S hospital through operation.
Discharge from S hospital after delivery- Father- It took one week. Mother-she was born on 3rd and I got discharge on 8th. Then after coming to home baby had fever, rash also appeared on her back, it was due to heat. We showed her in DCW, they gave ointments and medicine, then the rash was drying up, and they said that she got infection from S Hospital. They didn’t tell how this infection happened.
<behavior of doctor or nurse> Father- behavior was not good in starting on admission, in last also itt was not good.
<what you did not find good> Mother- first they didn’t allow anyone to come inside, I got operated during delivery of my child, I was not able to hold my child as I got stitches during delivery. My mother-in-law was inside, they were allowing just one member at a time, the child was very serious, yet they were not allowing . When the child was very serious in the last, then they called us both turn by turn. Secondly when we used to call the doctor, then didn’t used to come, they used to come and and went away after seeing it. When the child is taking medicine through glucose, then she was breathing, it had been half a day, when they didn’t check, then in half a day you are saying that the

child is not breathing. Well, when they put the tube the blood came from his mouth, they didn't explain why blood came from baby mouth no reason was told to us. It was not understood by anyone. father- as we reached hospital after one and half hour, baby treatment started, she got glucose, medicines, she was given breathing through ambu bag, after that after one and half hour they told us to sign on letter it was mentioned that your child is very serious and nothing can happen. Mother- baby only had fever. Father- they didn't tell us what the problem was or was there any infection. Directly after two hours of treatment, they said the girl will not be able to survive, they made me do signature on two papers, such a big letter was written over A 4 sheets and they got two papers signed by me, what I would do then. (This was when the first admit was done) mother- this all happened when we admitted for 1st time, we went after they inserted tube. Father- They had already broken the trust, then after that one child died, one more dead occurred of 11-year-old, one more baby died, they had fight with doctor. Then there was some fight between them. Then I started feeling like a fear, I thought it was better to get discharged from hospital.
<nurse behavior> Mother-nurse was all there. Father- the nurse was doing everything the doctor used to tell it to take and prepare the sample one was giving a bottle of glucose. Mother- everyone was taking blood sample. They didn't even know how to take properly as blood was falling from the tube. Father- He does not know how to take it, even the blood was coming out of hand. Then the blood was coming out of his hand then I took cotton and cleaned it with Dettol, after that blood stopped.

Death and post death period

6 Could you please tell us about the death and death declaration of your child?

Mother- i was there only, i called the doctor. father- i was with my sister and her husband, we were went down to bring food for my wife and mother, after that my wife called me from my mother phone to inform me.
<who declared about death> Mother- my baby was getting short of breath so I send my mother-in-law to call the doctor, then she went and called doctor. Then one was inserting pipe, and other one seeing that the child is running short of breath, yet she was still putting the pipe. After that baby was breathing less, there was no life left inside the child. Then they pressed her stomach still no breath came. Then after that, the doctor came at that time there was only one doctor at night time. No senior doctor came at that time. Father- when we went again the senior doctor was present, after that I don't know. My sister and mother used to get there treatment from S hospital. My sister told me this nurse was earlier a compounder at this hospital now she have become doctor. Mother- all were of small age. Father- out of those two were senior doctor there behavior was good, among ladies staff no behavior was good. One sir who was south indian came at time of death. Mother- when baby got expired then he came. Father- no he came earlier.
<what they told you> Mother – I went out when my husband came inside. What were they telling? (Mother asking father) Father- they didn't tell anything they gave Ambu and filled medicine with glucose. Mother- we bought ambu from chemist. Father- when I was discharging her they were not allowing us to take ambu, they said this ambu is government property you can not take it. First I rushed for getting ambulance and now for ambu, then i tried to get ambu from 5-6 chemist shop, then someone gave me contact number of person from where I could get ambu, then that person took 1100 rupees, otherwise it cost between 800-900 rupees.
<cost>father- only money ws spent on ambulance, no money was spent on treatment at S Hospital.

7 What was your and your family member reaction to the death of your child?

father- at that time my mother was present. mother- they left after telling that child is no more, they didnt give us anything in written, they directly said to take the child.

8 How was your experience about the procedure after death till handover of the body to you?

father- i said them to write on paper that we want to take the child, they didnt wrote anyhing. mother- when baby got expired it was there responsibility to write something, they just said you can take the child. Father- When the child expired after that they made me to do signature on paper, they said if you want death certificate you can come after one month and show this slip, I said you can keep this slip with you, I don't want as it is of no use. I signed at last.
<time taken to hand over body>mother- they said at that time only, my father and uncle was told imediately. father-death happened at 12:30, after one and half hour we left from the hospital.

9 Who all from your family and community/neighbourhood were present at the hospital and how they supported you during this period?

father- at hospital, my sister, her husband, my mother. after sometime my father and uncle came. uncle lives near to us.

10 What all rituals and procedures did you follow after death of your child?

Mother- we didn’t child to home, we took directly from hospital. We left from there at 3-4 pm, it was already morning. <Where were you till four o'clock> Father- We were told to go to near Kalkaji temple and bury the child we were sitting for quite some time till 4 o'clock , Outside the Kalkaji crematorium, which is in front of the Kalkaji temple. When we were sitting there outside crematorium one truck came, and he informed us that here burial doesn’t take place, you can go to kalandi kunj. You can bury on the banks of Yamuna river. Then we went there. Father- we buried the child, all procedure was done till 6 in morning and till 7 we reached home. I went with my father, uncle and two friends. Father- we bathed the child feet from taking water from Yamuna river, the burial person was telling us to perform ritual.

Mother- naming ceremony happen on 12th day, we happened her vanshika, we brought teddy for her. After so many years, a little baby came in the house, she was the first child.

<rituals>mother-we didnt do anything in case of child. we only did her naming ceremony on 12th day after she was born.

11 How did you, your spouse (wife/husband) and immediate family members cope with the difficult phase?

father- we are supporting each-other, try to remain happy but still remembers our child. mother- i started doing little bit household work after my stitches were cut.

12 Is/was there any change in the interpersonal relationships between you and your spouse and other family members after the event?

father-there is no change in relationship, we support each other and console eachothe, what else we could do.

13 How did the family members support you and your wife during the difficult phase?

mother- all family memember supported and consoled.

14 What were the reactions/ counselling and suggestions given by the relatives/neighbours/community after the episode?

Father- neighbors says they didnt believe how suddenly all this happened. mother- our baby was fine, she was 14-15 days old, but all used to say that she must be 3 months old. (mother-father showing pictures of the child in their phone) mother- this is picture of child on day she had fever, baby was vaccinated on that day, my sister-in-law came to see her as she didn’t came on naming ceremony of the child. No one could tell she was 14-15 days old child. Father- her birth weight was 2kg and 700 grams, she was delivered by operation.

15 Do you think and/or discuss with your spouse/ family member(s) about the death and related events and blame someone/something or feel guilty for the same?

Mother- We ask this to the doctor that what happened when tube was inserted, why blood came does the tube inserted hurt the lungs, what happened. We tell everyone these things, everyone says this was the fault of the doctor, because of the doctors this all happened and blood came. Father- On the day when the baby was born, she gave smile at the same time. She was born in the morning at 2.30 am, they were not letting go even for next day, visiting hour time was from 4-6, then I went, she was smiling. Mother- When she was born she had jaundice she was kept in light for 2-3 days at S Hospital after 3-4 days her jaundice was cured. Father- After the operation, the child was also discharged with mother. They didn’t discharge until the mother and child are well. When the child was well, they discharged with mother. Father- baby had fungal pimples after she was born, in stating it was only 1-2 rash after that it become pimple in 2-3 days. I asked what they were doing for that, then then the doctor did not give any response. They said, let the jaundice be cured, then when the jaundice is cured, then your wife will be discharged, now the new patient who comes will share the bed with your wife. My wife had operation for her delivery, how can they keep two patients on same bed, they said now 4 days have passed the stitches must have dried now they can keep other patient with her. What if accidently she got hit by her, then problem could occur for both. Mother- It was happening. Father- Fungus infection was present, they didn’t even see it, they just cured jaundice, and then the doctor discharged after curing jaundice. Mother – I had big operation, after that, two patients in one bed, and our child is also with us, how much is the size of the bed, it was small bed so a lot of problem was there. Father- Before our child, a boy had jaundice inside the ward, after that, our baby got jaundice

from him, after that the infection started spreading and it started happening in other children as well, so it started coming next to the next side. In the second ward, it was a machine inside which 2-3 child could be kept, but 5 children were kept in that lighted machine. It was so congested, what should I tell you. I thought it was bad experience at S Hospital. It was going to be very bad with us, I saw baby face again and again.

16 When did father and mother (if working) returned to work after the death of the child?

Father- I went after a week at work, I am in sales job at Adidas Company.

17 How your and your family's life has changed after the death of the child?

Father- few change like some negligence happened from our side as not keeping cleanliness, holding child without using sanitizer, all this was not told by anybody. i used to on job, at hospital they told infection has happened, whosoever used to come hold the child like that only, maybe this was the reason. we should remain careful in future, the report is with doctor, that after seeing that report after 2 hours senior doctor made us sign letter. <any substance abuse>father- no remained same as i was earlier. <next pregnancy>father- doctor told us to plan next pregnancy minimum after 3 years.

Knowing cause of death in detail- Autopsy and MITS

18 What was the cause of death of your child and what is your perception about the value of knowing the cause?

Father- We did not know the reason for the death of the child, we were just told that there was an infection, what was the infection was not told. <value of knowing the reason> Father- What I understand more than my child is, was not more than my child.

19 In your view, what more could have been done to know the exact cause of death and the reasons causing the disease?

father- you can go to S Hospital or send someone there you could see what happens there.

20 Are you aware of any method for identifying the exact cause of death?

Father- Yes, I had some information. Baby was not vaccinated which is given at time of birth, I have doubt I think BCG. Mother- baby was given that vaccine. Father- What could it be other than that, or a test that they had in the last blood test, after that we had taken the child after discharge, the report was still there, we have not taken that report Maybe something would have written in it.

21 What are your views about the autopsy/ post-mortem for identifying the cause of death?

Father- At that time it came in my mind that there would have been a post mortem or something like that, a lot of things and things are going on in my mind at that time, but could not be done, as we are middle class family problem also occur simultaneously. <necessity for post-mortem> Father- I would have gone for post-mortem to know what was the problem to my child or who was responsible for death of child. <religious barrier for post mortem> Mother-post mortem does not happen in the child done. Father-in elder there is no such problem.

22 What are your views about collection of the tissue (like done for biopsy to diagnose diseases) and body fluid samples for identifying the cause of death?

Father- i would have agreed for this technique, it is a good technique.

23 How parents and family members can be approached for the tissue and fluid sampling?

<who should approach> Father- something could happen if we would have tried. . <whom they should approach> Father- I could have done all these things my father also works in the hospital, is the Eye Hospital and the RC Hospital branch in MN, A Hospital, it is also for child. So I could find out some things from there too, if I tried to get it done. But did not do it, child was not there. Father- all this would have happen in S hospital? At that time there was no one, I console my wife, mother, because they lived with the child the most, the mother did not eat for at least a week. I was so upset, all these things were also necessary together. <how should they explain about technique> Father- by whatever way we would understand.

24 In your view, what factors are important for acceptance of the tissue sampling by the parents and family members?

Decision making dynamics

25 In routine day to day operation who takes decision about the major activities (purchases, treatment, spending money) about the family?

Father- My wife and mother, both of them take the decision, something has to be brought, so both of them do it. and discuss things together. <decision about treatment> Father- for small problem we go to muskan clinic near ISKCON temple. We get better by his medicine in day or two.

26 During the hospital stay who discussed with the doctors/ nurses to make the decision(s) about treatment and other procedures.

Father- at hospital my wife was taking decision, she said proper care of baby is not happening, take baby to private hospital, then we went, then we thought taking baby to lady harding hospital in CP. then my father had some reference at ajay hospital then we went there, there they said it is case of S hospital no one will take, then someone suggested rambo hospital but said it will cost rupees 4-5 lakhs, due to reference they said you can get treatment in 2.80 lakh, you can deposit rupees 80 thousand now and there is 10% guarantee your child can survive.

27 After death of your child, who decided about the cremation and other post-death activities and rituals?

Father – my uncle performed all the rituals, he dug the hole in mud to great extent after that I dug that, then I put baby inside it. After that we put some incense sticks. My uncle had some knowledge regarding this as it has happened to him. My father also went with us. We don't have any knowledge as it never happened with us, it was 1st time.

28 Now, we are completing the interaction/interview. Would you like to add anything?

Father- this was from our side, we have shared with you.

In-depth Interview parents of deceased neonate-7

**1.1**	**Primary respondent (relation with the child)**	Mother, mother-in-law
**1.2**	**Age of the primary respondent**	Mother-35-40 years<br>Father-40-45 years
**1.3**	**Other members present**	Mother-in-law

Context- Mother was not able to speak because of the health issue, so the mother-in-law was speaking on her behalf, Mother was adding to some questions. Age of her husband is 40years, mother got pregnant after 15 years. She has continuous abortion.

Events that led to death of the child

2. Can you please let us know about the illness and events that led to death of your child?

Mother-in-law-Baby was delivered at F hospital, my daughter in law was admitted there, she had BP, so she was admitted earlier, she delivered the baby at 6 months, she was not well, then doctor said there is delay of 15 days, so they said to operate, they said if we will deliver by normal delivery then there can be danger to child, then I said there should be no danger you can operate, so they delivered by operation. Doctor said child is fine but is weak. Since conception a lot of money has been spent. Then we thought to go to S Hospital, as doctor in fortis was not discharging her, at S Hospital my brother-in-law son knew some doctor at S Hospital, he talked to doctor and he said to bring baby to Hospital.

Mother- 6 months could not be completed even one day was left. They were saying child is weak, we will keep child in ICU for 3 months and we have to admit baby in nursery for 3 months, we were not able to bear the expenses of three months we had already spent 7 lakh rupees, and also expenses for my operation, spent 13 days in hospital. I used to get 3 injections for baby survival.

Hospitalization period

3. Now we would request you to tell us something about the period of hospitalization for your child?

Mother-in-law- we went at 6,on tuesday my son had off, we went at 6 in evening baby was there in hospital for whole night, i was there in evening, she was removing oxygen pipe again and again, even moving her hands and feet, my son stayed at night, baby was becoming weak, was turning black, then in morning i came to hospital at 9, and she expired at 12 in night.

4. Could you please describe your overall experience about the hospital care?

<about treatment> they were giving fine treatment.

<about investigation> it was fine.

<about cost> no money was spent in S hospital.

5. Could you please describe your experience about communication by the various hospital staffs during hospital stay?

<about behaviour HCP> It was good.

<communication of HCP> They were saying that we had already said that the child will not survive, they told her survival is difficult, we are keeping her and looking after her, if she survives that's good.

<frequency of communication> they used to tell us on time to time, doctor and nurse came to check baby, lady doctor was very good.

<completeness of information> they used to tell us whole thing, that her parts are not fully developed.

Death and post death period

6. Could you please tell us about the death and death declaration of your child?

During death my son and son-in-law was there at S hospital, i came back home at 9 in night, my son called doctor when he saw baby was not breathing, then doctor came and said baby is no more.

<skills of communicator>

nothing from doctor side, we were thinking if baby was kept in nursery, then she would have survived, she was kept in ward no.21, if i remember.

7. What was your and your family member reaction to the death of your child?

When baby goes away, mother will weep what the mother will do, at that time everyone felt equally bad, we also did not feel good, one member was small, even though he is older, and we felt bad, we were also crying.

8. How was your experience about the procedure after death till handover of the body to you?

Yes only some paper work was done and they discharged them, no time was wasted.

9. Who all from your family and community/neighbourhood were present at the hospital and how they supported you during this period?

Post death procedure and coping mechanism

10. What all rituals and procedures did you follow after death of your child?

We brought home, nothing happened at home, then we cremate with wood, not buried, we cremated.

<body preparation>

no bathing took place, as she was very small. 4-5 people went in car to cremate her in chirag delhi.

Mother- she was not alive what is to be done.

<about timing>

They came from hospital at night, they did not go at night we took the girl in the morning we do not burn at night. My son, son-in-law his friends went for cremation.

Mother- They came back in half an hour, we do not cremate at night.

11. How did you, your spouse (wife/husband) and immediate family members cope with the difficult phase?

We try to remain happy.

<change in physical state>

She is still not doing household work, as her operation was done, then she fell ill and got a shock, she has BP problem. Don't know what happened to her last week, she suddenly had some shock after that her voice become weak, she had BP problem since 1st pregnancy, there was no problem in 1st child she was delivered normal.

12. Is/was there any change in the interpersonal relationships between you and your spouse and other family members after the event?

No there is no problem between by son and his wife. he cares for her, today also they had to go to doctor but came late at night from marriage so they will go to doctor next day, she is getting her treatment from M hospital.

13. How did the family members support you and your wife during the difficult phase?

Relatives give support, yes they helped even with money, we got support from daughter-in-law side and relatives from baby father side.

14. What were the reactions/ counselling and suggestions given by relatives/neighbours/ community after the episode?

Yes, the relatives came for support everyone came and what could they have done, no stone unturned on our behalf, now whatever will be the will of God, it was not ours, she was 15 days old baby and she died after that.

15. Do you think and/or discuss with your spouse/ family member(s) about the death and related events and blame someone/something or feel guilty for the same?

We do not blame anyone because the girl was like this, she was weak what could we do? She was getting weary and weak for 13 days, doctor said the weight is increasing, one gram of it has increased, he was speaking like this, but there was no increase in weight, she had a weight of 450 grams. And the mother had also become ill at the time of pregnancy, she used to get 3 injections, it used to take two daily and it is used for growth of the baby, but no growth happened.

16. When did father and mother (if working) returned to work after the death of the child?

Baby father returned to work after 2-3 days, he is property dealer.

17. How your and your family's life has changed after the death of the child?

No there is no change in mother life.

Knowing cause of death in detail- Autopsy and MITS

18. What was the cause of death of your child and what is your perception about the value of knowing the cause?

Mother-in-law- We know she would not survive she was very weak, even doctor used to say this, her brain was not developed properly.

Mother- What to know

19. In your view, what more could have been done to know the exact cause of death and the reasons causing the disease?
If there is something, then nothing has happened to our girl, no we do not know anything like this.
20. Are you aware of any method for identifying the exact cause of death?
<about Postmortem>
we get to know about cause of death, we do postmortem to know how someone died.
21. What are your views about the autopsy/ post-mortem for identifying the cause of death?
No, we do not know this, it has not happened to anyone.
22. What are your views about collection of the tissue (like done for biopsy to diagnose diseases) and body fluid samples for identifying the cause of death?
It is good technique, it does not happen on our baby, we don't know anything about this, it will be time saving and also there will no tearing of body.
23. How parents and family members can be approached for the tissue and fluid sampling?
<whom> it should be explained to child mother and father.
<who should approach> Doctor should tell about this.
<how explain MITS> explain as procedure will take place.
24. In your view, what factors are important for acceptance of the tissue sampling by the parents and family members?
Decision making dynamics
25. In routine day to day operation who takes decision about the major activities (purchases, treatment, spending money) about the family?
Mother-in-law- Decision are taken by me and son, we are three person at home, we dont involve relatives in decision making.
26. During the hospital stay who discussed with the doctors/ nurses to make the decision(s) about treatment and other procedures.
Mother-in-law- I took the decision at that time, a lot of money was spent at that time. We knew baby was very weak, then we called at S Hospital they said bring the child there. We only think if we could have got nursery in S Hospital she could have survived.
27. After death of your child, who decided about the cremation and other post-death activities and rituals?
I took that decision about cremation, we don't bury child, because if we bury child dog can pull the child, so cremation is practiced. We didn't consult anyone it was my decision.
Summarization
28. Now, we are completing the interaction/interview. Would you like to add anything?
Mother-in-law- no we don't blame anyone, when it was not ours so what can we do. Our daughter-in-law has fallen I someone eyes, we went for that someone told about it. The first one was only 7 months old there will be a child to play.
The girl was born, 6-month-old was finished in the stomach, then a 7-month-old, at that time there was not much information, at that time I went to G hospital, when 2nd one got expired then all the tests were done and there, I had a problem in kidney so they are confused because of this that BP increases and then took a chance after many years, this is the third time. It is done, whatever God will give. Either boy or girl it should be fine. BP medicine is going on right now.

In-depth Interview parents of deceased neonate-9

**1.1**	**Primary respondent (relation with the child)**	Father, Mother
**1.2**	**Age of the primary respondent**	Mother- 20-25 years<br>Father- 25-30 years
**1.3**	**Other members present**	Sister-in-law

The mother of the child was staying at her mother place, at the time of interview father, mother and sister-in-law of the mother was present. The delivery of the child took place at home.
Sister-in-law: He is son-in-law in our relationship his mother has gone to the village. We had arrived from our village, we already knew about the situation, the child was upstairs, there was a doctor in the room, there was a babysitter with him, the child did not cry even after normal delivery, his hands were closed, and the doctor said, the condition getting worse, move to S hospital, we immediately took it by three-wheeler. On arrival at hospital, the nurse started shouting like you did not know what could be happen, like baby may not be able to speak, may be mentally retard.
Father: This was our first baby we all are living in a rented flat

2.1 Can you please let us know about the illness and events that led to death of your child?

Sister-in-law: The baby delivered in home, a doctor with nurse came to the home for assisting. They tried by pushing and pressing the stomach. Enough time they had spent in this process. They came around 8-9 pm and baby delivered at midnight 12 o'clock. At that time, child was normal, but did not cry. The doctor tried to cry the baby by tapping her legs and at back side. Her hands were tightly closed. After some time, they asked to move S hospital. We had hire a 3 wheeler for same and reached at S hospital. Here, doctor initially refused to admit but we had requested as the child was critical. Then, they admit and brief about the condition. The baby was in critical condition, having episodes of seizures.

Hospitalization period

3 Now we would request you to tell us something about the period of hospitalization for your child?

Father: Admitted in three ways at night, after that the doctor told that she was seen as such, she had to admit at night, left at 12 o'clock at night and reached 12.30, then after inquiry 2.30- Admitted at 3 o'clock. After that, the child expires at 7-8 pm on 5th. For the first two days, they were just giving glucose, they did not put any new formula, but they just added glucose and they used to press the continue which pressurizes the oxygen, which was breathing and air was going on inside the pump. Sister-in-law, but her fist did not open, her fist so tight means how many planks are there in it, we were not able to open it openly, so tight it was closed, I did not know what had happened to her.

4 Could you please describe your overall experience about the hospital care?

Father: We stayed with him for the whole night, the doctors were not even listening, once he gave it at 10 o'clock and his seat was changed in about four hours, he gave it at 10 o'clock, then someone would come around 3 o'clock, He filled the entry and came to give medicines at 2 o'clock and gave the time of 2 o'clock and came at 4 o'clock, there is a direct 3-hour gap. Entered that we came to give medicine thrice while giving the medicine once in the beginning, after that we slept for 2 hours and talked about it, there were many children there and if the condition is disturbed then it will not see the condition, it will die anyway They say this in advance, the doctor, they speak just like that, they do not even give it, they will die, where did you create it, they did it, they said it as soon as they left, this behavior is not getting right , Will not be said anywhere, it is said right.
Bhabhi- We said Doctor is not opening this mouth. Father- we have said that you do not refer, do not even refer, just take it with your surety of your responsibility, we will not write the transfer. If you refer, then we can get admissions anywhere or not. Yes, I said to them, then said, take away from your will that I am taking on my responsibility, if I write this, then it is written proof. Let's do. Everyone demanded that till now they were getting treatment proof. Referee gets admitted, if he takes Serious at his own will, the child will not put his hand. We became very strong and could not be moved.

5 Could you please describe your experience about communication by the various hospital staffs during hospital stay?

Father- They did not listen to us, and will give medicines twice and will offer four times that we are giving medicines so many times. Sister-in-law: Now as the child is vomiting, the race is going on, the sister is vomiting, look at the child, but you did not listen. Father- One of them was a learner who was giving medicine, there is a doctor with him, so according to his own practice, the medicine was to be used. He was applying glucose in whatever injections were put into it. A bottle of glucose was kept in it and there was a mosquito in it, we saw it was going to be put in the glucose. Then we said that it is a fishmonger, otherwise it will kill more children then it threw that glucose and the other one gave glucose. When it is open like this, the mosquito will not go in it was not known that even the mosquitos were inside.
Father: Big doctors used to come in the day, they have limited time to visit their junior doctor only talks comfortably in presence of seniors otherwise they don't entertain us. Even we cannot tell the problem. Condition of child
Father: They were talking that only 1% survival rate in this case.
who communicated the information: One lady doctor with stethoscope usually visit and one lady must be sister(nurse) who gave the medicines, having 20-22 years of age. She must be newly recruited.
<nurse behavior> Sister in Law: Nurse first insert cannula in hand of baby and told to hold that cannula. It was slipped many times and she told to take another cannula of 150 from market.
Father: Nurse told that you have tempered the cannula and need to purchase another one from market. Otherwise you need to hold this cannula manually whole time.
<investigations>Father: we had conducted all the prescribed test. At same day, we have done blood test, ultrasound and many more. Yes, another test, MRI was also done. An X Ray was done from another building of hospital. We have collected the report of same from another building.
<cost>There were no as such expenses had happened inside hospital. My wife and me, only were present there in hospital.

Death and post death period

6 Could you please tell us about the death and death declaration of your child?

Father: we were in the hospital and hold Ambu bag. We called a doctor to see the condition. She moves her legs, respond as some one touch her body. Initially, she moves her tongue outside. Sister in Law also feed the milk. Earlier the baby condition was in control but at 4th day in the night, her legs movement had stopped and condition becoming worse gradually. Nearly. 6-7 am, she stopped breathing and dead. then we called a doctor to check the condition. Doctor check her by stethoscope and declare dead. Earlier, at 4 am, irregular breathing was reported by doctor and at 6-7 am she expired.
<sympathy>From two days before death, urination and defecation were stopped. So the hospital staff told, all these were accumulated inside the body and that lead to stop the blood flow.
<doctor told any cause/reason> Father: according to doctor, at the time of born, contaminated water was entered in the stomach. We been delayed in hospitalization also. We had arrived at hospital near 12 PM but admit at 2 – 2:30 PM. So the contaminated water might be act as a poison and spread into entire body that could be reason of death. Another side, hospital was not good. They don't have equipment, even they were managing ICU without oxygen. The attendant like me, had to manage all things like holding cannula in hand every time and it was very risky

7 What was your and your family member reaction to the death of your child?

Sister in Law: Then what! everyone is drowned in sorrow, someone's tears come out. Sister-in-law (child mother), the condition was worsened, the child who was given the filling, this was like that, due to heavy crowd and not having space, she(mother) stand and roam around hospital since three days. It will be broken on the ground, so now it hurts its waist. Father- As soon as the baby is born, it remained standing for 3-5 days, after that she was suffering from severe backache. one should be rested in this condition. Sister-in-law, she remained standing day and night last time she slept on the ground near hospital for a while.
Father- We were in the H block, on the third floor.
Sister-in-law: Our baby was kept in a room surrounded by glass.
Father- Before our child, 5-6 children were dead, due to the same cause i.e.: contaminated water

8 How was your experience about the procedure after death till handover of the body to you?

<time>Father: then, the hospital staff said to sign on a paper and take stamp on same mentioning date of expiry and handover the dead body.

Then every step completed quickly, they did all formalities in short time and give a duplicate copy of handover of dead body. We return from there after getting pass slip. Downstairs, our Mom and Dad were waiting. Then we left the hospital at 7 pm. with all family, we had stepped to our flat in a rented Auto.

9 Who all from your family and community/neighbourhood were present at the hospital and how they supported you during this period?

Father: we both (my wife and me) were there. Wife: Father in law and mother in law were also there.

10 What all rituals and procedures did you follow after death of your child?

Father: we came to my maternal house. We have done burial procedure near a crematorium near maternal house. At there, the ritual starts after taking a bath of each member and dead baby. The dead body was rapped with flower and buried. Before that, eye liner and oil applied on the dead body so everything happen in write manner.

11 How did you, your spouse (wife/husband) and immediate family members cope with the difficult phase?

Father: Slowly-slowly, we been familiar with this situation and try to understand the situation. As we are practical and educated. Everyone in world has some pain and no one is satisfied. Everyone has different kind of problem. In our case, when she is more, we all have to adjust in this situation.

Mother: I use to see her photo and weeping till now. I have not emerged completely form that tragedy.

12 Is/was there any change in the interpersonal relationships between you and your spouse and other family members after the event?

Just had a regret that if you had taken it to private, then there would have been good arrangement there. Just like they just treated our baby with limited or partial equipment in S hospital, after that they had no other way or formula to drain the contaminated water from stomach.

Mother- See the photo (in Mobile), the pipe that is visible that was inserted in mouth.

Father- In the name of treatment, they just inserted a Ambu bag and tell us to hold it. What could be expected from that incomplete treatment. We just keep pushing the Ambu bag and her stomach become bulge.

13 How did the family members support you and your wife during the difficult phase?

Father: At the time of death, my mother in law, father in law, mother and father were there at my home for moral support.

14 What were the reactions/ counselling and suggestions given by the relatives/neighbours/community after the episode?

Father- See, what he is educated, he has become aged/experienced but he has no idea. We thought that nothing would be ahead of his treatment. How will the child get this benefit or not? If we were looking at their treatment, then we already knew that it is not right to be here and that they are not writing the referrer, we cannot go to a big hospitalist in private. Then we also thought as long as there is breath, show it here. People in the neighbourhood said that you lived in the Kannur district, why not consulted there, why you took it there, people all around, has their own opinion. Sister-in-law, when a person goes away, many people give their brains, that if they do not move here, if they move here, it would have been right, the child would have been right if they had stayed at home, at first no one said anything. The hospital was bad.

Father- It was big but it is not for small children, means that it is suitable for treatment of major accident and other youngh and old age related issue.

Sister-in-law: they did not care so much there. We had not experience to select a write hospital

15 Do you think and/or discuss with your spouse/ family member(s) about the death and related events and blame someone/something or feel guilty for the same?

Father- We came to know on the third day that the child will not be cured with the treatment here, it will be over, this is what we said to him that she can be cured if we visited private hospital, it can be improved there, and they could treat with respect. Here, in the name of treatment, they just infused glucose. And the infused pipe also been detached after some interval. Who can we expect that some medicine infused with this disconnected cannula.

Father- What do we blame, consider ourselves, that now it is a matter of regret that we would have stayed at home or admitted to Kannur.

Sister-in-law: It could be better from here, if we gave desi treatment from our village. They did it from leaf of Jamun.

Father- The child was not weak, everyone in the ward was thin this child was healthy. I think it was not treated properly. It is with us that now we will never be taken there and advised others to stop visiting S Hospital. We will say do not take it there. There is no proper treatment. According to me, the doctor is not taught well, just comes with sister and new doctor to visit the ward, there is no good treatment there. Mother - Had asked about blood too, there was no lack of blood. Father- If there is a lack of blood then we can make the blood come out. Put aside, let the child go, just as much as it will be. There was no feeling that blood was coming out from where the canula was planted. Sister-in-law treatment was not being done right there, it is just this. We said that I was agreeing to leave the child. He was walking hand and foot and was fed milk, honey is eating everything.

Father- Gradually the dirty water kept increasing into the baby's stomach. The lungs got stuck, the movement of hands and feet stopped, when the dirty water is gone, it will continue to grow, it is not exhausted, it did not have any facility to get out. Some children died there, some of whom had 4-5 people, they had arranged oxygen from outside, the child was put oxygen and shown somewhere private.

16 When did father and mother (if working) returned to work after the death of the child?

Father: Baby was expired at 5th of month and father and mother went to their home after 5-6 days. They move there for the job and also rented a flat there. Bhabhi return yesterday after 10 days. She also residing at Noida flat

17 How your and your family's life has changed after the death of the child?

Father: There is sadness in everywhere and in every activity. Whenever anyone passed sadness over there.

<any substance abuse> No! The life gradually moves on the path as it was earlier. We are trying to resume the previous life style.

Knowing cause of death in detail- Autopsy and MITS

18 What was the cause of death of your child and what is your perception about the value of knowing the cause?

Father: They asked the cause of death in the beginning as well as contaminated water enters in the stomach and due to that the infection spread across the entire body and that why she passes away at 5th of this month.

<perception about the value of knowing cause>

Mother: you tell the reason of death; it would be helpful for us in next case. We knew that the treatment was not up to mark. She needed special treatment so the contaminated water/liquid could be drained from stomach. They should give some injection and should use cannula to drain the infectious. I hope in private hospital; all this could be happening.

19 In your view, what more could have been done to know the exact cause of death and the reasons causing the disease?

Father: As per our understanding, treatment was not proper. They just hanging Glucose in the name of treatment and make 3-4 times entry of same. If they visited frequently, the result could be different. She may be live.

20 Are you aware of any method for identifying the exact cause of death?

Sister in Law: Post-mortem was not done in our case. The problem was identified through x ray. Black spot over chest area was seen in x ray. They did not cure that black spot, and the case become complicated.

21 What are your views about the autopsy/ post-mortem for identifying the cause of death?

Father: As every incidence had happen in front of my eyes, I think post-mortem was not required. It would be required if the death was happened by poison or any suspicious reason or had a history of any rival with the doctor.

<religious aspect>

Father: No! we told the details from baby birth to death including all incidence like when we went hospital, when we get admitted, relieved etc.

Mother: Post mortem is not required in baby case cut/incision is not done in baby case.

22 What are your views about collection of the tissue (like done for biopsy to diagnose diseases) and body fluid samples for identifying the cause of death?

Father: If there were any provision like this, we may opt this. Even after death, one could able to know the reason of death, it is always a good option and everyone opt this option.

23 How parents and family members can be approached for the tissue and fluid sampling?
We were all together, so if anyone tells through the phone about this. Dead body was already there at the time of death so one can come and collect the sample.
<who should approach>
Any hospital staff may be detail about this, usually doctor or staff nurse explain this type of information. Obviously, it would not reliable if the information comes through patient side or from their relative side.
<how should they explain about tissue sampling>
It would be better to explain the process through mobile, a short video related to this via mobile.
<what time they should approach>
Immediately after death, it would better to do this process. May be on being able to get related information from the doctor like which baby/child expired and their other details. The relative and patient who are educated and wise must interest to know the detail reason of death.
Form this one can explain and share the cause of death with relative and all will know the area of ignorance that lead to death of baby. I think, everyone who is educated, will convinced/agreed for this test. Anyway, while treatment more than 50 times injections has been given to patient, another one injection after death wills not a matter of concern. Everyone will agree for this and will help in further plan.
24 In your view, what factors are important for acceptance of the tissue sampling by the parents and family members?
Father: This test would detail the exact cause of death so what type of ignorance has happened can be detailed out. This would also detail that what exactly could not be controlled in this case.
Decision making dynamics
25 In routine day to day operation who takes decision about the major activities (purchases, treatment, spending money) about the family?
Father: We are living here, so all the related decisions have to be taken by us. Like ration, accommodation etc.
In treatment case also, based on last experience and knowledge about which doctor would be better in this case etc. In minor complication, we usually visit local private hospital. Like there is private hospital “A Hospital’’, my wife visits there in minor medical consultation
26 During the hospital stay who discussed with the doctors/ nurses to make the decision(s) about treatment and other procedures.
Had got the admission done, treatment was done and it was already guessed that the child would not be cured, and if we tried to refer, we could show the improvement elsewhere, maybe by spending 20-50 thousand, Maybe the child would have been correct, it would have been good, but they did not write the referral, and our relatives who had come also told that other specialized hospital would not take any such admission with referral from here, If they referred, our baby get special treatment there. There is a treatment of all type happening here, everyone has to see, there (in child hospital) only have to see the baby, there is a baby hospital and one is only a children's hospital, that is Where there will be a hospital with children only.
Despite all treating all one has to see only special cases of mother and child. That system would be better. Here in S Hospital, the doctors have to see every type of complication.
<any contact over phone>
Father: we have seen the baby and realized that this is a serious case and would not be cured. We had called my cousin brother. He came at hospital and tell that until we get a referral card from here no one is going to entertain us.
27 After death of your child, who decided about the cremation and other post-death activities and rituals?
Mother: Everyone from our family, were there at hospital.
Sister in Law: The other family member who were at home, prepare lunch for us and bring at hospital.
28 Now, we are completing the interaction/interview. Would you like to add anything?
Father: What to say for this! There is no any scheme from Indian Govt.
Mother: Don’t know why baby has this much issue. Someone has cleft lip, someone has blocked anal passage. I have seen many children in S Hospital had severed medical issues and were admitted at there. In a small space, they had kept 3-4 children. There was no space for treatment.

In-depth Interview parents of deceased neonate-9

**1.1**	**Primary respondent (relation with the child)**	Mother, father
**1.2**	**Age of the primary respondent**	Mother-20-25 years<br>Father-20-25 years
**1.3**	**Other members present**	None

Events that led to death of the child

2. Can you please let us know about the illness and events that led to death of your child?

Had taken to S hospital for delivery, delivery took place in S hospital itself, discharged after 24 hours of delivery from hospital. Come back to home after discharge from hospital, keep child here for 8 days, 8 days later, the child latrines and does it again. After 5 days child stopped doing latrine, so will took the children to S hospital where delivery happened. Child was drinking milk and all (M)) child was drinking milk, child had diarrhoea (F). Taken there, they said go to S hospital you are not able to pay here (M). Previously we had taken to SJ hospital. Said there, bring 10 thousand rupees, will have to keep child for 20 days in hospital, it was 9 o'clock at night (F). So from there reach to S hospital, went to hospital at 9'o clock at night where doctor called me, I told the entire thing and told that the child din not defecate (M). Doctor also said that child have gastric problem, he did some treatment by putting pipe and send us to room number 18.

Hospitalization period

3. Now we would request you to tell us something about the period of hospitalization for your child?

<duration of stay> I, my wife and my uncle stayed for 8 days in Hospital.

<course of illness> No, not this all, will get well, Nothing, will tell them all think will be ok.

4. Could you please describe your overall experience about the hospital care?

<about treatment> told nothing, the blood kept coming out, they used to say it's okay and used to check, gives medicines, yes drip continued for 8 days, for 8 days, I was there for care, my wife and my brother also (M) we 4 people are there for care, I stayed at night (F).

<about investigation> Yes Check-up happened, nothing was in report, they told nothing, when I asked they told that all things would be fine.(F)

<about cost> stayed in S hospital for 8 days, expenses are about to 1000-15000, No-No, medicine was provided from inside from the hospital only, also took medicine from collage centre.

5. Could you please describe your experience about communication by the various hospital staffs during hospital stay?

<behaviour of HCP>

All are doing good, all nurses are new they are under training because when child specialist doctor come scold them a lot, doctor told them that what medicine are you giving, what is this, they all are new, Senior doctor told before two days of death of child that this child won't survive, doctor told before two days that poison has spread in the body, child won't survive, yes said that breath is not running, drive the pump, this thing told by the senior doctor (F) told that poison spread over the body it will need a needle (M). when child come earlier then he was okay, told that child is breathing, will have to did operation, I asked that I need to call all mother and father, if not survive then what will do, need to did operation, So had to do operation at same place (F). Then Operation took place (M), yes during that 8 days only, after operation doctor told that child become normal now but after that he told that now child will not survive (F).

<about behaviour of HCP> Behaviour of staff was good, all of them were good in term of behaviour, they take care timely, why should I lie.

<communication about treatment> Did not tell, did not tell anyone, they told that child is small, can become ill at any time. They told about treatment, idea was all right, stayed there for 8 days, suppose child was there but no one care, which medicine is given. Senior doctor told, one child specialist doctor comes at 12 o'clock at night he do, he is old but other doctors are new. (F)

Death and post death period

6. Could you please tell us about the death and death declaration of your child?

<cause of death explained> No didn't tell, I was at hospital on that time, child was dead at 6 O' clock at morning, after death doctor inject needle, still no body said that child was dead.
<who declared> No one told, he had Nimboi in his mouth, I started weeping and said that child was dead then free it but after that no one was freeing the child, when I called them then he asked for signature on various documents and left the body, no didn't open, yes it was revealed that child is no more (M). by seeing face it was knowing, take it 1 or 1 and half hour, if died at 6 o' clock then they give us at 7 o' clock, doctor said at 6 O' clock on that time, colour was changed, child was not breathing, body became cold, then go to doctor to give information (F). Doctor came and changes the piece that was in mouth.

7. What was your and your family member reaction to the death of your child?

<mother reaction> I started weeping.
<father reaction> No Reaction, returned to home from hospital after the child died. But my wife started weeping.
<did someone try to console you> No one when child was died no doctor was there.

8. How was your experience about the procedure after death till handover of the body to you?

<about documentation> Mother was signed
<time taken> One and half hour.

9. Who all from your family and community/neighbourhood were present at the hospital and how they supported you during this period?

Me, my wife and my Uncle was there in hospital, they supported a lot.

Post death procedure and coping mechanism

10. What all rituals and procedures did you follow after death of your child?

Reached elder brothers' home at 11 O' clock, 20 to 25 relatives come there and did the last rituals bury the body approx. 5:00 PM
<body preparation> Child body was buried by maternal uncle; 40-50 people were there during this.

11. How did you, your spouse (wife/husband) and immediate family members cope with the difficult phase?

We need to control our self, returned and start living and doing business.

12. Is/was there any change in the interpersonal relationships between you and your spouse and other family members after the event?

There is no change, but it will take time to cope.

13. How did the family members support you and your wife during the difficult phase?

They supported us, after hospital live at brothers' home for 4 days.

14. What were the reactions/ counselling and suggestions given by relatives/neighbours/ community after the episode?

Relative and neighbours said that why you go for treatment in S hospital, they don't treat well.

15. Do you think and/or discuss with your spouse/ family member(s) about the death and related events and blame someone/something or feel guilty for the same?

I blame myself I should not have taken the child to S hospital.

16. When did father and mother (if working) returned to work after the death of the child?

Started work after 4 days.

17. How your and your family's life has changed after the death of the child?

<substance abuse> No change.

Knowing cause of death in detail- Autopsy and MITS

18. What was the cause of death of your child and what is your perception about the value of knowing the cause?

Yes, it is important to know the cause of death it will help us to care for next child.

19. In your view, what more could have been done to know the exact cause of death and the reasons causing the disease?

I don't know because no doctor was present at the time of death.

20. Are you aware of any method for identifying the exact cause of death?

21. What are your views about the autopsy/ post-mortem for identifying the cause of death?

<about PM> Yes, I know, I have seen it, the head is open, chest is torn and the doctor checks what is he cause of death.
<religious aspect> This should not happen post-mortem is not allowed in religion

<disfigurement> yes

22. What are your views about collection of the tissue (like done for biopsy to diagnose diseases) and body fluid samples for identifying the cause of death?

<about MITS> Yes, this is right.

<religious aspect> Why does it make sense, no one gives guarantee because take it out from body. This is right.

23. How parents and family members can be approached for the tissue and fluid sampling?

<who should approach> Doctors

<whom> Father and mother

<method of explained> by oral explanation

24. In your view, what factors are important for acceptance of the tissue sampling by the parents and family members?

It's important to know the cause of death.

Decision making dynamics

25. In routine day to day operation who takes decision about the major activities (purchases, treatment, spending money) about the family?

Majority of time decision is taken by me only because in-laws are in village, sometime ask suggestion from brother on phone. (F)

26. During the hospital stay who discussed with the doctors/ nurses to make the decision(s) about treatment and other procedures.

I was in hospital, So I make decisions. (F)

27. After death of your child, who decided about the cremation and other post-death activities and rituals?

I was deciding about the cremation and post-death activities and rituals. (F)

Summarization

28. Now, we are completing the interaction/interview. Would you like to add anything?

What happens by diagnosis, just got information only, look, ma'am there was nothing when I brought the child to hospital he was playing but got very upset when drip was applied.

In-depth Interview parents of deceased neonate-10

**1.1**	**Primary respondent (relation with the child)**	Mother
**1.2**	**Age of the primary respondent**	Mother-18-20 years
**1.3**	**Other members present**	Nani (Grand mother of child)

The child was admitted in NICU. Mother of the child was staying at her parent's house after the delivery.

2.1 Can you please let us know about the illness and events that led to death of your child?

As the child birth took place in S hospital, so he was directly admitted to NICU.

Hospitalization period

3 Now we would request you to tell us something about the period of hospitalization for your child?

Nani: When baby was in the stomach, then she came here, started saying that no more time left to deliver, it will be here. I went to the hospital. The child did not breathe as soon as the delivery took place there. Mother- was born, was born well, Just, did not cry, neither cradle her. After getting admitted, I should have patted in reverse. Should have cried, delivery has happened at this hospital (S hospital). Where he took it, just went inside and admitted it, tested blood, and weighed two kilos and six hundred grams. - Water goes in the mouth of the child, the breath also becomes slightly up and down. Mother- If something gets stuck, it does not cry in the mouth, then cries after a while, they go straight and get admitted. Wrapped up. And he is saying that he got the phone on the phone and not a single missed call came on the phone.

I called my husband and said that the phone is not picking up. The doctors were lying. A missed call did not come in the phone that I had, the phone rang at 5 am.

It was up to 8 o'clock in the morning, and then admitted there. He said that your child has not cried. Was under developed, Was not small, was moderate, occurred throughout the ninth month.

Nany – when they kept in the ICU, where the children live, now they give milk, so tell them that you can feed the baby now. I tell you. The first time we went, we did not know that we would say go and feed the baby, (Mother - go on your own), then, she started saying that you feed the baby yourself, you will see yourself coming (Mother - there was no one to tell there) they could have told about all this, we don't know enough. We went for the first time, then said on the second-third day, after two-three days he said that your child has passed away.

<How long has your child been in the hospital>

Mother- Only one day. Delivered on the 21st, and then discharged on the 22nd, we had Eid on that day, Muharram was also on the same day.

<The doctor gave some reason>

Mother- No reason given, just sent. Granny- Did not tell anything, Madam, started saying take it away. Did not give any reason Just gave a paper and said take it away. And said to take the treatment paper, we said what the child will do when the child is no more.

4 Could you please describe your overall experience about the hospital care?

Mother- Blood test was done but did not tell anything about it.

I had given blood to bring a report. I went there, so gave the report to them, I do not know what I saw, what I did not see, we are not so educated, we are illiterate.

<Spent in S Hospital >

Mother - It has happened, it has happened a lot. From here my husband took four thousand rupees. I donate the blood. It cost to feed and feed him. Excess of expenditure was incurred, then after taking the child, his clothes came with everything which was kept in the hospital. I had to throw all the clothes that were kept at home. There was such a situation there in hospital, only the stomach was being pressed, looking up from the top of the body, by checking up, when a child is in the stomach of a human being, it is not pressed so tight. He was pushing hard, shaking, which was wrong. The delivery was normal. There is a problem when there is a first child.

5 Could you please describe your experience about communication by the various hospital staffs during hospital stay?
<when child was admitted what they used to communicate>
Mother- They did not say anything; they spoke just fine. Nani - First they said that she is breathing now, then it will be alright, then later on they started saying, there is no improvement. After that they started infusing glucose. Somebody said that he is having epilepsy. Don't know what was happening seizures were coming, then giving medicines, Sometimes injection,
Mother - they are lying or the truth, I do not know. The hospital was in a bad condition.
Mani - I could not understand anything. First started saying that he has started breathing, baby is in normal condition, then after a couple of hours he started speaking something else, then started saying that now the child has died, take it. They Signed on a paper and then gave the dead child.
<When the child is no more, who was he with>
Nani- There was no one there, the child was in ICU. We were not allowed to go to the doctor.
<Who Calls from ICU>
Nanny- Doctor, inside ICU, the doctor made the call that the child is no longer there. In Mother-ICU, number five was kept in the ward.
Death and post death period
6 Could you please tell us about the death and death declaration of your child?
<sympathy>
Yes, he started saying that now there is no child, he just said this and nothing else. Then he said that if your child was alive, he would be lazy, he would remain fascinated, he would continue to read such a lame, if he is such a living, what life would be like.
Mother - giving excuse like this, The doctor was speaking all this, the doctor who was recruiting all the children, he was speaking the same thing.
Nani – they were non trust worthy
Mother: It was my first baby.
Nani - When the child is breathing, you will live, now you speak like this. I said that now there is no use, the child is here, it is not even alive, so the children are then asleep, we were helpless,
Doctor said do all formality and relive the dead body.
<doctor behavior>
I felt bad at the doctor's behavior, very bad. If the doctor was good, my child would be playing in his lap today. Nanny-Lady's behavior was very bad. Mother- She was speaking as if she had never become a mother herself. As the doctor was behaving there it was very bad.
Grandmother- used to behave very badly.
Mother- She was also screaming during my delivery. My first delivery was At first, it was hurting so much. The incision was being made, the scissors were cutting the skin. I was saying that I have my first child, then said that you should do everything yourself, hold your feet yourself.
Nany - Some people do this.
Mother- I was holding her leg myself, at that time she has to bear pain and hold their feet and manage all. How difficult is everything at that time. And that was my first experience, I didn't even know about them all.
Nany - It is better to have delivery at home. The midwife gives help, but, at hospital, there is very bad situation.
Mother- Stitches are being put all over the on the body, it seems like, I became not pregnant again after that incidence. Who can one behave like her.
<nurse behavior> Nani: The nurse drives him out, does not allow him to enter.
Mother- The nurse used to treat people like dogs, tell me! If someone will admit a daughter, then relatives will come. So what should humans do Never let anyone live more than one. Someone will die alone inside. Their reputation was so bad.
Granny - I was not allowed to go inside; she was driven out. The guards used to drive everyone away. The nurse used to chase them away.
Mother- Now I have thought that there is no need to go to the hospital again, even if the person dies in the house. Who will go in such a situation? Nani- Madam, I used to hear from other people that there at hospital one get proper treatment, but after going there I saw very bad.
7 What was your and your family member reaction to the death of your child?

<Who narrated to you>Mother- My husband did not tell that the child died in the hospital. He said that he has injected. Still sleeping Will get up later, bring home like this. Then it was later revealed that the child is not there. Then I lost my life.
Nani- What would you say there? Bring home from there. Weakness had become so anyway, telling about it was a big setback, so I said to take it home. Then it will tell that the child has passed away.
Mother- Did not tell me that the child is not there. I was very weak.
The grandmother was weak and she was feeling dizzy. I brought it with me.
<What was the reaction when the doctor told no child>
Nani - The child was given in the hands of my husband.
<father response>Nani - Everyone was crying. The woman's cry was heard by everyone, not the man's. He was happy at first, then sad. Mother- When he heard that the child was born, he was happy, told his friends, told his housemates, then when he heard about this sad news, become client, not prefer to say anything. He would not cry in his mind, that even my first child was not left. Where the hospital considers good, it turned out to be so bad.

8 How was your experience about the procedure after death till handover of the body to you?

Mother- It took a long time to handover the child dead body. Nani- they were doing lot of paper work and getting signed, it was 7 o'clock in the morning.
Mother- The doctor called at 5 oclock in the morning.
Nani- In the morning, they sit down to drink tea. At the same time called.
Mother- around 8-8:30, they complete the process and handover the body
The child died at five o'clock. Dead body returned at 8 o'clock. When he left at 5 o'clock, he said that he is breathing a little.
Nany- Because of not being doctors, they were lying in giving bodies. I called my husband. When the child was dead, they were not handover the body. What will you guys do with it? He said that he will wrap you in cloth and give it to you.

9 Who all from your family and community/neighbourhood were present at the hospital and how they supported you during this period?

Nani: My husband and Mine with grandmother will be there.

10 What all rituals and procedures did you follow after death of your child?

Mother had brought her home (nani place) from the hospital.
Grandmother - had brought here only. After bath, was buried as he is buried in his Muslim.
<Azan read at the time >
Nani: No! he was there in the hospital. Who will offer blessings there Like I do at home, I give love to a child.
<How many days do you give azan>
Mother- Just like, after three days, the hair wall is cut, after the sixth class, then they offer azaan. Because when the pak gets cleaned, baby, when the pak is not cleared there, what will a human being give there? Had taken the child to Nahla at home.

11 How did you, your spouse (wife/husband) and immediate family members cope with the difficult phase?

<rituals after death>
Mother- Yes, I did not do anything for a month, my condition was bad anyway, under the tension of the child, in tension anyway I went mad with Roh-Roh, one month remained like this for two months. Then go to your senses, what to do now, when Allah kept it, now tell me what to do, now by crying, singing, leaving food, now it will not come to me. So I thought, now slowly, slowly, now slowly, I have held my senses, when the ninth month was when it was done, then it broke from the tenth eleventh month.
<coping mechanism> Mother- How to emerge, how you are coming out, my mind is that how I am like, because I will not be my first child, how will I get out of there, sometimes sitting in sadness, sometimes thinking, that Maybe I too would have children, now what should Allah tell me.

12 Is/was there any change in the interpersonal relationships between you and your spouse and other family members after the event?

Nani- Its man is right, he explains it. Mother- My husband is very right, he says that there is no problem, it is not there, it is not right, you are there or you will be enough. I was cheering me on, where did I go?

13 How did the family members support you and your wife during the difficult phase?
Mother- Mom, my father, my brother-in-law, father-in-law, since this was normal delivery, every one counsel me, never mind, don't worry, everything will be fine.
14 What were the reactions/ counselling and suggestions given by the relatives/neighbours/community after the episode?
Mother- What will everyone say, how is it that what has passed, what has happened, what to do now, tell everyone what had happened or the speaker thought, what the event was supposed to happen to me. It is done, now no one can bear me for a long time. <any reaction / suggestion> Nani- no one gives advice.
15 Do you think and/or discuss with your spouse/ family member(s) about the death and related events and blame someone/something or feel guilty for the same?
<do you blame someone for this event>
I consider the doctor guilty. Granny- Does anyone believe a guilty person, will blame their fate. Mother-man considers her luck to be the blame, and will not consider anyone guilty, what can anyone say now. If our luck is bad, what to do, what we have done wrong (S Hospital).
<whom do you blame from hospital>
Nani- He will know exactly who has a child in the hospital, now who will speak there, now there is a lot of child being born, now this is happening to everyone, someone says that by taking money. Mother- The doctor should tell the right thing, sometimes he is saying that he has got it right, now he is breathing, sometimes he said that epileptic tears are coming, what to do now. Granny- It is a big deal for a big man, a person who writes and knows, what the illiterate knows, just as they sit where they sit, sit down and tell them later, this is what happens. Well educated people understand the matter, why do they do such poor things?
Mother and Nani both crying during speaking
There (S Hospital) there is less of freedom, they are getting a job from the government, they are getting notes, then it is a job of self-interest, poor poor is dying, let him die, what does that mean to him? The Mother- Do not do this, you should treat everyone as equal, whether Muslim or Hindu, a woman is a woman.
16 When did father and mother (if working) returned to work after the death of the child?
Granny works in office, Private. Mother- You get something to pick something up, pass it, still working in the same company, to order someone or to speak to someone, they are calling,
<returned to work>
Nani- Ever since he was with his parents, he has been doing this work. Mother- The child has been working ever since he was finished. Did not sit at home on any day. She is discharged on Sunday, but she took leave that day. Because we reached there at night on Wednesday. Granny- son, now it is a matter of where the man will sit at home, will work even at home, now he will not sit with sadness broken. Mother- I started doing household chores after a month or two.
17 How your and your family's life has changed after the death of the child?
No Such change has seen
Knowing cause of death in detail- Autopsy and MITS
18 What was the cause of death of your child and what is your perception about the value of knowing the cause?
Madam, who will take such a tension, now who will go there and ask. <value of knowing the cause> Mother- I knew everything when there are so many presses in my stomach, there are so many fingers in the place of the body, I knew at the time that my child was no longer there. And how are you going to get out from there? You are not letting it out from there, when it was admitted, it was admitted, now the shadow is dead, the shade is normal, it is from the operation, the child has to go from here or not Left. What should I leave, no one is going to leave, my mother is not allowing me to come, how to get out of there, and to whom I speak, how do I tell my mother, I am not letting you come there, kept me inside. Is recruitment.
19 In your view, what more could have been done to know the exact cause of death and the reasons causing the disease?
Mother: How would I know this. No !
20 Are you aware of any method for identifying the exact cause of death?
Mother :don't know

21 What are your views about the autopsy/ post-mortem for identifying the cause of death?
<What is your opinion about post mortem>
Granny - Now buried, how will the post mortem. The post mortem is to be done by him, that has not happened.
<In which case post mortem is done>
Mother- In which case post mortem is done, we do not know.
Granny - We do not know all this.
<What happens in post mortem>
Mother- What happens in the post mortem, they do not even know.
22 What are your views about collection of the tissue (like done for biopsy to diagnose diseases) and body fluid samples for identifying the cause of death?
Mother- Now this is the point that there is no one to tell there, went for the first time, how would we know, there is so much crowd, no one is telling anything, there everyone is busy with himself.
<What would the doctors do if they told you>
Mother- Yes, I would have investigated him, so that we could know the cause of death
<religious aspect>
Granny- don't know, no we don't get it done, leave it to God,
The one who is doing it is God, the one who gives it and the one who takes it,
Now get the post mortem done, do a blood test, who has died from which disease, there are lot of disease, how long will the person keep checking, now where will anyone show it, who will see which doctor, which doctor will tell. Mother: Some doctors drive away.
Nani - Now what is the disease, what is not there, who will be so upset, what happens in the destiny, what to do now, the information is there where children are born in the hospital, there is no one willing to tell and no information from whom. Will take.
Mother- Now I do not understand that something must have gone wrong in the mouth of the child, then he would not have cried, at first, it gets stuck on the stomach, the breath is moving, this is how I feel They said that they have passed, in the first place they are saying that they are not crying, then they went and admitted that their beating was going on, then after two-three days they said that now it has passed. is. Until the child gives Roh too, now he does not know what he did with him, he knows something. <mother of the child asking her mother> Mother- you don't know, they will know who they have done with her. Nani - What to do, what not to do now
23 How parents and family members can be approached for the tissue and fluid sampling?
Mother- No such person is found there (S Hospital) who will explain this, there is no one there, and no person looks good there.
Nani- Madam, ask the people outside, who are lying outside, take information from them, they will tell everyone.
Mother - who lives outside the hospital. How the days are passing, their condition, how they are, they tell everything. How doctors are not listening, how to do it. Nani - do not let go in, how are one can standing with tea, and ignore the patient and relative. When ever ask something from them, they ask security guard to get out us.
ing cut, Madame is driving out, she is the watchman, security guards tell everyone how they do. They are poor, they are their children, they tell them everything. The one who passes over it tells.
24 In your view, what factors are important for acceptance of the tissue sampling by the parents and family members?
Mother: we opt this to know the death reason. But, nothing is possible this time.
Decision making dynamics
25 In routine day to day operation who takes decision about the major activities (purchases, treatment, spending money) about the family?
Mother: My husband and mother in law take a call for all things
26 During the hospital stay who discussed with the doctors/ nurses to make the decision(s) about treatment and other procedures.
Mother: My mother said that you can manage the delivery done at home, you would stay at home, the midwife would also help. I thought that my mother-in-law would blame on me if anything wrong had happened, so I thought that the hospital would be well supported, there is a lot of caretaker,

It also helps. I did not know that would be so bad with me. Yes, it was my decision to go to the hospital for delivery. Now I came to know that all this will happen, I do not have to go ahead from there.

27 After death of your child, who decided about the cremation and other post-death activities and rituals?

Ritual bath, in the last was done by my father and my husband, all the family of my brother was there (burial ground) to go to burial.

28 Now, we are completing the interaction/interview. Would you like to add anything?

Nani - Only when we have any information, we will tell you. What is not known, how will they tell? They only say that the child has died. They directly say that if this treatment is to be done, get it done or go back home. Do not let anyone go inside. Do anything. The nurse should help, but they do not do it at all. Whether the first child or the second one treats everyone badly. They should improve their speaking.

In-depth Interview parents of deceased neonate-11

**1.1**	**Primary respondent (relation with the child)**	Mother
**1.2**	**Age of the primary respondent**	Mother- 18-20 years
**1.3**	**Other members present during the interaction**	Mother-in-law

During the interview mother of the child and grandmother was present. The mother delivered twin babies at S hospital. After the delivery both mother and child were admitted in KMC (Kangaroo mother care) ward. One child is alive and healthy.

2.1 Can you please let us know about the illness and events that led to death of your child?

Mother-in-law: Both were twins. Mother- The problem was nothing. He was fine until morning. Mother-in-law, we came from the hospital in good condition. It was a healthy upto 10 days at home and I used to sleep with him (mother). Now she (mother) was feeding. I came to cook breakfast with the child. After keeping household garbage at outside, he had some difficulty breathing in belching, and then he (2nd child) started crying and then started feeding him. Now when he saw him sleeping, his (1st child) breath had stopped breathing. My son came to speak then immediately we took him to M Hospital because we had enough work time and did not take S Hospital. It was a child's thing we thought that what would happen would be seen. Then got the child admitted here. They were saying that there was difficulty in breathing. We will recruit it here. We will keep it admitted here (M hospital). It will take 25 days and 60 thousand rupees a day. So we said then you will guarantee for the health of the child, if you take 60 thousand rupees a day, then it is M Hospital, so I said who was preparing the paper for its discharge, it was good ladies. He told me that the child's condition is very bad. And if you take it then it is good otherwise this (M hospital) people will make you money and will do nothing. It was good that the one above prayed that he told the truth, then that thing sat in my mind, then the doctor said that it will have to be kept in nursery for 25 days the cost is 60 thousand a day. So I said Doctor Saab then you will guarantee me, then the child's my child is fine, to say that the guarantee is nothing, then the ladies told me first, who was going to write, yes, then I said when not taking the guarantee So, what should I administer, then I will go to S Hospital by taking it (1st child). Then called the S Hospital, the staff asked us to bring the child here. He had given us the number the KMC ward had left us by car, so he had said that no one can see you call us. We were in a hurry at that time, went there early, so he made a maximum hospital bill of 12 thousand within an hour and a half. Put the child on the machine for one-and-a-half hour, there was no movement in the child, pump the child, so the child used to move with it. Then after that, he called the S hospital and said, "Bring you here," the doctor at the nursery said, "You bring the child to the child." Then we told them (M hospital) that we will leave our child with S Hospital and then start saying, "First you ask the bed that the bed is not empty then we will give it to the child." We called again, so he (S hospital) said, we tell you in 10 minutes, then he called after 10 minutes there, then he (S hospital) said yes bring the bed empty. Then we told them (M hospital), give us our baby, we will take it, then started speaking to bring an ambulance. Then there is M Hospital here, there was a person to know, then he called him and reached with an ambulance. Then do not say that the child will go in our ambulance, the child will go to our machine, our doctor will accompany. Then he returned the ambulance, then he called his ambulance by phone, then he took him to the ambulance, and the doctor went along, the doctor kept doing the same pump.

Then when we went there, in S Hospital in an emergency ward, then we had a doctor. Then those who had come to leave the KMC had given the number to call the same number, called the same brother and said to them then they said to the doctor that they have taken the child. so doctor said, you do not come to remove the child from the machine, I come in the vehicle (ambulance), then I saw the child in the car, then I saw nothing in the child, no breath, then those doctors Some talk with each other, we talked among ourselves in English, we could not understand. Then those who have taken in that they go inside and admit, see, the child can be right, so I hope you know what it is said that it will be alright, then there he played 4 like that, kept it on him M Hospital's doctor came back to admit the child on the machine (M hospital doctor).

Hospitalization period

3 Now we would request you to tell us something about the period of hospitalization for your child?

Then put pipes in his nose, it was too late, four o'clock in the morning, 8.30 to 4. At 10 o'clock we had taken him to S Hospital, and reached there at 10.30. There was no movement in the child in such a long time, as he had brought the child it was the same since morning. Then we told the doctor to tell us the truth, do not deceive us. We too have children; I also have three children. Nothing is happening in the child since morning. Then he said that we tell you after 10-15 minutes. Say at 5-5.15 takes away the child. S Hospital went for 10.30 and discharged 5.15. It was said that take the child. But the doctors with the nursery had spoken in the morning with a view they said that there is no hope in the child. S Hospital's nurse said on seeing the vehicle saw the child in the ambulance and said nothing in the child. Then do not know what happened, the doctor talked to each other and then assigned him. Both children were born in S Hospital, meaning I had taken it for check-up, the day of check-up was in S Hospital, by the way, there is MM hospital. It was running in it, all the ladies write the same name when it is time for delivery. Then he (MM hospital) told us that you have two children, you can be seen in a big hospital sometime later, you came to the hospital a month ago, he wrote the name (S Hospital). Is then after a month we called, then we took it, then when it was taken with it, I saw it and said, admit it. There was only one day left for 8 months; 30 months would have been completed in 8 months. Then bid admit, it can be a child anytime. We admitted that to him then he said, wait for the blood, then the boy also gave a bottle of blood, then the child was born at 2.30 in the night, one was HOA at 2.35, the other was HOA at 2.40, both children were poor and weak. Then the doctor called me when he was born and said did you know that I had two children, I said yes, I knew that the hospital people of Malivya Nagar had said, this is the paper. So said, look, the children are weak, so they have to be kept in the nursery, so I said it is okay, the doctor will speak for the right, then they have admitted in the nursery. Then there was a child in the nursery, then after the nursery, gave the child in the KMC ward, first they used to go to the nursery to feed, then the child in the KMC ward would tie it to the chest in the KMC ward, then they were discharged, then came at home. The child was also fine for 9-10 days. At home, there was no visible.

4 Could you please describe your overall experience about the hospital care?

Mother-in-law: There was no fault of the hospita. He did not take any carelessness. He did very well, in every way the child was right, but his life was so much. So what will I blame someone, the child came right from there.

5 Could you please describe your experience about communication by the various hospital staffs during hospital stay?

<when you stayed in hospital from morning till evening, what doctor were telling you>

Mother-in-law (M hospital) said, the child is having trouble breathing, this hospital person said that he is having trouble breathing while weaning, like he fed (1st child) then He did not belch, thought that the child was asleep, the other one started crying, I came down to make breakfast. This (mother) stays up, so in so long she thought it was a sleep let's first give it yellow it also started crying. When it turned yellow when he saw him (1st child), he was dead, he died at home, when the child stopped breathing at the same time.

Death and post death period

6 Could you please tell us about the death and death declaration of your child?

<When doctor came to you at 5 o clock what did they told you>

Mother-in-law: Went out of the house from 8 in the morning, stayed in M Hospital for two-and-a-half hours, we reached here (S Hospital) at 10–10.30, they accepted, by five in the evening. No child will be cured, so you answer us, but we said, but then he said that we tell you in 10-15 minutes then he said.

<The child was put on oxygen>

That machine which is light, the machine was just on it. And he did not do anything, no medicine, no injection, nothing.

7 What was your and your family member reaction to the death of your child?

<reaction of father in hospital as mother was not present in the hospital>

Mother-in-law: Was very bad, in the hospital itself

(Mother crying during interview.)

8 How was your experience about the procedure after death till handover of the body to you?

Mother-in-law: It took a while, gave the discharge paper everything in hand. It did not take much time for all this work. After that he had brought home. They said that they pack the body in polythene we said no, they will be taken in clothes. Then we brought home

Mother-in-law was also crying during this question.

9 Who all from your family and community/neighbourhood were present at the hospital and how they supported you during this period?

Mother-in-law: I was in the hospital; his grandfather was also there. His father was also there, he had not taken it (mother), because he had a small child, he said (grandfather), do not take his mother and go, the younger child is afraid of infection. We were 5-6 people. He also had a mother.

10 What all rituals and procedures did you follow after death of your child?

Mother-in-law: They brought home from the hospital, many people came to the house, our family, and everyone came from the village. Then went to the crematorium.

<rituals>

Came home with the baby and bathed him

Mother- Mummy had agreed, the doctor said that until the baby is 2.30-3 kg, she does not have to take a bath. When he came from S Hospital, he was not even taken he took the cremation ghat directly. In it my husband and father-in-law went there.

<Something was worshiped in the house after that>

Mother: There was no worship, nothing happens in the case of children.

<procedure>

Mother-in-law: Just like this for 4-5 days, people came from her mother's house, my girl had all come, had a child, now she has another child so it is important to see everything.

Mother-in-law, I had a bhandara here. There is also kirtan in my house every month I thought that just normal delivery should be done whether it is boy or girl.

Navaratri was also going on, so I thought that Bhandara will do it, many people will get Prasad. Then when we came to the house, I went to Oh Pandit, that our children are fine, both, there is no Prasad Wagaira, even after the arrival of the hospital, I was 18 days later, I thought I will do the bhandara, when the name Karan Will do it, will set the time for the fifth Navratte in October, but then the child was finished on the 24th, then after that we went to the Pandit, so we said, now tell me what to do, so to speak. , Navratte is coming, Mother's gift was her own He said that his life was the same, the second child is right with the blessing of the one above, so please store it. So on 14 October, we have a further temple, there was a bhandara in the temple, distributed 700-800 man's offerings, then even in his name, 21 days had passed.

11 How did you, your spouse (wife/husband) and immediate family members cope with the difficult phase?

Mother: Now I will take good care of this child.

12 Is/was there any change in the interpersonal relationships between you and your spouse and other family members after the event?

Mother: My husband used to take care of me from the beginning, now he has started taking care of me and my child a little more.

13 How did the family members support you and your wife during the difficult phase?

Mother: Everyone was there, Ma, aunt, grandmother other and from here my aunt-in-law was mine, and all the others.

14 What were the reactions/ counselling and suggestions given by the relatives/neighbours/community after the episode?

15 Do you think and/or discuss with your spouse/ family member(s) about the death and related events and blame someone/something or feel guilty for the same?

Mother-in-law: No, we don't blame anyone, not anyone.

All I say is that God gave two toys for just this day.

16 When did father and mother (if working) returned to work after the death of the child?

Mother in Law: He started leaving after three to four days.

17 How your and your family's life has changed after the death of the child?

Mother in Law: No, nothing happened. Everything is the same.

Knowing cause of death in detail- Autopsy and MITS

18 What was the cause of death of your child and what is your perception about the value of knowing the cause?
Mother-in-law, we have to know the reason, yes, we wish. How did the breath suddenly disappear?
19 In your view, what more could have been done to know the exact cause of death and the reasons causing the disease?
Mother-in-law: You tell me you are a doctor; we don't know about it.
20 Are you aware of any method for identifying the exact cause of death?
21 What are your views about the autopsy/ post-mortem for identifying the cause of death?
Mother-in-law, understand from Post Mortem that if someone is a big man, ate some poison, then he should have it, this child was innocent. Don't want to be in the child, Bikul was such a child, what would be his post mortem, doctor, I did not even allow him to pack in polythene, I took my child like this. Now what would you have packed him, even if you have seen here, otherwise he would have packed it and given it like this. We brought our house by auto. The cremation ground is here and went there. Because the time was too much, it opens till 7 o'clock, then the rain also came. Then his grandfather went there (shamshan ghat) but then he told him that Panditji is an innocent child, now how will we keep in the house at night, it will be a small child, so we came here by 6.30 pm Got auto by 5.30, then came here, it took an hour here to 6.30, then he (panditji) said, bring it. Dug the hole for so long, then there were many people from here.
22 What are your views about collection of the tissue (like done for biopsy to diagnose diseases) and body fluid samples for identifying the cause of death?
<religious barrier>
Mother-in-law: would have agreed to this technique, which they did not say anything about.
<religious barrier>
Mother-in-law: There is no problem on behalf of religion.
23 How parents and family members can be approached for the tissue and fluid sampling?
Mother-in-law: Telling me or telling my grandfather, if there were any, I would definitely consult among us. In this matter, we would be pleased to say yes.
<in hospital who should approach>
Mother-in-law, as the doctor tells you it will happen, as if you do not know how someone has stopped breathing, you will know that if we do this. So we would say that it is okay, even for the future, we would have knowledge (knowledge) because of which death has happened, it was supposed to happen.
<how to explain about this technique>
Mother-in-law, even if they do not speak to us like this, do not see that you want to know how we will stop the child's breath, we will do this, he would have agreed to what he said. Had to break it even further
24 In your view, what factors are important for acceptance of the tissue sampling by the parents and family members?
Mother-in-law: I can't say anything about this. He came right from the hospital. The child had no problem. He got good care in the KMC ward too, all was good and there was no showing.
Decision making dynamics
25 In routine day to day operation who takes decision about the major activities (purchases, treatment, spending money) about the family?
Mother: The expenses are decided by my mother-in-law.
<decision about treatment> Who to take to the hospital, who has to be seen, all of them also do my father-in-law.
26 During the hospital stay who discussed with the doctors/ nurses to make the decision(s) about treatment and other procedures.
Mother-in-law: The one who becomes the ladies inside the hospital, where they said that to get the child's paper made for admission, we said, "Okay, let's get the admission papers made, I was also with her grandfather. There was a ladies' south -Indian was mean, you don't know what came in his mind that there is nothing in the child, it was understood that money will be made, we live here, our so is home, how many people stay in M Hospital, Even after selling everything without interruption, nothing happens to the patient. Yes, it was my and my husband's decision, he (S hospital) spoke to us, the nursery ward staff told the KMC ward that anytime the child is seen, you should bring it directly

here (S hospital). But when I saw it at home, it was nothing but breath means the child was nothing, at that time. At that time, I saw M Hospital in front of me, how could my child survive.

27 After death of your child, who decided about the cremation and other post-death activities and rituals?

Mother-in-law: The child was not bathed. We were all householders. There is a cremation ground here in the nearby village, the same thing happens to all elders, children and children. They burn the elders, bury the children. The priest is there, takes 500 rupees means then takes the child from the house in a new cloth with a cloth and buries it there.

28 Now, we are completing the interaction/interview. Would you like to add anything?

Mother: If the cause was known, then it would have been known what caused the death. Mother-in-law, did not mention this tissue sample to us, did not mean that the child is like this, it is okay that it was done, but you say that you should do it like this, if at that time we would have done the same. As soon as the injection is known by the class, it becomes known. He did not mention this to us, just said take away the child, nothing else.

In-depth Interview parents of deceased neonate-12

**1.1**	**Primary respondent (relation with the child)**	Mother
**1.2**	**Age of the primary respondent**	Mother’s age 30-35 years
**1.3**	**Other members present**	None

Context-

Events that led to death of the child

2. Can you please let us know about the illness and events that led to death of your child?

This was my first child. He came here to get treatment. Previously used to live in Calcutta. Due to this my husband lost his job. The child was shown in S Hospital. It was not seen by the eye. The man did not recognize and did not even walk. He was just five years old. He who has just died, died two days after birth. Stayed in the hospital for two days after its birth, then brought it home. Then the health of the child started deteriorating and went to the hospital with him again. He took her early at 4 in the morning. One day, he was hospitalized and died the next day in the nursery. At around 4 in the morning my child died.

Hospitalization period

3. Now we would request you to tell us something about the period of hospitalization for your child?

Yes, I and his father were in the hospital. One child was delivered at home and one was at S Hospital. Everyone said that this child should be treated well. Before that my child who had died was also in S Hospital. This time also the heart check up and all other tests were done earlier. My child was not treated properly. This time it was sent after a little investigation. We have come home. After feeding here, foam started coming from the mouth. The heartbeat was accelerating.

4. Could you please describe your overall experience about the hospital care?

<about treatment>

Went to the hospital. I had tried my best. Had an x-ray done, a blood test was done. A day and a night after getting admitted, a senior doctor came. Seeing that, quickly get it done by x ray. After checking the empty blood, the doctor was waiting for the doctor to see if the doctor would come. Later put it in the lead and injected it into the vein everywhere. We did not tell anything in time, if we were quick, we would have taken the child to another hospital.

<about investigation>

The investigation was correct but nothing was known from it. There was no blood in the body.

<about cost>

Did not take money in S Hospital, did not take it there.

5. Could you please describe your experience about communication by the various hospital staffs during hospital stay?

The child had to pipe to breathe. The doctor had come and seen well in the first child. This time neither saw the investigation report, nor x ray, just discharged. The behavior of the doctor and nurse was not good. The treatment was also not done properly. People used to say that your child's condition is not looking good but the doctors did not tell anything.

Death and post death period

6. Could you please tell us about the death and death declaration of your child?

<who declared>

I did not know that who is a doctor and a who nurse. Somebody used to come and do some investigation and would not even tell. A senior doctor came and told the rest of the people that they should do this, do not do this. The child was not breathing. Still do not know what you were looking for. Did not tell us anything. My husband used to tell me that I do not understand what is happening. Those people were lying. Was saying that keep giving medicine.

<cause of death explained> Did not tell anything. Someone said that he has a heart disease. Nothing was found.

<sympathy/empathy> not given

7. What was your and your family member reaction to the death of your child?

No, the child is dead, take it. The father made his discharge paper and said, take it and get it signed. All the papers and medicines were with the doctor. His hands and feet were all swollen. Showed the doctor in Govindpuri. At that time my condition was very bad.

8. How was your experience about the procedure after death till handover of the body to you?

<Time taken> Signed in immediately. It was 12 o'clock here, 12 o'clock in the night.

<cost or payment> No

9. Who all from your family and community/neighbourhood were present at the hospital and how they supported you during this period?

Yes, he had called the neighbour, his name is Kishan. He had gone to his village, called him. Husband had called, called, helped, everyone brought the child. Over here everyone had left the child. Papa came with 3 people. Everyone helped.

Post death procedure and coping mechanism

10. What all rituals and procedures did you follow after death of your child?

The child was dead at 7:30, so we had gone at 12. First came home, then took it away. It was raining heavily. The child was kept outside. Was buried on the banks of river Yamuna, near Kalindi Kunj. About 10 people came. My relatives, who live in Ludhiana, had called them and called them. There was no worship on that day. Everyone had taken the same there.

11. How did you, your spouse (wife/husband) and immediate family members cope with the difficult phase?

12. Is/was there any change in the interpersonal relationships between you and your spouse and other family members after the event?

Nothing change !

13. How did the family members support you and your wife during the difficult phase?

No ! we had called one person and went with him

14. What were the reactions/ counselling and suggestions given by relatives/neighbours/ community after the episode?

Nadad and other relatives came and they were giving some suggestions and they understood those people stayed for 2-3 days. Their boy brought them. They say that what was to happen has happened, do not worry about it. Do not worry about that

15. Do you think and/or discuss with your spouse/ family member(s) about the death and related events and blame someone/something or feel guilty for the same?

The guilty do not understand anyone. As if it had done x-ray and blood tests, it would have been known as to why the child had died because of this. Only said this is heart disease.

16. When did father and mother (if working) returned to work after the death of the child?

Now ! By the way, he started working after 3-4 days, started work after 3 days.

17. How your and your family's life has changed after the death of the child?

Yes! Now more attention is paid to these children. We take him to the doctor only if there is some problem. Think that if he had taken the child in the night, it would have been right, he was late at home. Husbands do not have a bad habit such as drinking alcohol or cigarettes.

Knowing cause of death in detail- Autopsy and MITS

18. What was the cause of death of your child and what is your perception about the value of knowing the cause?

Should know we were told by the doctor that he had a beating disease, so he has died. If someone asks us how the child died, they cannot even tell.

19. In your view, what more could have been done to know the exact cause of death and the reasons causing the disease?

There is no information about it told me that he has a heart disease. Then it is over.

20. Are you aware of any method for identifying the exact cause of death?

<about post mortem> No ! don't know about that

21. What are your views about the autopsy/ post-mortem for identifying the cause of death?

22. What are your views about collection of the tissue (like done for biopsy to diagnose diseases) and body fluid samples for identifying the cause of death?

<about MITS> I am telling so many people. It would have been good to know the reason. Right now I have incomplete information about how my child died.

23. How parents and family members can be approached for the tissue and fluid sampling?

It is right to tell the doctor, but they do not even speak what has happened? How has it happened?
24. In your view, what factors are important for acceptance of the tissue sampling by the parents and family members?
Decision making dynamics
25. In routine day to day operation who takes decision about the major activities (purchases, treatment, spending money) about the family?
26. During the hospital stay who discussed with the doctors/ nurses to make the decision(s) about treatment and other procedures.
Its father! Used to do all the work
27. After death of your child, who decided about the cremation and other post-death activities and rituals?
Do not bathe him. We all kept weeping and went away like this. By the way let's bathe. Old people in the house tell all this, bathe it and wear it. Kafan wrapped him and took him, his uncle had brought him. His uncle had taken him under wraps. Not bathed is considered not washed. Mane was left in a weeping state the bathing is in the wash it was my pleasure that the same 2-4 men took it away.
Summarization
28. Now, we are completing the interaction/interview. Would you like to add anything?

In-depth Interviews –Healthcare Providers at Hospital

S. No.	Participant	Occupation
1	Participant 1	Doctor 1 (Pediatric Ward)
2	Participant 2	Doctor 2 (Pediatric Ward)
3	Participant 3	Doctor 3 (Pediatric Ward)
4	Participant 4	Doctor 4 (Newborn care unit)
5	Participant 5	Doctor 5 (Newborn care unit)
6	Participant 6	Doctor 6 (Newborn care unit)
7	Participant 7	Nurse 1 (Newborn care unit)
8	Participant 8	Nurse 2 (Newborn care unit)
9	Participant 9	Nurse 3 (Newborn care unit)
10	Participant 10	Nurse 4 (Pediatric Ward)
11	Participant 11	Nurse 5 (Pediatric Ward)
12	Participant 12	Nurse 6 (Pediatric Ward)
13	Participant 13	Support staff 1 (Pediatric Ward)
14	Participant 14	Support staff 2 (Pediatric Ward)
15	Participant 15	Support staff 3 (Neonatal care unit)
16	Participant 16	Support staff 4 (Neonatal care unit)

In-depth Interview- Doctor (Pediatrics) 1

1. **Basic demography**
   1.1. **Designation** – Specialist pediatrician

Service delivery and communication

2. **Please describe your role related to the clinical care for patients and types of patients seen by you.**
   We see all types of patients, examine the patient, confirm the diagnosis, talk to parents, we tell them regarding diagnosis, explain the prognosis, especially to more sick child. The type of patient usually seen are from birth to 12 years of age, all type of diseases is seen.

3. **You come across several patients on daily basis. How do you inform and counsel the parents/ family members of children/newborn under your care?**
   (Probe: Approach immediately after hospitalization, during the course of illness and discharge)
   We immediately after the hospitalization see the patient, usually SR (senior resident) at the duty treat the child, if it is during morning time, we see all the patient during the round. The patient who are being discharged parents are informed to take care of the child.

4. **You come across several sick patients, who are at high risk for death. How do you inform and counsel the parents/ family members of critically ill children/newborn under your care?**
   (Probe: How does the approach for critically ill children differ from the regular non-critically ill children, time devoted, frequency, person counseling/discussing with family)
   Counselling for severely ill patients starts from the beginning, diagnosis in terms of present condition is told to family. <who in family> usually to the father or mother who so ever is present, if the child is accompanied by aunt or any other person then they are asked to call father of the child. Complications are explained, diagnosis and complications at the time of stay at hospital is explained. The time given to patients depends upon the condition of the child. During rounds we tell the condition after that, SRs are always present for their queries.

5. **What has been the hardest part about being a doctor treating sick children/newborn?**
   (Probe: informing/counseling the parents and family members, handling the deaths, etc)
   Informing the parents in case of death of the child is more difficult as they don’t usually accept the situation. There is empathy in mind that the family is losing the child. Handling death is also difficult, especially during declaration as many times family don’t accept the situation.

Death and related procedures

6. **Several of children/newborn under your care die. Please describe the death declaration process usually followed at the hospital.**
   (Probe: How is the death declaration done, place of declaration, time taken, whom primarily targeted, usual reaction from families)
   During that time, we usually call the parents usually the father or grandparents or any other close family member to the bedside, then we tell them about child condition and explains that we tried our best to save the child but he/she could not survive, then declare that child is no more. After that the document process is followed, death form is filled which is done by SR or

JR present on the duty, then gate pass is issued to the family to take the child with them. <usual reaction from families> it depends upon the family, many a times they accept and many a times they deny.

7. **Please let us know about your personal experience of last death declaration in patient under your care?**
(Probe: Mode of declaration, time taken, family members present, reaction from family members)
Last week one, neonate died during my duty. The parents of the neonate were not present at the time of baby death only grandmother was present, she did not react much as the condition of the baby was critical and she accepted the situation, they brought the neonate in very critical position, to my knowledge baby was referred from AIIMS. But usually I don't declare death, it is mostly done by SR present on the duty.

8. **What are the challenges encountered by you regarding death declaration in patients under your care?**
(Probe: explaining and convincing parents/family members, handling reactions, time needed, workload and other competing priorities, support from other colleagues/senior and staffs, security)
The challenge usually comes when the child is brought and if the death happens in some hours only then, there is difficulty in explaining the parents about the child death, as they don't accept the death and many a times, they say the child was fine at home and here he is no more. Workload is usually managed as a team work.

9. **How your juniors (including trainees), co-workers, and other hospital staffs usually support around death of a child under your care?**
(Probe: Who supports, what type of support given, any expectations)
Usually the SRs, JRs present with the consultant. All know their respective duties.

10. **How are the children brought dead to the emergency department handled in this hospital?**
(Probe: assessment, documentation, medico-legal aspects, autopsy)
In the cases where child is brought dead, we check history of fault case or past records or documents are seen, if MLC is required then it is written. The post mortem is offered to the family if the cause is not known but they don't give consent in the fear of further disfigurement of the child.

11. **How do you prepare yourself and parents/family members of the child who is dying/dead?**
(Probe: mental preparation, seeking support from seniors/colleagues, informing parents/family in advance to prepare them)
It is not the process in which you have to prepare. It starts with empathy towards the parents. Then when the child is sick, we start with explaining diagnosis, nature of disease to the parents. After the child dies, we explain to family members or who so ever is present that what can be done in future to prevent such deaths.

12. **How does the death(s) of children under your care affect you?**
(Probe: mental stress, frustration, work performance)
Sometimes, for whole day it affects but it does not the work performance as we have other patients to see. As during resident ship, it (death of child) affected more, but with you mature and adapt to situation, where you have feeling that you tried best to save the life of the child.

13. **How do you handle the reaction/response of parents/family members related to death?**
(Probe: explanation, seeking support from senior/colleagues, security)
After declaration we give time to family or support if they need some help, sometimes family waits for their other family member to come to the hospital, till then they are in the ward. Parents are usually sad at that moment.

14. **What is your view about the causes of death, declared and exact/underlying cause/etiology for the children dying in the hospital or brought dead to the hospital?**
(Probe: approach to declaration, past records, mention of cause of death, effort for autopsy)
In the cases where child is brought dead, past records or documents are seen, if MLC is required then it is written. But parents do not accept that their child is no more they have difficulty in accepting the truth.

15. **In your view, how can such deaths be prevented?**
(Probe: information about causes of death, efforts for autopsy, advanced investigations, care seeking and referral practices)
Such deaths can only be prevented if there is awareness among the community and families and when they know that when they have to go to the doctor for getting consultation for the illness and they need to follow up with the doctor and not when the child is in critical condition. The child should be referred well in advance not when he/she comes to critical condition when private doctor send the child. So, the awareness and referral practice need to be focused.

16. **Have you received any training in communicating and handling death and critical illnesses? If not what all you would like to have in such training?**
(Probe: any training on communication- formal or informal, reading or self-learning)
No, I have not received any such training during my studies, but I have learned with my experience. I think no orientation program or workshop of 3-4 days can teach or impart these skills, it can only be done or good communication skills can be achieved by including these as a part of under graduation, then only it helps to handle the situation in better manner.

17. **What has been your experience with autopsy, post-mortem biopsy after death?**
(Probe: Are these being done, benefit, action based on the findings)
We do tell the family to undergo autopsy especially when the cause is not known or when the child is brought dead but parents does not give consent for the procedure, as they believe the child should not undergo more procedure and they blame us for not saving the child and also, they have fear of further disfigurement to the child body. No, autopsy has been done in my service.

You might have heard about Minimally Invasive Tissue Sampling (MITS). MITS involves collection of tissue samples similar to biopsy and body fluids from a deceased patient for

investigations. The samples and fluids collection are subjected for various tests to identify the cause(s) of death.

18. **What is your view about MITS being used as a mode of identifying cause(s) of death in children/ newborn in your hospital?**
    (Probe: feasibility, acceptability, perceived benefit, workload)
    The acceptability I don't know will they accept or not as in cases when they refuse for autopsy so it is difficult whether they will accept to go for this technique. The feasibility to perform this procedure will be there in this hospital that's why they have initiated the project. The workload will also increase if the present doctors have to perform these tests. It would be better if other team is allotted to perform MITS procedure.

19. **In your view, how the parents/ family members can be approached and convinced for MITS?**
    (Probe: which health staff should approach, which family member should be approached, timing for informing, mode of communication, place of informing)
    Father or grandparents should be explained about this procedure, that time mother is not in the situation to understand the things. We consultant or senior doctor present at the time of death could inform the parents about this technique, or in off hours even SRs can explain, but this can only be done after when the death happens because before that nobody will be ready to listen the procedure. <mode of communication> picture about the technique could help them understand better, wait but then by this they can be afraid that what hospital will do to the child even after the death, so that could counteract to technique, so it is better that parents should be informed verbally, there could be separate place where they could be explained about MITS.

20. **In your view, what could be the expectations of parents/family members if they agree for MITS?**
    (Probe: detailed report, convey of the findings, time, cost/money)
    If it's free of cost and time taken is less because they have already lost their child and they might think why it is done now when the child has already died so this needs to be explained them clearly that it can prevent further deaths or it can help them in future pregnancies. Also, the reports of the test must be handed over to the family then only it would be helpful.

21. **What are the potential challenges/barriers for autopsy & MITS in the hospital setting?**
    (Probe: procedural, space, manpower, workload, time needed, cost)
    The challenge is mainly in getting consent from the family and convincing them to undergo this technique. And as only said, the workload will increase if the present doctors have to perform these tests. It would be better if other team or project person is allotted to perform MITS procedure. Space is not the issue at hospital like Safdarjung hospital.

22. Any other comment/ suggestions.

Thank the respondent for his/her critical contribution.

In-depth Interview- Doctor (Pediatrics) 2

1. **Basic demography**
   1.1. **Designation**- Pediatrician

Service delivery and communication

2. **Please describe your role related to the clinical care for patients and types of patients seen by you.**
   We see all kind of patients, every aspect from mild illness to the very severe illness. In care starting from the admission baby, examination, diagnosis, till the baby or child is discharged, this our whole responsibility as treating doctor.

3. **You come across several patients on daily basis. How do you inform and counsel the parents/ family members of children/newborn under your care?**
   (Probe: Approach immediately after hospitalization, during the course of illness and discharge)
   We generally approach the child at time of admission only. The process of informing and counselling the family or parents, it is in multiple headings, first we tell about what is the illness, whether it is very severe, then we tell about the facility what is available and what are the treatment options and what are we giving. And when it is too sick and we feel it might come to illness, sometimes there are patients who are so sick that they die within one hour of the admission so we inform them anything can happen this all is told at the time of admission only, and we take written consent as well.

4. **You come across several sick patients, who are at high risk for death. How do you inform and counsel the parents/ family members of critically ill children/newborn under your care?**
   (Probe: How does the approach for critically ill children differ from the regular non-critically ill children, time devoted, frequency, person counseling/discussing with family)
   This is done for all sick patients. <approach for critically ill children differ from the regular non-critically ill children> This is the room it is like so much of patients loads, so we prioritize how much time each patient will get, sick patient because the doctor is only 1 doctor or two doctor are there, and there will be like 40 patients, so the only difference is that sick patient will get more time and treatment will not differ, the standard treatment will be given to all but time will differ, sick patient will take half an hour, 40 minutes to 1 hour, after that also we go to watch him, to stable patient it is less time. <counsel/informing the family>the person who has admitted, the consultant and physician informs the family only on rounds, when they come for rounds they at that time they will counsel, sometimes they counsel and sometimes they don't counsel, that depends upon case to case, otherwise admitting doctor counsel the patients family.

5. **What has been the hardest part about being a doctor treating sick children/newborn?**
   (Probe: informing/counseling the parents and family members, handling the deaths, etc)
   It is like informing the family, as most of the time it is a denial, denial that child is not that sick, they feel that he was walking few hours back and now he is like so sick, like almost at the verge of the collapse, so that part is the hardest part.

Death and related procedures

6. **Several of children/newborn under your care die. Please describe the death declaration process usually followed at the hospital.**

   (Probe: How is the death declaration done, place of declaration, time taken, whom primarily targeted, usual reaction from families)

   Deaths are never declared like all of the sudden, most of the child which are at the verge of death so you know the clinical course that how the child is behaving so the patient is counselled right from the time of admission you feel that it is critical, like somebody is just normal and mild illness and not going to die, so at the time of admission only you that the child is very sick and this child can die, so at that time it start counselling that the child is very sick and child can stump to the illness after that you just keep counselling them, every day, if the child is staying for 4-5 days then we counsel every day still sick, we tell about treatment option that we are giving everything, all the things are given despite that the child is not improving, after that even before death we counsel them few times, that the time of death we call them and we tell them that we did everything but child could not make it. <whom primarily targeted>usually the father and the mother, sometimes father feel that they don’t want mother to get involved in that so we will tell that don’t tell mother, otherwise it is told to father mother both. <usual reaction of the family>it is variable, sometimes it is like some families feels that, like if they are staying for 7-8 days then the denial part gets over and they understand that the child was sick and we did everything, sometimes there are people like less educated or sometimes they feel like that the treatment was not adequate, sometimes they are violence and sometimes there is like verbal abuse. It depends on the family and it depends upon from individual to individual.

7. **Please let us know about your personal experience of last death declaration in patient under your care?**

   (Probe: Mode of declaration, time taken, family members present, reaction from family members)

   It was like two days ago, so the child was brought him like almost he was, that not much we can do, the child died within half an hour or so, within half an hour of the admission. They knew that the child is almost then, but I think I felt bad but they just wanted to go home, that was it, it was not like nothing I could say, they were like we have to take and they took the dead body and we had to stop them for the papers otherwise, last was not hard but sometimes it gets like really horrible. <family member who were present>only mother and I think some aunt was there, two members were there.

8. **What are the challenges encountered by you regarding death declaration in patients under your care?**

   (Probe: explaining and convincing parents/family members, handling reactions, time needed, workload and other competing priorities, support from other colleagues/senior and staffs, security)

   Both are important and both are challenges, sometimes they don’t understand of course like nobody will understand and second is like the workload is so much that sometimes it is like only one doctor is there and there are almost more than 100 patients, like one senior pediatrician is there and one might be 1st year junior resident will be there along with it, so almost sometimes it is around 150 patients in the ward, 100,120,130 and sometimes it can reach 150 sometimes and there are only two doctors there then there are 10-15 sick patients,

sometimes 20 patients can be there, on life support there are 20 patients, we cant give time to all there are just 24 hours, even if you divide 150 patients by 24 hours, one patient will get almost 20 minutes in a whole day that is if you see him only once, that administration have to be, it is just not humanely possible. <support from other colleagues/senior and staffs>security is like there are guards are there and sister are there they are doing there work they are helping parents and patient getting treated, otherwise from seniors on duty it is not much help, they will even try to suppress you so that is, so they can non-supportive, they will not understand and they will actually try to find your fault in case of care or death.

9. **How your juniors (including trainees), co-workers, and other hospital staffs usually support around death of a child under your care?**
   (Probe: Who supports, what type of support given, any expectations)

10. **How are the children brought dead to the emergency department handled in this hospital?**
    (Probe: assessment, documentation, medico-legal aspects, autopsy)
    It depends if the patient is referred as complete treatment history and illness is there documented history is there and brought dead like two days ago one patient was brought dead from AIIMS so he had proper documentation and everything was there, so in those cases we documented our, we have separate book for brought dead patients and then we give and write it in the form, before that we just take an ECG to confirm, if we feel that it is just, it just that the patient just came, he just went to cardiac arrest, we try to revive if it is not reviving we just declare that it is brought dead and then, in this case it came from AIIMS and we did initial resuscitation and patient still not and there was no heartbeat, we documented it in our book then we send back to AIIMS for the declaration of the death, death declaration took place at AIIMS. If otherwise it is unknown patient then the procedure is medicolegal procedure full postmortem should be there, so that MLC have to be made, then there will be MLC after that death will be declared. It depends upon case to case.

11. **How do you prepare yourself and parents/family members of the child who is dying/dead?**
    (Probe: mental preparation, seeking support from seniors/colleagues, informing parents/family in advance to prepare them)
    We just try to convince that we did everything we can that child is getting, it starts way before death, tell illness we are treating and these are the treatment options and your child is serious, your child is getting all the treatment which is available, no matter where is the child from, in our hospital so the treatment will not differ, despite that he is deteriorating towards the final movement so, we try to say that we did everything, somethings are not in our hand.

12. **How does the death(s) of children under your care affect you?**
    (Probe: mental stress, frustration, work performance)
    In our hospital there is like so much work so you can't afford to decrease our performance, like you have to work otherwise if you will not work then other patients will also die. If that's affects you then next patient will die. Frustration comes because like there is nothing that you can do like there is no hospital policy like if 1 doctor will see 100 patients, patients are going to die and then you can't do anything. All you have to do is to do with whatever you can do.

13. **How do you handle the reaction/response of parents/family members related to death?**
(Probe: explanation, seeking support from senior/colleagues, security)
It is also case to case, there are like trying to threaten you or they are just trying to abuse you then you just ignore and then you will carry on your work, if they are trying to physically assault you then, of course you will call security and they try to save you from violence.

14. **What is your view about the causes of death, declared and exact/underlying cause/etiology for the children dying in the hospital or brought dead to the hospital?**
(Probe: approach to declaration, past records, mention of cause of death, effort for autopsy)
Most of the time it is delay of the transportation to the hospital sometimes the child was ill from 2 days but they are staying at home, and then they decided to take the child when almost when sometimes the illness becomes non-reversible, it goes beyond the time before the you can intervene. After that I told you that the cause can only be known after the post mortem in those cases. <are post mortem conducted for child in Safdarjung>it does, for unknown deaths the post mortem is done.

15. **In your view, how can such deaths be prevented?**
(Probe: information about causes of death, efforts for autopsy, advanced investigations, care seeking and referral practices)
Sometimes, people are so poor that they can not afford hospitals, and healthcare is almost negligible so I don't think these deaths can be prevented, they are only certain in night in Delhi so can only see tertiary care hospitals working otherwise you can go. These can be prevented only if by education if they take their child on time, sometimes the child is not that sick if he gets those supportive treatment on time then he might on deteriorate to such a bad condition that it is beyond salvageable. So, if they take child to some good facility where intervention can be done at earlier stage that is the only way otherwise if they bring in that critical condition then they cannot be treatable. <advanced investigations/referral practice> referral practice are the main problem like if they are, it is a tertiary care hospital cater to the extreme sick patients and the patients like who are not that sick or not requiring ventilatory care can be treated at secondary level hospitals, but the practice is like that the secondary care hospital do not admit the patients, so the load will ultimately increase to tertiary care hospital were we have limited resources like only one or two doctors are there, if the load will decrease then automatically death will decrease, doctors will able to give more time to the patient. Instead of like 150 patients then patients will be 40-50 patients so care will definitely increase.

16. **Have you received any training in communicating and handling death and critical illnesses? If not what all you would like to have in such training?**
(Probe: any training on communication- formal or informal, reading or self-learning)
No, we have not received any such thing. <what all you would like to have in such training >there should be counselling sessions, I think most important part is that the senior doctor should talk to the family more often like every patient should be counselled, maybe it should be the part of, clubbed with rounds or separate like that senior consultant are available from 9 to 4, so then they can after rounds call the attendants to their chamber one by one then they can separately counsel them, it is like more effective when a senior person is counselling as compared to the junior doctors that can be one part. Here SRs and JRs declare the death, all

the deaths are declared by senior resident and junior resident, there are day when SR is not available only JR is on duty there are some days because the manpower is limited, there are many days when only junior resident is on duty night duty, if death will happen at that time then junior resident will declare death.

17. **What has been your experience with autopsy, post-mortem biopsy after death?**
    (Probe: Are these being done, benefit, action based on the findings)
    I have not done that much causality so right now in my senior residency no post mortem has been done, under my work. The only benefit is that if death is due to some, there is abuse or physical or all those causes sometimes neglected child are there, some family deliberately tried to kill, in those cases it will be beneficial otherwise there is just like wastage of resources.

    You might have heard about Minimally Invasive Tissue Sampling (MITS). MITS involves collection of tissue samples similar to biopsy and body fluids from a deceased patient for investigations. The samples and fluids collection are subjected for various tests to identify the cause(s) of death.

18. **What is your view about MITS being used as a mode of identifying cause(s) of death in children/ newborn in your hospital?**
    (Probe: feasibility, acceptability, perceived benefit, workload)
    It (MITS) can be done but then administration have to provide all those facilities like I told you that like only 1 or 2 doctors are there and if they will try to do this thing done and leave clinical then more patients will die, then you will need more MITS. So, in some hospitals there are like forensic people are on causality duty 24*7 so they handle all these legal cases, like somebody is brought dead then, they will call forensic department person, then he will come then he will do his procedure, that is done in many medical colleges. Here you have to do autopsy all these things clinician have to decide. It is performed in the mortuary but all procedure of transferring, all that it is like paper work is done by clinician. So, it will take 1 hour. If it takes 1 hour then other work is going to suffer or other critical patients will suffer.

19. **In your view, how the parents/ family members can be approached and convinced for MITS?**
    (Probe: which health staff should approach, which family member should be approached, timing for informing, mode of communication, place of informing)
    Most of the time, they will not likely to give consent because like you can imagine that most of the doctor’s face violence and verbal abuse, imagine if you will say that we are going to take samples out of body then, maybe that will counter benefit then beneficial. But still they will feel that they don’t want to mutilate the babies body like you could not save when he was alive then what you are going to do when he is dead. <there is no mutilation of the body in the MITS> then they will ask that what is benefit of that, they are not getting any benefit, maybe hospital is getting any benefit, their baby has died. <they can be told it will benefit in future pregnancies> maybe, but if diagnosis is all clear cut and there is no diagnostic dilemma and, in that case, if you are going for post mortem then I don’t think it is ethical, it is beneficial if diagnosis can not be made and child died in those cases definitely it should be done. If they give written consent then it should be done.

20. **In your view, what could be the expectations of parents/family members if they agree for MITS?**
(Probe: detailed report, convey of the findings, time, cost/money)
Most of the time they want to go home, that is only expectation of the family after the death, either they are not happy that treatment is not done properly in that case they will not agree, in other cases their child may be critically ill last 15 to 20 days in those cases they just want to go home.

21. **What are the potential challenges/barriers for autopsy & MITS in the hospital setting?**
(Probe: procedural, space, manpower, workload, time needed, cost)
Maybe it can be done if they on call faculty will come and talk to them, senior doctor will be there on floor, so in those cases maybe we can get few people to agree for procedure. But that is also an issue it can be during 9 to 4 or 9 to 9 when some faculty members are there, otherwise during night time it is almost like impossible because you don't have any senior consultant support. <so, they will agree when senior doctor will approach>it is not that but it is like almost more effective, like when you are treating the person who is on floor declares death and they feel that some more senior doctor is taking rounds and then they feel like overseeing all the treatment, the impact is more if they talk to them.

22. **Any other comment/ suggestions**.
No, thank you.

Thank the respondent for his/her critical contribution.

In-depth Interview- Doctor (Pediatrics) 3

1. **Basic demography**
   1.1. **Designation**- Pediatrician

Service delivery and communication

2. **Please describe your role related to the clinical care for patients and types of patients seen by you.**

Role like, when we do duties in emergency, we are the first hand doctors that the patient encounter like diagnosis and all types of patients, ICU care, in every, we handle every type of patient, single handedly from second year onwards. Usually like Respiratory, Bronchiolitis, Pneumonia, and meningitis. In neonates; preterm, low birth weight, patient with birth asphyxia, meningitis, and requiring ICU care or what all should I tell.

3. **You come across several patients on daily basis. How do you inform and counsel the parents/ family members of children/newborn under your care?**

Actually depends upon the severity of the situation, or severity of the illness, if the child is in terminal condition, in sick condition, first we take like if child needs ICU care, and we don't have the beds, so first we inform them that there are no beds available in the ICU but the child needs ICU care. So by taking consent we only admit the patient that you are ready to do, the patient will be admitted in the general ward, not in the ICU. Then you are ready, admit the patient in the general ward itself, if the bed will be available in the ICU then only we will shift the patient. Like there is no beds available, then you have to take the patient in normal, general ward only. The patient is terminally ill because some patients come in terminally sick condition like, died within one to two hours, like the died in new emergency block only. Then we counsel the patient, that the patient is in dying condition and then it depends on the condition of the child, if the patient is not sick, like normal, in vitally stable, bronchiolitis, pneumonia patients then counsel according to that the child might be discharged within 6-7 days and might be discharged within 1-2 days. Like, if patient comes with AG, acute gastroenteritis, diarrhea, dehydration, and if the situation of the child is dice then we say to the parents it will be the course of the illness that will decide the child will be ok or not. Whether the condition of the child will be deteriorated or child becomes well. It depends upon the course of the illness. If we don't know, what the course of the illness will be, like if the child is in between the sick condition or in well condition. It depends, like if the load of patients are more, we usually try to counsel if the condition is deteriorating like we try to counsel those patients more who are deteriorating and are like, can expire within one to two days and can go, can become sick within one to two days or immediately because if the condition of the child is improving then it is ok, we don't tell them and we tell them like child is ok but if we don't tell them that the child is sick then there will be problem. So we try to, if the load is more and patients are more then we try to, counsel those patients who are sick and near expiry or some.

4. **You come across several sick patients, who are at high risk for death. How do you inform and counsel the parents/ family members of critically ill children/newborn under your care?**
   (Probe: How does the approach for critically ill children differ from the regular non-critically ill children, time devoted, frequency, person counseling/discussing with family)

The condition of the child is very sick, if the child is sick from birth only, we try to tell them what is the situation around here when you took the child here, what was the situation there. What change has been there, or child has been sick from beginning also, when the child was taken here only from another hospital, sent to the, it depend on that situation we tell them about the illness of the child, that we suspect this and this is going on, and we are giving this much, this much, this much we can do and that we can't do. And we tell them about that, the treatment, prognosis, illness and all that we counsel them about that. Most of the times of ours is consumed in those patients only who are sick, because they are sick and we have to monitor those patients regularly and all. Most of the time of ours is devoted to them only, but counseling like around 10-15 minutes and everyday, like who is coming to the duty, he counsels before the start of the duty and all, and when the consultants come, senior, rounds are taken, so counseling is done by consultants only, like how is the situation and how the progress will be. To the immediate parents (counseling), or the guardians, if parents are not available, we don't counsel family members. We always tell them father, or the mother, or the grandparents if the father, or the mother are not available, we don't tell aunty or someone, because every time we have to explain from the beginning. Then we only try to counsel the patient, I mean, patients's relatives, like immediate mother, father and.

5. **What has been the hardest part about being a doctor treating sick children/newborn?**
   (Probe: informing/counseling the parents and family members, handling the deaths, etc)

I think, because most of the patient that come around here are terminal patients, like it is like a dumping zone for the sickest patient, they are refused from everywhere, we feel like helpless. You know going from here, I only expect that sick children are there, just see most of the time sick only patients, like normal patient, near normal patients is very less, we encountered. Its like, there are manpower problem and all, we could do more, but we are not able to do. We are, duty hours and all, that is the hardest part, there is always a continuous reminder to us, we can do more, we can do more. But there are physical limitations and mental limitations are always there. We cant expect so much from a doctor, after 24 hours duty and when he is coming fresh, that is a problem, we can't give our 100 percent if we are continuously working and we are not given time to relax us. We are treated not like other human beings, like we can't do, we can't do so much things so much work around here with 100 percent capacity that is the main thing. No, actually after doing two years job around here that is not a difficult for us to counsel or something but to make parents or relatives realize the situation of the child is this, that is most difficult, some parents are not accepting the situation only, they are illiterate, we can't tell them why it has happened, their level of mental makeup is not up to that point we can counsel why this has happened or not. That is the main issue but counseling after experiencing so many deaths and counseling, we have just thorough with that how to counsel and all.

Death and related procedures

6. **Several of children/newborn under your care die. Please describe the death declaration process usually followed at the hospital.**
   (Probe: How is the death declaration done, place of declaration, time taken, whom primarily targeted, usual reaction from families)

Yes, two days before (asked when is the last time death is declared). Actually, if the child is in arrest like when we started the CPR, CPR is given for 30 mins atleast, we tell them, the child might go, might expire, we have given all the medications and all, and it takes around one to half an hour to one and half an hour, it depends upon the condition of the child, if the child has come

brought dead patients are also there, then we have to declare within half an hour because the child is not there, we have to tell them that the child is not there whenever you come the child is not there, and after 30 minutes, after giving effective CPR if the child is not there, we have to declare the death after 30 mins only. It depends on the condition, around half an hour to one and half an hour, is the time, declaration of the death. Paper work is like, there is a child death review form, which is filled less than 5 years, and there is a green form like, that is death certificate, there is CPR note, there is death summary, and there is immediately death slip given to the parents so that they didn't get any difficulty while taking the body. So, that's 4-5 paper. Usually post graduate or senior resident, declares the death only.

7. **Please let us know about your personal experience of last death declaration in patient under your care?**

(asked how do you declare) That the child is not there and. (Asked time taken) If the child comes in arrest only, then only half an hour, if the child is brought dead and all, then only half an hour you have to declare. (asked who they declare to) only parent only, if parents are not available, immediate guardians. (asked reaction) most of them don't accept if the condition is very acute and if we counsel them on regular basis and all, if the child is sick from beginning then they are said the death, there are different types of people and different reactions to acceptance to death only.

8. **What are the challenges encountered by you regarding death declaration in patients under your care?**

If the child is around 10-12 years and suddenly some illness has occurred in and family and so long the child has been bought up by the parents for 10-12 years, then that is difficult for them to accept it. The most difficult part is to realize them to accept the death, like the child will be no more and some are like they are living with the child in illness only. Some child are like global developmental delay, they go to ICU, they come back, they go to ICU, come back. They always say like last time, he was out, now why not. They are like attached to the sickness of the patient. To realize them, this is the end.

9. **How your juniors (including trainees), co-workers, and other hospital staffs usually support around death of a child under your care?**

   (Probe: Who supports, what type of support given, any expectations)

Most of the time we get support from seniors also, and sometimes other supporting staff is not there, so much of supporting staff is not there with us, only sometime one sister, one doctor is all we have, nobody else, so, that is, supporting staff is very less around. Seniors only (help in declaration of death), like from beginning they told us how to declare the child, how to counsel them regarding this situations and all, only seniors guides us only. (asked if there is any expectations from co-workers) Yeah, a lot, from atleast co-workers atleast. But we get hardly any support from. Like, management of the child only, there are, manier times suction machine is not working, and oxygen point is not working, the staff has gone to some other work, only one staff for help in care, there is only one staff.

10. **How are the children brought dead to the emergency department handled in this hospital?**

   (Probe: assessment, documentation, medico-legal aspects, autopsy)

If the child has been taken from another hospital, we inform them that the child has been brought dead, and that child is taken to them only. And if previous papers of illness are there, and we know that this is the cause of the death, then it is ok. If don't know the cause of the death, some sudden death, then we it has to go under MLC, medico-legal and post mortem, and. (asked

documents) We only write in the, like last time, one patient was taken from AIIMS and brought dead, whoever has taken the child, we inform them also, like phone, or write in their notebook, like the child has been brought dead and all. (asked if further investigations done to confirm death) Clinically and ECG only.

11. **How do you prepare yourself and parents/family members of the child who is dying/dead?**
    (Probe: mental preparation, seeking support from seniors/colleagues, informing parents/family in advance to prepare them)

(Asked how do you prepare yourself) Actually we go to deaths daily, around daily basis, one or two, used to it. (asked how parents are prepared) Just by repeatedly telling them that how the situation is.

12. **How does the death(s) of children under your care affect you?**
    (Probe: mental stress, frustration, work performance)

Initially it used to effect a lot. Sometimes if some expiry is like we can't explain to our self like how does it happen, or something, we don't know, like we are not so experienced by that, then initially it used to effect a lot, like we can't able to sleep and all. Like that is, it has effected our character also, from beginning where we have not been in this clinical practice to today, there is a lot been changed in our personality also. Like seeing the scenario of life, death, it has changed a lot in us also. Like how to react to a situation. Initially sometimes we react differently in different situation and in our personal life also, then we go back to our homes and where we do not see these things, like life, death, we realize that we have come way far away from that situation. (asked about frustration) A lot, a lot, everyday. (asked effect work performance) some, some, it effects our personal life more I think.

13. **How do you handle the reaction/response of parents/family members related to death?**
    (Probe: explanation, seeking support from senior/colleagues, security)

Sometimes, somebody reacts a lot, then there is danger to our life also, then we call security and bouncers are also there to help us. Sometimes we have to be like, we can't take physical reactions from there, then we call the security, like, to help us declaring the death. We call the bouncers and security before we are declaring the death. So that takes any emergency, or something, some physical situation comes around there then. Some specific cases (security is called). (asked when they call security prehand) Like I am saying, we are counseling like before, two and half hours, then what is their reaction only. How are they, angry or, then we know that the situation will come around, if they are accepting the death from beginning only, we know the the situation will not come.

14. **What is your view about the causes of death, declared and exact/underlying cause/etiology for the children dying in the hospital or brought dead to the hospital?**

(asked if knowing the cause of death is important and how) Yeah, so then we can pre-predict the cause of death beforehand in other patients, to treat down like, if we know the cause of death around here, maximum cause of death, we can use that information in other patients and in upcoming patients only. (asked help in declaration to parents) Yeah definitely, it helps.

15. **In your view, how can such deaths be prevented?**
    (Probe: information about causes of death, efforts for autopsy, advanced investigations, care seeking and referral practices)

I think, there should be more nursing care and extra supporting staff should be there. Atleast junior doctors like JR's and interns, there is no junior resident, like post graduate stands in non-teaching, if the manual work is reduced then we can do more for the patient like, if are doing

suctioning, cannuling and everything we are doing by ourself, then it effects our thinking process and all, it effects all. If the supporting staff is more, like if nursing care is different, everything is divided and known to those you have to do this, we have to do this, then the survival and survival care will also be affected, it can be improved also. (asked if efforts for autopsy can prevent child death) Actually we have no experience alone, no experience of autopsy and. (asked autopsy can reduce the number of deaths) I don't think so, because that will help but there is no staff to do that. There is no extra staff to take care of that, we are doing our best, we are doing our 100 percent. That can't be changed like if more extra staff and helping staff is not recruited and that.

16. **Have you received any training in communicating and handling death and critical illnesses? If not what all you would like to have in such training?**
    (Probe: any training on communication- formal or informal, reading or self-learning)

Yes, I have received. I have received one workshop and it was done last year only, in safdarjung hospital only. We have four-four, five-five PG's were selected and given from every department four to five PG's were selected and given the training. Not all of them, that was like first primary demo. I have done that and received the. It was like an interactive section.

17. **What has been your experience with autopsy, post-mortem biopsy after death?**
    (Probe: Are these being done, benefit, action based on the findings)

No experience (post mortem, or biopsy). It will benefit definitely. Like if the cause of death is unknown, we are also in the dilemma like what is the cause of the death. In that it will help us.

You might have heard about Minimally Invasive Tissue Sampling (MITS). MITS involves collection of tissue samples similar to biopsy and body fluids from a deceased patient for investigations. The samples and fluids collection are subjected for various tests to identify the cause(s) of death.

18. **What is your view about MITS being used as a mode of identifying cause(s) of death in children/ newborn in your hospital?**
    (Probe: feasibility, acceptability, perceived benefit, workload)

It will help (MITS) but I don't think so people will give permission to that. Because the patient comes, the patient around here is illiterate people, I don't think so they will give permission to that only also. They don't give permission to autopsy, definitely they don't give. But I don't think so whether they will provide consent for that. It will depend upon, I will tell you, who accepted the death, and who don't. It will depend upon the nature of those seriousness who wants, I think people wants to know what is the cause of the death but whether they will allow it or not, I am not sure. (asked preference for post mortem or MITS) I don't know about the results and outcomes of that only. It will be good if MITS is done, because no cuts and yeah (there will be no disfigurement).

19. **In your view, how the parents/ family members can be approached and convinced for MITS?**
    (Probe: which health staff should approach, which family member should be approached, timing for informing, mode of communication, place of informing)

They can be told like, it will help in diagnosing the patient what is the cause of the death, and there will be no scar marks or disfigurement, as you said, like there will be only small spots on the body. It will not affect their emotions and all. that is the way we can talk to them. That will help the patient. Or might be future perspective if the disease is in the family, runs in the family, other ones, siblings and all can be benefitted from that. (asked time) I think two-to three hours before the death only. So that we can repeatedly counsel them, that it will be benefitted like. It

should be to the immediate parents only because they are the only ones who will give the consent only. And if we counsel the other ones, they might anti-counsel for that. We can tell them, its like autopsy only but there will be no change in the. Obviously if videos and photographs or pictorial presentation is always good. But verbal counseling is well enough like. (asked if senior consultant should approach parents) then it will be, definitely, they will consider it more, if some senior person will come and talk to them.

20. **In your view, what could be the expectations of parents/family members if they agree for MITS?**

They always wanted to know what the cause of the death is.

21. **What are the potential challenges/barriers for autopsy & MITS in the hospital setting?** (Probe: procedural, space, manpower, workload, time needed, cost)

that, accepting the death, and letting. There should be extra staff for that only. It should not be expected from the same doctor who is doing monitoring and who is doing the same work. There should be extra staff supported for that 24 hours. Not like 9-4 and 4-12. There should be always should be extra staff for doing this. because this is some extra work and there might be paper work also. To fix that doctor, doing all this is not good, not feasible and it is not possible at all. No, because if the staff is there, money will not change that, if the same doctor has to do and,. manpower there is a point.

22. **Any other comment/ suggestions**.

I just want to tell as per the load and the deaths going around are very high and the load is very high. doctors should not be extra burdened with this only who are working with clinically and managing the patient. Because it will deteriorate the condition more. Like if we are doing that procedure of telling them counseling them, doing them, then that will cost around half an hour, one hour extra, doing paper work, one, one and half hour. It will affect the other patients who are surviving and fighting for their life. It will definitely affect those, will not be able, we are filling like 4-5 forms around here now. And adding 2-3 forms extra and doing paper work, it will increase the load and we will deteriorate other patient’s clinical condition because sometimes one people in duty only, and he has to do everything, putting the cannula, to monitoring the child, to giving treatment. And this is not at all feasible for that one person. Humanly not possible.

In-depth Interview- Doctor (Newborn Care Unit) 1

1. **Basic demography**
   1.1. **Designation**- Neonatologist

Service delivery and communication

2. **Please describe your role related to the clinical care for patients and types of patients seen by you.**

I m basically seeing newborns , neonates who are in born in the hospital, and , so role is related to post graduate teaching, training and clinical care.

3. **You come across several patients on daily basis. How do you inform and counsel the parents/ family members of children/newborn under your care?**

Those who are sick are directly counseled by us. Baby have some critical condition, like heart disease, baby is not likely to make it, those situations parents will be counseled. We sit down and then we tell about what all problems are expected , if a preterm is born, then what all problems are , the baby is likely to undergo, heart, eyes, brain , infection, how long the course is going to be, what the mother is supposed to do at that time, all those things.

If the baby is having long stay, then also baby is not going to go home, baby is going to stay at least for a month. So those parents are the ones, other counseling is done by the residents. (discharge time) how to take care of the baby at home, temperature, maintenance, what are the danger signs, how to feed the baby, hygiene, hand hygiene specially the first time of feeding, how to position the baby, follow up, all those.

4. **You come across several sick patients, who are at high risk for death. How do you inform and counsel the parents/ family members of critically ill children/newborn under your care?**

Yes, so one is the diagnosis part, which is like, suppose if you are suspecting something, you are not able to get it done, some special tests are needed sometimes, the baby is not likely to make it. So the diagnosis is not clear why the baby underwent that, then the counseling is being done. Some of the tests if they are not available, we would counsel to get it done from outside, post mortem sampling can be taken, because now baby is in shock, So, we may do autopsy later. Ya babies who are in nursery, are relatively sicker baby, and some of them are in the ICU room who are critically ill. So we devote most of the time to those only. The ones who are stable babies and can be shifted to the mothers, taken care by the residents, because there are 100 deliveries happening per day, so it is not possible for one person , to see all the babies. So you would see those who are admitted to the nursery, those who are critically ill or those who are at risk and look very low birth weight, preterms, rest of the normal newborns population like who are healthy fetus with their mothers, they are counseled by the residents.

Usually mothers are available in the neonatal ICU. We counsel the mother but in case the, the baby is not going to make it, we don’t disclose it to the mother directly, we would like the father or somebody else to be present.

5. **What has been the hardest part about being a doctor treating sick children/newborn?**

(taking time to answer this qs, being thoughtful at this time, gives a smile) See, aaa……in some cases you can anticipate what the outcome is going to be, given the baby’s condition. So, you counsel that the baby is getting the best of care and whatever could be done for the baby is being done, and that gives you solace, whatever you could do. And see that the baby couldn’t make it,

so, I mean, yes do realize and have empathy that the family is losing the baby, but then what can you do about it. Declaring death is hardest part.

Death and related procedures

6. **Several of children/newborn under your care die. Please describe the death declaration process usually followed at the hospital.**

So, usually we call the father, or a male member, not disclose it directly to the mother. And you call them and tell them that the baby's condition, and what all the baby has been through, and why the baby could not make it. And then you declare that the baby is no more.

Papers work….sisters they clean the body and wrap the body, and handover to the parents, there is a death form which is filled. And the signatures are taken that the body has being handed over. We call the parent bedside, and show the baby and then inform them, that baby has expired at so and so time. Mostly (explain the cause of death). Mostly the father (whom do you declare), or the male member mostly, we don't directly declare to the mother. See most of the times, these babies, the parents have been counseled, that the baby is sick and the baby might not make it. So, 2-3 times counseling is done and then they are prepared, that the baby might not make it. So, that when finally you declare, it does not come as a surprise that the baby is no more.

See if it happens suddenly, they bring the baby from the post natal ward sudden demise which was unexpected neonatal death, then there would be anger, there could be pressure, lot of things, but in house, usually we don't find those kind of situations.

7. **Please let us know about your personal experience of last death declaration in patient under your care?**

I directly do not declare, most of the time the SR who is present declares over there, and the last time we did was in the morning itself in front of me, morning around 9:30, today. So the father was called, he was there and the mother was there, and the SR, I was there, sister was there. Well, they sad, and quiet and…

8. **What are the challenges encountered by you regarding death declaration in patients under your care?**

One challenge is in case of unexpected deaths, when the baby comes from the post-natal wards, they, when you do not know why the baby died, suddenly when the baby was feeding, while everything was fine, why the baby expired. They don't agree for any post-mortem usually, which we offer them. So the cause remains unverified. Well they should be empathy, they should get the family area, sit down and care may be, because some of them might not be able to handle, they should have a private area, where they can you know express whatever emotions they have and they should not be in a hurry. Need to see the baby at least for that moment of time. That is what the expectation is. Usually they are overloaded all the times. Busy nursery, around 100 deliveries per day, babies coming in every now and then.

9. **How your juniors (including trainees), co-workers, and other hospital staffs usually support around death of a child under your care?**

So they would concentrate, they would not declare directly to the mothers, they would allow and wait for the father to come, the body would be there, they allow the father or the parent to hold the baby, so that they could say goodbye.

The senior consultant speaks to parents and counsel and also resident counsel and staff briefing that ok you did your best and this was the condition and the baby has to go, because they also have put in a lot of effort many times, So they also feel bad when a baby dies, they also undergo lot of stress, they are also concerned.

10. How are the children brought dead to the emergency department handled in this hospital?

Baby is taken inside in the nursery, and the parent is told that we are trying to resuscitate, so they try their best, resuscitation efforts, whatever and if still the baby doesnot show any signs of life and if there is no heartbeat, or then they declare the baby. And they offer a post mortem if the cause is not known, suppose if its known condition and they bring the baby, baby comes and expires, its ok, they give a death certificate, that the baby has expired. Otherwise if unexpected death, they offer a post-mortem, and of course documentation, certificate….

11. How do you prepare yourself and parents/family members of the child who is dying/dead?

the preparation starts by counseling them about the baby's condition, ………so you know certain things from your medical education that these condition have a high mortality, they are not likely to make it, you know these things, so from that background, and even if the baby, suppose if it's a severe dyslexia baby, even if the baby makes it, its not a valid life and it's the baby is going to be handicapped or a burden to the family, and you know what kind of a life the family would have caring for such a baby. So then you prepare yourself that way because you have seen so many babies gone home also and then later on the family say you that if this baby wound not have survived, it would have been good. S rather than having a handicapped, bedridden, vegetative baby. If the baby is dying now, its ok.

(asked about some mental preparation before declaring death or have become used to the situation now)

No, we are not used to it, it is always a difficult situation declaring death to the parents, and you do your best to comfort them, and ask them to hold the baby, say a goodbye for whatever amount of time, the baby is left now, and you provide them with some privacy to have their emotions and finally say goodbye to the baby.

12. How does the death(s) of children under your care affect you?

(thinking) It affect the sub-consciously may be, because you can't keep on grieving over one death, because you have so much of other work to do, but yes those, that experience, through that difficult situation makes you stronger, vigilant and you can give some, some kind of comfort to the next family or situation, like your residents are might be new, the first day you are declaring death, is very very difficult, you are, may be you go home and cry that oh three babies expired and I couldn't save them. But as you grow and as you mature as you see more of such situation, you are better prepare to handle, those situation and maintain your calm, and composure, but they do effect you at sub conscious level. (asked does it effect your work performance) no, it doesn't. (asked about frustration) no, if despite doing your best of efforts if the baby doesn't make it, then you don't feel frustrated, then you feel, ok you have done your best, given your best, and still if, the baby doesn't respond or if the body doesn't respond, and if baby's process is going to take its toll, it's going to take its toll. And that is what you could do.

13. How do you handle the reaction/response of parents/family members related to death?

You learn how to handle and talk over time with experience.

14. What is your view about the causes of death, declared and exact/underlying cause/etiology for the children dying in the hospital or brought dead to the hospital?

It is important because certain situations are there, so if the residents get to know that these are the scenario, and this is how the baby is present, then they could pick up earlier, with some clues, and it is important to know why the baby could not be saved, what was the actual cause of death. What was the underlying defect, or why your best of efforts have not saved the baby. Like

suppose there was a baby a few days ago, if you know that there is a hypo-plastic heart, so no matter, how fast you put your central line, how much of medications you gave, what ventilations you put, and if it is malformed already, its not going to make it, its not going to make it. So then its not in your hand, the baby has been made like that.

15. In your view, how can such deaths be prevented?

By a better antenatal diagnosis, most of the times they are not in proper follow up, so conditions which should be managed properly like, high blood pressure, maternal nutrition in patients, they come late in labor, obstructed labor, already having meconium, so the baby is going to be born asphyxiated. So then no matter how hard you resuscitate, the damage has already happened. So then, so that point, early care, better care, better antenatal diagnosis or existing malformations, so the mother also does not go through so much of pain, carrying on the pregnancy for nine months and then coming to know oh,

16. Have you received any training in communicating and handling death and critical illnesses? If not what all you would like to have in such training?

There is routine counseling training which we undergo when we are post graduates and senior residents, how to communicate, how to reveal a difficult situation, document, sit down with the family and have a quiet area where they can ask question where they can communicate, give written information in their language so that they can go home read about. Extra thing is, we are trying for bedside echo, bedside ultrasound, so that we don't have to shift babies, most of the time, they are sick, shifting to get a better diagnosis, that becomes a major hassle. So having things bedside would improve. And then post mortem is one thing which has not done almost, I haven't seen its been done till now, we could do that, then they would know the actual cause of death.

17. What has been your experience with autopsy, post-mortem biopsy after death?

If it is a suspected genetic disease, we do take samples, we can take liver biopsy by a shotgun, or skin biopsy, urine frozen sample we can keep, so those things we do, where its important where we are suspected a genetic disease and it can have implications in the next baby. Plus the family should be affording, unless its available in………..so you will know your exact diagnosis, may be you could offer some genetic counseling for the next baby to come. And for the family as a whole. Post mortem is available (asked if hospital provide post mortem). (counsel parents) Only in cases where there is unexpected death, where the baby has brought from post natal care, we do not know, or the baby is inside the nursery, you do not know what really has happened. Otherwise most of the time, the busiest ……………, there it is evident enough.

18. What is your view about MITS being used as a mode of identifying cause(s) of death in children/ newborn in your hospital?

Yeah, so its going to be good, because at present we are not having, no post mortem is being done. So not that, there will be more compliance, if we tell them its going to be minimally invasive. So the acceptance is going to be better, so of course you will get diagnosis. It should be feasible ….should accept if counseled properly, and if its free of cost, if cost is not the issue, because they have already lost the baby and they might think what's the point in getting it done, when we already lost the baby. So, proper counseling. (asked if a separate team should perform MITS) That will be better. (asked if same hospital staff are trained to perform MITS, will it be overload for them) No, it won't be, it should not be, once it is defined this sample has to be taken, its ok they can take, but yes, its already overloaded. So, it will be better if someone, some project person …..

19. In your view, how the parents/ family members can be approached and convinced for MITS?

Counsel pre-hand death, some of the points which we want, which will be clarified after the, so we have to counsel them properly. May be the senior most member, who is on the floor, at that time, who is present, the SR who is posted there. The male, the father, the male member from the family, because the female do not take independent decision, plus they are themselves going through you know, lot of problems, so its better if the father is told. When you have done your first and second counseling, that we are doing our best and that the baby might not make it, or the baby is not improving, at that time, you mention and brief about and then again you can mention before the actual ……….

20. In your view, what could be the expectations of parents/family members if they agree for MITS?

They would expect, if they could get some help in the diagnosis, which could you know can tell them about the next pregnancy prenatal counseling, or genetic workup, whatever is available, and having another baby with such a defect, so, those kind of things, that they would want. Yes, they should keep a record, most of the times, they do not have any information on what happened to the previous baby, to know exactly what went wrong the first time, so that any pre natal, suppose if, the earlier baby was born pre mature, if you know there is a cervical incompetency, so that the next baby doesn't come pre mature. Similarly if the mother is Hepta positive (Hepatitis) or something, so you know that you treat that so that the baby can progress into full term and then get delivered, rather than feeding the pre-term 600 gm for three months, and then still not getting a neuro-developed normal baby. So better care from the next pregnancy right from the start itself. So, you know if there is a melingo myelosima, you start from folic acid from today itself, so that before, by the time you conceive the next baby, you do not get that kind of defect, rather than going ahead with neurosurgery and correcting the defect and still landing up to hydrocephalous and meningitis all those complications.

21. What are the potential challenges/barriers for autopsy & MITS in the hospital setting?

See one thing is the timing, once you have declared the death, if you say, ok now you want to go ahead for this for autopsy specially, they think it will take more time, already they have lost the baby and what they are going to gain out of it, so the counseling has to be pre, pre-pre death declaration. When they are in a mindset to accept or have receptance for some information. when they are in the graining/grieving mode, they have to take the baby, they have to go, meet family member, whatever rituals they have to do, so it is difficult at that time to, the counseling has to be prior, and consent form signed, if in case the baby doesn't make it, then we will be taking so and so samples, if that's ok with you, just sign on the form. When you actually declare the death, you don't have to take consent again, because that time its difficult for them to sign, run around there in a grief state. It's difficult. We may actually need you know privacy area, may be a green shield or something, for newborn , it's hardly any space, the baby is on the normal bed, has to be a single bed, sometimes there is doubling, it has to be single bed, anyways baby is so sick, baby is dying, will be on single bed. And then there should be shielding from rest of the babies and whatever because the person who is doing the procedure, biopsies autopsies all that, might need that space, so that space would be needed. And sampling just have to be kept proper, you know proper temperature, storage, labeling, and then transport into the lab, where the diagnosis will be done. These are important…

22. **Any other comment/ suggestions**.

There should be a written information, which can be, you know how much you are saying, parent is able to perceive or how much receptive the parent is, should be in their language, they can go and read it later on, like we counsel once but everything might not resist, they might not even understand the words that you speak, this will happen, that will happen, this, this, this, don’t what all will happen, so whatever you counsel, then there should be a written information, which they can read ok, suppose there is a pre-term baby there are ten problems which can occur, not all the organs can cause problem, but one or either of the organ, but that prepares them mentally that ok, its not just that, the one kg baby is to be made into a 2.5 kg baby. There is lot more than that. And then they would appreciate, how much effort have gone into to bring the baby from 1 to 2.5, and then they would care for that baby if they really want that baby. So, same ways, if it’s a written information then, ok this this this sample they will take and it will be done like this. so, they are not anxious, don’t know what will they do to baby, cut the body, should be a proper written information in their language, that would help. And then follow update when to get back to have the results, if any, discuss, which is not done any of the government set ups.

In-depth Interview- Doctor (Newborn Care Unit) 2

1. **Basic demography**
   1.1. **Designation**- Doctor

Service delivery and communication

2. **Please describe your role related to the clinical care for patients and types of patients seen by you.**
   Ok this is a Tertiary care centre and medical college to basically a referral centre many patient come from like periphery right now working in nursery so to take all the babies in born babies who required the nursery care like birth asphyxia, Hyaline Membrane Disease, apnea, Low birth weight care, middle low birth mid birth care, preterm care or any kind of post registration care put those babies inside and from routine care to intensive care we are giving both type of care and we also see the babies to Shifted to mothers side who delivered normal healthy babies term babies good babies they shifted to mothers side to post-natal to post-natal care 48 hours we also go and see them in the morning.

3. **You come across several patients on daily basis. How do you inform and counsel the parents/ family members of children/newborn under your care?**
   (Probe: Approach immediately after hospitalization, during the course of illness and discharge)
   It seems very important like a counseling of pregnant women's is important as well as accompanying family members is very important to deliver adequate antenatal care and also post-natal care to mother and babies and It is very important to tell them what is the importance of breastfeeding very important to tell them to maintain a good maternal nutrition because that is going to affect babies body weight to be tell them what should be the diet of the mother I was the gyne. people should do this but whenever we come across them and we are to counsel them we tell them importance of breastfeeding it's very important so and the emphasize and re force again and again that as soon as baby born baby should put on breast milk and breast milk should be complete for six month exclusive breastfeeding and what all kind of preparation they need to have the baby arrives they maintain the temperature of the baby and most-most important thing is we emphasis on breastfeeding because that is the time of in the mother and in the family most receptive to said them one important component we emphasis lot on exclusive breastfeeding. <during admission> pregnant ladies or we don't come across the pregnant women we are basically put in the babies when the delivered when the delivered if the babies are admitted to a nursery we explain the relative mother available at that time what the condition of the babies why the babies have come to the nursery what on intervention and treatment of babies receiving approximately how much time we anticipated the baby will in the nursery and what was the prognosis of the baby condition whatever we the condition of the baby low birth weight care we tell them that time the baby will how much weight the baby will gain the we can plan to shift out what will the role of mother it's very important the mothers come and visit and does give the KMC or give express breast milk we tell them these thing. <during discharge> daily there is a time period when relatives father and some other they come to see the baby we explain the baby condition to them and at the time of discharge during around we call the relatives father mother who are there who are taken care bay at home we speak to them to what are the special care needs baby at home and what are all danger sign when need to follow up and what the intervention baby has received in the nursery.

4. **You come across several sick patients, who are at high risk for death. How do you inform and counsel the parents/ family members of critically ill children/newborn under your care?**
   (Probe: How does the approach for critically ill children differ from the regular non-critically ill children, time devoted, frequency, person counseling/discussing with family)
   See we explain them the condition we explain the prognoses on their language we explain the disease on their language, we tell them that how much ratio of the children of disease survive with this grave condition so then they are properly counselled and about babies suffering from. <time devoted> right now I am working over here in ICU almost whole day I have seen a critically ill child. must say approximately hour to patient because approximately there are 6 to 8 patients in ICU. <counselling time for critically ill/regular non-sick> Yes it differs, yes obviously the more time given to critical ones its take time to explain them the condition as compare to the baby was stable and non-critical so.

5. **What has been the hardest part about being a doctor treating sick children/newborn?**
   (Probe: informing/counseling the parents and family members, handling the deaths, etc)
   In cases of sick babies, it is very difficult to explain the Progress to mother and also, we emphasize lots of on hand hygiene practices at home needs to do. <problem counselling to mother or father> it is important that emphasis on the correct practices like breast feeding and hygiene. <during death> No because we adequately explain the prognoses again and again the parents to the family and we show them the babies and we tell them in detail of intervention we have done what the babies suffering from the illness and as soon as babies condition is critical we call them and explain them prognoses and somehow partially prepared them for this.

Death and related procedures

6. **Several of children/newborn under your care die. Please describe the death declaration process usually followed at the hospital.**
   (Probe: How is the death declaration done, place of declaration, time taken, whom primarily targeted, usual reaction from families)
   As soon as baby admitted in intensive care with serious illness we call the parents to explain them to why the baby shifted to ICU and what are the treatment given to baby suppose baby required the ventilation to tell them why the baby required the ventilation what is actually in simple language what is the ventilation and daily we explain them the prognoses to the family and as soon as baby is critical and anticipating the baby will not survive we call them we call the father and mother on hourly basis and tell them this is the condition baby and we are anticipating we try our level best to all these things and this is how we explain them and as the baby dies we call them and explain them to this is the illness and baby has come to this. this we gave the try level best and gave the best possible care that was possible and in spite of all these efforts baby could not make it. <usual reaction> the family is obviously shocking and heartbroken because their loss of baby but and we also console them because what is the illness they have done the best they can do and we have done the best we can do so at least the parents get the satisfaction that the baby received the best possible care you can do it helps at least some extent, it helps them to some extent.

7. **Please let us know about your personal experience of last death declaration in patient under your care?**
   (Probe: Mode of declaration, time taken, family members present, reaction from family members)
   My last experience I have been posted to nursery for last few days only and new born death my experience around a month back. <family member present> during admission or death declaring, mother father even the grandfather<usual reaction> see the baby was very sick we have already prognoses the parents regarding the sickness level so obviously they were very sad and very dishearten completely broken but they have the acceptance of baby death.

8. **What are the challenges encountered by you regarding death declaration in patients under your care?**
   (Probe: explaining and convincing parents/family members, handling reactions, time needed, workload and other competing priorities, support from other colleagues/senior and staffs, security)
   Challenges are like first off all is the acceptance there should be acceptance to be the death of the baby some time there is a denied imbalance and state of shock and some time they don't accept that then he became difficult. <workload> there is lots of workload because there are numbers of patients are more in Safdarjung hospital compared to other government hospital in Delhi its requires more resources should be more number of doctors or team and more numbers of nurses to improve the quality of care.

9. **How your juniors (including trainees), co-workers, and other hospital staffs usually support around death of a child under your care?**
   (Probe: Who supports, what type of support given, any expectations)
   Suppose if you find a difficult case like when there is denial or there is some medical legal issues in the death then we consult them and need a help and sometime senior also arrive at odd hours, <who senior support you> in terms of management clinical judgment in all these things in case of decision making to seizures we consult them and they advise us. See it is a team work one person cannot manage intensive care unit so the work is divided between among others all each one does their responsibility to be best, duties to the best.

10. **How are the children brought dead to the emergency department handled in this hospital?**
    (Probe: assessment, documentation, medico-legal aspects, autopsy)
    Who are brought dead in this department some time, happens in the baby shifted to post-natal side and baby comes brought dead the baby was seen, usually we see a baby once a twice in a day but sometimes the baby go to a sudden death it could be a sudden cardiac arrest, hyperglycemia or Seizure anything that condition is become very difficult to explain but at that time we explain the parents these are the possibility of sudden death of and the baby we also counsel them to postmortem baby of autopsy if they agree with this for it if you find out the cause of that if not then we also do a verbal autopsy like what was the cause how the baby behaving since morning how many time baby has fed how many time baby pass the stool or urine all these things. <other investigations to confirm death> depends upon what is the state of the baby if the baby completely brought dead then other investigations are not possible in that condition depends upon of the how much time the baby has come to us if the baby is

obviously we do the CPR and we try to revive the baby if the baby revive its all the routine investigation need to be done if the baby not revive any kind of investigation is not possible. <documentation> baby documents required because sister will note it down, we will note it down we will make a death form which will certify the cause of death in all these things and CVR form is also filled.

11. **How do you prepare yourself and parents/family members of the child who is dying/dead?**
(Probe: mental preparation, seeking support from seniors/colleagues, informing parents/family in advance to prepare them)
See we obviously did a lot of efforts and it dishearten us also because we putting all our sincere efforts and time and devotion and similarly we also prepare the parents to saying this that they have been doing good for kid and all this is the disease process not in their hand and we all have been trying our best even they have trying best going through all the trauma and we definitely give him a hope that the baby can still make it. <do you prepare yourself> Yes of course if required because every baby is different, we need to decide our self to what to tell the baby what to tell the parents because they will not able understand they need to understand the disease of the baby so it has to be their language at that their time when they are comfortable and they are ready to receive. <support from seniors> yes of course they tell that you tried your level best what are the intervention can we done even we review it what could have should be done and we don't have done for the next time we will go through the review in whole case.

12. **How does the death(s) of children under your care affect you?**
(Probe: mental stress, frustration, work performance)
Frustration on parts like sometime if there is more staff that is dedicated to this work, I think improve the quality of care the frustration is a part of the patients load and ratio doctor to patient's ratio even nurse to patient's ratio sometime the quality is hampered on that part otherwise. <work performance> No, this is temporary actually, this is temporary this is not like a sometime there are too many patients who are coming for similar like brought dead or dying that feel like the problem but we don't let this in daily work performance we perform a duty daily with fresh mind. <death of the child effect of> yes of course it affects us. <how do you deal with that> see we try to improve the only thing that we can help these babies try to improve our care that is how to try to improve. We try it we review it back and back in again what better can we done to prevent this thing. <any mental stress> Yes.

13. **How do you handle the reaction/response of parents/family members related to death?**
(Probe: explanation, seeking support from senior/colleagues, security)
Depends upon like if there is denial then we need counsel them more if, there is acceptance then we console the parents and we put the hold. <security> sometimes otherwise mostly it is not needed cases of newborn because we counsel the parent's lot of counseling and previous things that been taught and prognoses have been explain. So, I have never required in my life.

14. **What is your view about the causes of death, declared and exact/underlying cause/etiology for the children dying in the hospital or brought dead to the hospital?**
(Probe: approach to declaration, past records, mention of cause of death, effort for autopsy)

It is important suppose it is a preventable death or it is a genetic condition in a baby for the next time for the next conception we counsel them what are the precaution need to be taken what testing have to be done it is important.

15. **In your view, how can such deaths be prevented?**
(Probe: information about causes of death, efforts for autopsy, advanced investigations, care seeking and referral practices)
Depend upon the cause you know parents should properly counselled about how to take baby at home when he consults when he come to health care centre most of the time it's very late when parents they should realize they should visit the doctor, it is very important.

16. **Have you received any training in communicating and handling death and critical illnesses? If not what all you would like to have in such training?**
(Probe: any training on communication- formal or informal, reading or self-learning)
Yes of course 3 years training in post graduate and even now. no special training but during post-graduation course only we get this training. We see our seniors they help us and tell us how to counsel the parents and the family throughout the procedure and we also see this day to day life in a duties and this is how we do. yes, the training was informal we don't get any specific teaching and reading materials on the part of counseling only it is the part of it is self-reading, observational sometime it is thought in classes sometimes in formal, formal and informal both.

17. **What has been your experience with autopsy, post-mortem biopsy after death?**
(Probe: Are these being done, benefit, action based on the findings)
Autopsy it is very difficult to convince the parents for autopsy because the cause of death yes once the parents agree to autopsy and it was normal, it was just sudden cardiac death only and that's it. <are these being done>**-** if its requires if the parents are agree if there are also it is only if there is positive response to parents it only consent after the parents, most of the parents do not agree, in only those cases where cause of death is not known or for brought dead patients. <benefits> like a previously mention prevention of death how to prevent the death if know the cause of death then only we will be able to prevent them and if it brought dead or sudden death autopsy can help us with cause of the death.

You might have heard about Minimally Invasive Tissue Sampling (MITS). MITS involves collection of tissue samples similar to biopsy and body fluids from a deceased patient for investigations. The samples and fluids collection are subjected for various tests to identify the cause(s) of death.

18. **What is your view about MITS being used as a mode of identifying cause(s) of death in children/ newborn in your hospital?**
(Probe: feasibility, acceptability, perceived benefit, workload)
See if the parents agree than we should go for the postmortem because that will help us if they don't agree we can still advise them and counsel them to MITS. <according to you which technique is good> more feasible it's definitely MITS. Because autopsy is a time taken procedure so may be. <religious concern> yes of course many parents' sentimental concerns, emotional concern religious concern so many times autopsy is not possible.

19. **In your view, how the parents/ family members can be approached and convinced for MITS?**
(Probe: which health staff should approach, which family member should be approached, timing for informing, mode of communication, place of informing)
See if we explain them the needs to know the cause of death for the betterment of them and their child and for the next conception, I think they will agree. <which family member should be convinced>I think we should go to the father because it is the decision maker of the family because mother is sometimes not in the state to make any decision, she is in shock, we should speak to the person who is decision maker in the family. <what will be the right time> for MITS it is an important to counsel them as soon as baby brought dead and we should counsel them for some time should explain them after declaring the death and give sometime to settle down to accept the death then they explain them the importance of the cause of death. we should counsel them at the time when the child is critically sick, we are anticipating this at the time also we should counsel them and they are also prepared at back of their mind this is the procedure they can help them. <mode of communication> yes, I don't think so it will help because no parents will be receptive for these techniques this is a technique done after the death of the baby so which parent will anticipate death before. <through advertisement> Yes, but there should be proper timing of this advertisement it should be done focusing these types of parents and these types of family which we know which will require MITS. It should be selective advertisement my advice is focusing only a group of population group of people who is required it.

20. **In your view, what could be the expectations of parents/family members if they agree for MITS?**
(Probe: detailed report, convey of the findings, time, cost/money)
Obviously, they expect a positive result and they expect that something productive and helpful for the next baby comes up. written records proper reports should be given them and proper report should be given to them.

21. **What are the potential challenges/barriers for autopsy & MITS in the hospital setting?**
(Probe: procedural, space, manpower, workload, time needed, cost)
The sample processing sample collection and the most important is this the sample processing and sample collection, there needs to be very good sample for this. <manpower> Yes very hectic, we require more number of manpower for this. <extra money should be put in this>yes because tissue collection sample preservative materials and transport of the sample for resisting, so obviously.

22. **Any other comment/ suggestions.**
It is a good intervention of MITS for helping out these babies and we can do this and add these I our work.

Thank the respondent for his/her critical contribution.

In-depth Interview- Doctor (Newborn Care Unit) 3

1. **Basic demography**
   1.1. **Designation-** Doctor

Service delivery and communication

2. **Please describe your role related to the clinical care for patients and types of patients seen by you.**
   Basically, I am acting as a resident physician in this setup and I am working on a management of sick children, sick neonates who are coming to the nursery, that includes treatment charting, cannulation and sampling.

3. **You come across several patients on daily basis. How do you inform and counsel the parents/ family members of children/newborn under your care?**
   (Probe: Approach immediately after hospitalization, during the course of illness and discharge)
   Basically, we start with feeding, we counsel like whenever the child is born, we counsel the mother how to breast feed and how early she should breastfeed started, the appropriate technique of breastfeeding, how to keep the child warm specially in winters and how to notice all the danger signs of neonates like the temperature is falling, or if the child is vomiting, the child is running blue. Mostly we do that at the time like we are working in the setup where it is about neonatal deliveries, so we are approaching the mother when she is in the labor room or operation theater for the delivery.

4. **You come across several sick patients, who are at high risk for death. How do you inform and counsel the parents/ family members of critically ill children/newborn under your care?**
   (Probe: How does the approach for critically ill children differ from the regular non-critically ill children, time devoted, frequency, person counseling/discussing with family)
   <critically ill new born child> basically we counsel both the parents like 1st we call the mother whenever the mother has come we call the mother, and explain whatever the condition of the child is what is the diseased process, what treatment we are giving what medication are giving to change the disease process or to prolong the life of the child then in the evening we call the father and explain the same thing to the father.
   <different between the approaches for the critically ill child verses the non-critically ill > Basically, critically ill children we are keeping in the ICU so we are so that we are attaching monitors to them so that they are in direct surveillance of any doctor or nurses working here regarding other children most of them are whenever they are sick, they are in the ICU otherwise they are kept in two separate rooms where we can take a round after we have seen the ICU.
   <counselling of parents between critically ill or non-critically ill children>mostly we are counselling on the critically ill children in our setup because we have to tell them the condition on the daily basis, the children who are not very critically ill we are counselling regarding but we are doing in the nursery and we are transferring out the child we counsel them how to take care of child bedside and how to notice if the child become sick again.

5. **What has been the hardest part about being a doctor treating sick children/newborn?**
   (Probe: informing/counseling the parents and family members, handling the deaths, etc)

I think the hardest part will be when we are not it there comes a point, we cannot do anything in some children were comes a point like where we have given everything but the child is not recovering, that is the hardest part. <informing and counselling the parents> yaah that also is included in that because at that point we don't know what the exactly we have to just tell them that this is all we can do it and we are doing. **<handling the death>** it is I think we get used to it once it is difficult initially but as we see that we are doing whatever we can but some children they have poor prognosis then gradually sets in and we get adapt to it but it is obviously very hard specially when we are trying our best and we cannot do anything beyond a point then we realize that some things are beyond our dimension also.

Death and related procedures

6. **Several of children/newborn under your care die. Please describe the death declaration process usually followed at the hospital.**
(Probe: How is the death declaration done, place of declaration, time taken, whom primarily targeted, usual reaction from families)
Initially in the NICU setting we are because the parents are not always there inside so initially we give all the CPR but if the child is in going towards their cardiopulmonary arrest and we give CPR we try our best to revive the child and we will wait for a certain period of time because we let the medication take execute so within 15 to 20 minutes if the child is not surviving we will call the parents and we show that this is what has to be done to the child and this was again because we have been counselling the parents about the condition of the child plenty times so it is not very hard at the point of death.
<time taken> half an hour to 45 minutes.
<whom>basically the parents both the parents are explained.
<usual reaction of families>most of them because they have been counselled they have a munch about the condition of the child obviously they will react in a very sorrowful manner but they are not very much shocked about it because most of the time we counselling for a long time.
<if the child is in the last stage how parents react> then it is very difficult for us to counsel them because suddenly the child has come and we don't know how the counsel the mother who has brought the child and how can we tell the mother the child has brought dead so it is a very shocking reaction for them and for us also.

7. **Please let us know about your personal experience of last death declaration in patient under your care?**
(Probe: Mode of declaration, time taken, family members present, reaction from family members)
No, in my presence there is not been any death matter happened, in last one month again whatever death has happened has been very predictable so it has not been very hard to me I always counseled the parents in my duty and so.
<family member present>both the parents were present.

8. **What are the challenges encountered by you regarding death declaration in patients under your care?**
(Probe: explaining and convincing parents/family members, handling reactions, time needed, workload and other competing priorities, support from other colleagues/senior and staffs, security)
Challenging part is mostly the handling the reactions of parents, mostly if they are explained well they will obviously have a sorrowful reaction, they will not be very violent

or they will not be very provoked about it but so, that is why the usual response if they are counselled, but if there is sudden death like pointed out there is child brought death sometimes they can also behaving very violent manner. So, security issues do come.

9. **How your juniors (including trainees), co-workers, and other hospital staffs usually support around death of a child under your care?**
   (Probe: Who supports, what type of support given, any expectations)
   We all counsel the parents together in a supportive manner, it is a team effort whenever a death of declaration taking place, we all stand by and counsel the parents together. < what type of support given> Basically, it is the senior most person on duty who has to declare the death. So, whenever we have SR with us they come and finally declare the death, it is the senior most person.

10. **How are the children brought dead to the emergency department handled in this hospital?**
    (Probe: assessment, documentation, medico-legal aspects, autopsy)
    First we receive the child, we take the vitals we see there is heart or respiratory effort, if there is no heart rate, no respiratory effort then we will start with CPR we will give all the implication on necessary line, we will give amount of CPR, many a times we are able to revive the child, and also we are able to bring the heart rate back but even if we are able to, most of the children who are brought dead even if we are able to revive back by our CPR, they are not able to maintain for more than six to seven hours. <documentation/medico-legal aspect> we counsel every parent for autopsy but most of them deny autopsy. Reasons for the denial are there are religious reasons like they don’t want to open the death body of the child for their own religious reason that time, and many of them say we want child to finally leave if don’t want any cut opening of the dead body, basically they are afraid of the disfigurement.

11. **How do you prepare yourself and parents/family members of the child who is dying/dead?**
    (Probe: mental preparation, seeking support from seniors/colleagues, informing parents/family in advance to prepare them)
    Again, like I told you, most of the time we counsel the parents everyday about the condition of the child, at least twice the day, if it is every sick child we counsel the parents twice the day, so that is what helping.

12. **How does the death(s) of children under your care affect you?**
    (Probe: mental stress, frustration, work performance)
    It is very bad for us, no matter how rigid we appear in the face but in the night when we go home there is always a striking force that child expired on our duty, many a times I am not able sleep because of this fact. <work performance>No, this doesn’t affect the work performance.

13. **How do you handle the reaction/response of parents/family members related to death?**
    (Probe: explanation, seeking support from senior/colleagues, security)
    Many a times I try to counsel the parents again, to give them supportive hand but if they are turning very aggressive then, usually I call the security and take there help because at times parents do turn aggressive.

14. What is your view about the causes of death, declared and exact/underlying cause/etiology for the children dying in the hospital or brought dead to the hospital?
(Probe: approach to declaration, past records, mention of cause of death, effort for autopsy)
In most of the cases whatever we are keeping as the provisional diagnosis we explain to the parents, like in many cases there is sepsis setting then we will say that whenever the child is being fed from the outside milk or whether they are not maintaining hygiene, mostly we keep provisional diagnosis as sepsis so whatever provisional diagnosis we are keeping we will tell the parents. <effort for autopsy>we don't really push the parents, because most of them are already going through the bad times.

15. In your view, how can such deaths be prevented?
(Probe: information about causes of death, efforts for autopsy, advanced investigations, care seeking and referral practices)
It is very broad perspective, it is not the narrow perspective. Firstly, we can at least try to be more vigilant at our discharge whenever we are discharging the children, otherwise we need better skills at the primary care level and also referral practices should be improved.

16. Have you received any training in communicating and handling death and critical illnesses? If not what all you would like to have in such training?
(Probe: any training on communication- formal or informal, reading or self-learning)
We have not received any training per se but every day we are being told by our senior that how to do it, we have not received any training. <what all you would like to have in such training>we will like as what we are getting, like an informal way in everyday basis, whenever we are exposed or maybe one class also could help.

17. What has been your experience with autopsy, post-mortem biopsy after death?
(Probe: Are these being done, benefit, action based on the findings)
No, I have not experienced any autopsy. <benefit of autopsy>we basically look for the cause of death so at least we can ascertain the cause of death, and tell the parents that this is what you have to take care for the next child, that is how we also counsel them also.

You might have heard about Minimally Invasive Tissue Sampling (MITS). MITS involves collection of tissue samples similar to biopsy and body fluids from a deceased patient for investigations. The samples and fluids collection are subjected for various tests to identify the cause(s) of death.

18. What is your view about MITS being used as a mode of identifying cause(s) of death in children/ newborn in your hospital?
(Probe: feasibility, acceptability, perceived benefit, workload)
I have never experienced it but I have only heard about it so, I think it is a good way to prevent the autopsy. The parents who will be willing would know the cause of death of their child.

19. In your view, how the parents/ family members can be approached and convinced for MITS?
(Probe: which health staff should approach, which family member should be approached, timing for informing, mode of communication, place of informing)
We can tell them that it will not lead to disfigurement of the body which they are scared of in the autopsy, we just what to take some amount of tissues from various parts of the body which will not really cause much harm. <timing for informing>it will be when the child is getting critically sick, and we are not assured of the cause, we will start counselling. <mode

of communication>it should be the video but, in our setup, we can do verbally. <which family member should be approached>both mother and father should be explained about the procedure.

20. In your view, what could be the expectations of parents/family members if they agree for MITS?
(Probe: detailed report, convey of the findings, time, cost/money)
I think again they will, like we are counselling them that it will help you in next pregnancy or generations to improve their lifestyle and have a better hygienic condition at home, that is what they are also expecting, they are expecting if they will find the cause they will try to work for it in next time or for the future pregnancies.

21. What are the potential challenges/barriers for autopsy & MITS in the hospital setting?
(Probe: procedural, space, manpower, workload, time needed, cost)
Again, the a lot of load is here like at times we are not able to counsel parents as we have to, like we are not able to reinforce the importance of autopsy to them in our emergencies because there is already a lot of load, I think that is one of the drawback. Additional team would be useful for this procedure.

22. Any other comment/ suggestions.
None

In-depth Interview- Nurse (Pediatric Ward) 1

1. **Basic demography**
   1.1. Designation- Nurse

Service delivery and communication

2. **Please describe your role related to the care for patients in this ward/unit.**

First of all, it is our right to have patient care which is first. What we do in the patient, we do provide medicine, etc., the rest of the patient is hygiene, etc., they maintain, for sepsis, etc. Duty is not the case of nursing it is not man patient care. First of all, everything comes in the patient care, like how you are doing that in the patient with the patient. if any complain do the solution, As if someone is coming, we have to see the children, we have to tell the doctors, all those things are that they have to give medicine time to time, right time right dose. Six hours work is done. If there is a problem of the patient, then there are many problems that we, the people actually have, as if a patient is having oxygen etc., then what happens many times that the patient who is a pediatric child is not able to put it properly So, sometimes we lose sight Sometimes pull the cannula, these are all the complaints that the patient comes to and speak again and again, someone took out the nose, the cannula has removed, all these things, and the fluid keeps going like fluid If we put it in the burette, it ends well, so the patient keeps on saying that the head is over, it is over, there are many such things that the person with the patient tells that there is difficulty in breathing. Is getting, Then they say that there is a patient like diarrhea, so many times they come that the child is coming to the child loose motion again and again, vomiting is happening to the patient with all these complaints and the people with the patient tell us, People come out with their solution as to why they are vomiting, we worry about the doctor who is there, so they say that the doctor prescribes the medicine and we give medicine.

3. **You come across several patients on daily basis. How are the parents/ family members of children/newborn in this ward/unit informed/counseled about the illness and condition?**

(Probe: immediately after hospitalization, during the course of illness and discharge)
When it comes at the time of admission, then we tell about the general condition of the patient, this is the condition of the head patient, he tells about his disease, that this child has a disease, it is a disease. The doctor explains the patient's condition and disease first, after that when we take admission, at that time we repeat the patient that the doctor must have told you all these things. The patient who is many times is extra ordinary and does not ask separately and if asked, then what we have to tell in that, then we tell that which is of doctors level, as if anyone is asking about the disease, If someone is going deeply, then we speak for the doctors that this is not our business, Sir, Dr. Sahib will explain whatever is the problem. Many times it happens that the patient says that Sir, how much time will it take, then the expectation we do not tell them that the disease is possible, it may be cured in two days and it may take ten days, only the doctors explain to them. , Ask us about care then we can tell about care, like many times we ask that sir does not take medicine in the morning, then we see why we did not ask for the cause, then after that we tell his solution for him. <during discharge/or during stay counseling> Counseling, which is the counseling that is done at our level, we tell people like they ask about any medicine that they have to take medicine. No, he counsels the doctor

when the doctor sees the patient take those rounds, at that time he tells us that after that we have very less chance that someone is asking because the doctor explains them. At the time of discharge, the doctor who advises them, the doctor advises them that they understand which medicine is to be given, how much time is to be given, when to be given and for how many days they have to be given, about medicine. We tell them, what is the immunization schedule of them, we tell them at which time the vaccines are to be taken, Those who are vaccinated, they all have to be given routinely, right after that we advise them to follow up, if the doctor has written 7 days, then we tell them that after 7 days you have to show them in OPD.

4. **You come across several sick patients, who are at high risk for death. How are the parents/ family members of critically ill children/newborn in this ward/unit informed/counseled about the illness and condition?**

(Probe: How does the approach for critically ill children differ from the regular non-critically ill children, time devoted, frequency, person counseling/discussing with family)
It is their counseling which is a critical condition, only the doctors explain to them that the condition of the child is because they do not belong to those explanations, they are the doctors who are counseling them, how is the child's condition? They take the consent and get them signed and those doctors will tell that this is the condition if we do not ask people By the way, if we ask, then we are with the doctor like the attendant of the patient is also there, then we tell you that the doctor has consulted you well and if they ask for repeat then we tell the doctor We repeat the same thing in front of us, that the doctor had said this thing right in front of him and in front of him, we also say what the doctor told him.
<approach for critically ill/regular non-critical>There is not much time in this hospital, in fact, because there are three, three, four staff and there is a census of one and a half hundred and a half, then we are not able to give so much but those who are normal patients we routinely take care like Whatever time is their routine time, we give the time, the rest of the time the attendant cares. Well this is what happens to the attendant by telling them that if there is any problem then immediately inform us. So those who are normal patients who are not critical, they get routine care, but in those critical cases we have a man's purpose that if they have a critical case like that, they are on ventilator or ambu bag. If they mean special care, then by going to them again and again, we observe that the reading is going on correctly or whether any medicine is going on or dopamine etc, Whether they are running properly or not, the cannulation that happens repeatedly like the cannula gets pediatric out, all those things mean that we provide a special care to whom we speak.

5. **What has been the hardest part about being a nurse caring sick children/newborn?**

(Probe: informing/counseling the parents and family members, handling the deaths, etc)
See, what we have in such a patient is that counseling is difficult and after death counseling is also difficult, because at times it happens that the attendant of the patient goes into so much depression. Or that they get a little angry that it becomes their thinking that if the doctor did not provide care properly then it is a bit difficult to tell them at that time, Then one is that if someone is a patient, he has died, then even at that time it is very difficult to explain a little bit to the attendant that if we tell those people, what will be understood in front, what will be the effect on that thing. Of, then death counseling is the most difficult.

Death and related procedures

6. **Several of children/newborn whom you care die. Please describe the death declaration process usually followed in this ward/unit.**

(Probe: How is the death declaration done, place of declaration, time taken, whom primarily targeted, usual reaction from families)

Look at the death declaration that is done by the doctors, we are the ones who have instructions from the doctors, we follow them. If a patient is going to sudden cardiac arrest, then doctors call people that what is advanced life support which is like going into cardiac arrest, then to pay ADR or CPR, whatever happens, it is from the doctor. Together we get it done, If after that the patient is not able to survive, then the doctor counsels the attendant that sir the patient will die, and we tell the doctors who is that I have declared what I have now, whatever is done next. They also tell us, our doctors that we.

<what you do after declaration> After the declaration, our job is that we enter and maintain the record in our death record, all the IV lines of the patient, etc., are stocked and we remove them by stock, cannula. Whatever happens, ET tube is all right, after all these clean the body and pack and handover the patient.

<documentation> Yes, in the death register that is our death record, we get those patients signed, they sign so that what we have is clean the body of the patient and handover the patient by packing, we sign on it.

<family member> At the time of the patient who is together. No, the patient has both mother and father, so we do not tell the mother, inform her fathers and all the ladies who are there, we mostly get them out because they become a little too much, neither There is also a ruckus, like crying, all these things, the ladies are only a lot more, soft heart is the mother's, so we give more information to the fathers. If there is no father or mother, then whatever information is relative with him in the gents we inform them.

<reaction> Reaction is very bad and not in a way they say suddenly yes they get depressed and the mother becomes even more like it seems like we are doing CPR and the child stopped breathing cardio arrest I am going in, and if CPR is done by him, then they understand that if expire, then at the same time they become aggressive and cry all these things then they have a lot of reaction.

7. **Please let us know about your personal experience of last death declaration in patient you cared?**

(Probe: Mode of declaration, time taken, family members present, reaction from family members)

Now it has been three or four days, what happens to the patients who are on ventilator or on that, what is the respiratory failure of them, respiratory failures then we are the ones who keep the inotropes on due to what happens. From which his heart continues to beat, as soon as cardio goes into arrest, the heart stops working, then after that he does not survive the child.

<Who declared> Doctors declare in it

<Mode of declaration> No, in patients with cardiac arrest, inotropes are given, like adrenalins is pushed directly, the infusion goes on. Up to three doses of ADR are given. If patient survive with it, it is okay. If there is no response and then doctors tell. For those with a patient like ADR, once the child is done, he will beat the heart for 5-10 minutes, he will do it for 15 minutes because given CPR, ADR will do it, 70% is that heart beat Once the heart

beats, at that time the doctors are counseling that yes we have given the medicine, the child's heart is back but there is no chance of survival, they verbally tell them and take consent from them in writing. When ADR and CPR are done, neither take written consent from them that we have given this medicine to the child and the chances of survival are zero, then at that time they are also told verbally and inform consent is also taken later. They tell them that the heart of the patient will beat for 15-20 minutes, they will not do it after that, they are the last stage and the relatives who are patient, tell them that this is the last stage. We cannot do anything after this because we had already done so much to win, we will not be able to survive again.
<Family member present> Mother-father is the rest and other relatives. Yes, it was the mother and the father, mostly we know the mother-father who is with the patient. In 15-20 minutes because she was given CPR and the patient was given CPR. We gave ADR 3 doses.
<Reaction> reaction, what happens to those whose mother is the same, crying and all these things Yes he is in every death The father is also a little depress, he controls a little or the fathers themselves, because they have to understand all the things, we also support them a little, they also give them psychological support, so they go into a little depress, Tension is enough for the father too, but the one who reacts to them, they are not able to change so much, although they are internally quite depressed.

8. **What are the challenges encountered by you regarding death declaration in patients you care?**

(Probe: explaining and convincing parents/family members, handling reactions, time needed, workload and other competing priorities, support from doctors, other colleagues/senior and staffs, security)
If we declare death, then it is only a little that his patient reaction is the same, it is a little difficult that at times it happens that the patients become more aggressive and they get beaten up then there are more challenges that there is no such atmosphere. Know that there is a ruckus, all these things are there, are we doctors and are we explained together through four to five people, so that even a little patient should have a team working in this thing Yes, it is a little difficult that, it accepts that it happens in it, it is a mother, she is not able to accept that much because I had seen a death, then the doctors had declared the death. IV lines were attached to it, I was myself, at that time I removed it myself, so at the time I was finding it difficult that I could understand her mother, she told them but she was not ready to understand, Father was I understood her. Granted, doctors declare, We gave psychological support to what I have, so the father understood, the father understood that it was okay, but it was very difficult to convince the mother that she was repeatedly telling me that my child has Apply this oxygen, apply this, so that time was very difficult for me How would I be able to explain to him, both the mother and his grandmother were together, he was the father, after that I explained to the father, told the father that if you explain them, he understands them, how can he control Let's take it.

9. **How the senior doctors/consultants, residents, other nurses, and other hospital staffs usually support around death of a child whom you care?**

(Probe: Who supports, what type of support given, any expectations)
The support is that as soon as the doctor has declared it, he is the greatest doctor, it is the explanation of the doctor, only the doctor understands them then when the doctor explained it

to him, the doctors came. We go and tell people, we go to remove those IV lines, at that time they are a little aggressive, so their reaction at that time is different. Just like we put the ET tube to the children, we are giving it to them through the mouth, when we release its ET tube from the mouth, it keeps asking again and again why it is coming out of my baby, why leave You are this, although the doctor explained it to her, but the mother she was, but the mother she is not ready to understand that thing is more, In which we do come out, then later it is a little of the fathers, the father of the child, a little he explains it, controls it, then when the doctor sees this, the doctor is nearby. If you explain that again, we support a lot, there are seniors as well, if I am a junior today, my seniors are also supporting them, they try to explain extra by doing extra extras and give them some psychological support.

10. How do you prepare yourself and parents/family members of the child who is dying/dead?

(Probe: mental preparation, seeking support from seniors/colleagues, informing parents/family in advance to prepare them)

Yes, there is no such thing as mental preparation because it is a bit like we are in the routine of people it happens that how to explain ourselves, we have to be prepared only after seeing the reaction of the patient. In which reaction is the patient or attendant, Sometimes it happens that no one is ready to understand, sometimes it happens that some of them say something to us, they understand it, then their reaction is that they are feeling after declaration at that time. According to how they feel, we are prepared and tell them.

<prepare family in advance> Yes they are in the last stage like that then before that CPR is given to the child because once the cardio goes into arrest, the patient is given CPR after that, then at that time they are supported, they are explained. If that is the explanation then there is not much problem in declaring death later, This does not mean much because we already explain that the chances of survival of the patient are zero, we are trying, and have given medicines, have done CPR of the child, have brought back the heart beat, but It has not come for much time, if the child dies in 15 or 20 minutes, then we tell them what is the result which is ahead. So, it becomes a bit mentally for the patient that yes, we have told people that what happens is thinking it changes, so when death declare, at that time of the patient, they have so much father then we already Only we tell that this is the condition of the patient, and there is no chance to escape because we have done what we had to be in the last stage, then they are prepared, they understand a little, so when they declare Later, they think that it is okay that we have told people.

11. How does the death(s) of children under your care affect you?

(Probe: mental stress, frustration, work performance)

Well, I do so much here, so I have not encountered this much in front of me, when I was in private earlier, I was very mentally disturbed there at that time, then one happened in front of me in Jaipur, I was first private I happened to be in the hospital on the day of Diwali, I was so depressed with myself so much that day I did not eat food, I did not eat food all night, it means a lot that they do not weaken In a way there are family members, it is one that it is a member of the family, those people also feel that it was a member of our family, it depresses me a lot, I wonder why this happens with a newborn baby.

<Work performance> Yes, but there is a death that happens suddenly, then which we could not accept, then it greatly influences that thing.

<frustration>No, it is so much, yes they are enough that so many newborn babies come in it, why is it all, what is the reason for this, we also think many times that what is the reason that becomes so sudden death Babies, many times it happens that the doctor cannot diagnose what caused the death. So this is the most effective effect of how this sudden death occurred with this child, many patients are fine and suddenly they go into such a cardio arrest that they will die in 2-3 hours. Is it more than that, what happened was that what was good with this baby, it was feeding, everything was fine, it died within 2 hours of that thing is very much.

12. How do you handle the reaction/response of parents/family members related to death?

(Probe: explanation, seeking support from senior/colleagues, security)

Reaction is there to handle what is the situation at that time, then according to that situation, we understand them a bit, they are quite that, so it means that how we handle them is so difficult, then others like ours There are more nurses in the health team, they also support them and they also know, so maintain them.

13. What is your view about the causes of death, declared and exact/underlying cause/etiology for the children dying?

(Probe: approach to declaration, past records, mention of cause of death, effort for autopsy)

Yes, so it is important that the child is normal, all these things are happening, so why have there been so many deaths, then what is the reason for them, then it becomes very important to know what is causing all this death. He has his own and that is what the whole world believes, if you believe in your India, then it happens that there are so many deaths happening in India, why is it happening, maybe tomorrow our family will be a member, If we also have children, then all these things are happening, so we are already facing so many deaths, the most common reason which is the reason that we should know, so that we are not too difficult to understand, henceforth We know that we should be well prepared for that, as such cases come many times, many people with CSD, then we know, If there is a problem with cardiac, then we are pre-prepared for what is to be done and what is not to be done, so if the common cause is what we know, then be prepared in advance according to that, sudden death If it is done, what preparations can be made in it, they cannot do anything in it.

14. Have you received any training in communicating and handling death and critical illnesses? If not what all you would like to have in such training?

(Probe: any training on communication- formal or informal, reading or self-learning)

No, when we have a nursing course, we are told all these things, yes there is an admission procedure, a death procedure, a topic that happens, if we have to do any child in it, then how would we do its procedure? What do we tell the patient at that time, how will we clarify if there is death, after that, the procedure that is done is recorded.

<You got this training in S Hospital, where did you get it> No, I got it in Jaipur, I am from Rajasthan, I was from Rajasthan, and from there I did a nursing course, so all this is what I had at my college time. People were taught. No, training was given in our nursing course which was both theoretical and practical.

15. What has been your experience with autopsy, post-mortem biopsy after death?

(Probe: Are these being done, benefit, action based on the findings)

No, there was no such experience from me. <views> Yes what happens in the postmortem is mainly that the death that happens is a reason to know that when the patient has died, what is the reason that he has been the most man then the other is that if someone If the patient or the parts are not damaged, then someone else can be used in the organ donation. No I do not know

< You think the child should have a postmortem> Yes, if you want to catch the cause of death then it should be so that we, who should know what, caused the death, because if it is not, then you will not know for what reason has died.

<Benefits> Now I don't know it

<religious concern> No, I don't think so because if we do postmortem, whatever is cut, then by suturing it back and packing it, it is not like if we are doing postmortem in which a part is being cut or cut. He cannot be left openly and not left like this, when suturing him from back then he comes back to the same stage, almost comes to the same stage as when performing surgery of a patient. So this is also equivalent to a surgery that if you are cutting, then you are suturing in the surgery, in the same way that you are doing postmortem, if you are cutting, then you will give it by suturing it; Not that we leave openly, even by saying that it is not so.

You might have heard about Minimally Invasive Tissue Sampling (MITS). MITS involves collection of tissue samples similar to biopsy and body fluids from a deceased patient for investigations. The samples and fluids collection are subjected for various tests to identify the cause(s) of death.

16. **What is your view about MITS being used as a mode of identifying cause(s) of death in children/ newborn?** (Probe: feasibility, acceptability, perceived benefit, workload)

Yes, it will be better than postmortem because there will be no one who is in it, disfigurement is better, it will also be known (death reason) in it.

17. **In your view, how the parents/ family members can be approached and convinced for MITS?**

(Probe: which health staff should approach, which family member should be approached, timing for informing, mode of communication, place of informing)

In this, the person who is a male role father and others who are male attendant, the relatives who are relatives are more that those who are relatives can explain both father and mother because we are the father of the patient. Even in depression, they will not allow that thing to be done because most of the attendants say that what happened has happened now, when we are no longer our child, then whatever we have is not our only What is the reason for knowing the reason, he will come back, all these answers are there, then what happens in him, then that those who are relatives should explain about this thing, brother who is a relative, they can convince him that this child is right. The relatives understand that they are not able to be ready, but for the children who are going to be there, there are no such problems inside them. If they understand his father and mother, then it is more important that there is no one who is born to whom we speak.

<who should approach> Yes, the health workers who are in it, if they tell more of the doctors in it, then they will soon agree, those who are senior doctors believe more because they are senior doctors, they have enough experience to get them done.

< Which time> it is in that after death we tell them that they are more relatives of the patient and their reaction is not a little towards them as much as towards a live patient if they are

going to die. But if it has not happened, then consider it alive, at that time no attendant or any parents will believe that if I do this with my child, then it is in this that after death we tell them that it is okay for the child to die. It is then that if they understand that this patient is a patient, then his patient has died, but to find out what was the cause of his death, he will take some samples from it, then a report will come out of it, then it would have been much better.
<mode of communication> Yes, although we can tell about the health education that happens in it, such as the patients who go to the hospital, etc., they also have doctors to show them, they also understand that there is a problem that such a person should have it should be done, it should be done like that, but like many ADS are running on TV, on radio, everything is said, so nowadays people who are living nowadays, they believe these things more. That what is being shown on TV, what is being told in the radio, then those who watch on TV nowadays do not even believe in the doctor, 20% who will be there or not people who know that yes is getting fever now Which tablet should be given, now the ads of crocin are shown to be a headache, a headache, a complete rest, a quick rest, take a crocin. If so, they believe more of that thing, there are many meanings whatever methods. Mostly to understand, they are ads etc. It should be shown in this so that everyone can have a tail, there is any health worker if someone is going to tell this thing, then every member in every house is not able to ask if he is asked. For one, there is a shortage of time and there are many problems which cannot be asked if the method is the best one. If this is the case, then show it in ads, if this is happening with a patient, then this thing should be done and as if they were showing before the sterilization was seen everywhere, get the prohibition done, paste it in the back of every vehicle. , Without incision, without him, so what awareness increases from him, then according to me, the most is that you show it in ads.

18. In your view, what could be the expectations of parents/family members if they agree for MITS?

(Probe: detailed report, convey of the findings, time, cost/money)
Except, they will do it that they are doing better for us, they are doing better for our child that it is okay that he has died but what is the reason for his death so that he can know. Yes, their expectation will be that they should be given the reports that are in the return so that they feel that yes our child has been mits because of his death because they have to keep their records till then. If you know, they also have a record and then the next one is a baby Some people have it as if someone has a first baby, someone has a second baby, if someone has a first baby, he is dying, then it would not have happened that he would not have further babies or if he would have babies. Their record becomes more important that if the first baby is our expire then what is the reason for his death, if there is a record of that, then the next baby which happens or when the mother is pregnant, then whatever gynae at that time would be the doctor She also asks her history that there was a baby before, There is a baby, how many babies were there, how many are alive, how many are live, and how many deaths have happened, when they take that record from the mother, then they tell them that yes they had a baby. For what reason did it happen, there was no such birthright, due to which he died, then at that time it is his job that the record which is his report The death, which is also useful at that time, shows the doctors, then they have proof that in a way that we had a baby, due to this, they died, the doctor also gets a sense that yes it is Death was due to reason, otherwise it happens many times that the doctors feel that due to what happened, then both the mother-father, the mother tells something else and the father tells something else, many times they

lie No sir, if our children are not there then they get a wrong information. To the doctor, gynae, then if someone had a congenital problem then it has happened because of that and Mother Father said, "Sir, he had no such problem, then the doctor believes what he is saying verbally because the doctor knows Does not happen that he obeys mother-father then when the delivery is If the same problem is happening with that baby at that time, then the doctor also has a problem in understanding why this is happening, because the mother-father had not told anything like this, then this wrong information The doctor is not aware of doing anything else.

19. What are the potential challenges/barriers for autopsy & MITS in the hospital setting?

(Probe: procedural, space, manpower, workload, time needed, cost)

If it is turned on here then the biggest problem is that the parents of the patient will be the most difficult to explain to them, this problem will be because they will be told how to do their sampling or they will have this procedure. It may be the most difficult to explain it.

<Manpower> Yes, this thing will have pressure of this thing, one is that there is not so many crowds here and there is so much patient that we do not have time already, we do not already care so much in the patient. As much as a patient except that my child should be cared for so much, he is not able to do it already because the most is that there is a shortage of staff, now there is no staff, now you see Jake is 120-130 census Emergency is going on simultaneously, with three staff and four staff, it is the highest of the time, and if the workload is not able to do so much, then if it is operational then there will be more workload. If it is included, we do not say that it should not happen, it is good that whatever time is coming out of us, then for that we will take time out and will have to leave, if it is going on, it is our greatness we will remove it. Yes, it is for this that if there is a separate team for this, it will be better, especially if they will be trained like they have to take the sample, now everyone has to take a sample of that thing, then the sampling could not be done properly, then the reports were wrong. Will come, so if it has a separate team then it will be better because they will be trained specially for this thing, then it will happen and it is important that a separate team is formed for it then it will be better.

20. Any other comment/ suggestions.

No nothing, add that instead of postmortem, it is coming with mits it should be better, it should be from my side, I will try my best to leave it as long

In-depth Interview- Nurse (Newborn care unit) 1

1. **Basic demography**
   1.1. Designation- Nurse

Service delivery and communication

2. **Please describe your role related to the care for patients in this ward/unit.**
   Our baby has two to four responsibilities like a little baby, if there is an adult check, then you have to check, sex, of course, the attendant must be together, the attendant also asks for sex, its father's name, etc. identifies Is, verifies, verifies what she writes in the file, come here, then the label, such as chest labels, hand labels should be attached to the child, so that the child does not move around, there is a lot of responsibility. Yes, let me explain the routine, by 7:30 I will come, Here, I check the entire inventory, after this, when Aarti gives handover, after her handover, we give the baby to the mother for breastfeed in the morning, after that whatever baby Now she is fed milk again by making toffee, and also advises the spunch, after this the doctors will come for the rounds, will put new orders to carry out, and prepare for every trolley, in every shift we give two times changes diapers.

3. **You come across several patients on daily basis. How are the parents/ family members of children/newborn in this ward/unit informed/counseled about the illness and condition?**
   (Probe: immediately after hospitalization, during the course of illness and discharge)
   Behavior, many people will be illiterate, they will have to do more and more, as the visitor time is there, if the father has to match, then he sees the mother and father, the sick patient sees the mother and father, if the mother feed If you are not very sick then you will not show the father because the infection is chance, the visitor time is here from 4 o'clock to 5 o'clock, at the same time you will show the father if the sick is sick. Then we will show the Father also. If more seek is being done, then our ward information book (information) is written in the ward information book, the mother sends it there, by sending it, we inform our sister that the sister informs them, if it is too sick then Later we will talk on the phone and call him, then the doctor will do a condition explain. <discharge>The process of discharge, before the child is discharged from here, we send a slip in the ward, the mother discharges and notches, the sister is there, she will send it back by writing, if the mother is discharged, the child will also be discharged from here. , And those who will apply vaccination at the time of discharge. And then the whole identification from the mother, because she identifies twice, looks like sex, Sarah identifies something one by one etc, Chest Label, then going on home after discharge on whatever she tells it all, and the immunization schedule, and the horoscope et cetera.

4. **You come across several sick patients, who are at high risk for death. How are the parents/ family members of critically ill children/newborn in this ward/unit informed/counseled about the illness and condition?**
   (Probe: How does the approach for critically ill children differ from the regular non-critically ill children, time devoted, frequency, person counseling/discussing with family)
   At Very Sick, at the time of Sick, at the same time, we will call Mother and Father and the condition doctor will do the exercise.

<Who explains the condition> those who are PGs or do not have two PGs in 24 hours, they will do the exams, SR will explain if it is in the morning time.
<approach for critically ill/regular> Critically ill, care will have to be provided with ventilator care, if the child is on ventilator critically, and he will also be given fluids and give care by doing ABC, see airway breathing circulation. Gives a little empathetic care, gives a little empathetic approach, if the mother has to show because the baby will not be in the incubator anyway, breastfeed is not needed, yet the mother is shown by speaking if she needs to spend a little time. Let's give. The one who is more strict will have to pay more care, more attention will have to be paid. We do not consult doctors, doctors express all the conditions, they will tell all the conditions and what will happen next, they will talk about it and will take high risk consent for whatever high risk consent is required.

5. **What has been the hardest part about being a nurse caring sick children/newborn?**
(Probe: informing/counseling the parents and family members, handling the deaths, etc)
It is difficult to handle the reaction of the mother at the time of death. Some people believe that some are a little difficult to explain.

Death and related procedures

6. **Several of children/newborn whom you care die. Please describe the death declaration process usually followed in this ward/unit.**
(Probe: How is the death declaration done, place of declaration, time taken, whom primarily targeted, usual reaction from families)
It is the doctor who declare, SR who is in nursery 5, if it is morning time, SR till 2 pm, which is probably till 4 pm, after which if there is death then the SR in nursery 5 declare.
<How death declaration is done> To declare, first it should be complete pulse rate, breathing, whole heart etc. should be zero and after that we should stop. The behavior of doctors is good, cooperative.
<place of declaration> Mother will call her, if she is in the ICU, she will declare here and after that she will speak to the parents as if the child has been expired, parents will be told to father, not mother to father. <Time taken> after the death, parents will speak at the same time and after the expire, after the complete closure, the parents will speak and declare. <Reaction> Will they be sad at that time? At the time of admission, the father's mobile number is completely taken, meaning if there is anything, he will speak to the father, if there is no father, then whatever responsible person will be told.

7. **Please let us know about your personal experience of last death declaration in patient you cared?**
(Probe: Mode of declaration, time taken, family members present, reaction from family members)
Right now my last week is done. In the <who declared the death and declaration process> SR had declared it, SR has declared at the time of death, the declaration process is that its heart rate was completely closed, not even maintaining on the ventilator, Then Sara resuscitate after it is over, after Sarah is done, summons Father, and calls Father to explain. <Reaction of father> Father had accepted, already the child was running so sick, so he had accepted.

8. **What are the challenges encountered by you regarding death declaration in patients you care?**
(Probe: explaining and convincing parents/family members, handling reactions, time needed, workload and other competing priorities, support from doctors, other colleagues/senior and staffs, security)
What is sometimes at the time of declare people do not admit, because someone brought dead comes from the ward brought dead, mother and father do not admit how it happened so far, it was okay to speak like this It is then seen to explain them. <Security> at that time there is no such thing ever.

9. **How the senior doctors/consultants, residents, other nurses, and other hospital staffs usually support around death of a child whom you care?**
(Probe: Who supports, what type of support given, any expectations)
At the same time, we have to give a lot of life saving medicine, we are very busy, we give life saving medicine at the last time and whatever is going on in its weight, its rate will have to be reduced and if we want to pay CPR, then we will have to pay CPR, that doctor We support. At that time everything works together.

10. **How do you prepare yourself and parents/family members of the child who is dying/dead?**
(Probe: mental preparation, seeking support from seniors/colleagues, informing parents/family in advance to prepare them)
All the counseling is done by a doctor it is a doctor's job. The doctor keeps telling mother and father about the condition of the child from the very beginning.

11. **How does the death(s) of children under your care affect you?**
(Probe: mental stress, frustration, work performance)
It happens at the personal level, we also have a small child, it is so, but after a while. <stress / work performance> At the same time there is stress, yet we overcome because the rest of the children have to see, if they remain under stress then how will they see the other children, in the same way they overcome.

12. **How do you handle the reaction/response of parents/family members related to death?**
(Probe: explanation, seeking support from senior/colleagues, security)
If something like this happens then only empathetic care will be given, empathetic approach will be done. The rest of the doctors are there to handle, the parents are sad at that time.

13. **What is your view about the causes of death, declared and exact/underlying cause/etiology for the children dying?**
(Probe: approach to declaration, past records, mention of cause of death, effort for autopsy)
Yes, the cause tells which death is done. This may be due to the fact that they do not know the exact cause, which are the risk factors, nor do they explain completely. <effort for autopsy / postmortem> Sometimes the parents tell us to get the post mortem done, to get the post mortem done, yes, even for newborns. Whoever brought dead, their fathers say that we have to do post mortem it should be known what has happened because the child was still

talking. Post mortem has only one experience, a child been sent in such a way that I have not heard before you.

14. **Have you received any training in communicating and handling death and critical illnesses? If not what all you would like to have in such training?**
(Probe: any training on communication- formal or informal, reading or self-learning)
Yes, if there is a child in the training period, at the same time what is the response of the entire family, they will not accept it, then it will be like bargaining, then after going to the last stage, they will accept the grief response of death. The stage of the same was told at the time of nursing course. Later such training is nothing.

15. **What has been your experience with autopsy, post-mortem biopsy after death?**
(Probe: Are these being done, benefit, action based on the findings)
One was on our training time experience but he was the adult death. He was probably an accident. <Benefit> exact region or cause of death is known. Proper region is also able to know from this.

You might have heard about Minimally Invasive Tissue Sampling (MITS). MITS involves collection of tissue samples similar to biopsy and body fluids from a deceased patient for investigations. The samples and fluids collection are subjected for various tests to identify the cause(s) of death.

16. **What is your view about MITS being used as a mode of identifying cause(s) of death in children/ newborn?** (Probe: feasibility, acceptability, perceived benefit, workload)
I am guessing 60% wouldn't believe that. <Why> is an emotional region, they will not touch the child whether he or she has some other procedure. <Perceived benefit> benefit is the same that the cause of death will be known, and those who want to get 40% will be educated, so they can agree. <Workload> I think there will be an increase for doctors, here the staff nurse does not do the sampling, the cannula sometimes does it, but any invasive procedure is done by the doctor. This will increase the doctor's pressure slightly.

17. **In your view, how the parents/ family members can be approached and convinced for MITS?**
(Probe: which health staff should approach, which family member should be approached, timing for informing, mode of communication, place of informing)
Whenever the child is very sick, then the doctor should approach at the same time, the sick tells the condition at the time of admission and from 4 to 5 pm, whatever the ICU child is, whatever his father or mother is, his condition doctor. By sitting and telling the whole, what is the status.
<When should they approach> you can tell after death immediately. <Which family member should they approach> the father only has to do it because the consent is given by the parents. <Who should approach> consultant can never come or cannot come at all times, then SR can do, they will understand SR's point. <Mode of communication> they can tell things only by thinking, they can explain them.

18. **In your view, what could be the expectations of parents/family members if they agree for MITS?**
    (Probe: detailed report, convey of the findings, time, cost/money)
    Whatever the expectations, if the doctor does not tell the outcome class, then it will show the reason for it, then it will be known that there is a genetic region. Can also be done.

19. **What are the potential challenges/barriers for autopsy & MITS in the hospital setting?**
    (Probe: procedural, space, manpower, workload, time needed, cost)
    The challenge will be that the illiterate people who are illiterate will take more time to counsel them as they will not get 100% success rate every time. A little counseling will be seen, it becomes difficult to explain to the illiterate people. <Workload> manpower will be a challenge, there is a shortage of doctors, and shortage of doctors is also shortage. <Time needed> is the same as shortage of time is sometimes.

20. **Any other comment/ suggestions.**

In-depth Interview- Support Staff Nurse (Pediatric Ward) 1

1. **Basic demography**
   1.1. Designation- Support Staff

Service delivery and communication

2. **Please describe your role related to the care for patients in this ward/unit.**
   I have to do mop sweeping, dusting and nurse orderly as well. When there is no staff here, then that job also has to be done. And if a child is serious then he has to run away with it, sometimes he has to leave and come up in ward.

3. **You come across several patients on daily basis. How are the parents/ family members of children/newborn in this ward/unit informed/counseled about the illness and condition?**
   (Probe: immediately after hospitalization, during the course of illness and discharge)
   When it gets a little normal The doctor then talks about when he comes in a little normal, only by chance, if he is serious, he has an ambu bag, if he cannot speak then what will the doctor talk about, then he will treat him first. He does not talk about the treatment of the first child, after the treatments when he is normal, then his parents are concerned.

4. **You come across several sick patients, who are at high risk for death. How are the parents/ family members of critically ill children/newborn in this ward/unit informed/counseled about the illness and condition?**
   (Probe: How does the approach for critically ill children differ from the regular non-critically ill children, time devoted, frequency, person counseling/discussing with family)
   <Time devotion> some children get full time and some even expire. If you are very serious, then the doctors are not able to save him, they do their best, they offer glucose, give these medicines, give injections, try their best, from above.
   <Person counseling / discussing with family> they also talk to the family members, in some serious children, they speak time and time.

5. **What has been the hardest part about being a staff caring sick children/newborn?**
   (Probe: handling death, day-to-day care)
   No, Sir ji is not visible to us, if the child is more serious, then if our nursing is not orderly, then it is like applying oxygen to the child and the doctor nurse is not orderly, then I also put oxygen on him. When the nurse is not orderly, it is necessary to avoid the child who is unable to breathe, which will breathe if there is no oxygen.
   <handling death> There is nothing like this at the time of death, when the death is done then those who have parents, they cry and do what they can see., His paper goes away.

Death and related procedures

6. **Several of children/newborn in this ward/unit die. You might have observed death declaration for several children/newborn. Please describe the death declaration process usually followed in this ward/unit**
   (Probe: How is the death declaration done, place of declaration, time taken, whom primarily targeted, usual reaction from families)

No, they do not declare at all, declaring completely makes them difficult to handle, because what is it that they declare that your child is over, they may have a heart attack, any patient is like our It is in the heart or somewhere that it has been declared completely that if your patient is over, then what was there in it, others who were with him, his wife is his father, he is his mother, he feels absolutely shocked. So they can also have a heart attack. We go to the ward and speak to his father and tell him that such a thing has happened we could not save him sorry.
<Time taken> Now even after hours we talk or take half an hour or one third of hour, we cannot declare at all.
<Primary target in the family> whatever their daddy is, they take him to the side and tell him alone that such things have happened, do not tell the ladies so soon they wait for their (child father) father is.

7. **Please let us know about your personal experience of last death declaration you observed?**
(Probe: Mode of declaration, time taken, family members present, reaction from family members)
It was just a week ago, so he was expire at night, in the morning his parents came to Daddy and told him, as it was 2-2.30 in the night, it was 4 o'clock in the morning in his written reading their parents come.
<Mode of declaration> those who were doctors on duty, they did it they do what they do on duty.
<Reaction from family> I am no longer a child, cry whistling is done, it is normal, in India. This is how it all happens.

8. **In your view, what are the challenges encountered by the doctors/nurses and other staffs while death declaration in patients?**
(Probe: explaining and convincing parents/family members, handling reactions, time needed, workload, security)
What do you have to face, what can doctors do right here, doctors do their best to save, they try their best as far as possible, I support them in that too.
<What is your support> You can give it to those who are young children, that there may be an accident from outside, in the OT square, like taking it to pieces, then putting it on the stretcher, then applying oxygen or doctor Speaking to it, it comes quickly and offers treatment. Whose work is done quickly.
<Security>nothing like this happens. Yes, sometimes it happens, there was someone in the heart, so we had done with us, the doctor was a big doctor, the senior was such a fat man, and he had tasted expire, so we were giving him pressure. When he was giving pressure, he got a hiccup, and was treating him further, then he was all tired, he was very fat, there were many doctor ladies, he (doctor) asked me to give some pressure, so I pressure Started giving, meaning it was over, And she was also his wife, she had even gone to the name of me, her Mrs. It was, she did not know the name, after asking someone, she said that she killed my husband in such a way, then did the police come to the ward, then the doctors came, what is the matter, said that there is nothing to expire He was tired, tired of giving pressure, he was giving pressure, and he was already dead, the doctor said.

9. **How the senior doctors/consultants, residents, other nurses, and other hospital staffs usually support each other around death of a child/newborn?**
(Probe: Who supports, what type of support given, any expectations)
Big doctors come, they tell everyone to do something, first thing in children, parents themselves know that our child is not going to survive, we are trying, if something happens by trying Okay, what else can we do.

10. **In your view, how are the parents/family members of the child who is dying/dead prepared for death declaration?**
(Probe: informing parents/family in advance, explanation)
See it is like this, you give him comfort that you are not disturbed, we are giving treatment, maybe we can save the child, we will try to save the child, and if it is not left then leave it over the Lord, where You could even try, that of the doctor, that of every employee, they were saving the child, it would have to start preparations a little in advance. One of our children had brought a child from Gwalior, the doctor there had agreed, all the doctors there had agreed that you had gone to S Hospital to show that the child had escaped here, the doctors did full treatment here.

11. **How are the reaction/response of parents/family members related to death handled?**
(Probe: crying, outrage/fight, question)
In that case, it is handled with love, as our family comes to their parents, grandparents come, then they are told that your child has finished expire, and the more curry we tried, the longer Next up is the will of the person, so what can we do now. They themselves also see that the doctors are doing this much to us, so what will they say.

12. **What is your view and experience about the autopsy for children/newborn dying in this hospital?**
(Probe: Are these being done, benefit, action based on the findings)
No, what will be the post mortem of a small child. Yes, if someone who is above the age of 15 years has eaten some poison, because nowadays children are lying in medicine, they eat it, some expire medicine, then they get poisoned, then after that they expire. Is, we also try to put the tube itself so that it survives, try to put the pipe inside the mouth, make the glucose square so that it is turned back and it survives, and if it expires then its police case Will, then he knows about post mortem.
<In case of small children, post mortem is conducted> It is like what will be the post mortem of the young child, then nothing will remain of it, neither it will be used to burn, nor will it be used for making or burial, then what will be the post mortem of the small child. No, a child does not have a post mortem. If someone is someone with MLC, if they have taken something, who is 5-6 years old, what post mortem of someone who is born, if a child accidentally drank then make his MLC, that's it I am Telling first thing, Madam, like we have a one year old child, what will be the post mortem of a one year old child, he cannot rise or sit like that. And the first thing is that there is nothing left in the post mortem, some will come out of the mind, the heart will come out, the kidneys will get everything out, then it is seen in the post mortem what is not there.

In some instances, autopsy/ post-mortem is not possible due to various reasons. To find out the exact cause of death and underlying disease, some tissue biopsy (collection of very small sample using needles) and fluids (blood, urine, etc.) from the body, as done for biopsy. This procedure is called as Minimally Invasive Tissue Sampling (MITS).

13. **What is your view about MITS being used as a mode of identifying cause(s) of death in children/ newborn?**
    (Probe: feasibility, acceptability, perceived benefit, workload)
    In the case of a child, the family members do not believe that there will be nothing left in it, if such a big child is there, what will be left in it if they squirm. If you take a sample of blood by needle, it can hurt, then why the family will not accept it. Because the family members themselves know that there is nothing left from the post mortem, neither will it be useful for burial or for burning. I have never been there in front of me, I am a great done who is a done, how can I tell, yes, what did I tell you that if it is 15 years old then it is possible to be a child of 5-6 years, playing at home Something is kept in acid, acid is kept, something which is used to clean the lettering, he drank something or any chemical is kept, he drank it or the rats got the medicine for killing them, the children who took out the search, they ate it. His post mortem is a police case.

14. **In your view, how the parents/ family members can be approached and convinced for MITS?**
    (Probe: Who/which health staff should approach, which family member should be approached, timing for informing, mode of communication, place of informing)
    I told you this earlier that you should speak to your father or your grandparents and tell them that they are sensible, they have to believe brother, now they have done whatever treatment they want to do with the doctor, What can we do now is spoken to them, then they understand lovingly, whatever the son is.<which health staff should approach>SR, JR is unable to speak, only SR will speak this thing or those above them also understand it with love, they will speak to those who are rightly supported as you are sitting. It is not like saying that your boy is finished, nothing like that, when the child expires, he will be treated with love, then he will understand why he is doing this, what happens with the world now. To be done. So let's understand what is happening.
    <mode of communication> No one can talk with the video, just like that, you can try and say, I have tried my best and I have not put any effort in it, in medicines or in any test or in anything, so sorry for it, what can we do Can do this, then can tell about this technique.

15. **In your view, what could be the expectations of parents/family members if they agree for MITS?**
    (Probe: detailed report, convey of the findings, time, cost/money)
    No, madam, this is a test that is done as if there is pneumonia or kidney is bad, or something is inside, then if we do not get the test done, then what do we know about the disease inside the child, like we do CT scan , MRI makes it happen, what happens to him, the whole condition comes inside him, what happens is the whole video comes inside him, after watching the video it is known that this child is lacking in this, Or as if there is a security in the heart, you will not know what is there in the heart, what is not there, until it is complete, when it comes to know that it is in the heart, it should be done only then . Just like there is

hunger, where they are hungry, somewhere is chikungunya, there are many dengues, some how we will know hunger till we get it tested.
<detailed report>Madam, when we get blood tests done, why do x-rays, so we have this child that is lacking in this and it will be treated and admit it, so its treatment will be good here, treatment will not be at home . The cause of death, like the blood test that we are doing, the blood test showed that it is chikungunya so we will treat chikungunya and that is the treatment of chikungunya, If our blood becomes water, blood will become water, now we have a small child, we will stop it from blood, it will be corrected with medicines, or else it will be done by technology, the first thing they will do is stop it, and If not, then it will be better then.

16. What are the potential challenges/barriers for autopsy & MITS in the hospital setting?
(Probe: procedural, space, manpower, workload, time needed, cost)
If there is a police case, it is not biopsy here if you do not have a police case then it is not biopsy. The needle does not show, if the machine comes, technicians will come, then it can start again.
<challenges / barriers> What is the problem, when the machine will come, there will be a blood test, after doing the blood test the automatic will know what was the reason.
<space /manpower> There is so much in S Hospital, there is so much space lying in S Hospital that no one else will be in the hospital, now your OPD is lying empty, where the heart belonged is also empty.
<Workload> pressure will increase, when our people will increase, when our machine comes, then technicians will also increase, doctors will also be on it, cleaners will also increase then everything will increase.

17. Any other comment/ suggestions.

In-depth Interview- Support Staff (Newborn care unit) 1

1. **Basic demography**
   1.1. Designation- Support staff

Service delivery and communication

2. **Please describe your role related to the care for patients in this ward/unit**.
   My routine is the work of the attendant, which is also done here like dusting, cleaning, and doing X-rays of the children, fetching blood, dropping samples, which means the job of the attendant. Apart from this, those who do their work of nursing attendants do the same.

3. **You come across several patients on daily basis. How are the parents/ family members of children/newborn in this ward/unit informed/counseled about the illness and condition?**
   (Probe: immediately after hospitalization, during the course of illness and discharge)
   It is a doctor's job, not a doctor's, sisters. Sometimes when they come, they say that they wait for two minutes, five minutes, the doctor will come and they will check and tell you, if they come, they will tell you. This is our only thing.
   <when interaction happens> One or two days go as if they never come to ask what the condition of the child is because there is no one on the counter, we are there, so say, wait two minutes, our doctors will come or nurses will tell about it. Here, there are no more days admitted, it depends. Means one week, yes, that's how it is. Depends on the condition.

4. **You come across several sick patients, who are at high risk for death. How are the parents/ family members of critically ill children/newborn in this ward/unit informed/counseled about the illness and condition?**
   (Probe: How does the approach for critically ill children differ from the regular non-critically ill children, time devoted, frequency, person counseling/discussing with family)
   Well, the doctor only tells about those who are very sick children. <Approach for critically ill children differs from the regular non-critically ill children> There is only one time, parents who are more ill children are also called separately. <now does doctor interact in NICU, as parents are not allowed inside> call them and talk outside at the counter.<time devotion> There is no time created here, which means from 4 o'clock to 6 o'clock, the one who wants to ask in it, because they do not allow nursery parents like this again and again, because of this it is made from 4 to 6 o'clock It is the condition in which the child is asked tail, if any condition is bad, then our doctor calls and calls the child for that too, then calls the parents separately.

5. **What has been the hardest part about being a staff caring sick children/newborn?**
   (Probe: handling death, day-to-day care)
   There is no such problem, but it is sometimes the load on the work becomes more, the staff is less, and then there is a little difficulty. By the way, it is not difficult, but it is difficult to manage a little when one goes to work and another emergency comes out. <Handling death / day-to-day care> there is no problem in that.

Death and related procedures

6. **Several of children/newborn in this ward/unit die. You might have observed death declaration for several children/newborn. Please describe the death declaration process usually followed in this ward/unit**
   (Probe: How is the death declaration done, place of declaration, time taken, whom primarily targeted, usual reaction from families)
   Big doctors do, declare death. In doctors, the same SR is mostly done. After being <time taken> in the last bit, declare.
   <Who primarily targeted> declare father <usual reaction from families> reaction, they are aware of that, they are given information about what the condition is, this is brother. Let's prepare, what is the condition and what is going on. Never fight or fight, it has not happened.

7. **Please let us know about your personal experience of last death declaration you observed?**
   (Probe: Mode of declaration, time taken, family members present, reaction from family members)
   Probably two or three days ago. <Who declared death> SR only.
   <Family member> Father was present from his family. <Reaction of family member> what was the reaction emotionally, whatever they are, but they are not given this information beforehand, nor are the conditions of the child, etc., what our doctors keep telling them.

8. **In your view, what are the challenges encountered by the doctors/nurses and other staffs while death declaration in patients?**
   (Probe: explaining and convincing parents/family members, handling reactions, time needed, workload, security)
   Nothing like this is visible, most understand, they understand the parents, no such problem is there.
   <Explaining and convincing parents / family members> our doctors comfort them, not such a thing.
   <Security> no nothing like that. This has never happened in a 13-year career. <Workload> everyone works so hard here, no one can even know.

9. **How the senior doctors/consultants, residents, other nurses, and other hospital staffs usually support each other around death of a child/newborn?**
   (Probe: Who supports, what type of support given, any expectations)
   To tell the family what they do, they also inform them on the phone, call them, information is also given to them where the patient is admitted then information is also given in a written form. (Written information for whom) The ward in which the mother is admitted.

10. **In your view, how are the parents/family members of the child who is dying/dead prepared for death declaration?**
    (Probe: informing parents/family in advance, explanation)
    Mother tells her family members about the child. If this had happened, we would have called the mother and told, then it would have been difficult to handle the mother, so we would not have called the mother, she has a father or if her family members are not father

<informing parents / family in advance> It depends on the condition that the condition of the child is getting bad, then tell the parents that don't give breastfeed, the child is not coming in the improvement, this way. <Explanation> yes complete explain is done, explain is done in every way.

11. **How are the reaction/response of parents/family members related to death handled?**
    (Probe: crying, outrage/fight, question)
    The parents would have been unhappy with the family. Do they ask that the doctors explain it beforehand? <Outrage / fight> never happened.

12. **What is your view and experience about the autopsy for children/newborn dying in this hospital?**
    (Probe: Are these being done, benefit, action based on the findings)
    This has not happened before me, have they ever thought about autopsy or it has happened here. <Your views about post mortem> I do not know. Post mortem is also done in these cases like sending it to a mother who expires what the problem is why it has happened. <Child autopsy> did not happen to me. <Benefit> doctors know all those things, yes they would have got it done in the case in which the diagnosis is not known, what is the problem.

    In some instances, autopsy/ post-mortem is not possible due to various reasons. To find out the exact cause of death and underlying disease, some tissue biopsy (collection of very small sample using needles) and fluids (blood, urine, etc.) from the body, as done for biopsy. This procedure is called as Minimally Invasive Tissue Sampling (MITS).

13. **What is your view about MITS being used as a mode of identifying cause(s) of death in children/ newborn?**
    (Probe: feasibility, acceptability, perceived benefit, workload)
    Technology is good. <Acceptability> it depends on the parents that it will be that there will be no willing. In that case (where reason is not known), then it is fine which procedure you are telling. <Perceived benefit> will know what caused it. <workload> The workload is what it is, but it is that even if the samples are still going to be given, then they too will become extra together and they too will become.

14. **In your view, how the parents/ family members can be approached and convinced for MITS?**
    (Probe: Who/which health staff should approach, which family member should be approached, timing for informing, mode of communication, place of informing)
    <Timing for informing> It should be told again in the last condition that they can do this check, or if it is already done. They depend, they will be told, they depend on the parents whether they do it or not, it is their choice. Well, all this should be told only after death. <Which family member> Father should explain that if you do this test, the reason will be known.
    <Who / which health staff should approach> consultant also explains well, our SR also understands. <Mode of communication> by sitting comfortably, talking.

15. **In your view, what could be the expectations of parents/family members if they agree for MITS?**

(Probe: detailed report, convey of the findings, time, cost/money)
He will feel that yes brother, this procedure is good for me and it is beneficial for me, even if it will be done for the future. <Detailed report> Yes, they will get full details as to why this is happening.
<Convey of the findings> the doctor will say.

16. **What are the potential challenges/barriers for autopsy & MITS in the hospital setting?**
(Probe: procedural, space, manpower, workload, time needed, cost)
There will be no such problem, when you do so much work, it is also fine. From the parents' side, it is neither there will be any willing nor there will be no willing, because without parents, you cannot consent.
<Workload> for this, you can increase the manpower a little bit then you will suffer.

17. **Any other comment/ suggestions.**
No
