# Supplementary material for: Perceptions of the parents of deceased children and of healthcare providers about end-of-life communication and breaking bad news at a tertiary care public hospital in India: A qualitative exploratory study
Source: PLoS One. 2021 Mar 18;16(3):e0248661. doi: 10.1371/journal.pone.0248661 (PMC7971872; doi:10.1371/journal.pone.0248661)
Supplement: S3 File — (PDF) [file pone.0248661.s004.pdf]

Observations of deaths at the hospital

Case 1

A five months old male child named 'A' was admitted to hospital because of complaint of respiratory problem. The parents were residents of Uttar Pradesh and rushed with the child to a local doctor in the area. Seeing the child's critical condition, the local doctors referred him to this hospital. The child was accompanied by the father, mother, two paternal uncles, and four other male members from the locality and reached hospital at midnight. The child was put in ambulatory bag. After 10 hours of admission, with continuous monitoring and assessment, the child was not responding when checked by three Junior Resident Doctors in the morning round. The child's heartbeat was not felt by the stethoscope and so Junior Resident Doctor's asked a supporting staff to perform X-ray as a last attempt of assessment. Paternal uncle, who was only present at that time in the ward, anticipated death after the Junior Resident Doctors checked the child. Supporting staff asked the paternal uncle to hold the wire of the X-ray machine. Paternal uncle was crying and helped the supporting staff. After that he continued to pump the bag and tube for the child which he was doing even before the morning round. Paternal uncle looked tensed as the child's file was not found and he was enquiring the staff about the file. After 15 minutes, the child's father, who was talking to someone over phone, anticipated death by seeing no signs of response by the child to the medication given. Father then started informing other family members about the death over phone. Father asked two or three nursing staff present in the ward for the child's file but did not get any answer. Out of tension, father started crying and murmured "They (nurses) are not saying anything where is the child's file, I have come from a long distance, if they give the file then we will leave and have also not told my wife about the child's death". Meanwhile nurse came and asked the paternal uncle "where is the child's file, it is misplaced" and asked him to pump the bag and tube properly. A brother (male nurse) came to give medication to all the patients in the ward and asked the paternal uncle to hold an injection for him. Father again requested the Junior Resident Doctor to give the child's file, then Junior Resident Doctor shouted at the father and saying "Sit down calmly as I have asked a staff to prepare file (duplicate)". Father continuously cried and murmured "At evening we will reach and I have to arrange for the death rituals also". Nurse again asked paternal uncle to pump bag and tube properly. Father was asking continuously for baby's file to the nurses. Nurse responded to the father that "file work will be done by the doctor, this is her work, ask her, why have you come again, you go". Junior Resident Doctor and nurses were discussing silently among themselves that we can only declare once the file is found. Meanwhile Junior Resident Doctor again came and checked the child by stethoscope and declared death to the father by showing hand movement and said "Child has expired", after 50 minutes of the last assessment done. After declaration, father touched the Junior Resident Doctor's feet and asked to do the discharge quickly and started crying. Junior Resident Doctor rudely answered the father "how will you take the baby, file is not there, and has the ambulance been booked? She further said to the father that five-five doctors were engaged in checking your child". Junior Resident Doctor called the paternal uncle when she was filling the death form and mentioned Bronchiolitis, Respiratory distress increase, SPO2 decrease, Pneumothorax as cause of death in the death register. Paternal uncle started cried after declaration and informed other family members about the death. Nurse got paternal uncle's signature on death register and gave a death slip and asked him to show the slip if anyone asked about the dead body while travelling. Nurse and Junior Resident Doctor took out pipes/tubes from baby's body. Before leaving the ward, Paternal uncle asked other Junior Resident Doctor (not one who declared) about the cause of child's death. Junior

Resident Doctor replied him saying "No machine (ventilator) available in ICU". Father and paternal uncle wrapped the child and leave the ward towards the ambulance. Mother cried loudly when she saw the child wrapped in the arms of the father. Parents and all the attendants along with the child made the final exit from the hospital after 20 minutes of death declaration.

Case 2

Child M, 8 months old male child was diagnosed with a hole in the heart and was admitted in A Hospital in emergency. After a day at A Hospital, he was referred to S Hospital. Both father and mother accompanied the child, who were from Uttar Pradesh. On the day of admission of the child at S Hospital, doctors discussed the case and necessary medication was started by the health care providers. On the second day, after morning consultant round, nurse gave an injection to the child, which was the last attempt by the doctors to revive the child's heart. Father was enquiring about his child condition from the doctors even before the rounds. After the assessment, the nurse said to the father that after 20 mins they will re-observe the condition of the child, if he is responding to the injection or not. Parents started anticipating the child death as child was not responding at all to the injection. Mother started crying and continuously pumping the bag and tube while the father was silent and looked angry. Father was again and again enquiring about the child's condition to the Senior Resident Doctor (SR). SR pumped the chest of the child by hand and declared death to the father after 15 minutes of the last assessment done. SR declared to the father saying that "child's heartbeat is not felt and has expired". SR removed the pipes/tubes from the child's body. Mother started crying badly while the father did not show any emotion (looked angry) and packed his bag. Father asked SR "What has to be done now?". SR responded that after cleaning the body and documentation you can go with the child. SR then asked the junior doctors and nurses to prepare the discharge papers. After listening to the SR, nurse smiled and asked the SR in the presence of the father that "Who has declared? You", and then asked the father to wait and get the paper work done. Nurse got signature in death register, gave death slip to show to the police if they enquire about the body, and asked to collect the death certificate from the hospital after 15 days or a month. Nurse advised the father to go to CMO emergency office for ambulance aid. Father asked the nurse to give the whole file of the child. Nurse said that the file cannot be given. Then father refused the nurse to clean the child's body and took the child in his arms and left the ward with his wife. No other family member was present outside the ward. Parents did not receive any ambulance aid from emergency control room officer as no ambulance was available at that point of time. Mother sat down and continuously cried and the father carrying the child looked helpless to arrange for vehicle and was calling someone over phone, probably to inform about the child death to the other family members. Twenty five minutes after declaration of death, the parents made the final exit from the hospital with the child.

Case 3

Child A, a two and half year old girl child suffered from fever from past two days and suddenly collapsed while returning with her mother from the market. The girl asked her mother to pick her up in her arms just before collapsing. The parents of the child were from U.P and immediately rushed to nearby local hospital where the child was declared brain dead but heartbeat was detectable so the local hospital referred the girl child to S hospital. The child was accompanied by her father, mother, grandmother, father's cousin, and two neighbors. The child was declared brain dead, but with detectable heartbeat, at admission in S Hospital, and was put in ambulatory bag to prevent respiratory distress. After two days of assessment and medication, the child did not

respond and no signs of improvement were seen. On the third day, Junior Resident Doctor checked the vitals and examined the child and discussed about the case with the senior resident (SR). SR checked the child by stethoscope and moved to see other patients. After the SR checked the child, father anticipated the death of his daughter and told one of his friends that child has expired. Father's friend continuously pumped bag and tube. SR came and checked the child again by stethoscope and declared death to the Junior Resident Doctor by nodding the head. Junior Resident Doctor then declared to the father by nodding head and by hand movement after 11 minutes of the last assessment done. Then the father and his friends started informing other family members about the death over phone. No one was crying among the attendants, including the father, as they were mentally prepared about the death. Twice the father took out tobacco and rubbed on his hand and had it while discussing with his friends about the delay in discharge of the child from the hospital. He looked tensed about how he will manage to do the death ritual arrangements on time. Meanwhile, Junior Resident Doctor informed to father's friend that discharge papers will be prepared after 5-7 minutes and mentioned respiratory failure with cardio-respiratory arrest, brain dead as the cause of death in the death register. On the other hand, father told his friend that it would have been better if child was admitted in private hospital. Suddenly, the child's mother came inside the ward (who was in the waiting area) to enquire about the child's condition but the father sent her back to the waiting area saying that the child is doing well. When the mother left, then the father said his friend that "She might have fainted, so I sent her back downstairs". Father then spoke to Hindu religious leader (Pandit) over phone about the death rituals, and disposal of the child's body, for that the Hindu religious leader advised him to dispose the body in Yamuna River and do not bring the body at home. Father discussed with his friend about death rituals saying that "child should not be cremated (burning body in fire)" and then asked him to arrange for ambulance. Father then went downstairs to fetch some money from his wife. Friends discussed among themselves that "we had lots of hope, when we came here". After continuous reminder to the nurse to tape the child's body, one of the father's friends was tired and said out of frustration "I am tired of telling the nurses to tape the child's body". Nurse told to father's friends that "Taping the body was not her work and that the supporting staff had gone to blood bank, he will do the taping after he returns. Nurse further said "They have a shortage of staff and so attendants should take the body of their own". Nurse then gave death slip to one of the father's friends and covered the child's face. A supporting staff (male) wrapped the child and taped her covering the body perfectly. For carrying the child downstairs, father's friends asked the nurse "Is it necessary to carry the child's body downstairs in trolley? Can't I carry the child in hands". The nurse then asked him to submit a ID and take trolley as taking child body in arms is not allowed. After listening to this the friend said "What kind of hospital is this?" Father's friend then asked for a trolley from the ward boy who tapped the body. First the ward boy refused saying that the trolley is not available then on continuous request by the father's friend, ward boy agreed and took the body in trolley till the ambulance area. On seeing the child's body, mother and grandmother started crying and the father was consoling them specially the mother, mother came holding the hand of the father. Father asked the friend to show the child's face to the mother for the last time. Father walked with the mother and grandmother to the exit door of the hospital. Father's friends took the body and left the hospital in ambulance. One hour after declaration of death, parents and all the other attendants made the final exit from the hospital with the child.

Case 4

Baby P, a three day old newborn (male) was admitted due to fever at S hospital. Baby was delivered at home when no one was around the mother at the time of delivery. After admission, Baby was diagnosed with Pneumonia and was put on ventilator. Baby was accompanied by father mother, paternal aunt, and two male members from the family. For two days, continuous monitoring and treatment was provided to the baby. On the third day of admission, doctors including Junior Resident Doctor, SR, and Consultant discussed about the case in the morning round. During the round discussion, it was noted that the baby was not responding to the treatment. SR checked the baby by stethoscope and gave medicine as an attempt for final assessment. Father was informed by the doctor (SR) that "Baby's heartbeat is not felt and not to keep any hope from the baby". Father anticipated death of the baby as the SR said the chances of baby's survival was less. After 50 minutes of last assessment done, Junior Resident Doctor declared to the father and paternal aunt saying that "Baby has expired. Medicine was given to the baby which usually revives the heartbeat again but baby is not responding to the medicine. Baby's heartbeat is not felt and baby is not breathing. We could not save your baby". Junior Resident Doctor went to her desk and mentioned Premature, respiratory distress, pneumonia as the cause of death of the neonate in the death register. Father informed other family members over phone that baby was no more and said to his sister (paternal aunt) to stay in the ward as he will go out to buy a towel to wrap the baby and will send his wife home (who was outside the ward) saying that baby's condition was critical so the baby has been shifted to ICU. Paternal aunt sat near the baby and started crying. For completing documentation, nurse enquired about the baby's father once. Two male members came to visit the baby and console the paternal aunt in the ward. Nurse again enquired about the father of the baby to complete the paper work. Father came after an hour with the towel. Nurse assisted him in completing the documentation formalities, i.e. death form, death slip. Father wrapped the body with towel and left with his sister towards the hospital exit. Several men waiting outside the ward discussed with father and paternal aunt where to bury the baby's body. After one hour twenty minutes of death declaration, father and the attendants leave the hospital with the baby.

Case 5

Baby S, a four day old newborn (male) was admitted at S Hospital due to respiratory distress. The neonate was born at MN hospital with respiratory distress and was referred to S Hospital. The parents were residents of Delhi and the baby was accompanied by his father, mother, maternal grandmother, maternal aunt and maternal uncle. The baby was put in ambulatory bag at admission. After five days of monitoring and treatment, the baby showed no signs of improvement. On sixth day morning, the baby stopped showing body movements, became quite and closed his eyes when the mother, father and maternal grandmother were present beside the baby. Mother immediately called the Junior Resident Doctor to check the baby. Junior Resident Doctor came and checked the vitals of the baby and asked the mother to pump the ambulatory bag continuously. Mother anticipated death of her baby and started crying. Father and maternal grandmother tried to console the mother saying "nothing has happened to the baby, he is breathing, please stop crying" and sent the mother outside the ward. Three hours after mother anticipated death of her baby, SR did the last attempt of assessment by pressing the chest of the baby while the Junior Resident Doctor, nurse, brother (male nurse) stood by her side for help. After the assessment, SR told the father that "All medicines were provided to the baby but then also heartbeat is going down". Maternal grandmother started crying because the baby was not responding to the medication and left the ward. Father was continuously pumping bag and tube, while mother, maternal uncle and aunt came

back to the ward. After half an hour of last assessment, SR declared death to the father in front of mother and other attendants. SR declared by saying “baby is no more”. Mother started crying loudly and maternal uncle and aunt consoled her and took her out of the ward. On the other hand, the father had tears in his eyes, looked sad and gently caressing the baby. Maternal uncle, maternal grandmother came inside the ward crying for the baby. On listening to the loud cry, nurse asked Junior Resident Doctor in front of the father that “doctor have you declared?” and then said the father “Let the doctor come and prepare the death documentation, then you can take the baby home”. Father asked the nurse for ambulance aid. Nurse responded that ambulance can be arranged from the CMO office. Father started informing other family members about the death of the baby over phone. Brother (male nurse) took out all the pipes from the baby’s body and nurse gave the death slip to the father. SR filled the death form and mentioned respiratory failure, hypoglycemia with Meningitis as the cause of death in the death register. Father continuously cried, packed his bag, closed the eyes of the baby and wrapped him in a blanket. Brother (male nurse) then wrapped the baby properly by blue disposable sheet and handed over the body to the father. Father along with maternal uncle left the ward after he could not arrange for ambulance. Forty five minutes after declaration of death, father and other attendants hired a local vehicle and made the final exit from the hospital with the baby.

Case 6

Baby N, a twenty seven day female neonate was admitted at S Hospital due to Jaundice, and extreme swelling of the stomach as the baby aspirated meconium when delivered at S Hospital. After baby’s stomach got swelled at home, parents visited local doctor where they referred the baby to A Hospital. Due to lack of incubator facility, A Hospital further referred the baby to S Hospital. After the delivery of the baby, doctors at S Hospital told that baby was born with hole in her heart. Both the father and the mother accompanied the baby. During six days of monitoring and medication of the baby, the parents were informed by the doctors about less chances of survival of the baby. Baby was put in ambulatory bag which was pumped by the parents continuously, especially by the mother. On the sixth day of admission, mother asked SR “Why the swelling of the stomach is not reducing?” SR responded to the mother that “how will it reduce, as her liver has enlarged”. Father asked the SR “Can’t we go for the operation”. SR responded to father that “No, you have come too late for the operation. Baby’s liver has already damaged, we did white blood cell transfusion also, but baby’s condition is critical”. Junior Resident Doctor also came to check the baby and father again asked her “Why the swelling of the stomach is not reducing?” Junior Resident Doctor responded that now the baby’s condition is comparatively better than before, now her body position is also alright”. That day afternoon, father had an argument with the guard when the guard said “When I stopped you from coming inside the ward, you said that you will come back after a while. Is this a park where you are roaming?” Father then replied the guard saying “We have not come here on our own will”. After that a consultant came for rounds, where the baby’s case was discussed. The consultant asked the SR to get a pediatric surgeon’s opinion. SR asked nurse if ADR was given to the baby. Then a pediatric surgeon came and said “This is a case of Ascites, why surgery should be performed in this case?” and asked the Junior Resident Doctor to send the sample for test. On the seventh day of admission, before the morning rounds, SR was pressing the chest of the baby and Junior Resident Doctor was standing on her side for any help. SR then asked the nurse to give ADR (to revive the heart) to the baby as a last attempt of assessment. Both the parents anticipated death of the baby, and so the mother started crying badly while the father was silent and pumped the Ambu bag. Father asked the mother to go out of the

ward. After 35 minutes of last assessment done, SR declared death to the father saying that "Baby's heartbeat has stopped beating, we have given medicine but heartbeat could not be revived. Baby was suffering a lot, and we even did white blood cells transfusion, but it did not help". Father started crying and informed other family members about the death. Brother (male nurse) started removing pipes and Ambu bag from the baby. Nurse wrapped the baby's body by blue disposable sheet and handed over to the father. Nurse asked the father to sign on the death register, so that we can provide you the death slip. SR mentioned neonatal cholestasis with septic ileus with Meningitis as the cause of death in the death register. After the father has signed in the death register, nurse gave the death slip to the father and then he left the ward. Mother was standing outside the ward and cried badly. Father then asked his wife to come with him to the exit gate. Thirty five minutes after declaration of death, both the parents made the final exit from the hospital with the baby in a public vehicle.

Case 7

Baby M, a two day old female neonate was admitted in S Hospital with low birth weight issues after been referred from Faridabad, Haryana. The parents were residents of Faridabad, and baby's mother had a history of anemia during her pregnancy. At admission, the baby was brought in infant incubator and was accompanied by her father, mother, and paternal grandmother. After two days of monitoring and medication, baby did not show any signs of improvement. On third day morning, baby was given an injection by the nurse on duty. During the morning round, consultant came and checked the baby and discussed the case with the Senior Resident Doctor (SR) and moved on to next patient in the ward. Father pumped the bag and tube for the baby and then called the Junior Resident Doctor to check the baby's condition. Both father and mother anticipated death of the baby and the mother started crying. Junior Resident Doctor on request of the father, checked the baby but did not tell them anything about the condition and left. After a couple of minutes, SR also checked the baby, and without talking to the parents moved to see other patients in the ward. Junior Resident Doctor started preparing the baby's file and documentation by saying to other colleagues "We will declare after the papers are ready". On the other hand, grandmother consoled the mother, as she was crying continuously. While the father continuously pumped the bag and tube. Junior Resident Doctor then called the father near the doctors table in the same ward and in front of him, the SR told the Junior Resident Doctor that "first you declare death to father, then you do all the other work". After 55 minutes of parent's anticipation of baby's death, SR declared death to the father in a polite manner saying "baby is no more" and further advised the father to take the mother out of the ward. After declaration, nurse provided the death slip to the father and asked him to take help of other nurses to remove the cannula from baby's body. Mother and grandmother started crying badly and mother was consoled and taken out of the ward by the grandmother. Father was also crying holding the baby when the brother (male nurse) came to remove the cannula. In middle of the work, brother left the baby without cleaning the baby and moved to give injection to other child. Father waited for the brother (male nurse) to come back. Blood was coming out from the baby's body but no health care provider reached for help. One of the patients' father provided cotton balls to the father to block the blood coming out of the baby's body. Father continuously cried silently holding the baby and wrapped her in a new towel. One of the patient's mother consoled the father saying "What happened to the baby suddenly, this kind of things happens in this hospital, you should take care of your wife and mother at this time". Nurse completed the documentation work while SR mentioned low birth weight as cause of death in the death register. Maternal grandfather and grandmother, and maternal aunt came to see the baby in

the hospital, and after seeing them the mother burst out crying loudly and said “my girl has left me, she left me mom”. Both the parents and the relatives; maternal grandfather, grandmother, aunt cried a lot. Maternal grandfather then asked the nurse if he can take the baby home as they have received the death slip. Nurse responded saying that “Yes, you can take baby home and show the death slip if someone asks you about the baby in exit gate of the hospital”. Guard stopped the maternal grandmother and aunt to enter the ward as so many members were not allowed to visit at a time. Mother kept on crying and blamed the doctors saying that “ my girl child has expired, I had told the doctor to check my baby, but the doctor did not come and see, my baby caught cold”. Maternal grandfather wrapped the baby and consoled the mother and asked her to stop crying. While they all were leaving the ward, the guard said to the maternal grandfather “Uncle, Have you got the slip? You take the baby home, the death of the baby was in destiny”. Maternal grandmother took the baby in her arms and all of them left the ward crying. Maternal grandfather hired a private ambulance and one hour and forty minutes after declaration of death, parents and all the attendants left from the hospital with the baby.

Case 8

Baby S, an eight day old male neonate was admitted to S Hospital due to less detectable heartbeat. The baby was delivered in a private hospital in Delhi, with very less heartbeat felt since his birth and was referred to S Hospital. The baby was put in ambulatory bag at admission and was accompanied by his paternal grandfather and maternal uncle. After four days of monitoring and treatment, baby did not show any signs of improvement. On the fifth day of admission, after the morning rounds, baby was checked by the SR but did not tell anything to the attendants. Then the intern came and noted the vitals of the baby on the information sheet attached to the bed. Ambulatory bag was pumped by the paternal grandfather and maternal uncle turn-wise. Paternal grandfather enquired about the condition of the baby when the Junior Resident Doctor came to check the baby. Junior Resident Doctor did not respond and left. After the last assessment done by the Junior Resident Doctor, the attendants pumped the bag and tube for three and half hours. Consultant came in afternoon round and asked the SR to perform suction after checking the baby. SR performed suction and the Junior Resident Doctor, and brother (male nurse) stood by her side for any help. SR then informed the paternal grandfather that “Baby’s condition is critical, we are trying”. After an hour, SR again checked the baby and said to the paternal grandfather that “uncle baby’s heartbeat is beating very slowly”. SR pressed the baby’s chest by her hand and then asked the nurse to give adrenaline (injection to revive the heart). Maternal uncle anticipated death of the baby and so asked the SR to check baby’s heartbeat. SR responded to him “we are checking the baby, medicine is given, let’s see, heartbeat is very less, now I can’t tell anything about the baby’s condition, after sometime I can tell you, as of now heartbeat is very less. You don’t stand like this, take a stool and sit down properly and then pump the bag and tube”. After the SR went, maternal uncle checked the heartbeat of the baby by himself and started crying while pumping the bag and tube. In evening visiting hours, maternal grandfather come to visit the baby. Paternal grandfather asked the Junior Resident Doctor to check the baby once. SR told the Junior Resident Doctor “If you want to declare to the attendants then declare”. Then the Junior Resident Doctor asked other Junior Resident Doctor “Prepare the file, file is misplaced, sister look for the file”. After 30 minutes of last assessment, Junior Resident Doctor declared death to the maternal uncle by saying “Stop pumping the ambu bag, the baby is no more, baby did not cry when he was born so, if he would have survived then also he would not have been a fit baby, I will prepare the discharge papers”. After declaration, maternal uncle and paternal grandfather started crying and both of them left the

ward. Junior Resident Doctor asked the intern present there to look for the file. Intern searched for the file and got it on baby's bedside. Nurse completed the documentation, while the Junior Resident Doctor mentioned cardiopulmonary arrest as a cause of death in the death register. Maternal grandfather signed on the death register and nurse gave him the death slip to show in the exit gate. After the nurse took out the cannula, she asked the maternal grandfather to keep the cotton on the cannula area to control the bleeding. Nurse wrapped the baby's body with blue disposable sheet and handed over to the maternal grandfather. Maternal grandfather wrapped the baby further and left the ward with the baby. Maternal uncle accompanied the maternal grandfather to the main exit gate where the paternal grandfather was standing with his bag packed. Thirty five minutes after declaration of death, all the attendants left the hospital with the baby in a public vehicle.
