# Supplementary material for: Perceptions of the parents of deceased children and of healthcare providers about end-of-life communication and breaking bad news at a tertiary care public hospital in India: A qualitative exploratory study
Source: PLoS One. 2021 Mar 18;16(3):e0248661. doi: 10.1371/journal.pone.0248661 (PMC7971872; doi:10.1371/journal.pone.0248661)
Supplement: S4 File — (PDF) [file pone.0248661.s005.pdf]

Coding Tree

1. Communication during hospital care
- 1.1. Attitude
- 1.2. Body language
- 1.3. Words used and appropriateness
- 1.4. Sympathy
- 1.5. Frequency and completeness
- 1.6. Clarity and conflicts in messages
- 1.7. Blame

2. Process of death declaration
- 2.1. Attitude
- 2.2. Language and words used
- 2.3. Sympathy
- 2.4. Time spent with parents

3. Respect and dignity for patient's family
- 3.1. Inappropriate behaviour
- 3.2. Differential behaviour
- 3.3. Time constraint
- 3.4. Period of hospital stay

4. Emotional support for family
- 4.1. Consoling support
- 4.2. Support for mothers

5. Services and care received
- 5.1. Experience based behaviour by doctors
- 5.2. Blame of negligence by HCPs
- 5.3. Perception about life support interventions
- 5.4. Staff shortage
- 5.5. Permission for family and attendants

6. Experience of HCPs
- 6.1. Prognostication and counselling
- 6.2. Acceptance of death by family
- 6.3. Emotional affect by death declaration
- 6.4. Coping the stress
- 6.5. Communication competency
- 6.6. Workload and time challenge
